# Arylsulfamates inhibit colonic Bacteroidota growth through a sulfatase-independent mechanism

**DOI:** 10.1073/pnas.2414331122

**Published:** 2025-07-10

**Authors:** Conor J. Crawford, Charles W. E. Tomlinson, Christian Gunawan, Zongjia Chen, Dominic P. Byrne, Cosette Darby, Martina L. G. Conti, Tony Larson, Ana S. Luis, Stefano Elli, Edwin A. Yates, David N. Bolam, Sjoerd van der Post, Spencer J. Williams, Alan Cartmell

**Affiliations:** ^a^Department of Biomolecular Systems, Max Planck Institute of Colloids and Interfaces, Potsdam 14476, Germany; ^b^Department of Biology, University of York, York YO10 5DD, United Kingdom; ^c^York Structural Biology Laboratory, Department of Chemistry, University of York, York YO10 5DD, United Kingdom; ^d^York Biomedical Research Institute, University of York, York YO10 5DD, United Kingdom; ^e^School of Chemistry and Bio21, Molecular Science and Biotechnology Institute, University of Melbourne, Parkville, Victoria 3010, Australia; ^f^Department of Biochemistry, Cell and Systems Biology, Institute of Systems, Molecular and Integrative Biology, University of Liverpool, Liverpool L69 7ZB, United Kingdom; ^g^Biosciences Institute, Faculty of Medical Sciences, Newcastle University, Medical School, Newcastle upon Tyne NE2 4HH, United Kingdom; ^h^Department of Medical Biochemistry and Cell Biology, University of Gothenburg, Gothenburg 405 30, Sweden; ^i^SciLifeLab, University of Gothenburg, 41390 Gothenburg, Sweden; ^j^Istituto di Ricerche Chimiche e Biochimiche G. Ronzoni, Milano 20133, Italy

**Keywords:** sulfatases, arylsulfamates, complex glycans, gut microbiota, drug discovery

## Abstract

Excessive degradation of the colonic mucin layer by *Bacteroides* within the human gut microbiota drives inflammatory bowel disease (IBD) in mice. Bacterial carbohydrate sulfatases are key enzymes in gut colonization, and they are elevated in human IBD and correlate with disease severity. Selective inhibitors of carbohydrate sulfatases could function as sulfatase-selective drugs, allowing precise control of sulfatase activity while preserving these otherwise beneficial bacteria. Arylsulfamates are covalent inhibitors that target a catalytic formylglycine residue of steroid sulfatases, a residue that is also conserved in carbohydrate sulfatases. Here, we find that a library of aryl- and carbohydrate sulfamates is ineffective against carbohydrate sulfatases, yet can inhibit human gut microbiota (HGM) species grown on sulfated glycans. Leveraging thermal proteome profiling (TPP), we identify a lipid kinase as the target responsible for these effects. This work highlights the imperative for developing specific inhibitors targeting carbohydrate sulfatases and reveals the adverse effects that arylsulfamates have on *Bacteroides* species of the HGM.

## Significance

Arylsulfamates are currently the only effective class of sulfatase inhibitors available and offer a potential strategy to treat inflammatory bowel disease (IBD) driven by gut microbiota carbohydrate sulfatases. Although arylsulfamates inhibit the growth of microbiota *Bacteroides* species on sulfated glycans, this is not mediated through carbohydrate sulfatases but via a conserved lipid kinase. Carbohydrate sulfatases are resistant to arylsulfamates while steroid sulfatases are susceptible despite a conserved active site. Finally, selected complex plant glycans confer a resistant/protective phenotype against the harmful effects of arylsulfamates. These data guide the future development of targeted carbohydrate sulfatase inhibitors and potential drug–prebiotic pairings.

The human gut microbiota (HGM) is a microbial community found throughout the gastrointestinal tract but is densest in the distal colon where it is composed of trillions of bacteria. This community is critical to human health, providing essential vitamins (1, 2), such as soluble B vitamins, calories through short chain fatty acid production (3), immune system regulation (4, 5), and production of metabolites that influence the gut–brain axis (6, 7). Fermentation of complex carbohydrates underpins all these processes; the complex carbohydrates being derived either from dietary fiber (plant glycans) or the host. In the latter case this involves mainly sulfated glycans from colonic mucin and glycosaminoglycans.

The colonic mucin layer is the most abundant host glycan in the colon. It is composed of a gel forming glycoprotein, MUC2, is 80% glycan by mass and is heavily sulfated (8). This layer has many biological functions, and provides a colonizable niche and food source for colonic bacteria, while simultaneously acting as largely impenetrable barrier protecting the colonic epithelium (9, 10). The Bacteroidota phylum are the major glycan degraders present in the HGM and are enriched in carbohydrate sulfatases, which are essential enzymes for the utilization of sulfated host glycans. In host symbiosis the model Bacteroidota species *Bacteroides thetaiotaomicron* VPI-5482 (*B. theta*), grazes on colonic mucin *O*-glycans in a carbohydrate sulfatase-dependent process (11). However, in dysbiosis, excessive degradation of the sulfated colonic mucin drives inflammatory bowel disease (IBD) and carbohydrate sulfatases are the enzymatic drivers of this effect in a “friend turned foe” scenario (12–14). Excessive inflammation caused by loss of the mucin barrier is also a risk factor for colon cancer (15), the second leading cause of cancer deaths. Thus, effective inhibitors of HGM carbohydrate sulfatases are of high medical relevance.

**Fig. 1.**
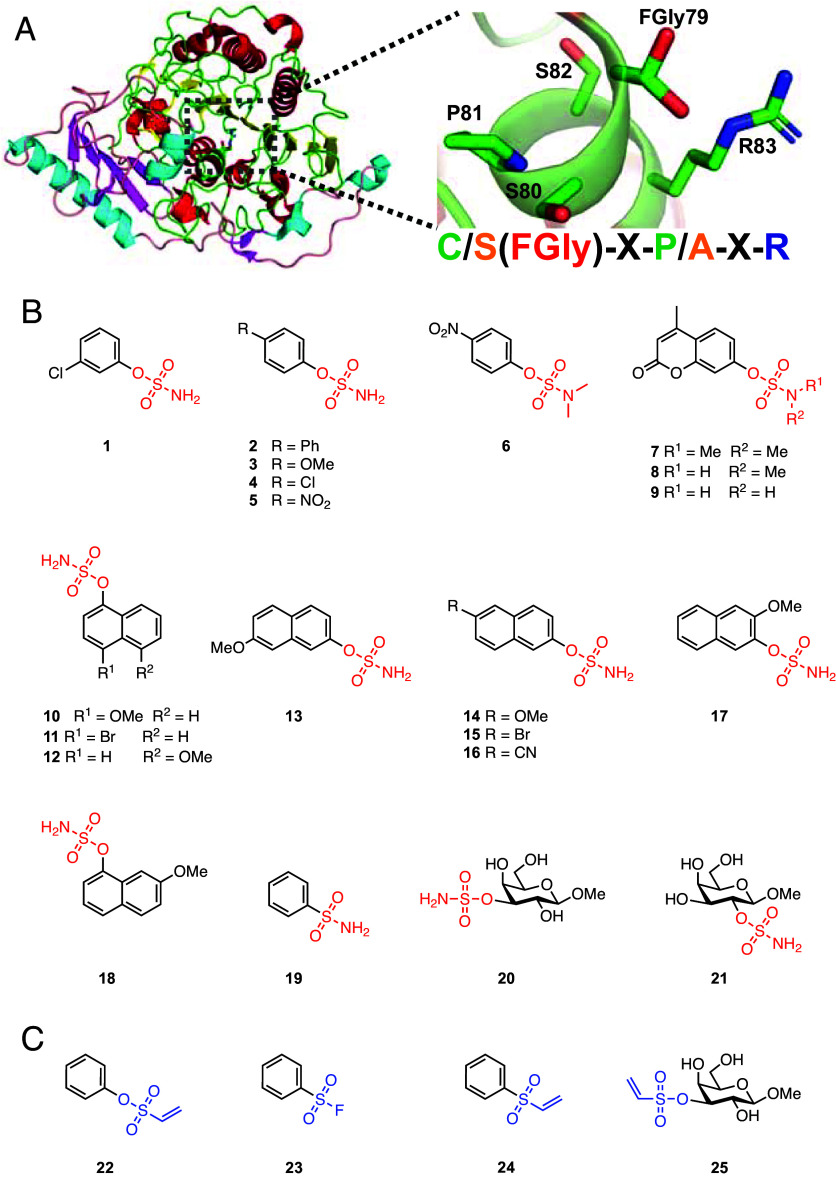
Example of the conserved S1 sulfatase fold, consensus active site sequence, and tested library of aryl- and carbohydrate sulfamates. (*A*) 3-D structure of human lysosomal sulfatase GALNS (PDB:4FDJ). The N-terminal domain is colored red and yellow for alpha helices and beta sheets, with the C-terminal subdomain colored cyan and magenta for alpha helices and beta sheets. Zoom shows the consensus sequence of GALNS with a formylglycine residue, and with the general consensus pictured to the *Right*. (*B*) Structures of aryl- and carbohydrate sulfamate inhibitors used in this study. (*C*) Structures of phenylvinyl sulfone, phenylsulfonyl fluoride, phenylvinyl sulfonate, and carbohydrate vinyl sulfone.

Sulfatases are classified into four families, S1, S2, S3, and S4 (16). Of the over 160,000 sequences cataloged in the SulfAtlas database, the majority (over 90%, or >145,000 sequences) belong to the S1 family (17). The S1 family occurs across all domains of life and is currently the only family that contains sulfatases able to desulfate carbohydrates (16, 18, 19). Members of the S1 family of sulfatases have a conserved alkaline phosphatase-like fold characterized by a larger N-terminal domain housing a central mixed β sheet flanked by α helices. This domain abuts a smaller C-terminal subdomain composed of a 4-stranded antiparallel β sheet and a single α helix (20) (Fig. 1*A*). Within this structural framework, the S1 family employs a catalytic formylglycine (FGly) residue, which is generated cotranslationally from a Cys or Ser within the consensus sequence **C**/**S**-X-P/A-X-R, and is situated within an invariant sulfate binding (S) site (18) (Fig. 1*A*).

The major class of sulfatase inhibitors are the arylsulfamates, with the general formula Ar-O-SO_2_NH_2_. These inhibitors, developed by replacing the sulfate group of assorted aryl sulfate substrates with a sulfamate group, are irreversible, active-site directed inhibitors that are broadly effective against steroid sulfatases of both bacterial and eukaryotic origin (21). Clinical development of arylsulfamates targeting human steroid sulfatase (STS) for breast cancer treatment has advanced to phase II clinical trials, with positive outcomes (22–24). Arylsulfamates work by targeting the unusual catalytic FGly found in the invariant S site of S1 family of sulfatases and are believed to be paninhibitors of the family (25) but this has not been systematically tested.

Here, we investigate the ability of a panel of aryl- and carbohydrate sulfamates/sulfonates, as well as vinyl sulfones and a sulfonylfluoride (Fig. 1 *B* and *C*), to inhibit the growth of S1 sulfatase-containing Bacteroidota bacteria on sulfated glycans, and to directly inhibit recombinant and lysate derived carbohydrate sulfatases from these species, with the goal of developing targeted strategies to treat IBD. Our data revealed that sulfamate based inhibitors inhibit the growth of *Bacteroides* species on sulfated glycans but, unexpectedly, do not inhibit any of the carbohydrate sulfatases from these organisms. Leveraging thermal proteome profiling (TPP) we identify lipid kinases, and not sulfatases, as the targets through which arylsulfamates mediate their effects. We also show that arylsulfamate inhibition of growth can be overcome through the utilization of select, nonsulfated, plant complex glycans. Our data suggest that arylsulfamates may have potential for the treatment of IBD but at the expense of *Bacteroides* species, while also uncovering potential prebiotic strategies to protect *Bacteroides* species of the HGM from arylsulfamate drugs when they are delivered orally to treat cancer, or IBD.

## Results

### Arylsulfamates Inhibit Growth of *B. thetaiotaomicron*.

**Fig. 2.**
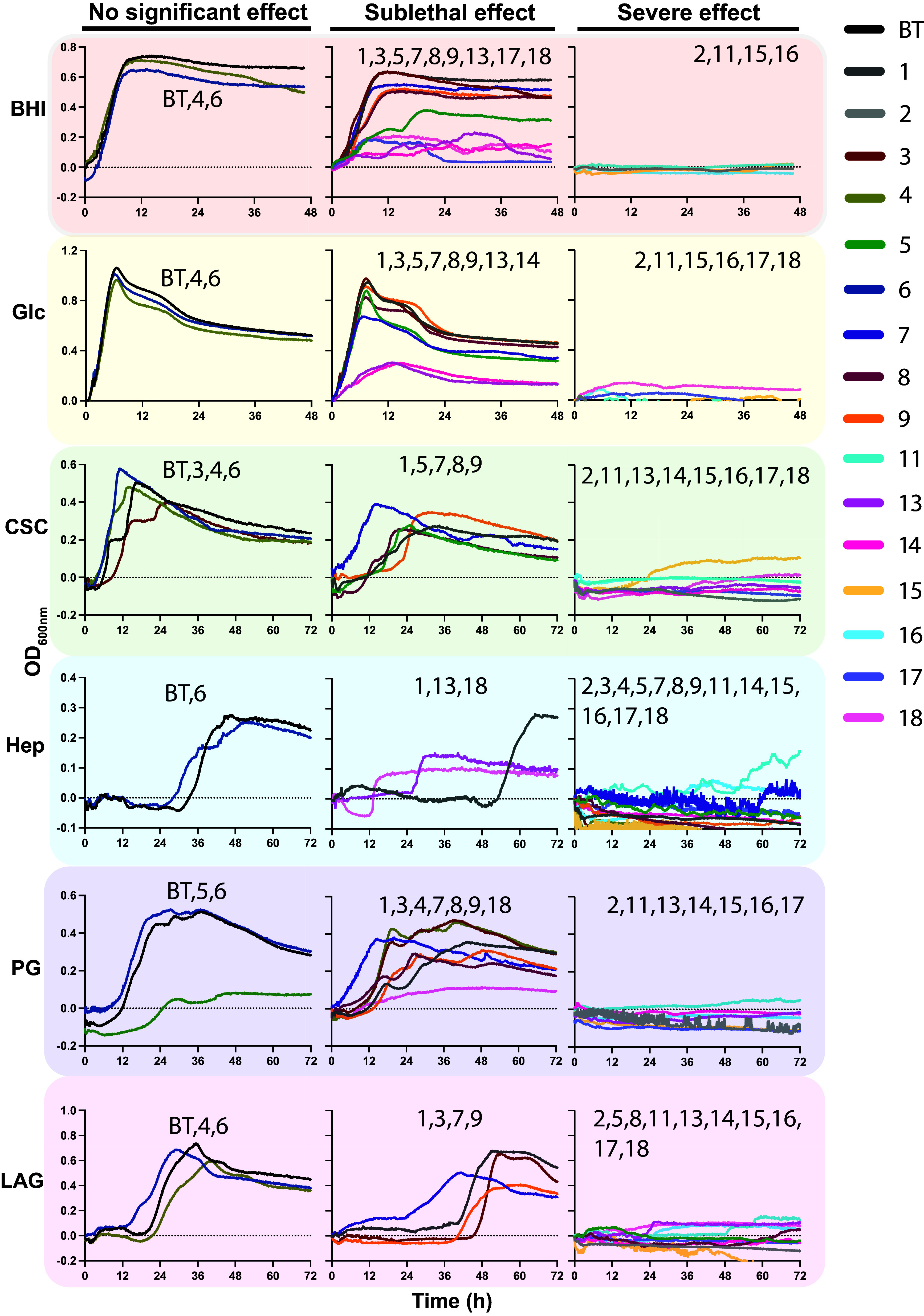
Growth of *Bacteroides thetaiotaomicron* on varied carbon sources in the presence of arylsulfamate inhibitors. *B. theta* grown on varied carbon sources; two rich carbon sources, brain heart infusion media (BHI) and glucose (Glc); two sulfated host glycan carbon sources, chondroitin sulfate C (CSC) and heparin (Hep), and two plant glycan carbon sources, potato galactan (PG) and larch arabinogalactan (LAG), in the presence of arylsulfamates inhibitors 1–18 (Fig. 1). Complex glycans and arylsulfamate inhibitors were used at a concentration of 5 mg/ml and 1 mM, respectively. All carbon sources were utilized in minimal media except BHI, which was purchased from Sigma and dissolved in water. The traces are the mean of 3 independent growth experiments, and error bars have been omitted for clarity; individual graphs with error bars are presented in *SI Appendix*, Figs. S2–S7. Severe effects were judged qualitatively on the basis that no classically identifiable features of a bacterial growth could be measured: a lag and mid-exponential growth phase leading to robust increases in OD_600nm_. For sublethal growth curves that displayed the aforementioned classical features, and were measurable, a two-tailed unpaired *t* test was performed to check for significance at a threshold of *P* < 0.05 (*SI Appendix*, Tables S1 and S2). A difference was called significant if it met this threshold in any one of three criteria: lag phase, growth rate, and max O.D. reached.

*Bacteroides* species of the HGM encode large numbers of S1 sulfatases, which are induced during growth on sulfated glycans. The model organism *B. thetaiotaomicron* VPI-5482 (*B. theta*) encodes 28 S1 sulfatases, 16 of which have been characterized as carbohydrate sulfatases. We hypothesized that growth of *B. theta* would be affected by the action of arylsulfamates when grown on sulfated glycans, but not on unsulfated glycans. Therefore, we cultivated *B. theta* in rich brain heart infusion media, and in minimal media supplemented with sulfated chondroitin sulfate C (CSC) and heparin and unsulfated [D-glucose, larch arabinogalactan (LAG), and potato galactan] carbon sources, each in the presence of a panel of arylsulfamate compounds at 1 mM (Fig. 2). This panel includes three control compounds: *N-*methyl and *N,N*-dimethyl sulfamates **6–8**, which should not inhibit S1 sulfatases. DMSO was used at a final concentration of 1%, which did not affect growth of *B. theta* (*SI Appendix*, Fig. S1).

We observed variable growth rates for *B. theta* with different arylsulfamate and carbon source combinations (Fig. 2 and *SI Appendix*, Figs. S2–S7). Arylsulfamates based on a naphthyl scaffold (compounds **11–17**) caused severe growth defects on all substrates. In contrast, the effect of arylsulfamates based on a substituted phenyl group (1, 3–6) varied substantially. *B. theta* displayed mild growth defects in the presence of these compounds when grown on brain heart infusion media and glucose, while growth on the sulfated host glycan CSC, and the unsulfated plant glycan potato galactan, was strongly perturbed by **1** and **5**. Compound **3** induced an increase in lag phase on CSC, and compound **4** caused complete inhibition of growth on the sulfated host glycan heparin (Fig. 2). On the unsulfated plant glycan LAG, **1** and **3** caused extension of *B. theta* lag phase while **5** completely inhibited growth. Compound **2**, a 4-phenylphenyl sulfamate, inhibited growth on all substrates. For the coumate scaffold, compounds **7-9** had only mild effects when *B. theta* was grown on brain heart infusion media and glucose. However, they increased the lag phase and reduced growth on CSC, and completely inhibited growth on heparin. Compounds **3** and **7** increased lag phase and reduced growth on LAG, while compound **8** completely inhibited growth. When potato galactan was used as the carbon source **3** caused a mild growth defect while **7** reduced the lag phase; **8** caused a significant defect but still allowed growth.

The variable impact of arylsulfamates on the growth of *B. theta*, observed for both sulfated and unsulfated glycan substrates, suggests that these compounds do not target S1 carbohydrate sulfatases. Additionally, the fact that the three negative control methylated sulfamates (compounds **6–8**) also influenced growth of *B. theta* provides further support that the observed growth defects are not mediated through an S1 sulfatase–dependent mechanism. A significant finding is that the same arylsulfamate structure has a different effect depending on the carbon source utilized. Brain heart infusion media, and the nonsulfated substrates potato galactan and glucose in a minimal media context, appear to switch *B. theta* to a more resistant/protective metabolic state evinced by milder phenotypes and fewer severe effects.

### Arylsulfamates do not Inhibit HGM *B. theta* S1 Carbohydrate Sulfatases.

**Fig. 3.**
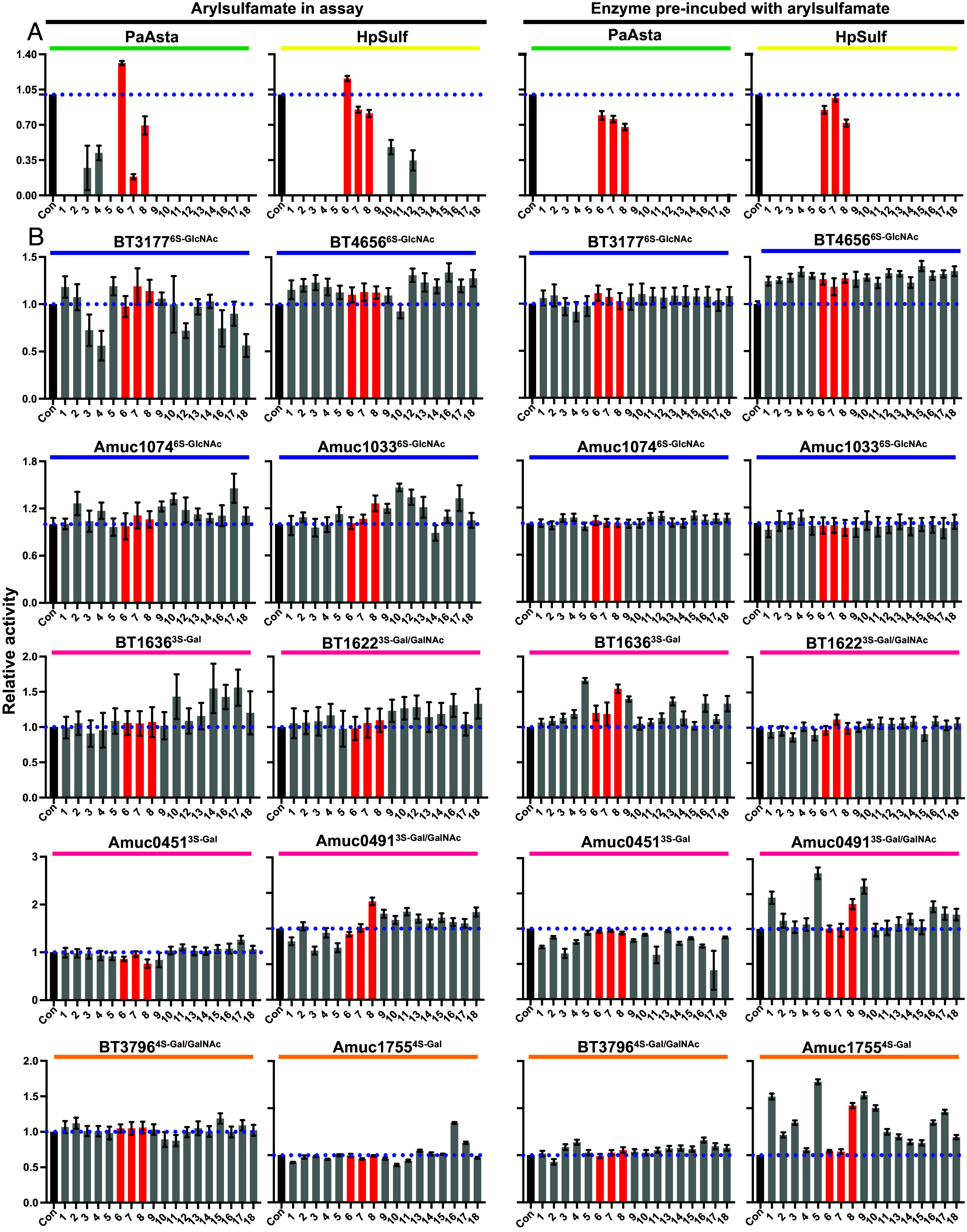
The effectiveness of arylsulfamate inhibitors against S1 steroid and carbohydrate sulfatases. (*A*) The steroid sulfatases *Pa*Asta and *Hp*Sulf, belonging to families S1_4 (green bar) and S1_2 (yellow bar), respectively, assayed against the panel of arylsulfamates (compounds **1**–**18** listed in Fig. 1). (*B*) Activities of family S1_11 sulfatases (BT3177^6S-GlcNAc^, BT4656^6S-GlcNAc^, Amuc_1033^6S-GlcNAc^, Amuc_1074^6S-GlcNAc^; blue bar above), S1_20 sulfatases (BT1622^3S-Gal/GalNAc^, BT1636^3S-Gal^, Amuc_0451^3S-Gal^, Amuc_0491^3S-Gal^; pink bar above), and the S1_16 sulfatases (BT3796^4S-Gal/GalNAc^ and BT1755^4S-Gal^; orange bar above) against the panel of arylsulfamates. For both *A* and *B* the *Right* two panels display activity with 1 mM arylsulfamate compound in the assay relative to untreated enzyme, while the *Left* two panels are overnight preincubation of the enzyme with selected 1 mM arylsulfamates then dilution of this preincubation mix into the reaction. Con indicates enzyme control without arylsulfamate. Kinetic curves from which the data are derived are shown in *SI Appendix*, Figs. S8–S31. The blue dotted line indicates 100% activity level and the red bars indicate control compounds.

With the observation that the arylsulfamates, including the negative control *N*-methylsulfamates, affected growth of *B. theta* on unsulfated and sulfated polysaccharides we next wanted to investigate the effect of our panel of arylsulfamates on purified S1 sulfatases. We first confirmed the potency of our panel of arylsulfamates against two S1 steroid sulfatases: *Pa*AstA from *Pseudomonas aeruginosa*, belonging to S1 subfamily 4 (S1_4), and commercially available snail sulfatase *Hp*Sulf from *Helix pomatia*, belonging to S1_2. The inhibitory activity of each compound was determined by measuring the sulfatase activity of each enzyme incubated in the presence of inhibitor, or following ~24 h preincubation and jump-dilution “washout” (26). At a concentration of 1 mM, all arylsulfamates were broadly equivalent in their ability to completely inhibit both enzymes; only minor differences were observed (Fig. 3 and *SI Appendix*, Figs. S8–S11 and Tables S3 and S4). Against *Pa*Asta, partial inhibition was observed for **3** and **4** before complete inactivation (Fig. 3 *A*, *Left* panel and *SI Appendix*, Fig. S8), while preincubation with the two compounds resulted in complete inhibition (Fig. 3 *A*, *Right* panel). Competitive inhibition of *Pa*Asta was observed with **7** in the assay experiments but not in the enzyme preincubation and jump dilution assays (Fig. 3 *A* and *B*). Similarly, for *Hp*Sulf, residual activity observed when treated with **10** and **12** was completely lost in the jump dilution experiment (Fig. 3*A* and *SI Appendix*, Fig. S11). These findings suggest different inactivation rates between the enzymes and are consistent with the irreversible, covalent, nature of the inactivation process. Importantly, near control levels of activity were observed upon treatment with the *N-*methyl and *N,N*-dimethyl sulfamates **6–8** (Fig. 3 *A*, *Right* panel).

Having confirmed the activities of the arylsulfamates against two S1 steroid sulfatases, we next examined their activity toward 10 bacterial S1 carbohydrate sulfatases from the HGM members *B. theta* and *Akkermansia muciniphila ATCC BAA-835 (A. muc*). These enzymes target common linkages found in host glycans: four from S1_11 (BT3177^6S-GlcNAc^, BT4656^6S-GlcNAc^, Amuc1033^6S-GlcNAc^, and Amuc1074^6S-GlcNAc^) target *O*6 sulfated *N*-acetyl-D-glucosamine (6S-GlcNAc), four from S1_20 (BT1636^3S-Gal^, BT1622^3S-Gal/GalNAc^, Amuc0451^3S-Gal^, and Amuc0491^3S-Gal/GalNAc^) target *O*3 sulfated D-galactose/*N*-acetyl-D-galactosamine (3S-Gal/GalNAc), and two from S1_16 (BT3796^4S-Gal/GalNAc^ and Amuc1755^4S-Gal^) target *O*4 sulfated D-galactose/*N*-acetyl-D-galactosamine (4S-Gal/GalNAc). No significant inhibition of any of the enzymes was observed when assaying their activity in the presence of 1 mM arylsulfamate, even after preincubation (Fig. 3*B* and *SI Appendix*, Figs. S12–S31 and Tables S3 and S4). Thus, while the panel of arylsulfamates inhibit bacterial and eukaryotic S1 steroid sulfatases, they do not inhibit S1 HGM carbohydrate sulfatases.

We also explored the ability of two potent growth inhibitory sulfamates, **2** and **17** (*SI Appendix*, Figs. S32 and S33), to inhibit S1 carbohydrate sulfatases in cell lysates. Cell lysates derived from *B. theta* grown to mid-exponential growth phase on chondroitin sulfate A (CSA) or Hep (to stimulate production of native S1 GAG carbohydrate sulfatases) were incubated with arylsulfamates **2** and **17** for 1 h, then sulfated mono- or disaccharide substrates were added. Compounds **2** and **17** did not impact the production of the final desulfated products (GlcNAc for Hep substrates and GalNAc for CSA substrates) as judged by TLC and HPAEC (*SI Appendix*, Figs. S32 *B* and *C*). Thus, compounds **2** and **17** are not effective inhibitors of natively produced S1 GAG carbohydrate sulfatases, and the effects observed on bacterial growth are likely to be due to effects on a nonsulfatase target.

### Carbohydrate Sulfamates and Vinylsulfonates do not Inhibit the S1_20 Sulfatase BT1636^3S-Gal^.

**Fig. 4.**
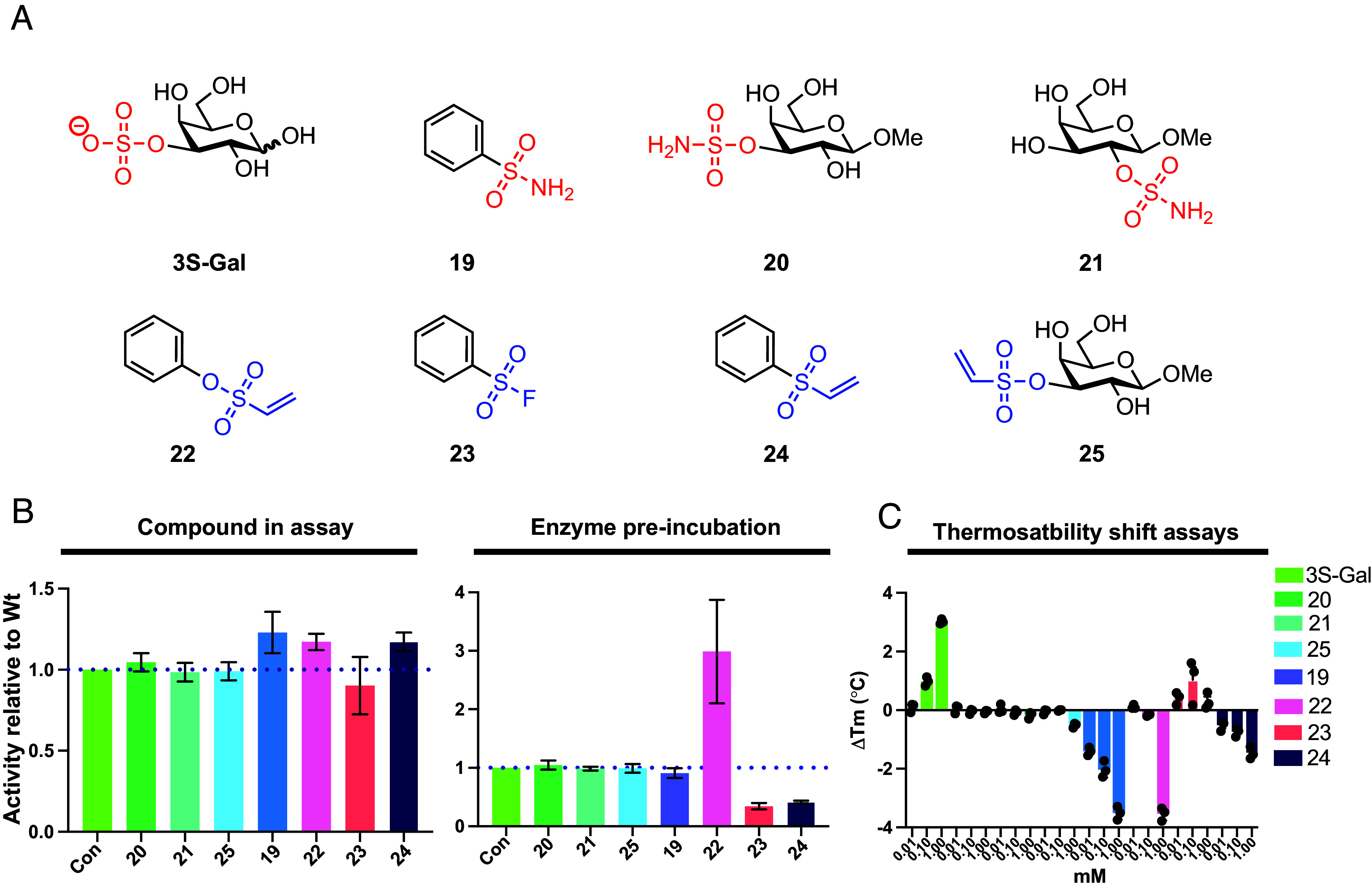
The carbohydrate sulfamate substrate mimics are ineffective against the S1 carbohydrate sulfatase BT1636^3S-Gal^. (*A*) Structure of the carbohydrate sulfonate/sulfamates and their aryl equivalents. (*B*) Plots show the activities of carbohydrate sulfamates, carbohydrate sulfovinyl, and arylsulfonates against BT1636^3S-Gal^. *Left* plot shows activity of enzyme treated with the compounds relative to untreated enzyme, while the *Right* plot shows the relative activity after the enzyme had been preincubated with the compounds overnight then diluted into the reaction mixture. (*C*) Thermostability shift assays (TSA) using inactive BT1636^3S-Gal^ against the substrate 3S-Gal, and the carbohydrate sulfamates, carbohydrate vinylsulfonate, and arylsulfonates. All compounds in *B* were used at a concentration of 1 mM.

The refractory nature of S1 carbohydrate sulfatases to arylsulfamate inhibition may arise from the inability to bind at the active site of these enzymes, which has evolved to bind sulfated glycans. Notably, S1 carbohydrate sulfatases exhibit low activity toward 4-nitrophenyl sulfate (4NP-SO_3_), which in contrast is rapidly hydrolyzed by the family S1 steroid sulfatase *Pa*Asta (*SI Appendix*, Fig. S34). We selected the well-studied S1_20 sulfatase BT1636^3S-Gal^, which desulfates 3*O* sulfated D-galactose (3S-Gal) and is essential for *B. theta* to utilize colonic mucin *O*-glycans, and to competitively colonize the colon of mice (11). We prepared two substrate analogues, D-galactose-3-*O*-sulfamate **20** and 3-*O*-vinylsulfonyl D-galactose **25**, the isomeric compound D-galactose-2-*O*-sulfamate **21**, aryl vinyl sulfone analogues **22** and **24**, aryl sulfonylfluoride **23**, and aryl sulfamate **19** (Fig. 4*a*). Of these compounds, the only activity toward BT1636^3S-Gal^ was for compounds **22**, **23,** and **24** but only after preincubation; **22** showed a ~3-fold increase in activity while **23** and **24** showed just over 50% inhibition (Fig. 4*B* and *SI Appendix*, Figs. S35 and S36 and Table S5). We assessed whether any of these compounds could bind by studying the inactive mutant Cys77Ser BT1636^3S-Gal^ using a thermal shift assay (TSA). No change in melting temperature was observed for compounds **20**, **21**, **23**, or **25** at concentrations up to 1.0 mM, while compounds **19, 22,** and **24** reduced the melting temperature (Tm), suggesting binding causes protein destabilization (Fig. 4*C* and *SI Appendix*, Fig. S37 and Table S6). In contrast, a substrate of BT1636^3S-Gal^, 3S-Gal, stabilized the protein (ΔTm = 3.0 °C) at a concentration of 1 mM (Fig. 4*C* and *SI Appendix*, Fig. S37 and Table S6). Collectively, these data demonstrate that the carbohydrate sulfamate **20**, and its vinylsulfonyl analogue **25**, are not inhibitors of BT1636^3S-Gal^ and do not bind the enzyme.

### Growth Inhibition By Arylsulfamates Extends to Multiple HGM Bacteroidota Species.

**Fig. 5.**
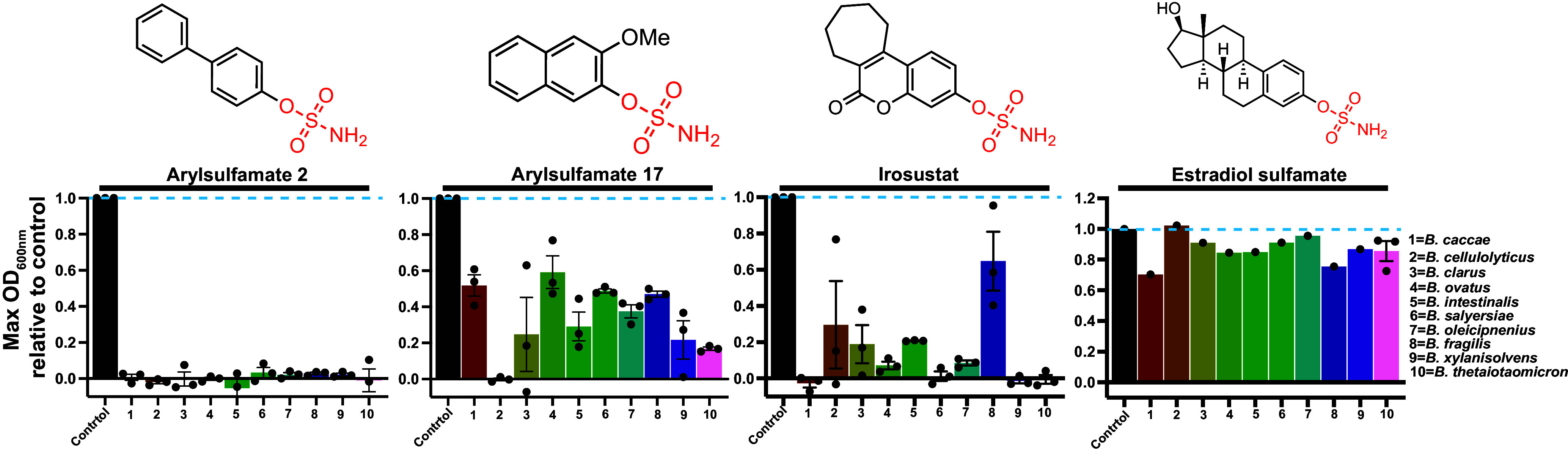
Arylsulfamates broadly affect growth of *Bacteroides* spp. of the HGM. *Bacteroides* species were grown in BHI media in the presence, or absence, of 1 mM of the listed arylsulfamate. The data plotted are the maximal OD_600nm_ observed for arylsulfamate treated growths relative to the untreated cells, which were each run in triplicate and randomly paired, then averages derived. For all the estradiol growth experiments limited material meant this shows a single experiment, except for *B. theta* which was run in triplicate.

We selected nine additional HGM *Bacteroides* species to see whether the growth effects observed for *B. theta* in the presence of arylsulfamate compounds **2** and **17** (Fig. 2) applied more broadly. We also included the arylsulfamate anticancer drugs Irosustat and estradiol sulfamate. No growth was observed for any species in the presence of 1 mM of arylsulfamate **2** (Fig. 5 and *SI Appendix*, Fig. S38). Arylsulfamate **17** exhibited growth defects across all species; in particular, *B. cellulosilyticus* for which no growth could be detected. Irosustat potently inhibited growth with all but one species, *B. fragilis*, showing limited or no growth (Fig. 5 and *SI Appendix*, Fig. S39). Only very mild growth defects were observed for estradiol sulfamate (Fig. 5 and *SI Appendix*, Fig. S39).

### TPP Identifies a Lipid Kinase Target for Arylsulfamates.

We next applied thermal proteome profiling (TPP) to determine the targets of arylsulfamate compounds **2** and **17**. TPP is a nontargeted mass spectrometry technique that can identify protein–compound interactions based on changes in a protein’s thermal stability. Increased, or decreased, thermal stability is quantified as an increase, or decrease, in the relative abundance of peptides originating from the soluble protein fraction over a temperature gradient following incubation with a ligand (27).

Cell lysates were derived from *B. theta* grown on chondroitin sulfate A. *B. theta* lysates were treated with inhibitors **2** or **17**, or DMSO control and then heated at 10 different temperatures between 45 to 72 °C, and the soluble proteome subjected to trypsinization, and analyzed by liquid chromatography-mass spectrometry. The *B. theta* lysate proteome showed a relatively narrow melting temperature ranging between 50 and 58 °C (90% range) (*SI Appendix*, Fig. S40*A*). A total of 2,550 proteins were identified in the combined analysis, from which 11 S1 sulfatases were identified, including the three upregulated in response to chondroitin sulfate A (BT1596, BT3333, and BT3349) (28) (*SI Appendix*, Fig. S40 *B* and *C*). The melting temperature (Tm) was established for 2,112 proteins (*SI Appendix*, Fig. S40*D*) based on the detection of at least two unique peptides in a minimum of two replicate analyses (*Methods*). Melting curves were determined for 8 out of the 11 identified S1 sulfatases (*SI Appendix*, Fig. S41), none of which was significantly affected by treatment with compounds **2** or **17**, indicating no direct interaction.

**Fig. 6.**
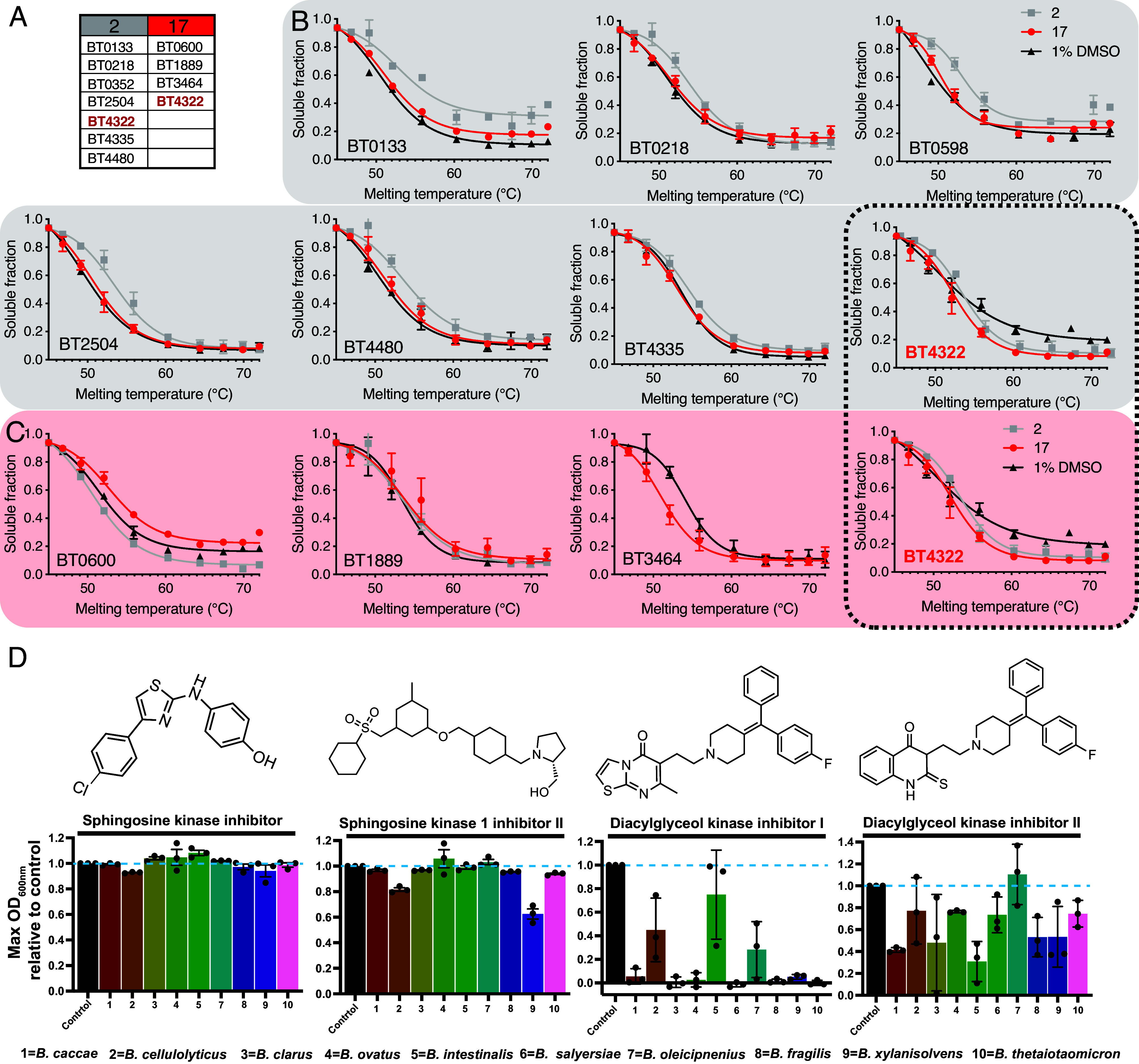
Global protein thermal stability reveals off target interactions of arylsulfamate inhibitors **2** and **17**. (*A*) Table of proteins that display an altered Tm in the presence of arylsulfamates **2** and **17**. (*B*) Proteins identified with a significant change (*Methods* and Dataset S1) in melting point upon treatment with inhibitor **2** (n = 2). (*C*) Proteins identified with a significant change in melting point upon treatment with inhibitor **17** (n = 2). The dashed box highlights BT4322 as the only protein common to both groups. (*D*) *Bacteroides* species were grown in BHI media in the presence, or absence, of 0.125 mM or 0.0625 mM diacylglycerol kinase inhibitor I and II, respectively. The data plotted are the maximal OD_600nm_ observed for inhibitor treated growths relative to the untreated controls, which were each run in triplicate and randomly paired, then averages derived.

TPP identified 7 proteins for which arylsulfamate **2** induced significant changes in Tm, and 4 proteins for arylsulfamate **17**, indicating a potential arylsulfamate–protein interaction (Fig. 6 *A*–*C*). Some of these hits can be discounted as causing the defective growth phenotype: Disruption of BT0600, BT2504, and BT3464 through transposon mutagenesis results in no phenotype when grown on supplemented BHI media (29). BT0133 is a homologue of the *Escherichia coli* protein MurQ (having 45% identity), a protein that produces GlcNAc-6-phosphate from 6*O* phosphorylated *N*-acetyl-D-muramic acid (MurNAc). Loss of MurQ in *E. coli* does not cause a no-growth phenotype (30) and no MurNAc was detected in our experiments. This leaves a total 7 candidate proteins (BT0218, BT1443, BT1889, BT4322, BT4335, BT4346, and BT4480), of which only one was identified in TPP experiments with both arylsulfamates **2** and **17**, the uncharacterized protein BT4322 (Fig. 6 *B* and *C* and *SI Appendix*, Fig. S40*E*).

BT4322 is homologous to the cytoplasmic diacylglycerol kinases (DGK) YerQ and DgkB, from the gram-positive bacteria *Bacillus subtilis* (31) and *Staphylococcus aureus* (32), respectively, and the human sphingosine kinase SphK1 (33). YerQ is essential for growth of *B. subtilis* and is involved in production of lipoteichoic acid (LTA), a cell wall glycophospholipid (31). BT4322 is predicted to have the same fold and catalytic apparatus as both DgkB (PDB:2QV7) and SphK1 (PDB:3VZB), and similar ATP binding site motifs and lipid binding pockets (*SI Appendix*, Fig. S42). BT4322 is therefore likely to be a *B. theta* lipid kinase, but the nature of the lipid substrate is yet to be determined. LTA is specific to gram-positive bacteria, and so BT4322 may act on another membrane lipid in *B. theta*, or another as yet unknown target. BLASTp using BT4322 against the nine other arylsulfamate-sensitive *Bacteroides* bacteria returned a single orthologue from each species, with 100% query coverage, and a minimum of 84% identity suggesting a conserved role for these proteins across these organisms (*SI Appendix*, Fig. S43 and Table S7).

### Diacylglycerol Kinase Inhibitors Significantly Inhibit the Growth of *Bacteroides* Species.

We next investigated whether we could recapitulate the arylsulfamate induced growth defects assigned to inhibition of the putative lipid kinase BT4322 using commercially available sphingosine and diacylglycerol kinase inhibitors. Diacylglycerol kinase inhibitor (DAGKI)-I was effective at inhibiting the growth of all species with only *B. cellulosilyticus*, *B. intestinalis*, and *B. oleicipnenius* showing limited, albeit highly variable, growth with extended lag phases (Fig. 6*D* and *SI Appendix*, Fig. S44). Bacteria treated with DAGKI-II exhibited a milder phenotype, but this compound still inhibited the growth of all species (except for *B. oleicipnenius*), lowering the final OD and extending the lag phase (Fig. 6*D* and *SI Appendix*, Fig. S44). No growth defect could was observed with sphingosine kinase inhibitor (SKI), while sphingosine kinase 1 inhibitor (SK1I)-II caused mild phenotypes in four species, manifesting as an elongated lag phase, except for *B. xylanisolvens* which showed severe defects (Fig. 6*D* and *SI Appendix*, Fig. S45). These data support the view that the observed arylsulfamate growth defects are mediated through inhibition of BT4322, which functions as a lipid kinase.

### Destabilizing Inhibitor Interactions with BT4322 Correlate with Severe Phenotypes.

**Fig. 7.**
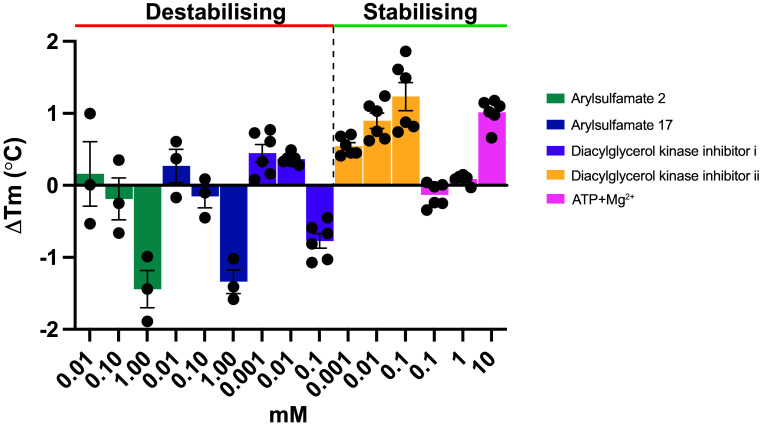
Recombinantly expressed BT4322 binds arylsulfamates, diacylglycerol kinase inhibitors, and ATP. TSA of BT4322 against arylsulfamate and diacylglycerol kinase inhibitors, and the kinase substrate ATP. A final protein concentration of 5 μM, in 100 mM Bis-Tris-Propane (BTP), pH 7.0, and 150 mM NaCl, supplemented with the appropriate ligand concentration and 5% DMSO. A minimum of three, and a maximum of six, independent assays were performed for each protein and protein ligand combination and SE of the mean derived.

BT4322 was expressed recombinantly in *E. coli* and its interaction with inhibitors and ATP assessed by TSA. Arylsulfamates **2** and **17** show a concentration-dependent destabilization up to 1 mM (Fig. 7) which is consistent with the TPP data (Fig. 6 *B* and *C*). The DAGK-I inhibitor also caused concentration-dependent destabilization, similar to arylsulfamates **2** and **17**, and correlates with a no growth phenotype for *B. theta* grown in the presence of DAGK-I (Fig. 6*D*). By contrast DAGK-II caused a concentration-dependent stabilization (Fig. 7) and correlated with only a mild growth defect (Fig. 6*D*). The substrate ATP also stabilized BT4322, supporting its activity as a lipid kinase.

### Ligand Docking of Arylsulfamates into Putative Lipid Kinase BT4322.

To understand how arylsulfamates may interact with BT4322 we performed blind ligand docking (no pocket specified) with arylsulfamates **2**, **17** and Irosustat using the AlphaFold 2 predicted model of BT4322. The docking results for Irosustat with BT4322 predicted binding almost exclusively to the putative lipid binding pocket, with some minor solutions elsewhere. Similar results were obtained for arylsulfamate **2** and **17**, but with some models also predicting binding in the ATP site (*SI Appendix*, Fig. S46). This binding of the arylsulfamates in the lipid pocket is consistent with what has been observed for other lipid kinase inhibitors (32, 34).

### In Vitro and In Vivo Effects of Arylsulfamates on Colonic Bacteria.

**Fig. 8.**
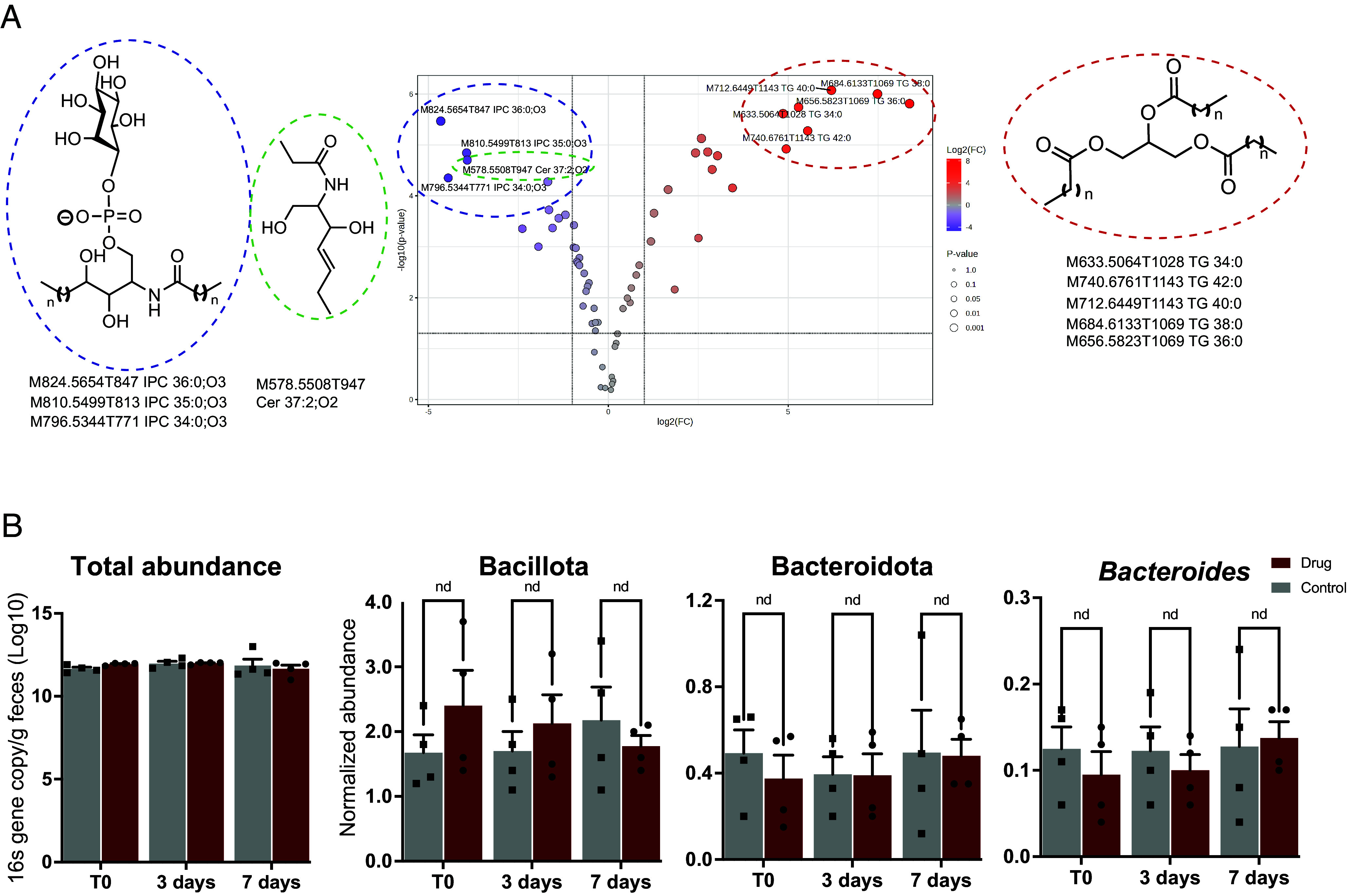
The effect of arylsulfamate on the growth of colonic bacteria in vitro and in vivo. (*A*) a volcano plot showing the alterations of membrane lipids when *B. theta* is grown in brain heart infusion media in the presence of 0.2 mM arylsulfamate 2. The structures of the up and down regulated lipids are shown to the *Right* and *Left* of the plot, respectively. (*B*) The changes in relative abundance of colonic bacteria in the mouse gut from animals that have been orally gavaged with the phase II arylsulfamate Irosustat; nd indicates no significant difference as determined from a two-tailed, unpaired, *t* test with a threshold of *P*-value of ≤0.05.

Growth of *B. theta* in the presence of sublethal doses of arylsulfamate **2**, and subsequent lipidomic analysis, showed a drastically altered membrane lipid composition while only causing a 15% reduction in total cell mass. These observations indicate arylsulfamate **2** affects lipid metabolism, supporting the TPP target identification of a lipid kinase as mediating the effects of arylsulfamates (Fig. 8*A*). The largest decrease (log_2_ FC ~ –5) was in the phosphorylated ceramides, suggesting ceramides as the potential lipid substrate for BT4322, while a large increase (log_2_ FC > 8) in membrane unphosphorylated triglycerides was also observed (Fig. 8*A*).

An in vivo animal study, where mice were gavaged with Irosustat, showed no significant alteration of Bacteroidota and Bacillota levels, or at the genus level for *Bacteroides* (Fig. 8*B*). However, these data lack the resolution to resolve at the species level where it is possible changes may have occurred. Furthermore, any changes in bacterial lipid composition in vivo were not assessed. Additionally, Irosustat may be absorbed in the small intestine and not reach the colon to exert an effect on colonic bacterial species.

## Discussion

Arylsulfamates are effective irreversible, active site directed, inhibitors of S1 steroid sulfatases (25) and display relatively mild side effects when administered to humans to treat hormone-dependent cancers in phase II clinical trials (24, 35). S1 carbohydrate sulfatases have been implicated in driving several disease states and it was presumed that arylsulfamates would be broadly effective across the S1 family. However, the work presented here shows that the panel of arylsulfamates tested have little to no effect on the ten HGM S1 carbohydrate sulfatases analyzed, despite sharing the invariant sulfate binding subsite (S subsite) and with a FGly nucleophile. Furthermore, a substrate mimicking carbohydrate sulfamate not only failed to inhibit the target S1 carbohydrate sulfatase, but showed no evidence of binding (Fig. 4 *B* and *C*). Collectively, these data demonstrate that aryl or carbohydrate sulfamates are not effective inhibitors of the HGM S1 carbohydrate sulfatases tested here. Similarly, the vinyl sulfone and sulfonylfluoride modifications are also ineffective inhibitors. With the S subsite being invariant, the apparent differing susceptibilities of these enzyme classes must be due to the distinctive chemical environments and/or structural geometries of glycan versus steroid binding subsites. Steroid binding sites include a hydrophobic patch that can accommodate lipophilic molecules and may allow the sulfamate moiety to adopt an alternative binding conformation not possible for carbohydrate sulfatases. In contrast, carbohydrates are hydrophilic, and S1 carbohydrate sulfatases have polar substrate binding regions, which form extensive hydrogen bonding networks (11, 20, 36, 37). The polar, hydrogen bond-driven nature of the glycan binding site may impose a level of rigidity on substrate interactions that may be incompatible with alternative binding modes that may be necessary to accommodate sulfamate groups. Moreover, the aryloxy group in arylsulfamates is a good leaving group (p*K*_a_ ≤ 10, depending on structure), whereas a carbohydrate alcohol is a much poorer leaving group (p*K*_a_ value approximately 16).

Carbohydrate sulfamate inhibitors have been developed against the *endo*-acting human S1 carbohydrate sulfatase Sulf1 by replacing the sulfate in HS di-, tri, and tetrasaccharides with a sulfamate (38, 39). The most potent compound was the trisaccharide sulfamate, with an IC_50_ value of 0.5 μM (versus 70 μM for the disaccharide), but this compound operated through a competitive mechanism (38, 39) distinct from the time-dependent inactivation seen with arylsulfamates, with potency largely driven by the number of sugar residues. In contrast, TSA gave no evidence of the monosaccharide sulfamate **20** binding to the *exo*-acting BT1636^3S-Gal^, suggesting that the sulfamate cannot interact with this sulfatase.

TPP analysis highlighted BT4322, a putative lipid kinase, as the most likely target through which the arylsulfamates mediate their growth inhibiting effects on *B. theta* and lipidomic analyses of *B. theta*, grown in the presence of arylsulfamate **2**, showed large changes in membrane lipid composition supporting BT4322 as the target. Studies using lipid kinase inhibitors further support BT4322 as being a lipid kinase. A single homologue of BT4322, with high sequence identity, is found in *B. theta* and the nine other species examined here, suggesting an important role for BT4322 in phospholipid biosynthesis and a lack of functional redundancy in *Bacteroides*. A homologue of BT4322 in *Bacillus subtilis* is implicated in peptidoglycan metabolism and cell membrane integrity, and the corresponding genetic knock out mutant displayed a no-growth phenotype (31). In vivo mouse data upon oral dosing with Irosustat did not identify any significant effects on the colonic microbiota at the phylum or *Bacteroides* genus level. This may be as a result of rapid uptake of the drug in the stomach or small intestine. It will be important to identify sulfamates that can transit to the distal gut and study mice inoculated with HGM bacteria, which will allow studies of species level changes and alteration of membrane lipid composition. Interestingly, it has recently been shown that altered *B. theta* membrane lipid composition can lead to the transfer of bacterial lipids to the host cell membrane (40).

This work reveals three findings with corresponding implications: 1) aryl and carbohydrate sulfamates and sulfonates are not effective inhibitors of *exo*-acting HGM S1 carbohydrate sulfatases. There are no known inhibitors of these enzymes, and this is an area where further research is needed. 2) Arylsulfamates have unexpected off-target effects with negative impacts on HGM *Bacteroides* species. Potentially, the removal of these mucin degrading bacteria could help treat IBD but the effects on the HGM may also lead to undesirable side-effects on the host. 3) Selected nonsulfated complex carbohydrates confer a resistant/protective phenotype on HGM *Bacteroides* species that can protect from the harmful effects of arylsulfamates. This could help to mitigate the effect of arylsulfamates (and potentially other drugs that transit to the colon), opening the potential for paired drug-glycan cotreatment strategies.

## Methods

### Recombinant Protein Production.

Catalytically-active recombinant S1 sulfatases [in which Ser, within the C/S-X/P/A-X-R consensus sequence, for *B*. *thetaiotaomicron* sulfatases was mutated to Cys, to permit conversion to the formylglycine (FGly) residue by *E. coli* (18)], or catalytically inactive sulfatases (in which Cys in the consensus sequence is mutated to Ser, thus preventing formation of FGly) were expressed in the *E. coli* strain TUNER (Novagen). Cultures were grown to mid-exponential phase in LB media supplemented with 50 μg/mL kanamycin at 37 °C, in an orbital shaker set to 180 rpm. Cultures were then cooled to 16 °C, and recombinant gene expression was induced by the addition of 0.1 mM isopropyl β-D-1-thiogalactopyranoside for 16 h at 16 °C, and 180 rpm. Cells were collected by centrifugation at 5,000×*g* and pellets were resuspended in 20 mM HEPES, pH 7.4, with 500 mM NaCl, then were sonicated on ice. Recombinant protein was then purified by immobilized metal ion affinity chromatography using a cobalt-based matrix (Talon, Clontech) and, after a wash with resuspension buffer, eluted with a step gradient of 10, 50, and then 100 mM imidazole in resuspension buffer. Proteins were then analyzed by SDS-PAGE and appropriately pure fractions were pooled and dialyzed into 10 mM HEPES pH 7.0 with 150 mM NaCl. Protein concentrations were determined by measuring absorbance at 280 nm using the molar extinction coefficient calculated by ProtParam on the ExPasy server (web.expasy.org/protparam/).

### Synthesis of Chemical Compounds.

Full detail of the synthetic procedures can be found in the supporting information.

### Spectrophotometric Based Sulfatase Assays.

Production of *para*-nitrophenolate from *para*-nitrophenol sulfate by steroid sulfatases was monitored at *A*_400nm_ using a HIDEX sense or spectra max plus 384 (molecular devices) plate reader in a 96-well plate format. Reaction mixtures (100 μL) were monitored at ambient temperature (20 to 25 °C) and contained 1 mM substrate with 100 mM Bis-Tris-Propane (BTP), pH 7.0 with 5% (v/v) DMSO, 150 mM NaCl, and 5 mM CaCl_2_.

### Microfluidic-Based Desulfation Assay.

Reducing end BODIPY (maximal excitation/emission coefficient of ∼503/511 nm) labeled sulfated substrates were detected using the EZ Reader II platform (Ret biochem) via LED-induced fluorescence, as described previously (41). Real-time kinetic evaluation of substrate desulfation was achieved using a nonradioactive microfluidic mobility shift carbohydrate sulfation assays were optimized in solution with a 12-sipper chip coated with CR8 reagent and performed using a PerkinElmer EZ Reader II system employing EDTA-based separation buffer. Pressure and voltage settings were adjusted manually (1.8 psi, upstream voltage: 2,250 V, downstream voltage: 500 V) to afford optimal separation in the reaction mixture of the sulfated substrate and unsulfated glycan product, with a sample (sip) time of 0.2 s, and total assay times appropriate for the experiment. Individual desulfation assays were carried out at 28 °C and were preassembled in a 384-well plate in a volume of 80 μl in the presence of substrate concentrations of 1 μ’M with 100 mM BTP, MES, or Tris, dependent on the pH optimum of the sulfatase being assayed, and 5% DMSO, 150 mM NaCl, 0.02% (v/v) Brij-35 and 5 mM CaCl_2_. The amount of desulfation was directly calculated in real-time using EZ Reader II software by measuring the sulfated carbohydrate: unsulfated carbohydrate ratio at each time-point during the assay. The activity of sulfatase enzymes was quantified in “kinetic mode” by monitoring the amount of unsulfated glycan generated over the assay time, relative to control assay with no enzyme; with desulfation of the substrate limited to ∼20% to prevent loss assay linearity via substrate depletion. *k*_cat_/*K*_M_ values, using the equation V_0_ = ( *k*_cat_/*K*_M_)/[E][S], were determined by linear regression analysis with GraphPad Prism software.

### Differential Scanning Fluorimetry.

Thermal shift/stability assays (TSAs) were performed using a StepOnePlus Real-Time PCR system (LifeTechnologies) and SYPRO-Orange dye, at a 1:1,000 dilution, (excitation 470 nm, emission maximum 570 nm, Invitrogen) with thermal ramping between 20 and 95 °C in 0.3 °C step intervals per data point to induce denaturation of purified, folded, inactive, wild-type (Ser77) BT1636^S1_20^ in the presence or absence of substrate or potential inhibitors. The melting temperature (Tm) corresponding to the midpoint for the protein unfolding transition was calculated by fitting the sigmoidal melt curve using the Boltzmann equation in GraphPad Prism, with R^2^ values of ≥0.99, as described in (41). Data points after the fluorescence intensity maximum were excluded from the fitting. Changes in the unfolding transition temperature compared with the control curve (ΔT_m_) were calculated for each ligand. A positive ΔT_m_ value indicates that the ligand stabilizes the protein from thermal denaturation, and confirms binding to the protein. All TSA experiments were conducted using a final protein concentration of 5 μM in 100 mM BTP, pH 7.0, and 150 mM NaCl, supplemented with the appropriate ligand concentration and 5% DMSO. Three independent assays were performed for each protein and protein ligand combination.

## Supplementary Material

Appendix 01 (PDF)

Dataset S01 (XLSX)

Dataset S02 (XLSX)

## Data Availability

Mass spectrometry data have been deposited in ProteomeXchange consortium via PRIDE (PXD065411) (42). Raw data files and data tables in .csv format including descriptions of samples and their associated files, and lipid annotations for all detected lipids and associated features areas across files, have been uploaded to MassIVE dataset MSV000098236 (43). All study data are included in the article and/or supporting information.
