# Supplementary material for: Arylsulfamates inhibit colonic Bacteroidota growth through a sulfatase-independent mechanism
Source: Proc Natl Acad Sci U S A. 2025 Jul 10;122(28):e2414331122. doi: 10.1073/pnas.2414331122 (PMC12280919; doi:10.1073/pnas.2414331122)
Supplement: Supplementary file 1 — Appendix 01 (PDF) [file pnas.2414331122.sapp.pdf]

Supporting Information for

Arylsulfamates inhibit colonic Bacteroidota growth through lipid kinases and not sulfatases

Conor J Crawford[1+], Charles W.E. Tomlinson[2,3,4+], Christian Gunawan[5], Zongjia Chen[5], Dominic P Byrne[6], Cosette Darby[7], Martina L. G. Conti[2], Tony Larson[2], Ana S Luis[8,9], Stefano Elli[10], Edwin A Yates[6], David N Bolam[7], Sjoerd van der Post[8], Spencer J Williams[5]*, and Alan Cartmell[2,3,4]*

[1]Max Planck Institute of Colloids and Interfaces, Am Mühlenberg 1, 14476, Potsdam, Germany
[2]Department of Biology, University of York, Wentworth Way, York, YO10 5DD, U.K. w
[3]York Structural Biology Laboratory (YSBL), University of York, Wentworth Way, York, YO10 5DD, U.K.
[4]York Biomedical Research Institute (YBRI), University of York, Wentworth Way, York, YO10 5DD, U.K.
[5]School of Chemistry and Bio21, Molecular Science and Biotechnology Institute, University of Melbourne, Parkville, Victoria3010, Australia
[6]Department of Biochemistry, Cell and Systems Biology, Institute of Systems, Molecular and Integrative biology, University of Liverpool, Liverpool L69 7ZB, U.K.
[7]Biosciences Institute, Faculty of Medical Sciences, Newcastle University, Medical School, Framlington place, Newcastle upon Tyne, NE2 4HH, U.K.
[8]Department of Medical Biochemistry and Cell Biology, University of Gothenburg, Gothenburg, Sweden
[9]SciLifeLab, University of Gothenburg, 41390 Gothenburg, Sweden
[10]Istituto di Ricerche Chimiche e Biochimiche G. Ronzoni, Milano,20133, Italy

[+]Indicates these authors contributed equally

*To whom correspondence should be addressed:
alan.cartmell@york.ac.uk
sjwill@unimelb.edu.au

This PDF file includes:

Supplemental methods
Pages 3-6

General procedure and analysis for chemical syntheses of compounds not previously reported
Pages 7-19

Figures S1 to S46
Pages37-84

Tables S1 to S7
Pages 85-93

Legends for Datasets S1 and S2, and SI References
Page 94

Methods

Bacterial growth experiments

Growth of various *Bacteroides* species was conducted in brain heart infusion media. BHI [Sigma 53286-500G], 37g per litre) was dissolved in 18.2Ω deionised water, and then sterilised by autoclaving. The media was then allowed to cool then haematin (1.2 mg/ml in 0.2 His-HCl pH 8.0) was added in a 1:1000 dilution. Five ml of the resulting BHI media was then inoculated with *Bacteroides* species and grown overnight, and diluted 1:20 into 96 well plate growths (10 μl in 200) to monitor growth. Growth of the *B. thetaiotaomicron* VPI-5482 on specified glycan sources was achieved by mixing 1:1 the 0.22 micron filter-sterilised polysaccharides (10.2 mg/ml in H_2O) with 0.22 micron filter-sterilised 2 x minimal media (per 50 ml was: 0.1g of ammonium sulfate and sodium carbonate, 0.05g cysteine, 10 ml 1 M potassium phosphate pH 7.2, 0.1 ml 1 $mg.ml^{-1}$ vitamin K, 1 ml of 0.4 $mg.ml^{-1}$ iron sulfate, 0.4 ml of 0.25 mg.ml resazurin, 0.05 ml of 0.01 $mg.ml^{-1}$ vitamin B12, 5 ml of mineral salts for defined media, and 100 μl of 1.2 mg/ml haematin in 0.2 M His-HCl pH8.0); this gives a final glycan concentration of 5 mg/ml in a 1 x minimal media. All growth experiments were monitored continuously in 96-well plates using a cerillo stratus plate reader within a Don whitely VA-500, A85, or A35 cabinet configured for anaerobic conditions (80% N_2, 10% CO_2, 10% H_2). Where appropriate, either 1% DMSO (control growths) or 1% DMSO and 1 mM arylsulfamates was added to growth conditions. For sphingosine kinase inhibitor (SKI, 567731) and sphingosine kinase 1 inhibitor II (SK1-II, 567741) a maximum of 0.1 and 0.5 mM, respectively, could be dissolved in BHI with 1% DMSO. For diacylglycerol kinase inhibitors i (D5794-5MG) and ii (D5919-5MG) a maximum concentration of 125 μM and 62.5 μM, respectively, could be dissolved in BHI with 1% DMSO but were hazy; this did not affect measurement readings at OD_{600nm}. Overnight growth cultures were diluted 1:20 into 96 well plate growths (10 μl in 200 μl). Growth curves presented are averages of three technical replicates.

Thin layer chromatography (TLC)

B. thetaiotaomicron VPI-5482 was grown in 10 ml minimal media with 10 mg/ml (w/v) appropriate GAG (either CSA or Heparin) as the sole carbon source to mid-exponential phase in glass test tubes. Cells were harvested by centrifugation at 5,000 × g for 10 min at room temperature and washed 2 x with 5 ml PBS (pH 7.2) before being resuspended in 1 mL PBS. Cells washed in PBS were lysed and the lysates assayed against 5 mM of the appropriate substrate at 37 °C for up to 24 h. Assays were analysed by TLC, and 2 μL each sample was spotted onto silica plates and resolved in butanol:acetic acid:water (2:1:1) mobile phase. The plates were dried, and the sugars were visualized using diphenylamine stain (DPA: 1 ml of 37.5% HCl, 2 ml of aniline, 10 ml of 85% H_3PO_3, 100 ml of ethyl acetate, and 2 g of diphenylamine) by heating using a heat gun set to 450 °C on medium flow. Arylsulfates and their pNP products were visualised using 1 M NaOH and then heated as described for DPA stains.

High performance anion exchange chromatography (HPAEC)

Sonicated bacterial cell lysates grown on the glycosaminoglycans CSA and heparin were treated with 1 mM of arylsulfamates 3 and 12 for 1 hour then challenged with CSA or heparin mono- and disaccharides by mixing 1:1, to a final concentration of 5 mM, to see if sulfatase activity was still present. Analysis of the sugar

products was performed by HPAEC using an ICS-6000 with pulsed amperometic detection using a gold work electrode and pDH reference electrode with a standard quad carbohydrate waveform. Separation was done using a Dionex PA-200 analytical column preceded by a PA-200 guard column. Samples were resolved isocratically in 100 mM NaOH for 20 mins, then the column cleaned with 500 mM NaOH for 10 mins, and finally ran back into 100 mM NaOH for 5 mins, with a flow rate of 0.25 ml/min.

Thermal proteome profiling

A 300 ml culture of minimal media (see 'Bacterial growth experiments' section) supplemented with 5 mg/ml chondroitin sulfate A (CSA), stored in the anaerobic chamber overnight, and inoculated with 10 ml of *B. thetaiotaomicron* VPI-5482 was grown overnight in BHI media. This culture was grown to a target OD_{600nm} of ~0.5-0.6 at which point cells were centrifuged at 5000 x g and washed with PBS, this process was repeated two more times. Cells were then treated as in *Mateus et al*[1]; briefly, cells were then resuspended in lysis buffer (50μg/ml lysozyme, 1×protease inhibitor(Roche), 250 U/ml benzonase, and 1 mM $MgCl_2$ in PBS) to give an OD_{600nm} of 50. Samples were then sonicated on ice for 15 seconds three times. The lysate was then split into three: one sample was a control (1% (v/v) DMSO) and experimental samples contained 1 mM arylsulfamate **3** or 1 mM arylsulfamate **12** (1% (v/v) DMSO). Next 20 μl of each condition was aliquoted into a 96 well PCR plate across 10 wells in quadruplicate. The plates were then subjected to a temperature gradient from 45 – 72 °C (45, 46.8, 49.1, 52.1, 55.9, 60.3, 64.4, 67.4, 69.9, 72°C) for 3 min, followed by 3 min at room temperature. NP-40 (nonyl phenoxypolyethoxylethanol) was then added to samples to a final concentration of 0.8% (v/v). Samples were transferred to a 0.22 μm 96 well filter plate (Millipore: MSGVS2210) and centrifuged at 500 x g for 5 min to remove protein aggregates. Following filtration, the flowthrough was combined 1:1 with 400 mM Tris pH 8.0 and 4% SDS (w/v). The protein concentration for each sample was determined by Bradford assay, and a volume corresponding to 10 µg for the lowest-temperature condition was used as input for all other samples in a set for analysis. Protein digestion was performed using a modified solid-phase-enhanced sample preparation (SP3) based method adapted to a 96 well format in 0.45µM filter plates (Millipore, MSRPN04)[2,3]. Premixed magnetic SpeedBeads (Cytiva, (1:1, GE45152105050250 and GE 65152105050250)) were added to the samples, combined with two volumes of ethanol and incubated for 10 min while mixing at 150 RPM. Samples were transferred to the filter plates and washed four times with 200 µL of 70% ethanol (v/v) with centrifugation for 2 min at 2000 x g between each wash. Beads were resuspended in 40 µL 0.1 mM HEPES pH 8.5 containing trypsin/Lys-c (Thermo Scientific), 1.25 mM TCEP and 5 mM chloroacetamide (Sigma-Aldrich) and incubated at room temperature overnight while mixing 500 rpm. Peptides were collected by centrifugation, followed by a second elution with 2% DMSO. TMT 10-plex labelling (Thermo Scientific) was performed at a 2:1 ratio for 1 hr while mixing at 500 RPM. The labelling reaction was quenched with 0.4% hydroxylamine (v/v) for 15 min before each sample was combined and dried under vacuum. Peptides were resolved in 0.1% trifluoroacetic acid (TFA) and fractionated using 0.1% triethylamine with increasing concentrations of acetonitrile by high pH reverse phase into 8 fractions using C18 desalting columns (Pierce, 89852) pooled into 4 fractions and dried under a vacuum.

Lyophilized samples were redissolved in 0.1% TFA and analysed by LC-MS/MS using a nano HPLC system (EASY-nLC 1200, Thermo Scientific) coupled to a Q-Exactive HF-X mass spectrometer (Thermo Scientific). Peptides were separated using in-house packed columns (150 x 0.075 mm) packed with Reprosil-Pur C18-AQ 3 µm particles (Dr. Maisch). Elution was performed with a 5 to 45% gradient (A: 0.1% formic acid, B: 0.1% formic acid ,80% acetonitrile) in 135 minutes at 250 nl. Full mass spectra were acquired over a mass range of minimum 400 m/z and maximum 1600 m/z, with a resolution of 60,000 at 200 m/z. The isolation window for fragmentation spectra collection was set to 1Da and a fixed first mass of 100 m/z with a resolution of 30,000 at 200 m/z. The top 15 most intense peaks with a charge state ≥2 – 5 were selected for fragmentation by HCD with a normalized collision energy of 32% and subsequent excluded for selection for 30 seconds. Thermo .RAW files were converted into MZml using msConvert (proteowizard) and FragPipe (v20.0) configured with Msfragger (v3.8) and Philosopher (v5.0)[4,5] was used for protein identification and TMT quantification. Database searches were performed against the *Bacteroides thetaiotaomicron* reference proteome (4782 entries, UP000001414, 2023_10) with the following settings, parent ion mass and fragment ion mass accuracy after recalibration was set to 20 ppm. Enzyme specificity was set to trypsin, fixed modifications were set for cysteine carbamidomethylation and TMT modified lysine, variable modifications considered were methionine oxidation, protein n-terminal acetylation and TMT modification on peptide n-terminal or serine. The identified proteins and peptides were both filtered at a 1% and only identifications based on ≥2 unique peptides with a minimum ion purity of 0.75 were considered for quantification and thermal proteome profiling. Further data analysis was performed in R environment to combine the search outcome, normalize reporter ion channels. Meltome curve fitting analysis and statistical analysis were executed using the TPP-TR workflow in the TPP package[6].Briefly, protein candidates with altered melting temperature were considered based on the following criteria. P-values for the two replicate experiments are <0.05 and <0.1, the melting point shifts in the vehicle versus treatment experiments have the same direction. Both melting point differences in the two pairs of control versus treatment experiments are greater than the melting point difference between the two vehicle controls and the minimum slope in each of the control versus treatment experiments was <0.06. In addition, proteins were only considered for melting curve analysis if the identification was based on a minimum of two unique peptide identification; see Dataset 1.

Ligand docking experiments

All docking experiments were conducted using SwissDock[7] on the Expasy server using default parameter; no binding site was specified. The Alphafold model of BT4322 (Q89ZQ4) and the crystal structure of SphK1 (3VZB) were used, and where appropriate heteroatoms removed, and uploaded to the SwissDock server as PDB files. Small molecules to be docked were built in Jligand[8], the generated PDB or CIF files converted to MOL2 files using the openbabel server, and uploaded to SwissDock as MOL2 files.

Lipidomic analysis

All solvents used were HPLC or LC-MS grade. To the cell pellet, 997 µL of 2:1 methanol : chloroform (MeOH:$CHCl_3$) and 208 µL of 0.005 N HCl were added. The tubes were vortexed briefly and incubated on ice for 5 minutes. Following centrifugation at 18,000 xg for 5 minutes at 4°C the supernatant was transferred to a fresh tube. $CHCl_3$ (332 µL)) and H_2O (332 µL) were added and briefly vortexed to form two phases.

After centrifugation at 3600 xg for 1 minute at 4°C, the lower organic phase was transferred to a LC-MS tapered vial and dried under a gentle stream of nitrogen. The samples were resuspended in 80 µL of 7:3 acetonitrile : isopropanol (ACN:IPA) for LC-MS analysis.

Untargeted lipid analysis was performed on bacterial pellets and data processed using R scripts as previously described[9], except that data was collected on a Thermo Orbitrap Exploris 480 mass spectrometer with MS1 and HCD ddMS2 scans collected at 120,000 and 15,000 resolution (FWHM), respectively. The following parameters were used to acquire MS data: spray voltage 3500 V (positive mode), 2500 V (negative mode), sheath gas 50, auxiliary gas 10, sweep gas 1, ion transfer tube temperature 280°C, vaporizer temperature 200°C, RF lens 30, scan range m/z 270-2000. For MS2 parameters, mass selection range m/z 400-2000, ramped HCD collision energies 30, 50, 70%. MS1 m/z values were searched against candidate ions from positive and negative ionization mode adduct databases generated from the LipidMAPS structural database (https://www.lipidmaps.org/) and the list of Bacteroides lipids described in Barone *et al* 2024[10]. Candidate lipids were additionally annotated by searching MS2 spectra using LipidMatch[11], LipidBlast[12] and SIRIUS (version 6.1.0)[13].

Annotations were manually curated using either Barone's names, or the sum composition notation described in LipidMAPS. To distinguish between multiple isomers, annotations were prepended with a unique label indicating MS1 m/z values ("M") and retention time in s ("T"). The feature list was filtered to remove background contaminants (cutoff = 3 standard deviations above average background feature area), and any feature where relative standard deviation exceeded 30%. Features that could not be annotated as lipids were removed and where there were redundant measurements across positive and negative ionization modes or adduct type, only the most abundant representative was kept. This resulted in a final feature list of 63 lipids, covering ceramides (CE), menaquinones, triglycerides (TG) , phosphatidylethanolamines (PE), and various glycolipids. Feature area data was further processed through MetaboAnalyst 6.0 (https://www.metaboanalyst.ca) using the Statistical Analysis (one factor) workflow. Data was sample normalized by median before calculating fold-changes and log10 transformed before performing t-tests to calculate p-values.

Mice experiments

All experimental procedures involving animals were approved by the Swedish Laboratory Animal Ethical Committee at the University of Gothenburg. 8-week-old C57BL/6N mice were maintained under standardized specific pathogen free conditions with ad libitum access to food and water. Mice were treated at day 0, 1 and 2 by gavage with 3.2 mM of Irosustat dissolved in PEG 400:water:DMSO (6:3:1). The control group was gavaged with the vehicle only. Fecal samples for all animals were collected at day 0, 3 and 7. The weight of the mice was recorded at the days the mice were gavaged or during fecal collection. Bacterial genomic DNA was extracted with QIAamp PowerFecalPro DNA kit (Qiagen®) and quantification by qPCR as described previously[14]. The following primers were used for relative quantification of phylum Bacteroidetes (Fw 5'-CRAACAGGATTAGATACCT; Rv 5'-GGTAAGGTTCCTCGCGTAT) , phylum Firmicutes (Fw 5'-TGAAACTYAAAGGAATTGAGG; Rv 5'-ACCATGCACCACCTGTC) and genus Bacteroides (Fw 5'-GAAGGTCCCCCACATTG; Rv 5'-CGCKACTTGGCTGGTTCAG). The relative quantification normalized of specific taxons was determined for each sample using the 16S rRNA gene as reference.

General procedures

All chemicals used were reagent grade and used as supplied unless otherwise noted. Compounds **19** and **22-24** were purchased from commercial suppliers. Analytical thin-layer chromatography (TLC) was performed on Merck silica gel 60 F254 plates (0.25 mm). Compounds were visualized by UV irradiation or dipping the plate in a 5% H_2SO_4 ethanol solution. Flash column chromatography was carried out on automated Grace flash chromatography system. Analysis and purification by normal and reverse phase HPLC were performed by using an Agilent 1200 series. Products were lyophilized using a Christ Alpha 2-4 LD plus freeze dryer. ^{1}H, ^{13}C and HSQC NMR spectra were recorded on a Varian 400MR (400 MHz), Varian 600MR (600 MHz), or Bruker Biospin AVANCE700 (700 MHz) spectrometer. Signals are reported in terms of chemical shift [δ in parts per million (ppm)] relative to tetramethylsilane (TMS) or in D_2O using the solvent as the internal standard in ^{1}H NMR (D2O: 4.79 ppm ^{1}H). NMR data is presented as follows: Chemical shift, multiplicity (s = singlet, d = doublet, t = triplet, dd = doublet of doublet, m = multiplet and/or multiple resonances), coupling constant in Hertz (Hz), integration. All NMR signals were assigned on the basis of ^{1}H NMR, ^{13}C NMR, COSY, and HSQC experiments. High resolution mass spectra were obtained using a 6210 ESI-TOF mass spectrometer (Agilent) and a MALDI-TOF autoflexTM (Bruker). MALDI and ESI mass spectra were run on IonSpec Ultima instruments.

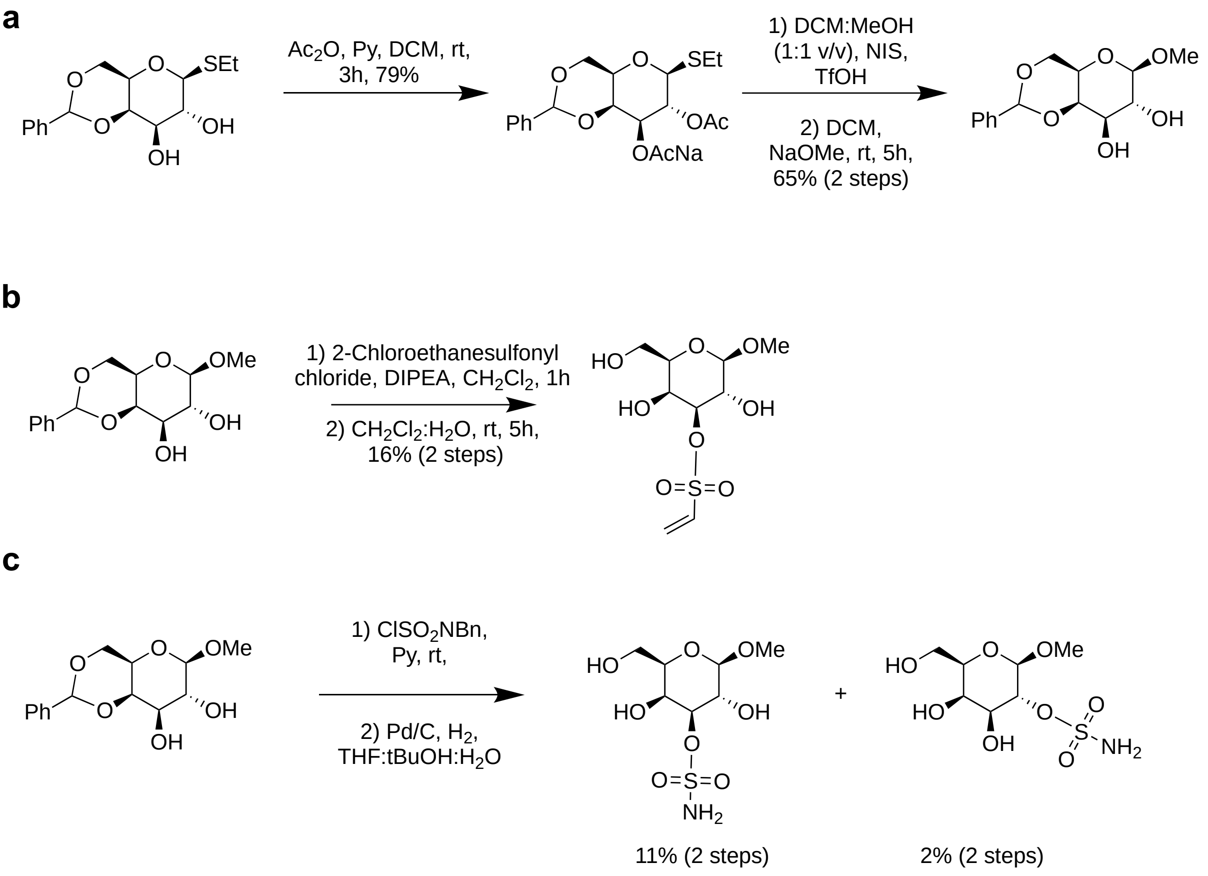

SI Scheme 1. Synthesis of galactosides equipped with sulfonate and vinyl sulfones groups. **a** synthesis of common ethyl 4,6-O-benzylidene-1-thio-β-D-galactoside intermediate**.** **b** synthesis of methyl 3-*O*-ethene-1-sulfonate-1-β-D-galactoside. **c** synthesis of methyl sulfonate galactosides.

Synthesis of aryl sulfamates

The following aryl sulfamates were prepared as described previously: 3-chlorophenyl sulfamate **1**,[15] *N*,*N*-dimethyl-4-nitrophenyl sulfamate **2**,[16] 4-phenylphenyl sulfamate **3**,[17] 4-nitrophenyl sulfamate **4**,[15] *N*,*N*-dimethyl-coumate **5**,[18] coumate **7**,[19] 4-methoxyphenyl sulfamate **17**,[15] 4-chlorophenyl sulfamate **18**,[15]

General procedure for the synthesis of aryl sulfamates

Chlorosulfonyl isocyanate (CSI, 1 equiv.) was cautiously added to a stirred, heated solution of the parent phenol (1 equiv.) in toluene (5-10 ml) at reflux. The solution was stirred at reflux overnight, then cooled to 0 °C. Water was added in a dropwise manner to the stirred, cooled toluene solution until evolution of gas ceased, resulting in the formation of a precipitate. Unless otherwise described, the precipitate was collected by filtration, washed (toluene), dried, and recrystallised (toluene) to afford the desired aryl sulfamate.

General procedure for the synthesis of naphthyl sulfamates

A mixture of formic acid (1.5 - 5 equiv.) and dimethylacetamide (DMA, 0.1 equiv.) was added to a stirred solution of CSI (1.5 - 5 equiv.) in CH_2Cl_2 at 40 °C. The mixture was refluxed for 15 min and cooled to r.t. then a solution of naphthol (1 equiv.) in DMA was added and stirred at r.t. for 17h. Water and EtOAc was added, the organic phase was collected and concentrated. Recrystallization or column chromatography afford the desired naphthyl sulfamate.

HPLC purification

Method 1, Prep RP-HPLC

Hypercarb column, 150 x 10 mm, 5 µm, flow rate of 3.5 mL/min with H2O (0.1% formic acid) as eluents [isocratic 100 % H_2O (0.1% formic acid) (5 min), linear gradient to 100% ACN (30 min)].

Method 2, analytical RP-HPLC

Hypercarb column, 150 x 4.6 mm, 3 µm, flow rate of 0.7 mL/min with H2O (0.1% formic acid) as eluents [isocratic 100 % H_2O (0.1% formic acid) (5 min), linear gradient to 100% ACN (30 min)].

Compound Characterisation

N-Methyl coumate (8)

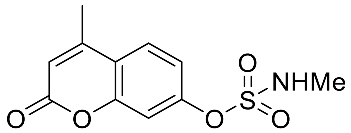

Freshly prepared *N*-methylsulfamoyl chloride (1.51 g, 11.65 mmol) was added to a stirred solution of 4-methyl-7-hydroxycoumarin (1.00 g, 4.66 mmol) in *N*,*N*-dimethylacetamide (10 mL) under an atmosphere of nitrogen. The solution was stirred under nitrogen (6 h). The reaction was quenched with water (100 mL), and the product extracted with ethyl acetate (3 × 30 mL), washed with water (3 × 30 mL) and dried ($MgSO_4$). The solvent was removed under reduced pressure, and the crude material subjected to flash chromatography (20% EtOAc: 80% pet. spirit). The product was repeatedly recrystallised (EtOAc: pet. spirit) due to the continual reappearance of an impurity to afford the *N*-methylsulfamate as cubic colourless crystals (794 mg; 59% yield). **m.p.** 171 °C. **^{1}H NMR** (500 MHz, d_6-DMSO) δ 2.44 (s, 3H), 2.75 (d, *J* = 4.7 Hz, 3H), 6.41 (s, 1H), 7.31 (dd, *J* = 8.7, 2.2 Hz, 1H), 7.35 (d, *J* = 2.2 Hz, 1H), 7.86 (d, *J* = 8.7 Hz, 1H), 8.45 (q, *J* = 4.4 Hz, 1H). **^{13}C NMR** (126 MHz, d_6-DMSO) δ 18.2, 29.2, 109.8, 114.1, 118.1, 118.2, 126.9, 152.0, 152.8, 153.6, 159.5.

4-Methoxynaphthalen-1-yl sulfamate (10)

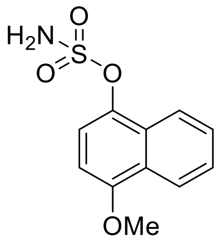

4-Methoxy-1-naphthol (390 mg, 2.24 mmol) and CSI (1.59 g, 11.2 mmol), column chromatography from 40% EtOAc/petrol (with 2% Et_3N) yielded the sulfamate (162 mg, 29%). **m.p.** 135 °C. **^{1}H NMR** (500 MHz, d_6-DMSO) δ 3.99 (s, 3H), 6.99 (d, *J* = 8.5 Hz, 1H), 7.43 (d, *J* = 8.4 Hz, 1H), 7.57 (ddd, *J* = 8.2, 6.9, 1.3 Hz, 1H), 7.63 (ddd, *J* = 8.3, 6.9, 1.3 Hz, 1H), 8.06 (s, 2H), 8.09 (d, *J* = 8.0 Hz, 1H), 8.18 (d, *J* = 8.2 Hz, 1H). **^{13}C NMR** (126 MHz, d_6-DMSO) δ 55.9, 103.6, 118.6, 121.6, 122.1, 125.4, 126.1, 127.1, 127.8, 139.4, 153.1.

7-Methoxynaphthalen-2-yl sulfamate (13)

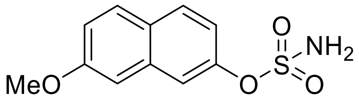

7-Methoxy-2-naphthol (390 mg, 2.24 mmol) and CSI (1.59 g, 11.2 mmol), column chromatography from 40% EtOAc/petrol (with 2% Et_3N) yielded the sulfamate (509 mg, 90%). **m.p.** 149 °C. **^{1}H NMR** (500 MHz, d_6-DMSO) δ 3.88 (s, 3H), 7.18 (dd, *J* = 8.9, 2.4 Hz, 1H), 7.26 (dd, *J* = 8.8, 2.2 Hz, 1H), 7.37 (d, *J* = 2.2 Hz, 1H), 7.71 (d, *J* = 2.0 Hz, 1H), 7.87 (d, *J* = 9.0 Hz, 1H), 7.92 (d, *J* = 8.8 Hz, 1H), 8.04 (s, 2H). **^{13}C NMR** (126 MHz, d_6-DMSO) δ 55.3, 106.0, 118.2, 118.8, 119.0, 126.8, 129.2, 129.4, 134.8, 148.4, 158.0.

6-Methoxynaphthalen-2-yl sulfamate (14)

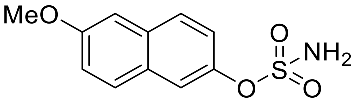

6-Methoxy-2-naphthol (300 mg, 1.72 mmol) and CSI (1.21 g, 8.61 mmol), column chromatography from 40% EtOAc/petrol (with 2% Et_3N) yielded the sulfamate (250 mg, 57%). **m.p.** 153–154 °C. **^{1}H NMR** (500 MHz, d_6-DMSO) δ 3.88 (s, 3H), 7.22 (dd, *J* = 8.9, 2.4 Hz, 1H), 7.44–7.35 (m, 2H), 7.74 (d, *J* = 1.9 Hz, 1H), 7.88 (dd, *J* = 11.7, 9.1 Hz, 2H), 7.99 (s, 2H). **^{13}C NMR** (126 MHz, d_6-DMSO) δ 55.3, 106.0, 119.2, 119.5, 122.0, 128.3, 129.2, 132.7, 146.1, 157.4.

4-Bromonaphthalen-1-yl sulfamate (11)

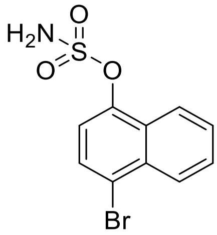

4-Bromo-1-naphthol (250 mg, 1.12 mmol) and CSI (793 mg, 5.6 mmol), column chromatography from 40% EtOAc/petrol (with 2% Et_3N) yielded the sulfamate (78 mg, 23%). **m.p.** 157 °C. **^{1}H NMR** (500 MHz, d_6-DMSO) δ 7.47 (d, *J* = 8.2 Hz, 1H), 7.76 (dt, *J* = 15.1, 7.1 Hz, 2H), 7.97 (d, *J* = 8.2 Hz, 1H), 8.18 (d, *J* = 8.3 Hz, 1H), 8.22 (d, *J* = 8.3 Hz, 1H), 8.28 (s, 2H). **^{13}C NMR** (126 MHz, d_6-DMSO) δ 119.1, 119.3, 122.9, 126.6, 127.8, 128.3, 128.7, 129.7, 132.1, 146.0.

3-Methoxynaphthalen-2-yl sulfamate (17)

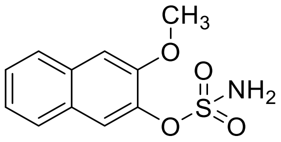

3-Methoxy-2-naphthol (390 mg, 2.24 mmol) and CSI (1.59 g, 11.2 mmol), column chromatography from 40% EtOAc/petrol (with 2% Et_3N) yielded the sulfamate (295 mg, 52%). **m.p.** 127–129 °C. **^{1}H NMR** (500 MHz, d_6-DMSO) δ 3.92 (s, 3H), 7.40 (t, *J* = 7.6 Hz, 1H), 7.51–7.47 (m, 2H), 7.87–7.81 (m, 3H), 8.03 (s, 2H). **^{13}C NMR** (126 MHz, d_6-DMSO) δ 55.9, 108.0, 120.4, 124.3, 126.4, 127.4, 127.6, 132.3, 139.4, 150.6.

6-Bromonaphthalen-2-yl sulfamate (15)

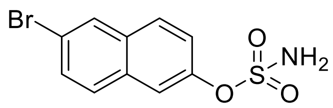

6-Bromo-2-naphthol (500 mg, 2.24 mmol) and CSI (475 mg, 3.36 mmol), recrystallization from $CHCl_3$/Petrol yielded the sulfamate (524 mg, 77%). **m.p.** 186 °C. **^{1}H NMR** (500 MHz, d_6-DMSO) δ 7.49 (dd, J = 8.9, 2.3 Hz, 1H), 7.70 (dd, J = 8.8, 1.9 Hz, 1H), 7.86 (d, J = 2.1 Hz, 1H), 7.96 (d, J = 8.8 Hz, 1H), 8.02 (d, J = 9.0 Hz, 1H), 8.10 (s, 2H), 8.28 (d, J = 1.4 Hz, 1H). **^{13}C NMR** (126 MHz, d_6-DMSO) δ 119.3, 119.3, 122.8, 129.1, 129.6, 129.8, 130.0, 131.9, 132.4, 148.2.

7-Methoxynaphthalen-1-yl sulfamate (12)

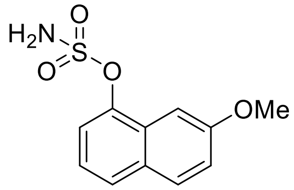

7-Methoxy-1-naphthol (150 mg, 0.86 mmol) and CSI (609 g, 4.3 mmol), column chromatography from 40% EtOAc/petrol (with 2% Et_3N) yielded the sulfamate (65 mg, 30%). **m.p.** 148–149 °C. **^{1}H NMR** (500 MHz, d_6-DMSO) δ 3.91 (s, 3H), 7.24 (dd, J = 8.9, 2.2 Hz, 1H), 7.38 (t, J = 7.9 Hz, 1H), 7.43 (d, J = 1.9 Hz, 1H), 7.48 (d, J = 7.6 Hz, 1H), 7.81 (d, J = 8.1 Hz, 1H), 7.91 (d, J = 9.0 Hz, 1H), 8.16 (s, 2H). **^{13}C NMR** (126 MHz, d_6-DMSO) δ 55.3, 100.4, 118.9, 119.3, 123.1, 126.1, 128.2, 129.6, 129.9, 145.3, 157.9.

6-Cyanonaphthalen-2-yl sulfamate (18)

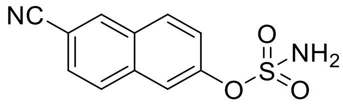

6-Cyano-2-naphthol (300 mg, 1.77 mmol) and CSI (1.25 g, 8.86 mmol), column chromatography from 30% EtOAc/Petrol (with 2% AcOH) yielded the sulfamate (210 mg, 48%). **m.p.** 198 °C. **^{1}H NMR** (500 MHz, d_6-DMSO) δ 7.60 (dd, J = 8.9, 1.5 Hz, 1H), 7.85 (d, J = 8.6 Hz, 1H), 7.97 (s, 1H), 8.23–8.15 (m, 4H), 8.64 (s, 1H). **^{13}C NMR** (126 MHz, d_6-DMSO) δ 108.55, 119.02, 119.30, 123.41, 127.24, 129.32, 130.15, 130.76, 134.26, 134.98, 150.10.

Ethyl 4,6-*O*-benzylidene-1-thio-β-D-galactoside (26)

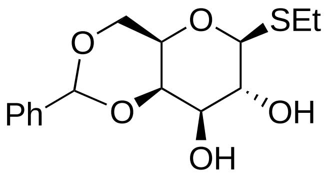

Purchased from GlycoUniverse.

^{1}**H NMR** (400 MHz, MeOD) δ 7.45 – 7.36 (m, 2H), 7.29 – 7.17 (m, 3H), 5.47 (s, 1H), 4.29 (d, *J* = 9.3 Hz, 1H), 4.12 (dd, *J* = 3.5, 1.1 Hz, 1H), 4.09 – 3.95 (m, 2H), 3.64 – 3.51 (m, 1H), 3.49 (dd, *J* = 9.4, 3.5 Hz, 1H), 3.44 (q, *J* = 1.5 Hz, 1H), 2.76 – 2.53 (m, 2H), 1.20 (t, *J* = 7.4 Hz, 3H). ^{13}**C NMR** (101 MHz, MeOD) δ 139.8, 129.9, 129.0, 127.6, 102.4, 86.9, 77.8, 75.1, 71.4, 70.6, 70.4, 49.6, 49.5, 49.4, 49.3, 49.2, 49.1, 49.0, 48.9, 48.8, 48.6, 48.4, 24.5, 15.6.

Ethyl 2,3-di-*O*-acetyl-4,6-*O*-benzylidene-1-thio-β-D-galactoside (27)

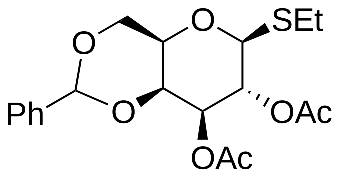

Ethyl 4,6-*O*-benzylidene-1-thio-β-D-galactoside **26** (5 g, 16.0 mmol) was suspended in CH_2Cl_2 (0.1M, 160 mL), pyridine (5.2 ml, 64.0 mmol, 4 eq.), and acetic acid (3.0 mL, 32.0 mmol, 2 eq.). Subsequently, a catalytic quantity of 4-(dimethylamino)pyridine (20 mg, 0.16 mmol, 0.01 eq.) was added. The mixture was left to stir at room temperature for 5 hours, and concentrated under vacuum to afford a yellow gel. Purification by flash chromatography (SiO_2, Hexane/Ethyl acetate) yielded title compound **27** (5.0 g, 79%) as a white amorphous solid. R_f = 0.35 (Hexane/Ethyl acetate, 80:20, v/v).

^{1}**H NMR** (400 MHz, $CDCl_3$) δ 7.46 – 7.37 (m, 2H), 7.36 – 7.25 (m, 3H), 5.45 – 5.35 (m, 2H), 4.91 (dd, *J* = 10.0, 3.5 Hz, 1H), 4.39 (d, *J* = 9.9 Hz, 1H), 4.34 (df, *J* = 3.6, 1.0 Hz, 1H), 4.26 (dd, *J* = 12.5, 1.7 Hz, 1H), 3.94 (dd, *J* = 12.5, 1.7 Hz, 1H), 3.49 (q, *J* = 1.4 Hz, 1H), 2.89 – 2.74 (m, 1H), 2.66 (dq, *J* = 12.2, 7.5 Hz, 1H), 2.00 (d, *J* = 4.1 Hz, 6H), 1.27 – 1.16 (m, 3H). ^{13}**C NMR** (101 MHz, $CDCl_3$) δ 170.8, 169.6, 137.6, 129.3, 128.3, 126.5, 101.3, 82.8, 73.7, 73.1, 69.8, 69.2, 66.6, 22.9, 21.0, 21.0, 14.9. **HRMS** calcd. $C_{19}H_{24}NaO_7S$ for $[M+Na]^+$ 419.1140, found 419.1151.

Methyl 4,6-*O*-benzylidene-β-D-galactoside (28)

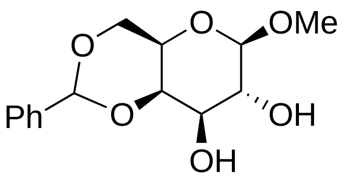

Ethyl 2,3-di-*O*-acetyl-4,6-*O*-benzylidene-1-thio-β-D-galactoside **27** (3.2 g, 8 mmol) was dissolved in anhydrous CH_2Cl_2 (0.05M, 160 mL), methanol (3.2 mL, 60 mmol, 10 eq) and cooled to 0°C. Next, NIS (3.6 g, 16 mmol, 10 eq) and TMSOTf (0.14 mL, 1.60 mmol, 0.2 eq) were added. Once complete, the reaction was quenched by the addition of triethylamine, filtered and washed with 10% aqueous $Na_2S_2O_3$, saturated aqueous $NaHCO_3$, and brine. The combined organic layer was dried over $MgSO_4$, filtered, concentrated, and purified by flash silica column chromatography (SiO_2, Hexane/EtOAc) to afford the intermediate glycoside. Next the methyl 2,3-di-*O*-acetyl-4,6-*O*-benzylidene-β-D-galactoside was dissolved in anhydrous CH_2Cl_2 (0.05M, 160 mL), cooled to 0°C, and sodium methoxide (0.5M) was added until the reaction reached pH 8. The temperature was allowed raised to room temperature and stirred complete (≈5 h). The reaction was tracked using thin layer chromatography (TLC) and once complete, it was quenched by the addition of Amberlite™ IR120(H), filtered, and concentrated. Purification by flash chromatography (SiO_2, CH_2Cl_2/Methanol) afforded **28** (1.6g, 65%) as an amorphous solid. R_f = 0.3 (CH_2Cl_2/Methanol, 90:10, v/v).

^{1}H NMR ^{1}H NMR (400 MHz, MeOD) δ 7.56 – 7.51 (m, 2H), 7.34 (d, *J* = 6.9 Hz, 2H), 5.60 (d, *J* = 1.5 Hz, 1H), 4.28 – 4.11 (m, 4H), 3.63 – 3.59 (m, 1H), 3.55 (s, 3H). **^{13}C NMR** (101 MHz, MeOD) δ 139.7, 129.9, 129.0, 129.0, 127.6, 127.5, 105.8, 102.5, 77.5, 73.7, 72.0, 70.2, 68.0, 57.4. **HRMS** calcd. $C_{14}H_{19}O_6$ for $[M+H]^+$ 283.1182, found 283.1210.

Methyl 3-*O*-(ethenylsulfonyl)-β-D-galactoside (25)

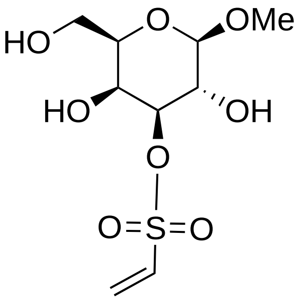

Methyl 4,6-*O*-benzylidene-β-D-galactoside (25 mg, 0.089 mmol) was dissolved in CH_2Cl_2 (0.1M, 1.0 mL) and 2-chloroethanesulfonyl chloride (10 µL, 0.095 mmol, 1.1 eq) and *N*,*N*-diisopropylethylamine (45 µL, 0.267 mmol, 3 eq) were added. After 10 mins the reaction mixture was concentrated and the crude residue was purified by flash chromatography on silica gel (Hexane/ethyl acetate) to

provide vinyl sulfonate intermediate. The crude material was dissolved in CH_2Cl_2 (25 mL) and 0.1M HCl (25 mL) was added. The reaction was stirred at room temperature until complete hydrolysis of the benzylidene acetal. The compound was then purified by HPLC (HyberCarb column, method 1) to isolate compound **25** (4 mg, 16 %)

^{1}H NMR (400 MHz, D_2O) δ 6.80 (dd, *J* = 16.6, 10.1 Hz, 1H), 6.48 (d, *J* = 16.6 Hz, 1H), 6.30 (d, *J* = 10.0 Hz, 1H), 4.53 (dd, *J* = 9.9, 3.4 Hz, 1H), 4.36 (d, *J* = 7.9 Hz, 1H), 4.17 (d, *J* = 3.4 Hz, 1H), 3.83 – 3.64 (m, 4H), 3.53 (s, 3H). **^{13}C NMR** (101 MHz, D_2O) δ 132.4, 131.4, 103.1, 83.1, 74.4, 68.3, 67.2, 60.5, 57.2. **HRMS** QTOF-MS: calcd. $C_9H_{16}NaO_8S$ for $[M+Na]^+$ 307.0464, found 307.0601.

Methyl 3-*O*-sulfamoyl-β-D-galactoside (20)

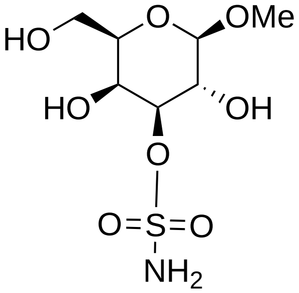

Methyl 4,6-O-benzylidene-β-D-galactoside (45 mg, 0.6 mmol) was dissolved in and pyridine (0.1M, 6 mL). Then *N*-benzyl sulfamoyl chloride (30 µL, 0.175 mmol, 1.1 eq) was added to the reaction.[6] Once complete, the reaction was quenched by the addition of methanol, concentrated and purified by column chromatography (SiO_2, Hexane/Ethyl acetate) to afford the 3-*O*-sulfonamide intermediate compound. At this stage also the 2-*O*-sulfonamide was separated. Next the 3-*O*-sulfonamide intermediate was dissolved in THF:t-BuOH:H_2O (3 mL, 60:10:30, v/v/v) and 5% Pd/C (200 mg) was added. The reaction put under an atmosphere of hydrogen and left overnight. The reaction was then filtered through a pad of Celite®, and concentrated. The material was then purified by reverse-phase HPLC (HyberCarb column, method 1) to yield the title compound **23** (5 mg, 11%).

^{1}H NMR (400 MHz, D_2O) δ 4.51 (dd, *J* = 9.9, 3.4 Hz, 1H), 4.41 (d, *J* = 7.9 Hz, 1H), 4.28 (dd, *J* = 3.4, 0.9 Hz, 1H), 3.83 – 3.65 (m, 4H), 3.57 (s, 3H). **^{13}C NMR** (101 MHz, D_2O) δ 103.2, 82.1, 74.5, 68.5, 66.8, 60.6, 57.2. **HRMS** QTOF-MS: calcd. $C_7H_{15}NNaO_8S$ for $[M+Na]^+$ 296.0416, found 296.0431.

Methyl 2-*O*-sulfamoyl-β-D-galactoside (21)

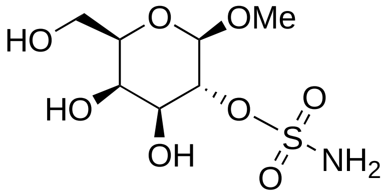

The 2-*O*-sulfamate intermediate was dissolved in THF:t-BuOH:H_2O (3 mL, 60:10:30, v/v/v) and 5% Pd/C (200 mg) was added. The reaction left overnight under an atmosphere of hydrogen. The suspension was filtered through a pad of Celite®, and concentrated. The material was purified by reverse-phase HPLC (HyberCarb column, method 1) to yield the title compound **24** (1 mg, 2.3%).

^{1}H NMR (400 MHz, D_2O) δ 4.51 (d, *J* = 7.7 Hz, 1H), 4.31 (td, *J* = 9.8, 7.9 Hz, 1H), 3.96 (d, *J* = 3.5 Hz, 1H), 3.84 (dd, *J* = 9.8, 3.4 Hz, 1H), 3.79 – 3.65 (m, 3H), 3.54 (s, 4H). **^{13}C NMR** (101 MHz, D_2O) δ 101.3, 81.1, 75.1, 70.8, 68.9, 60.7, 57.2. **HRMS** calcd. $C_7H_{15}NNaO_8S$ for $[M+Na]^+$ 296.041, found 296.0429.

NMR Spectra

4-Methoxynaphthalen-1-yl sulfamate (10)

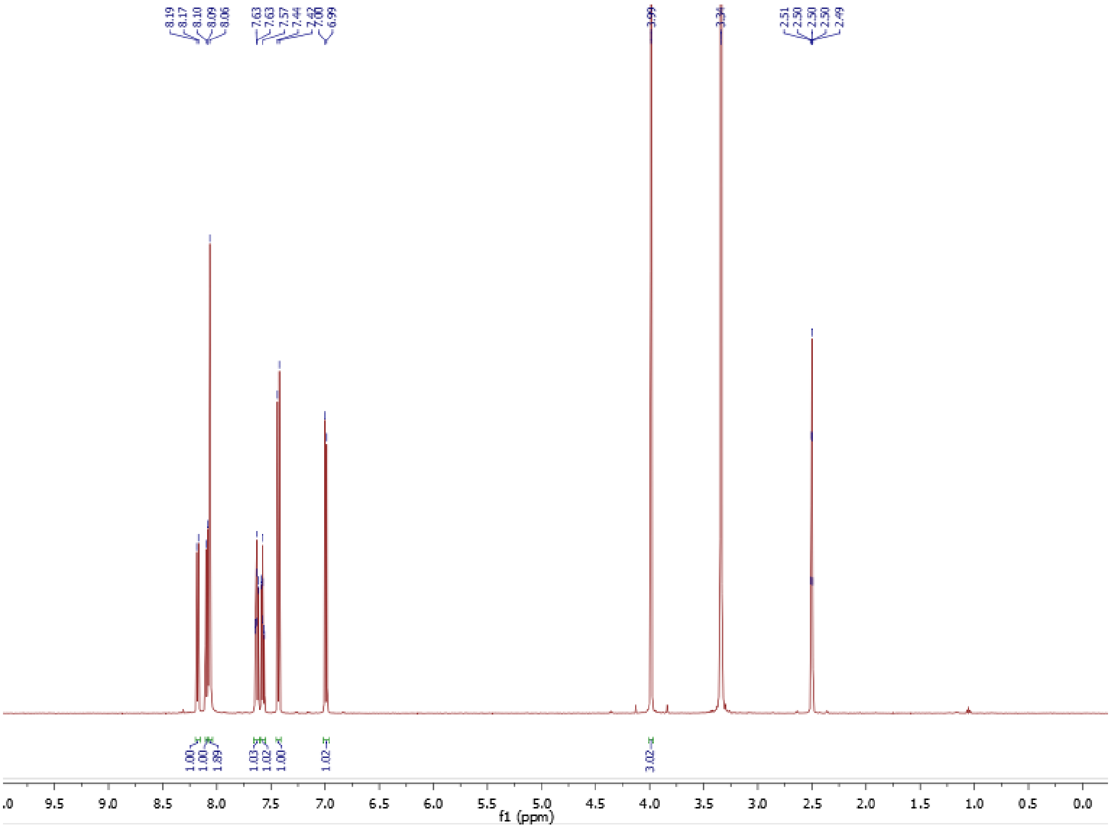

^{1}H NMR

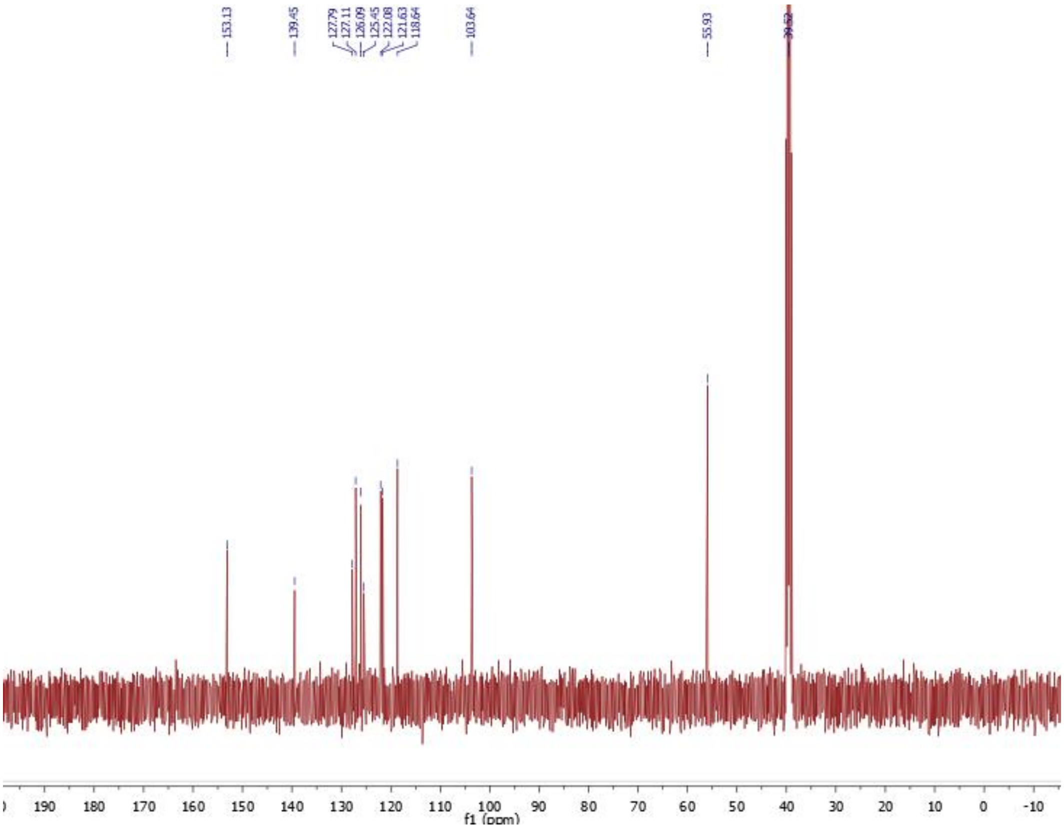

^{13}C NMR

4-Bromonaphthalen-1-yl sulfamate (11)

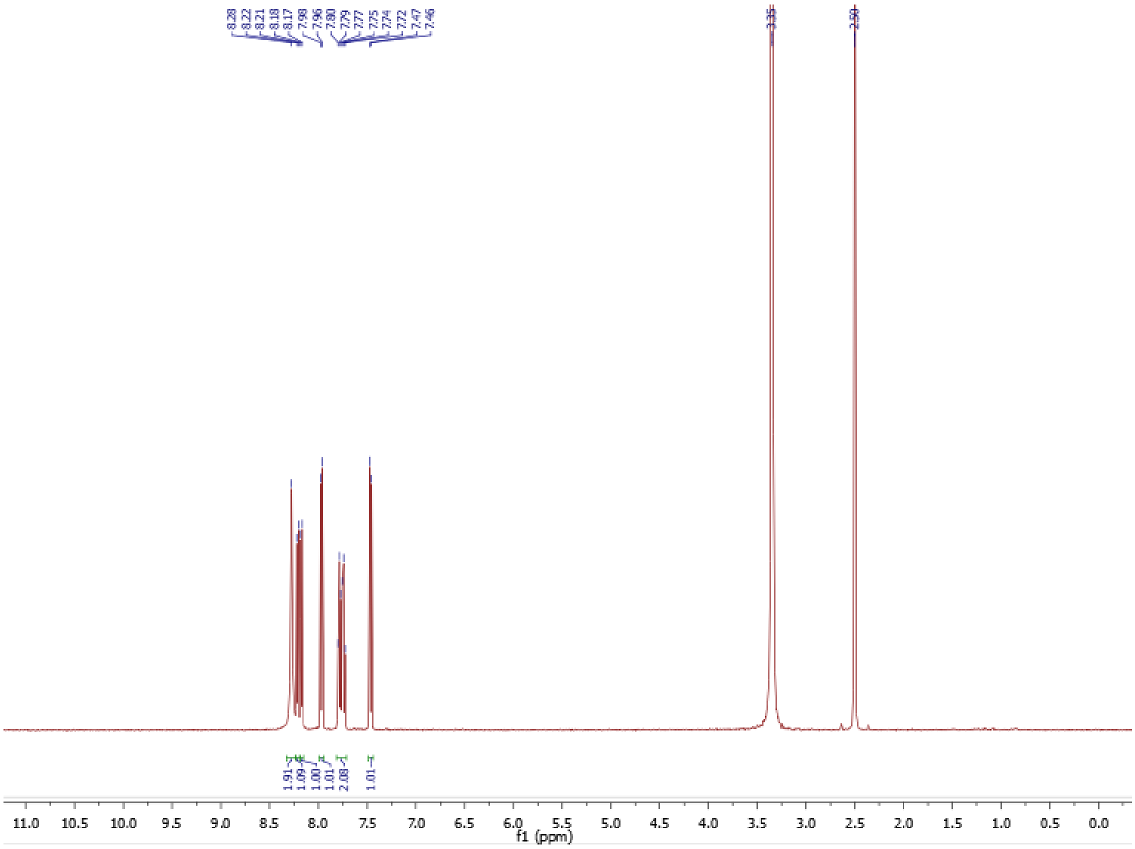

^{1}H NMR

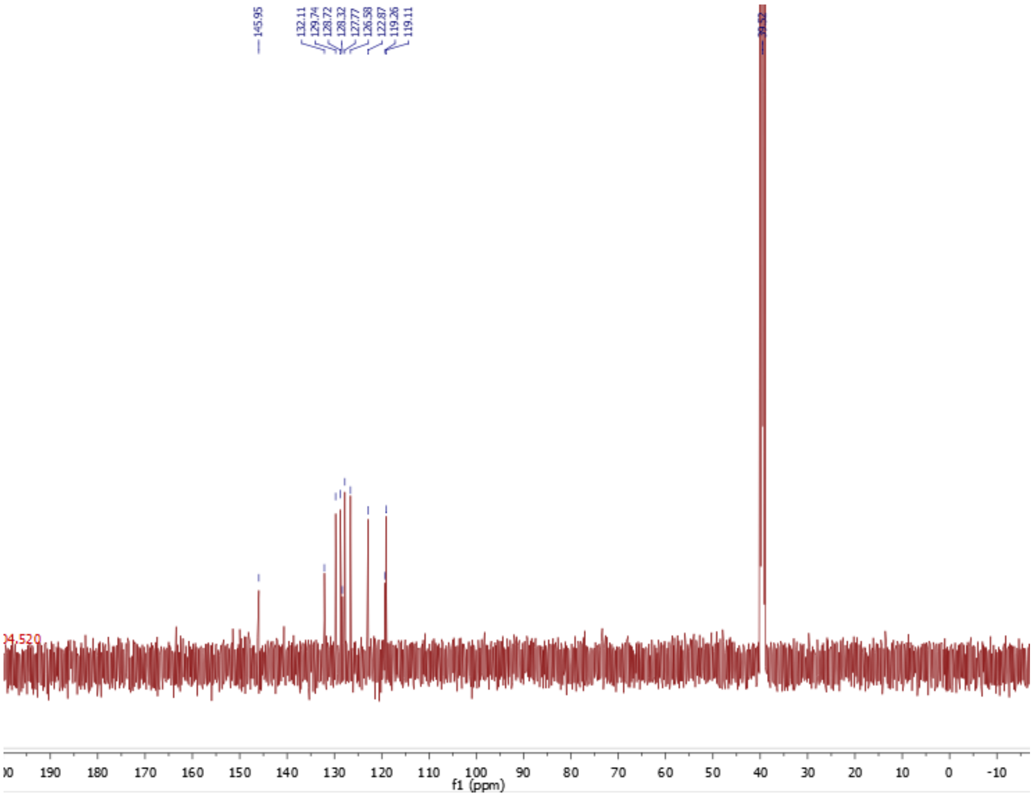

^{13}C NMR

7-Methoxynaphthalen-1-yl sulfamate (12)

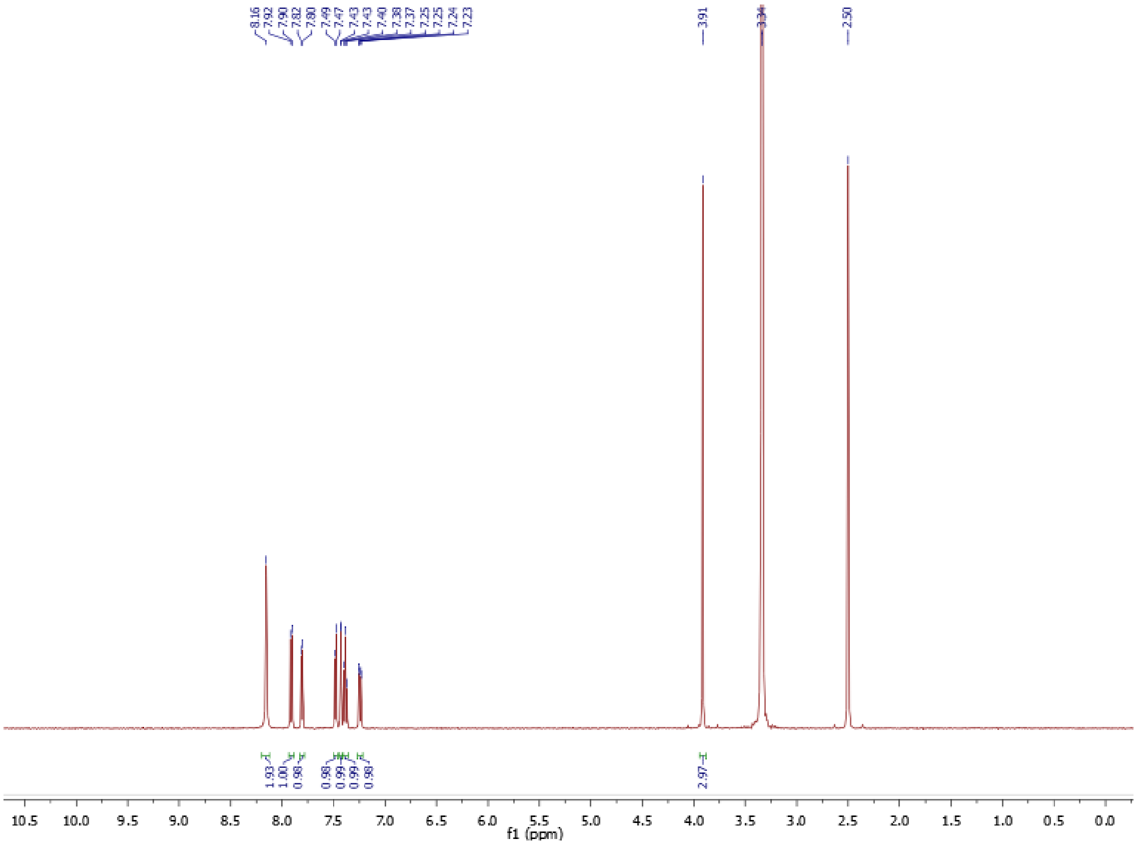

^{1}H NMR

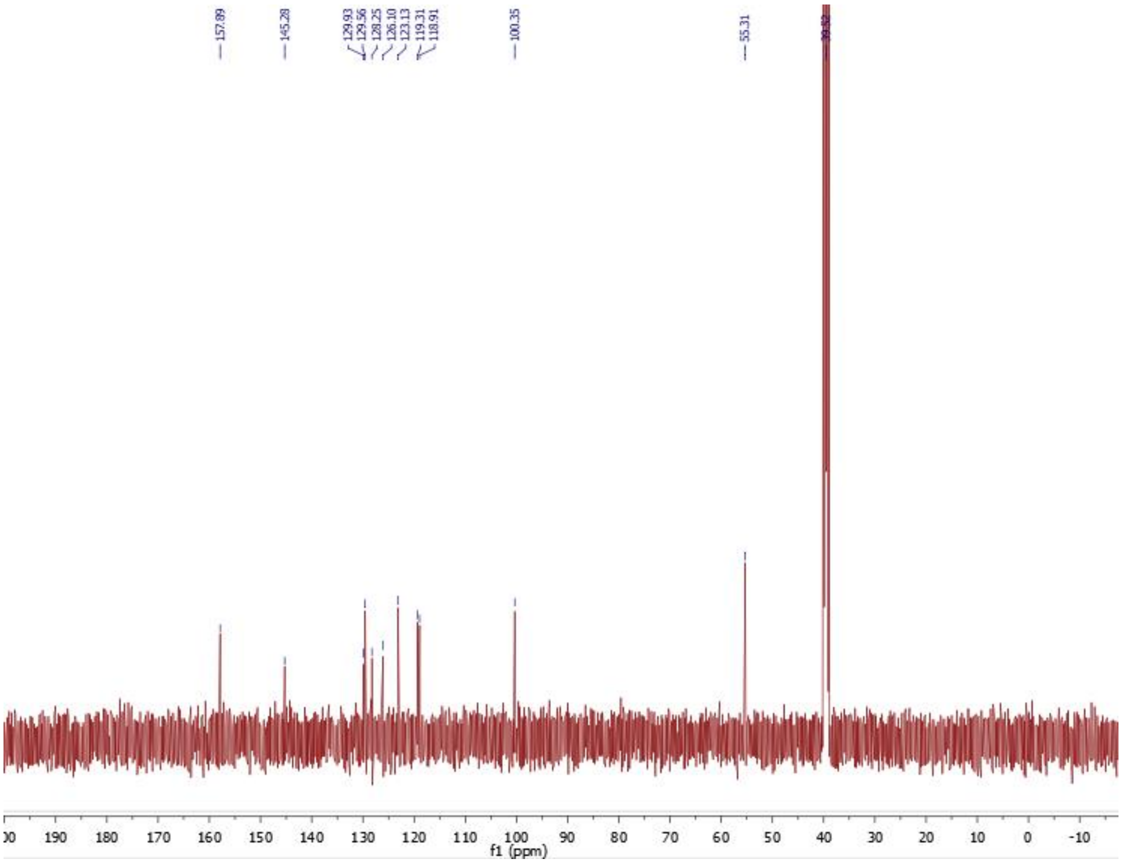

^{13}C NMR

7-Methoxynaphthalen-2-yl sulfamate (13)

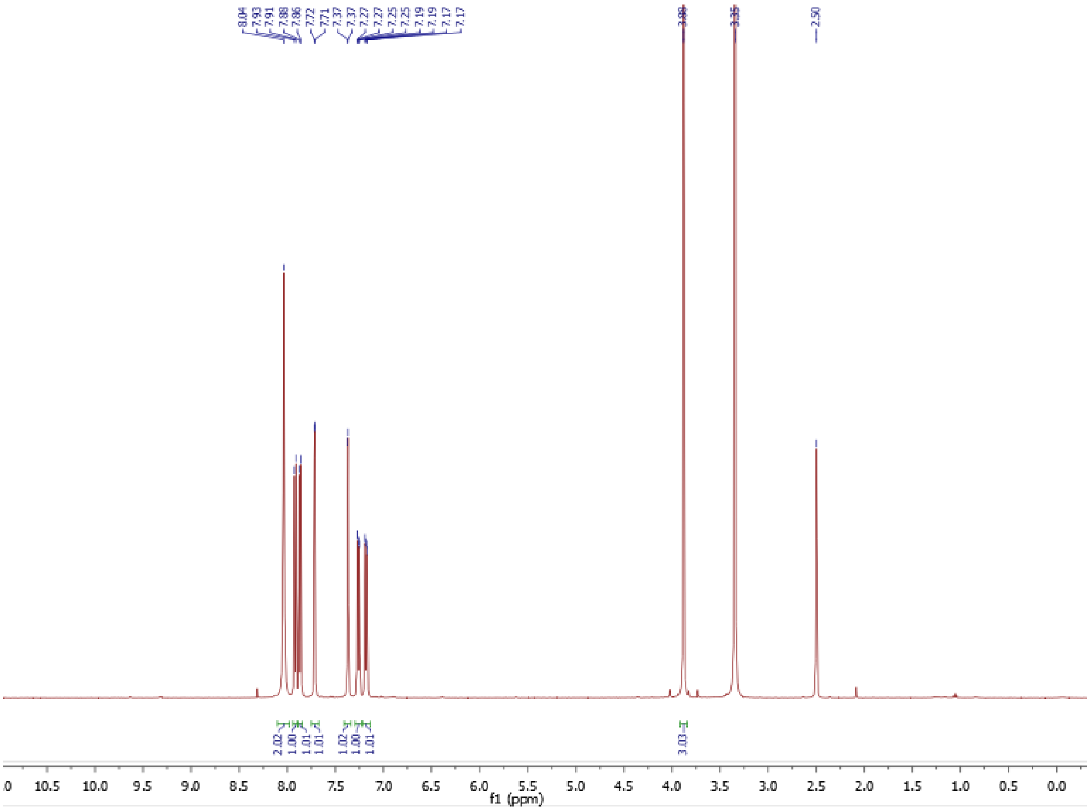

^{1}H NMR

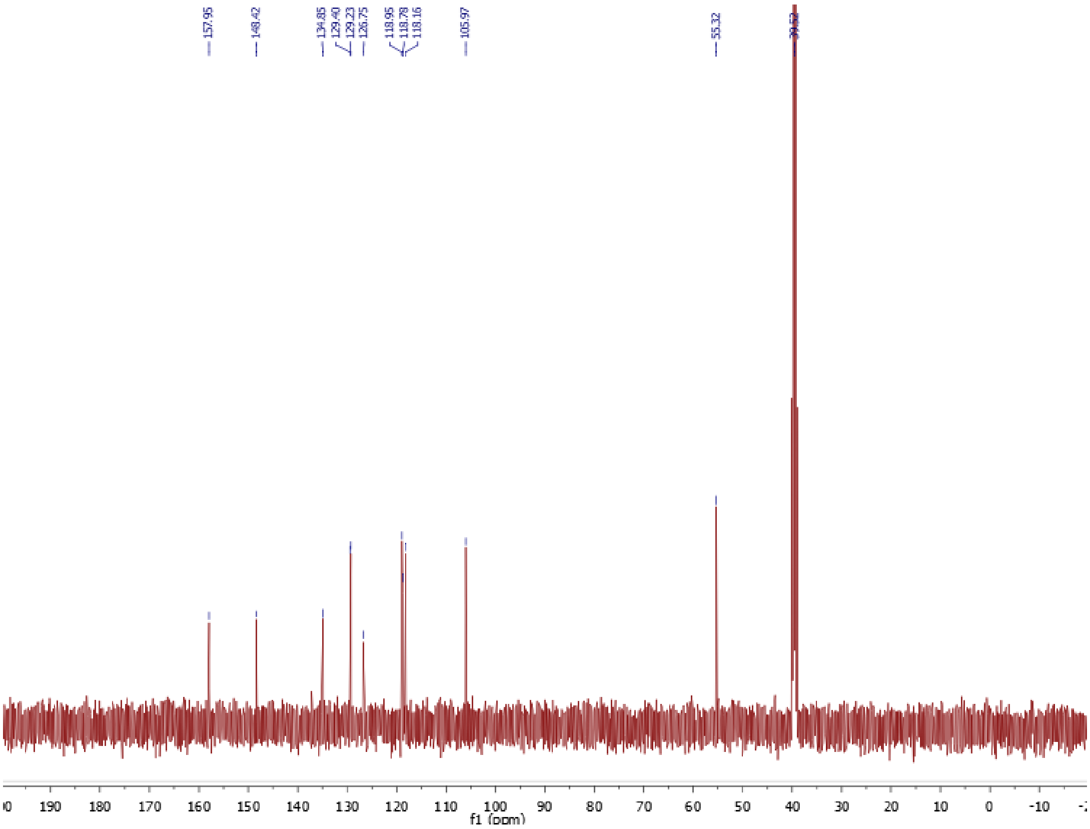

^{13}C NMR

6-Methoxynaphthalen-2-yl sulfamate (14)

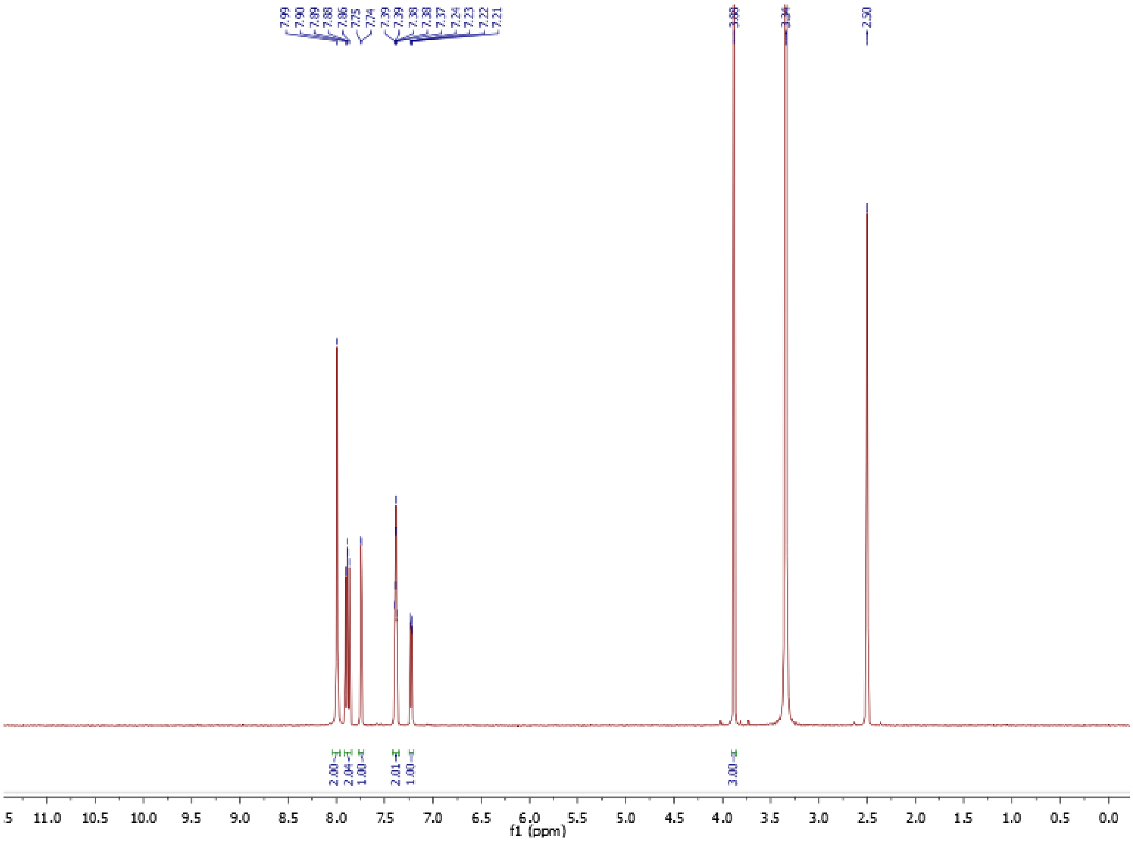

^{1}H NMR

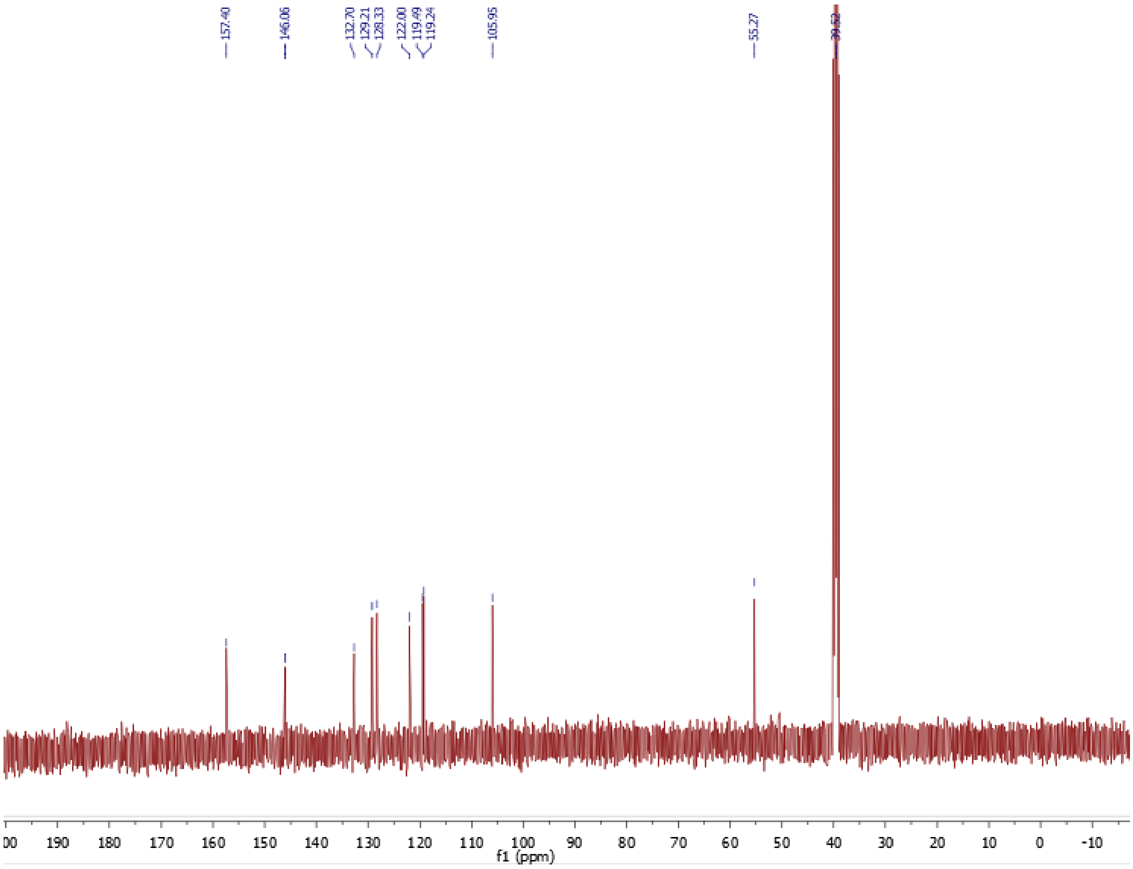

^{13}C NMR

3-Methoxynaphthalen-2-yl sulfamate (17)

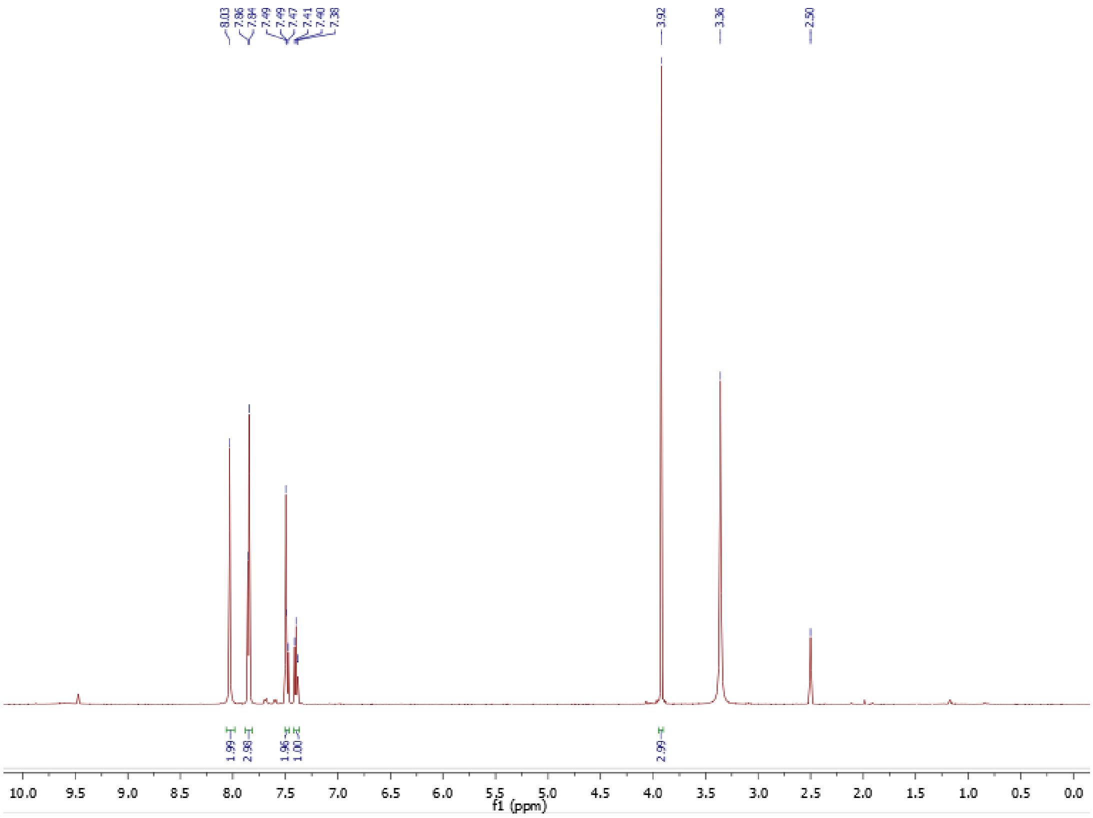

¹H NMR

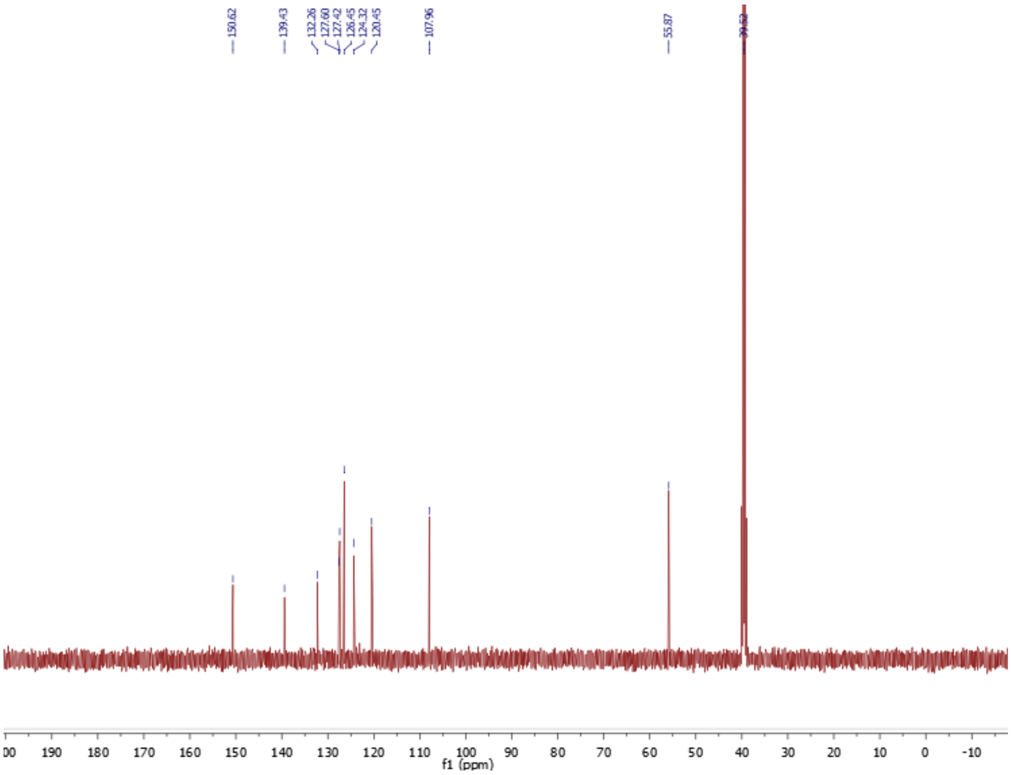

¹³C NMR

6-Bromonaphthalen-2-yl sulfamate (15)

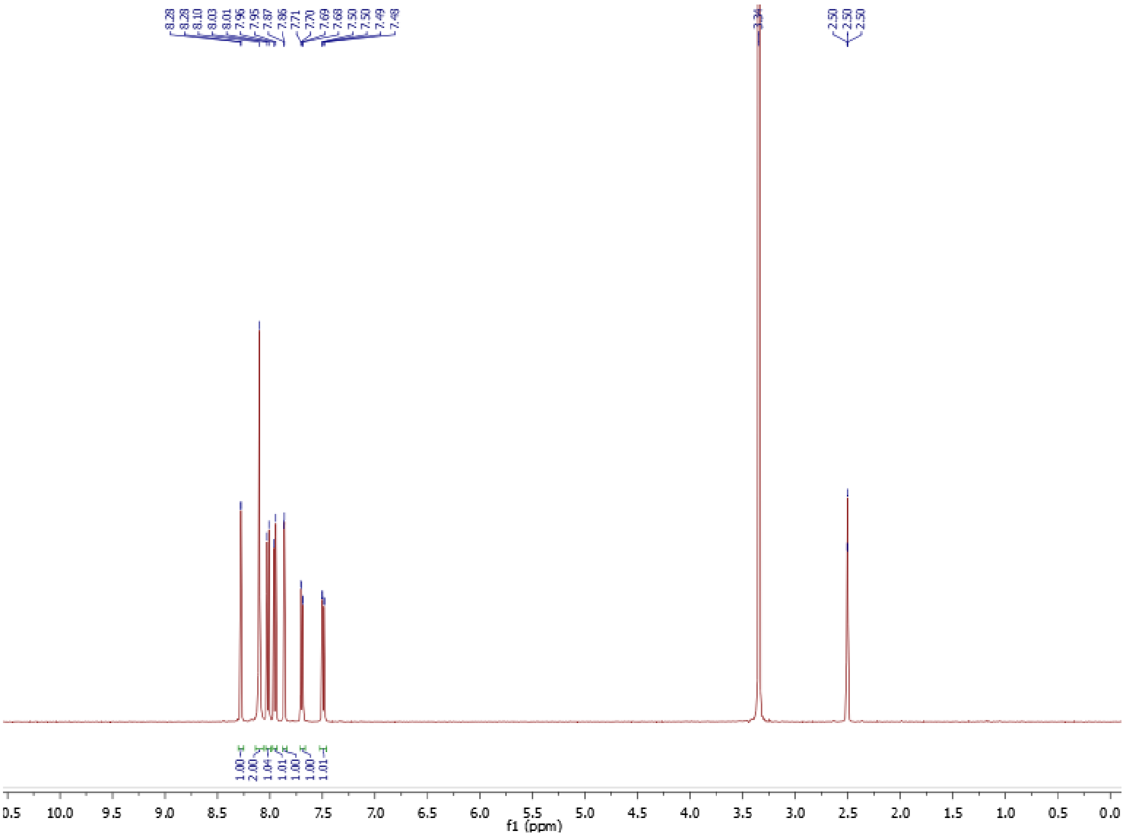

^{1}H NMR

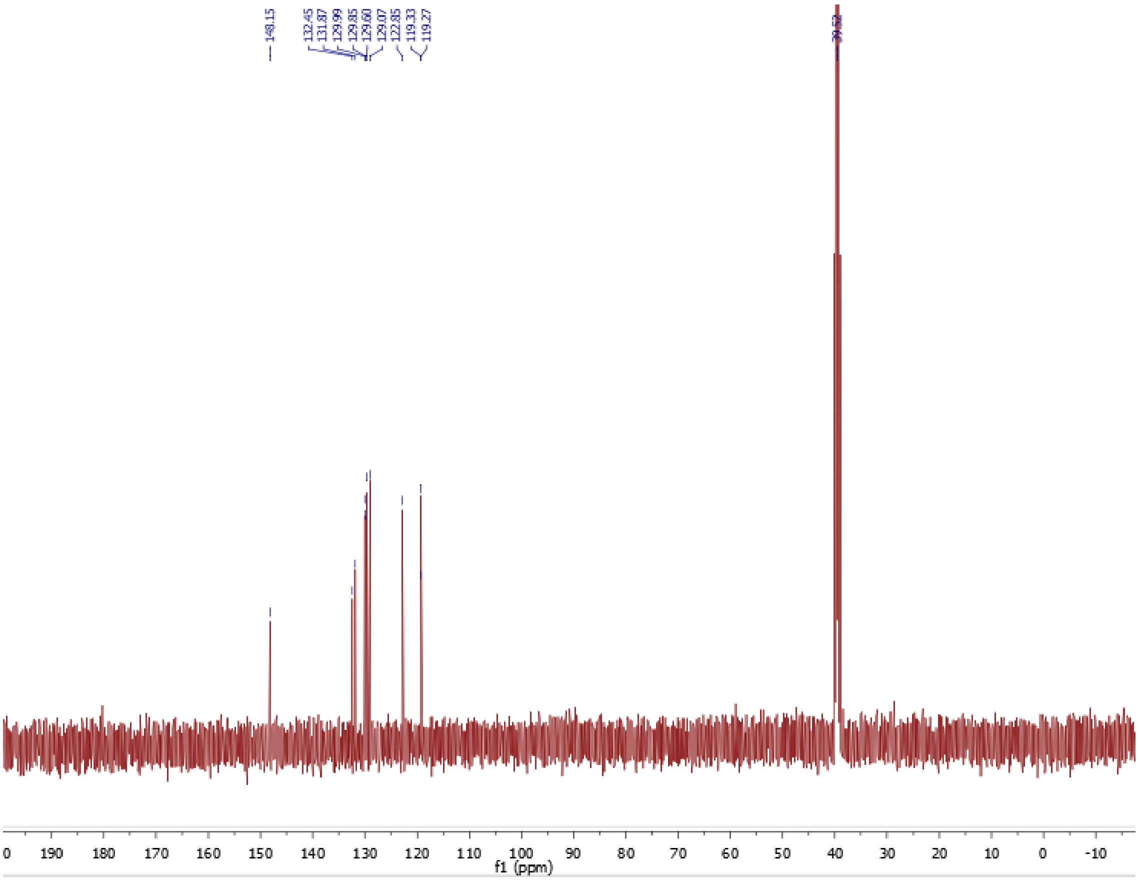

^{13}C NMR

6-Cyanonaphthalen-2-yl sulfamate (18)

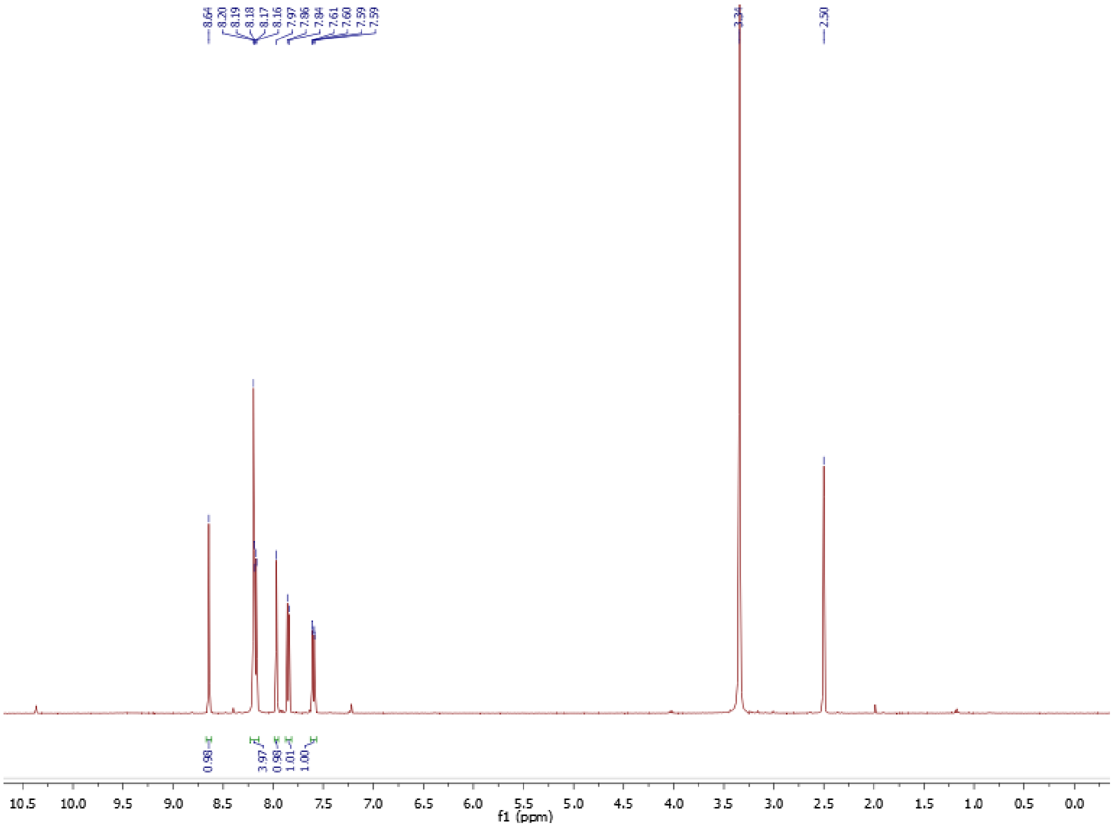

^{1}H NMR

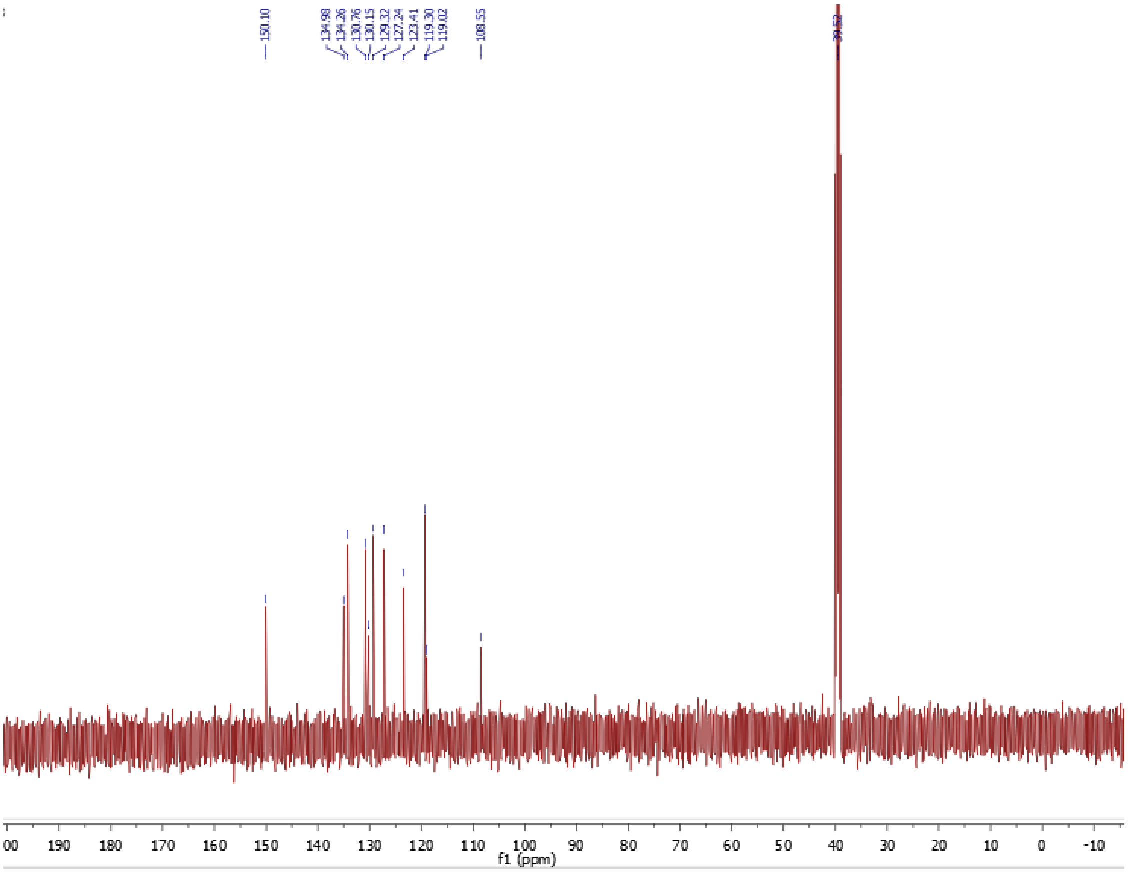

^{13}C NMR

***N*-Methyl COUMATE (8)**

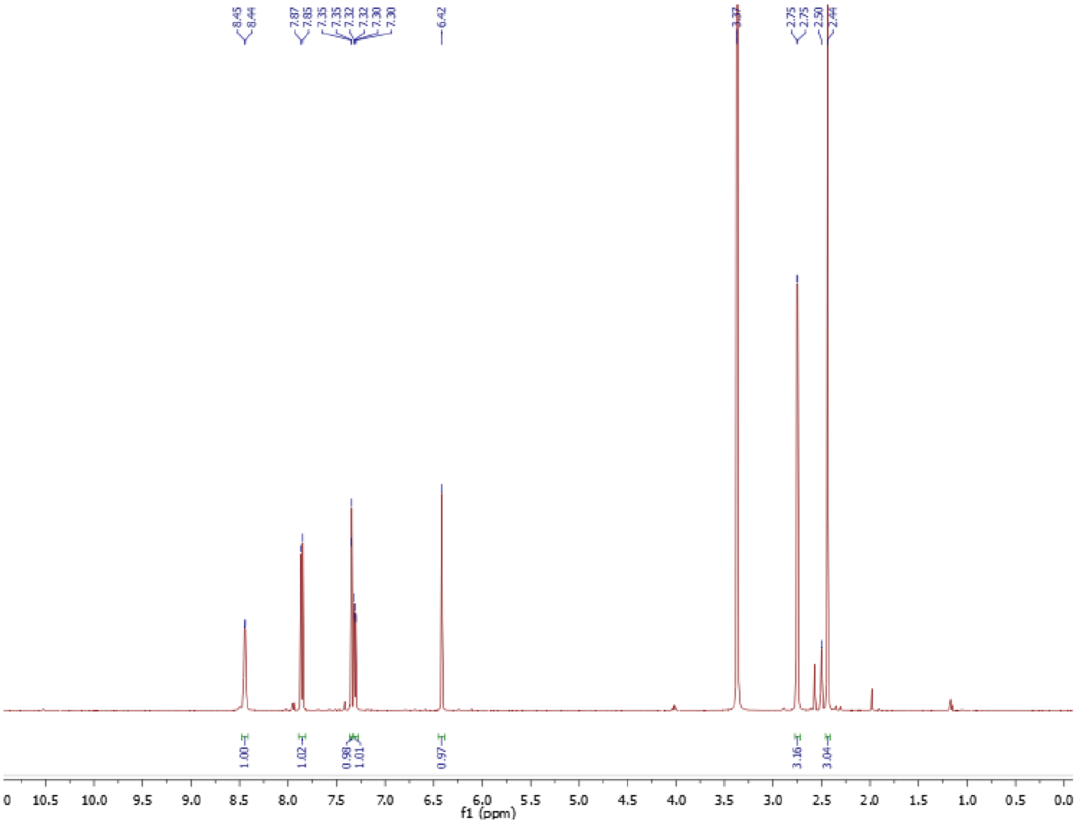

^{1}H NMR

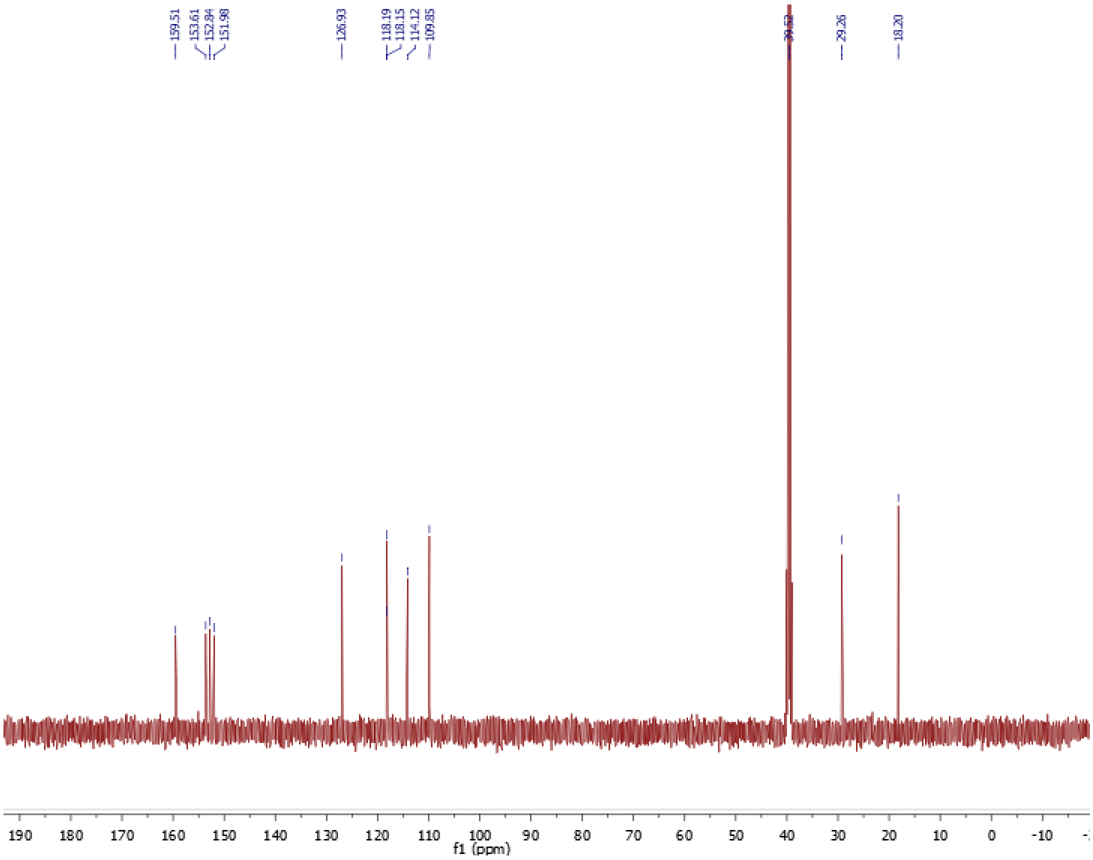

^{13}C NMR

Methyl 3-*O*-sulfamoyl-β-D-galactoside (20)

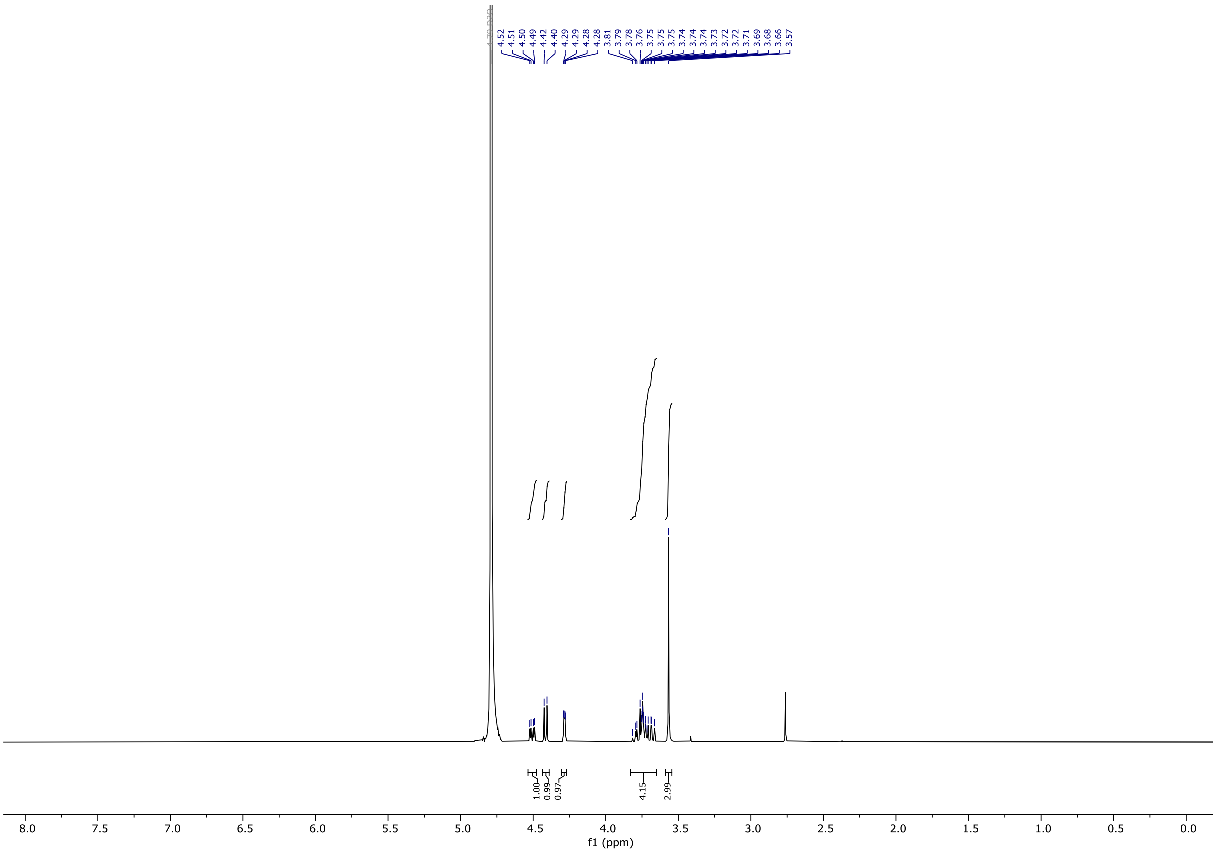

^{1}H NMR

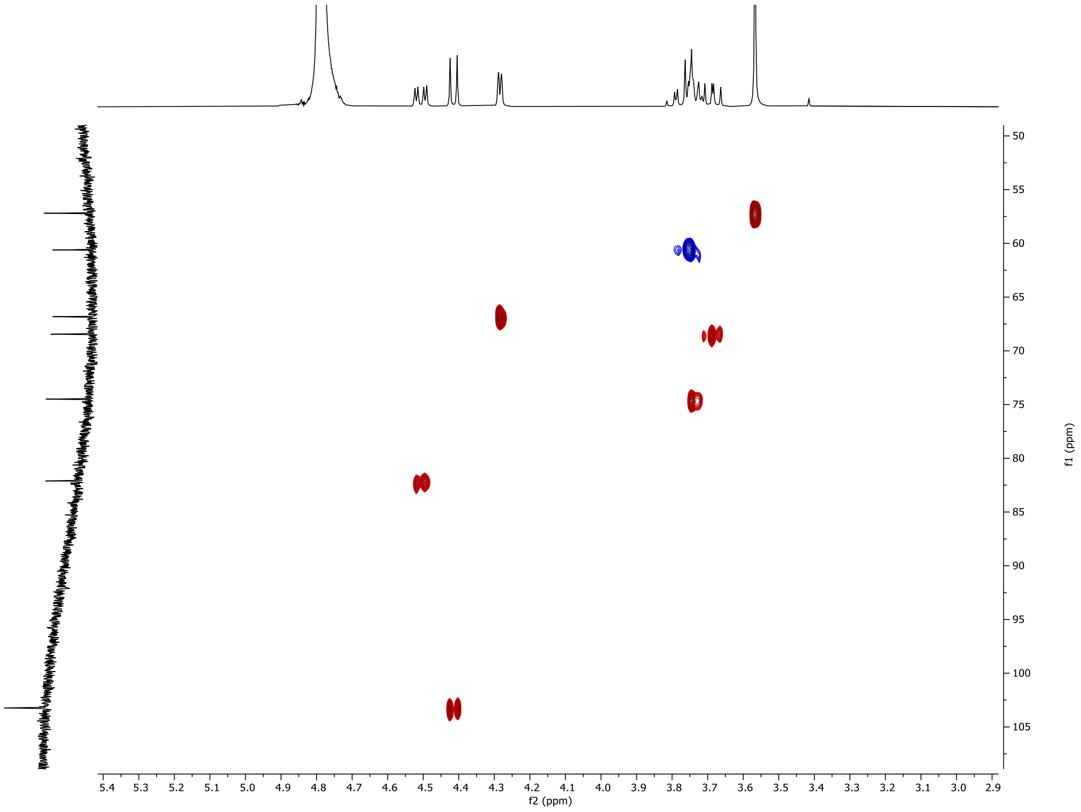

^{1}H-^{13}C NMR

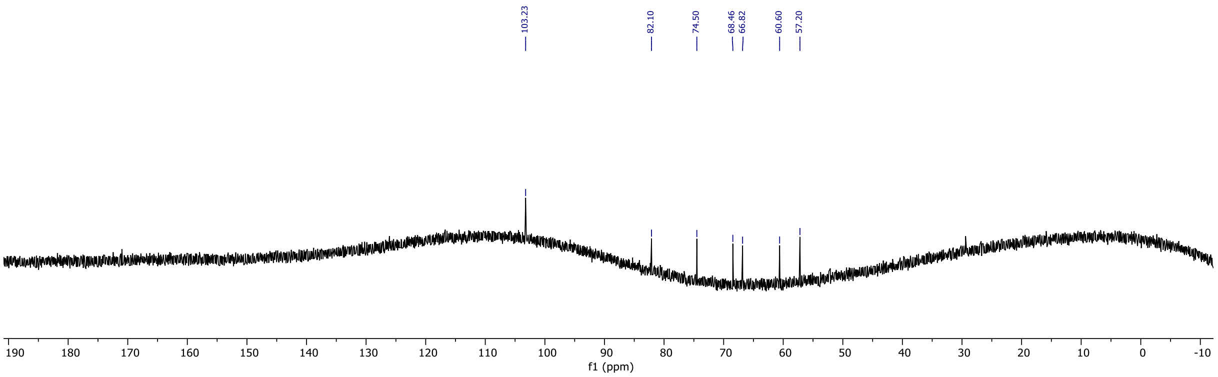

103.23
82.10
74.50
68.46
66.82
60.60
57.20
190 180 170 160 150 140 130 120 110 100 90 80 70 60 50 40 30 20 10 0 -10
f1 (ppm)

^{13}C NMR

Methyl 2-*O*-sulfamoyl-β-D-galactoside (21)

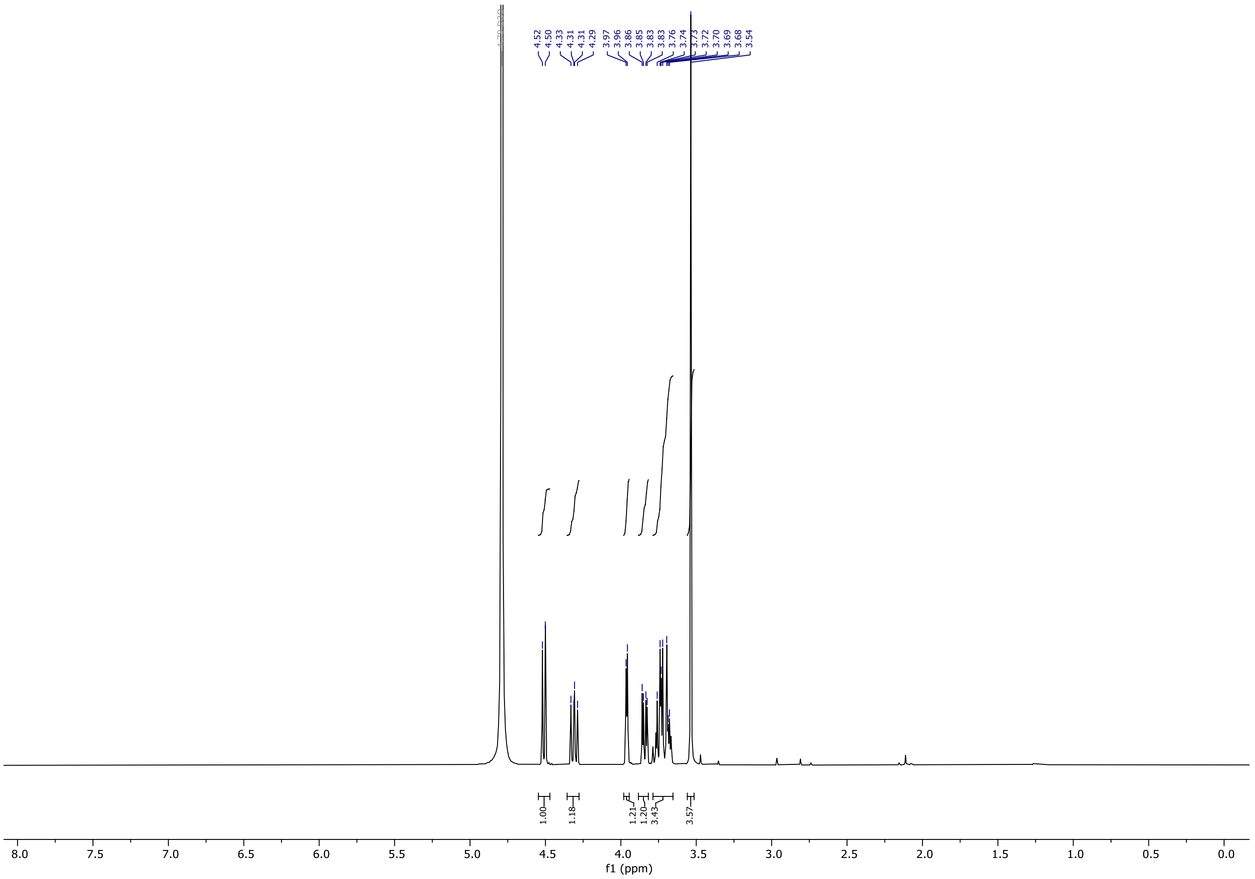

^{1}H NMR

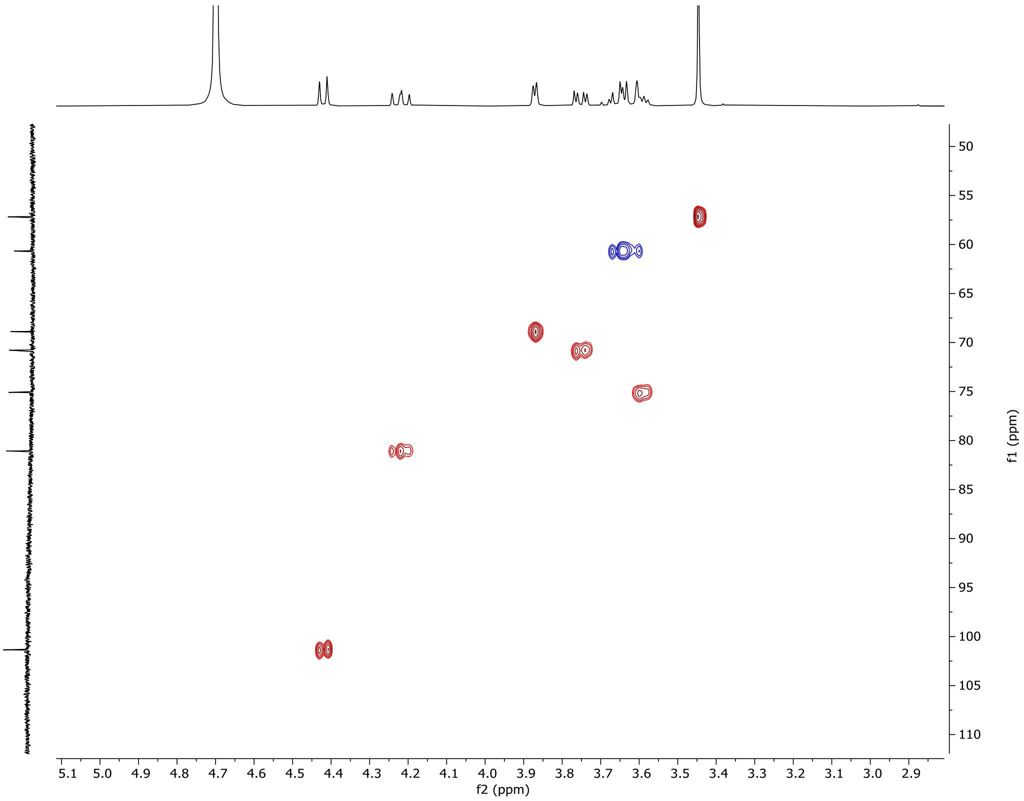

^{1}H- ^{13}C HSQC

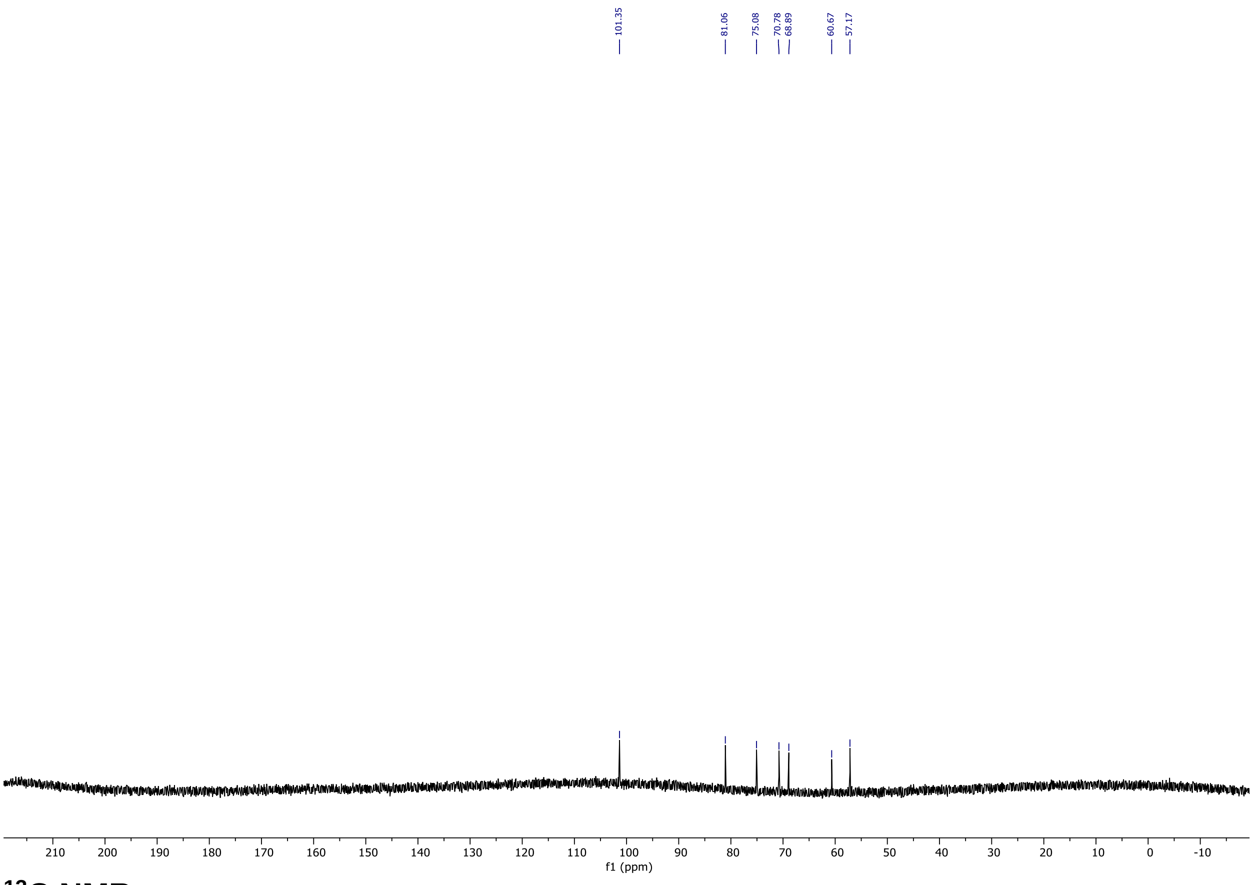
101.35
81.06
75.08
70.78
68.89
60.67
57.17
210 200 190 180 170 160 150 140 130 120 110 100 90 80 70 60 50 40 30 20 10 0 -10
f1 (ppm)

^{13}C NMR

Methyl 3-*O*-(ethenylsulfonyl)-β-D-galactoside (25)

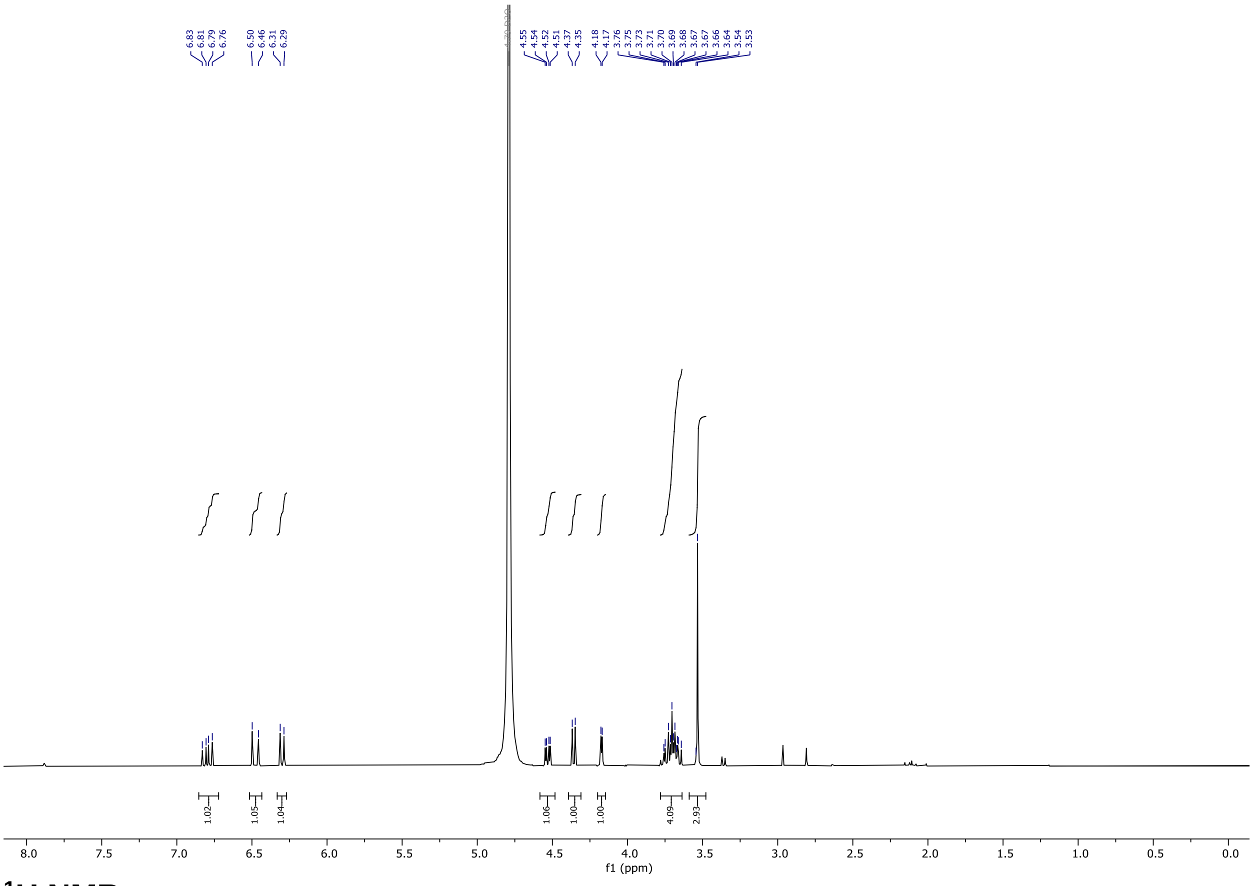

^{1}H NMR

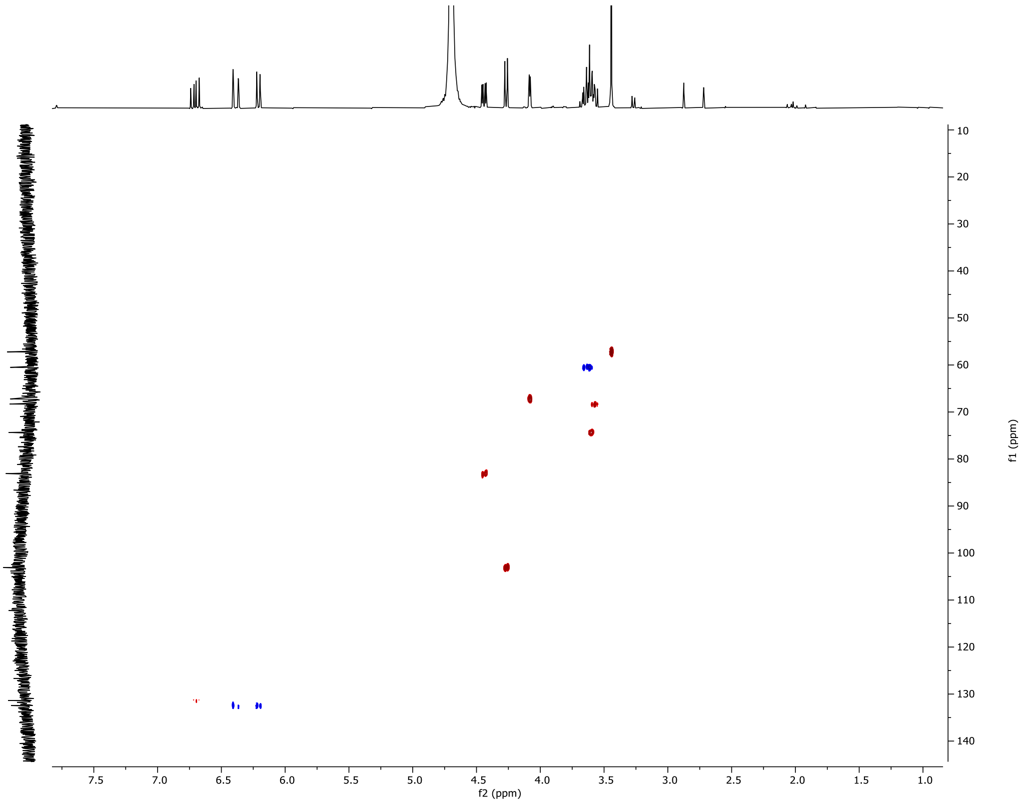

^{1}H-^{13}C HSQC NMR

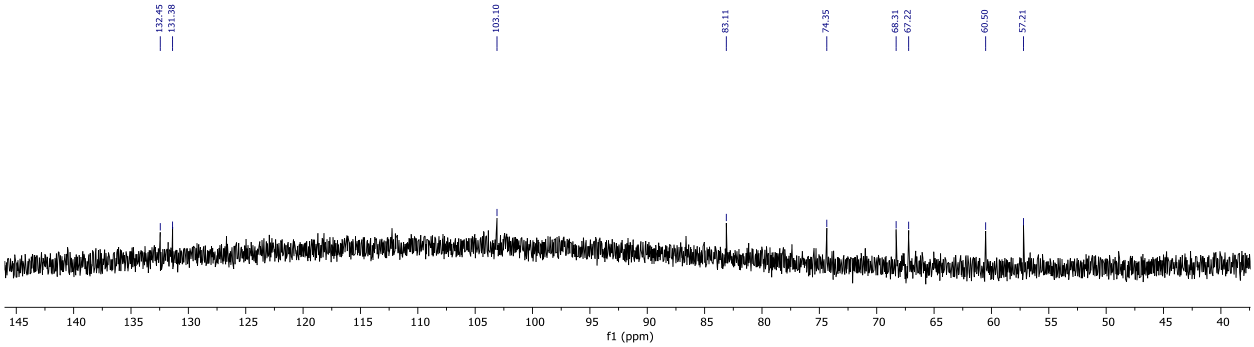
132.45
131.38
103.10
83.11
74.35
68.31
67.22
60.50
57.21
145
140
135
130
125
120
115
110
105
100
95
90
85
80
75
70
65
60
55
50
45
40
f1 (ppm)

^{13}C NMR

Ethyl 4,6-*O*-benzylidene-1-thio-β-D-galactoside (26)

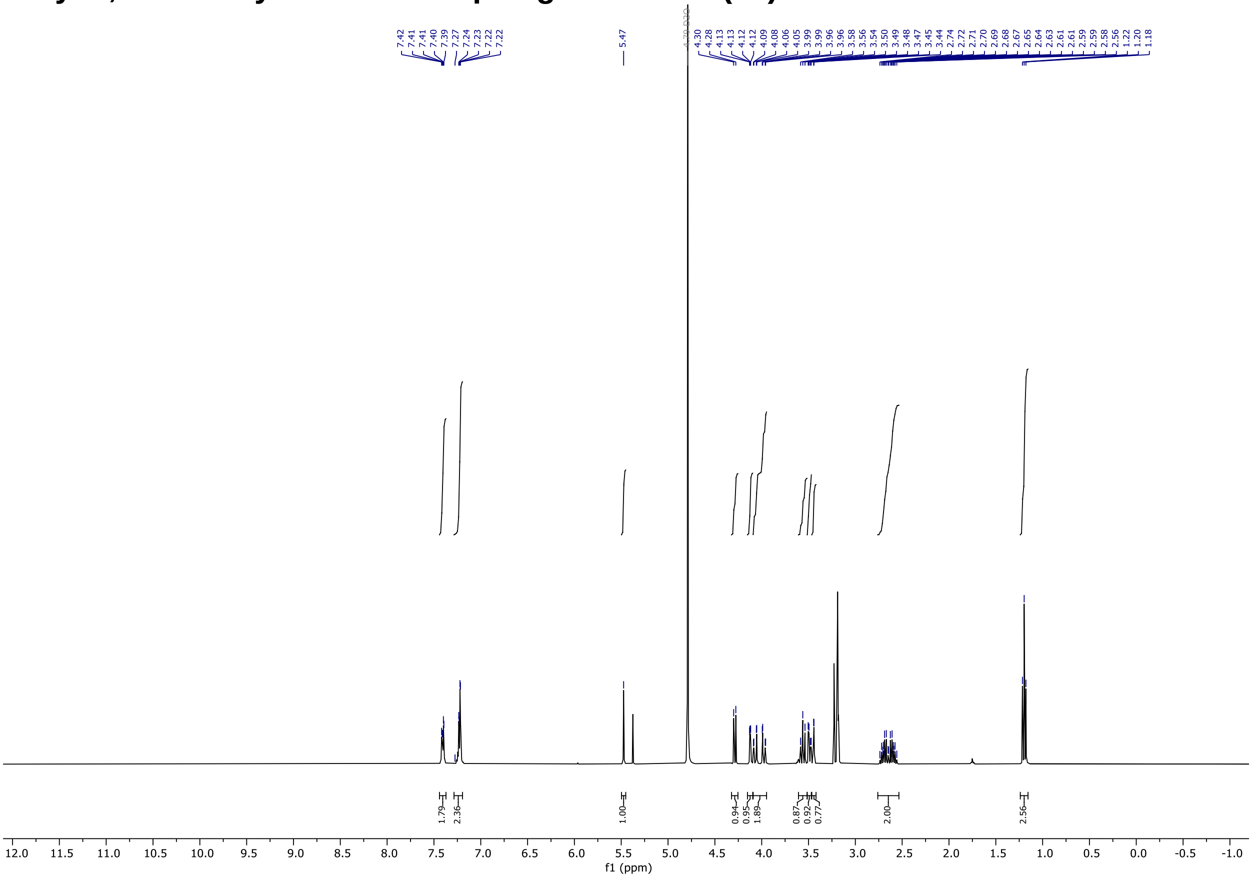

^{1}H NMR

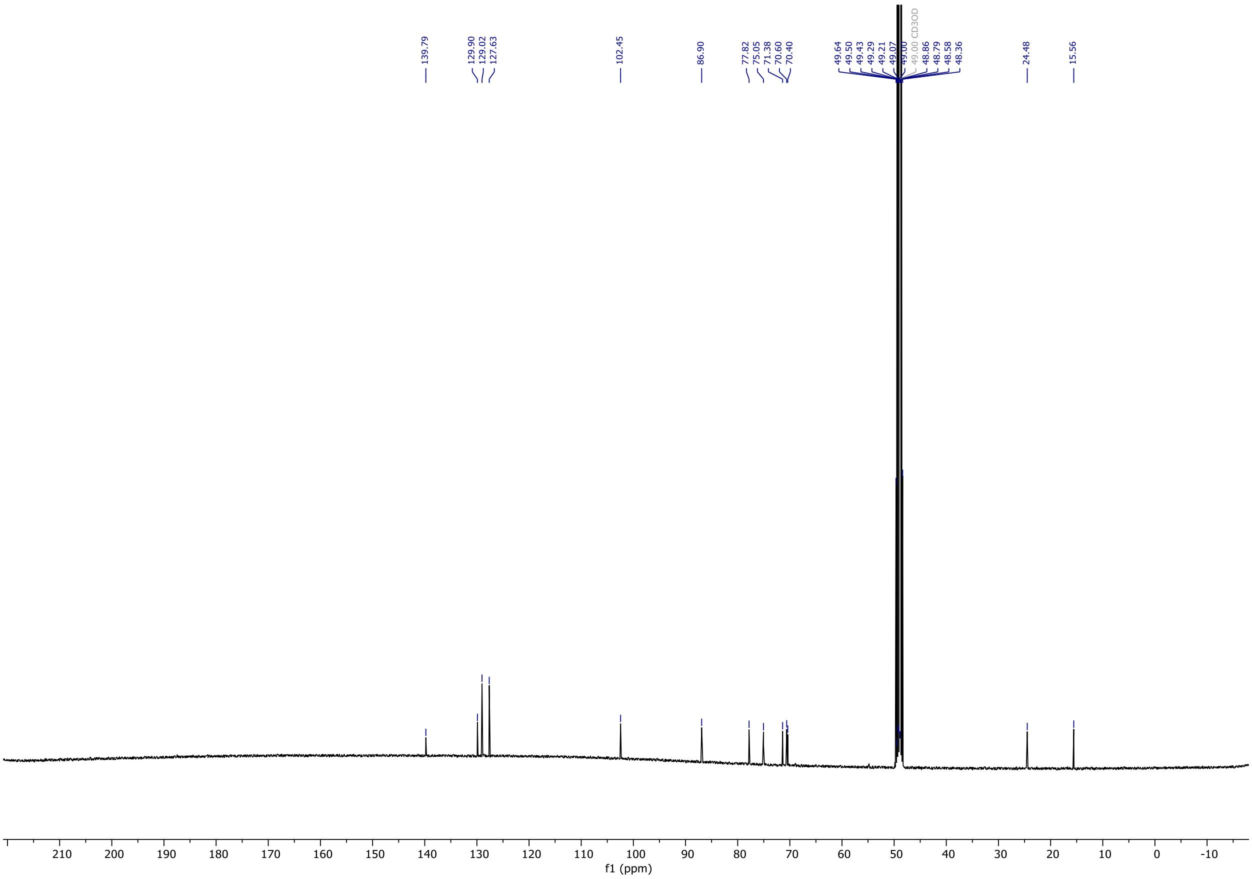

^{13}C NMR

Ethyl 2,3-di-*O*-acetyl-4,6-*O*-benzylidene-1-thio-β-D-galactoside (27)

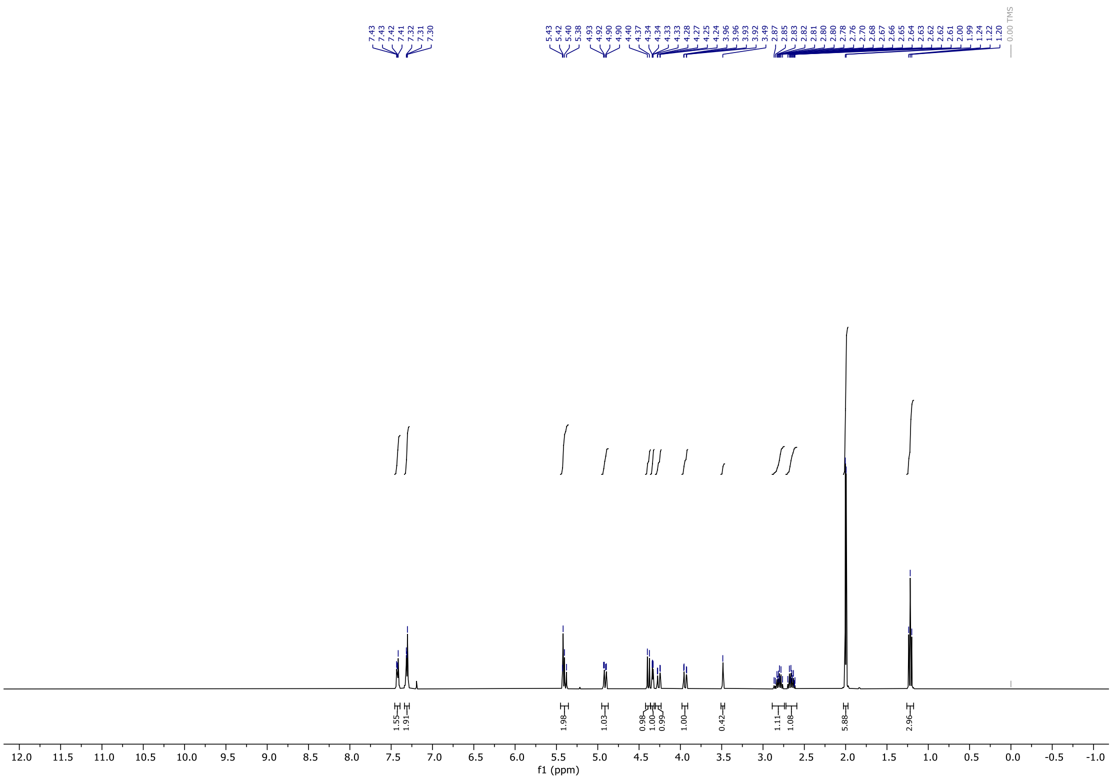

^{1}H NMR

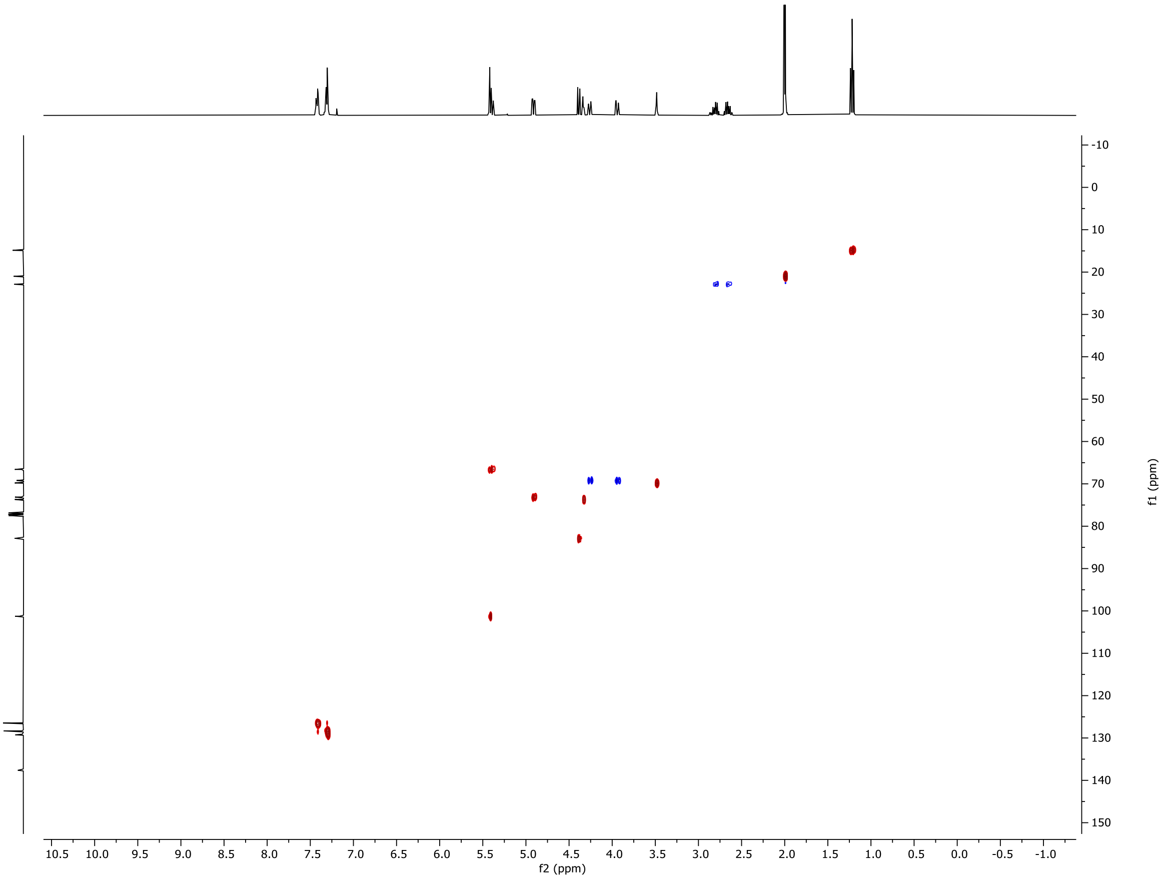

^{1}H-^{13}C HSQC

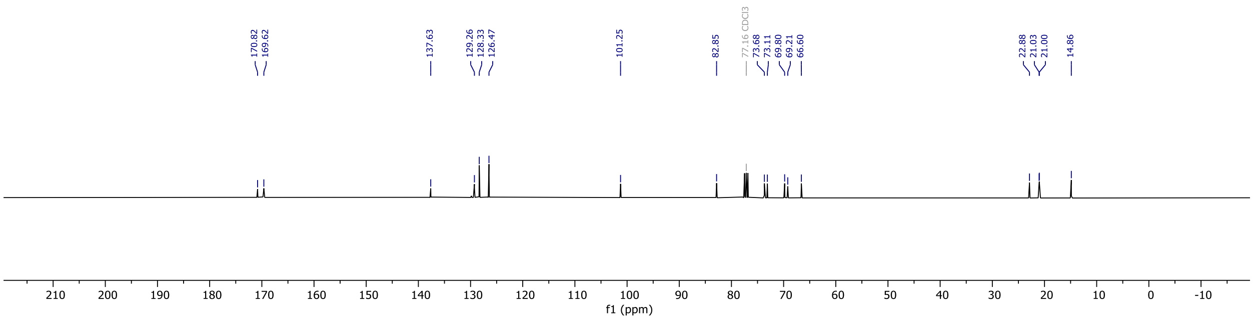
170.82
169.62
137.63
129.26
128.33
126.47
101.25
82.85
77.16 CDCl3
73.68
73.11
69.80
69.21
66.60
22.88
21.03
21.00
14.86
f1 (ppm)

^{13}C NMR

Methyl 4,6-*O*-benzylidene-β-D-galactoside (28)

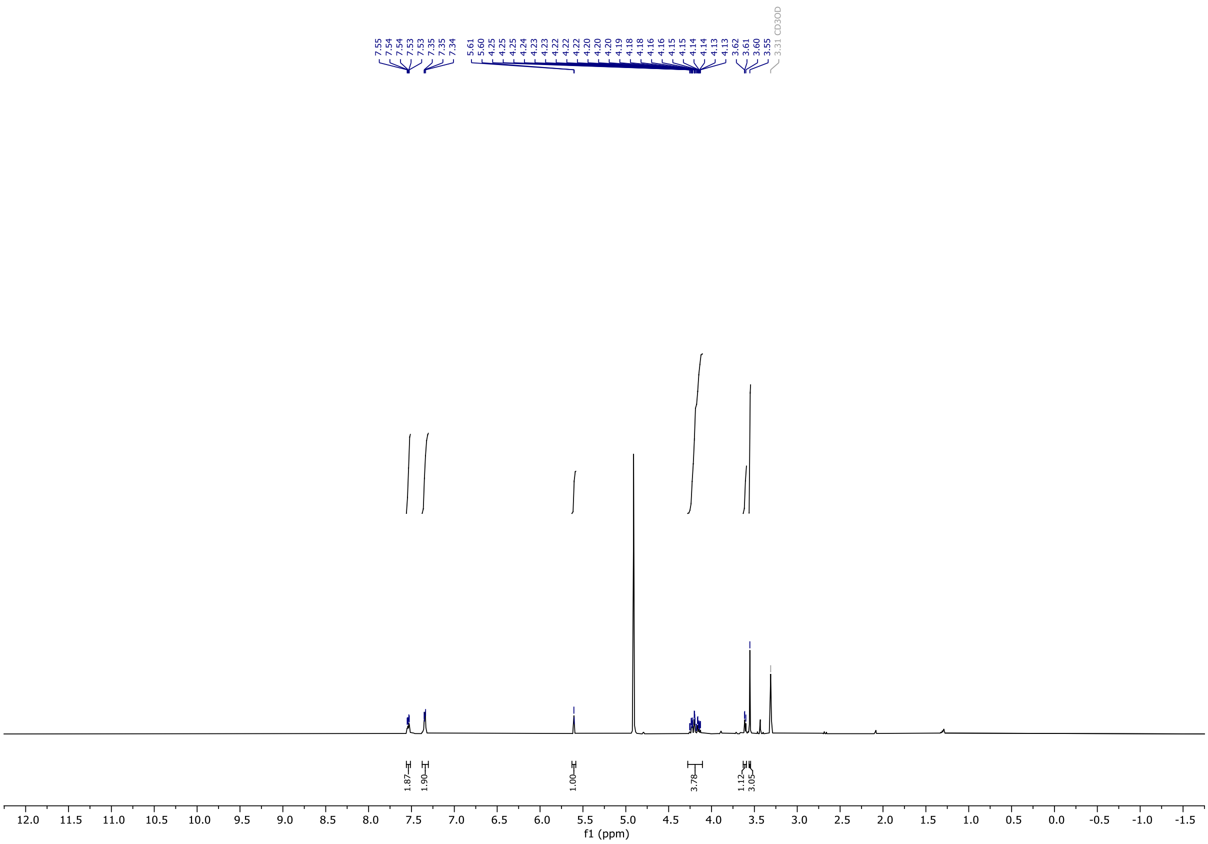

^{1}H NMR

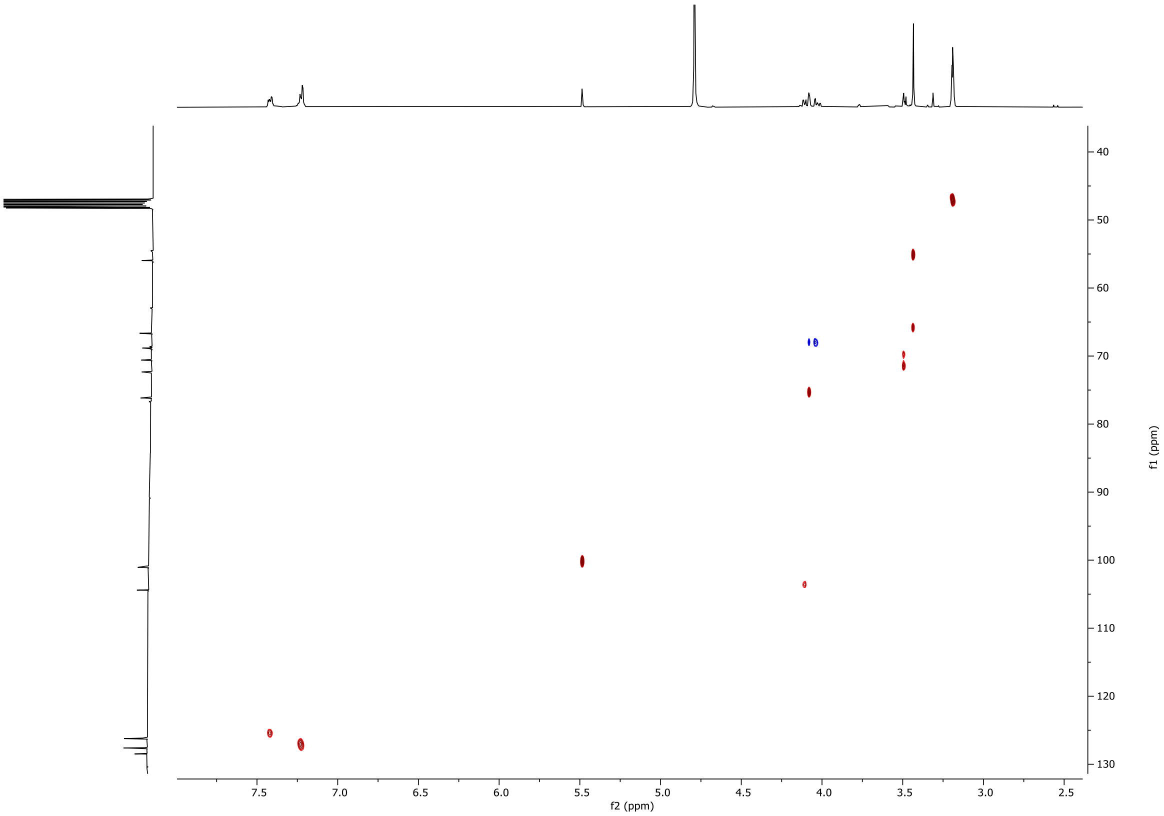

^{1}H-^{13}C HSQC NMR

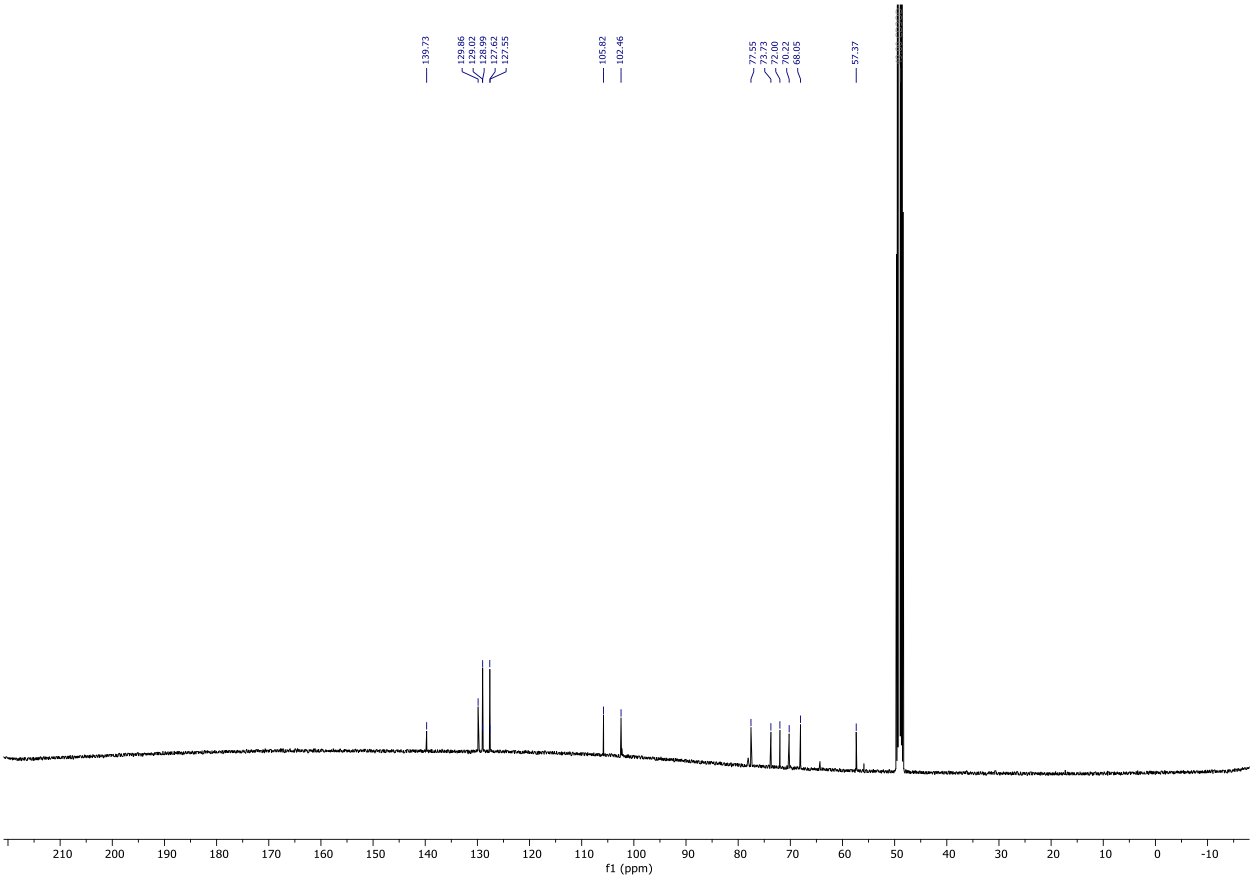
139.73
129.86
129.02
128.99
127.62
127.55
105.82
102.46
77.55
73.73
72.00
70.22
68.05
57.37
210 200 190 180 170 160 150 140 130 120 110 100 90 80 70 60 50 40 30 20 10 0 -10
f1 (ppm)

^{13}C NMR

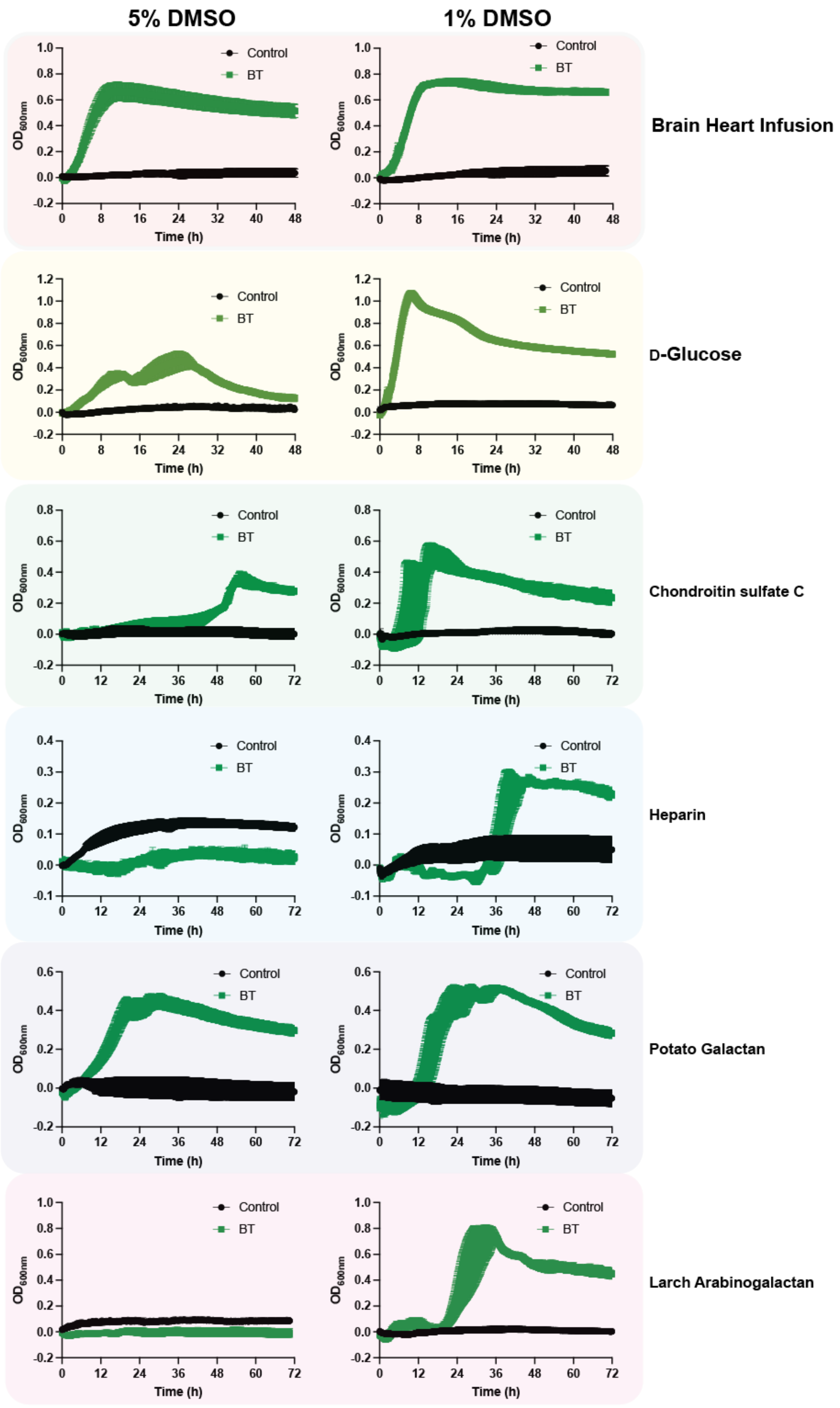

Supplementary figure 1. The effects of DMSO on the growth of *Bacteroides thetaiotaomicron* VPI-5482

B. thetaiotaomicron VPI-5482 was grown in rich media (BHI), or minimal media supplemented 5 mg/ml of an appropriate polysaccharide with either 5% or 1% DMSO. Data are technical triplicates with the standard error of the mean. Numbers indicate arylsulfamate compounds as in Figure 1

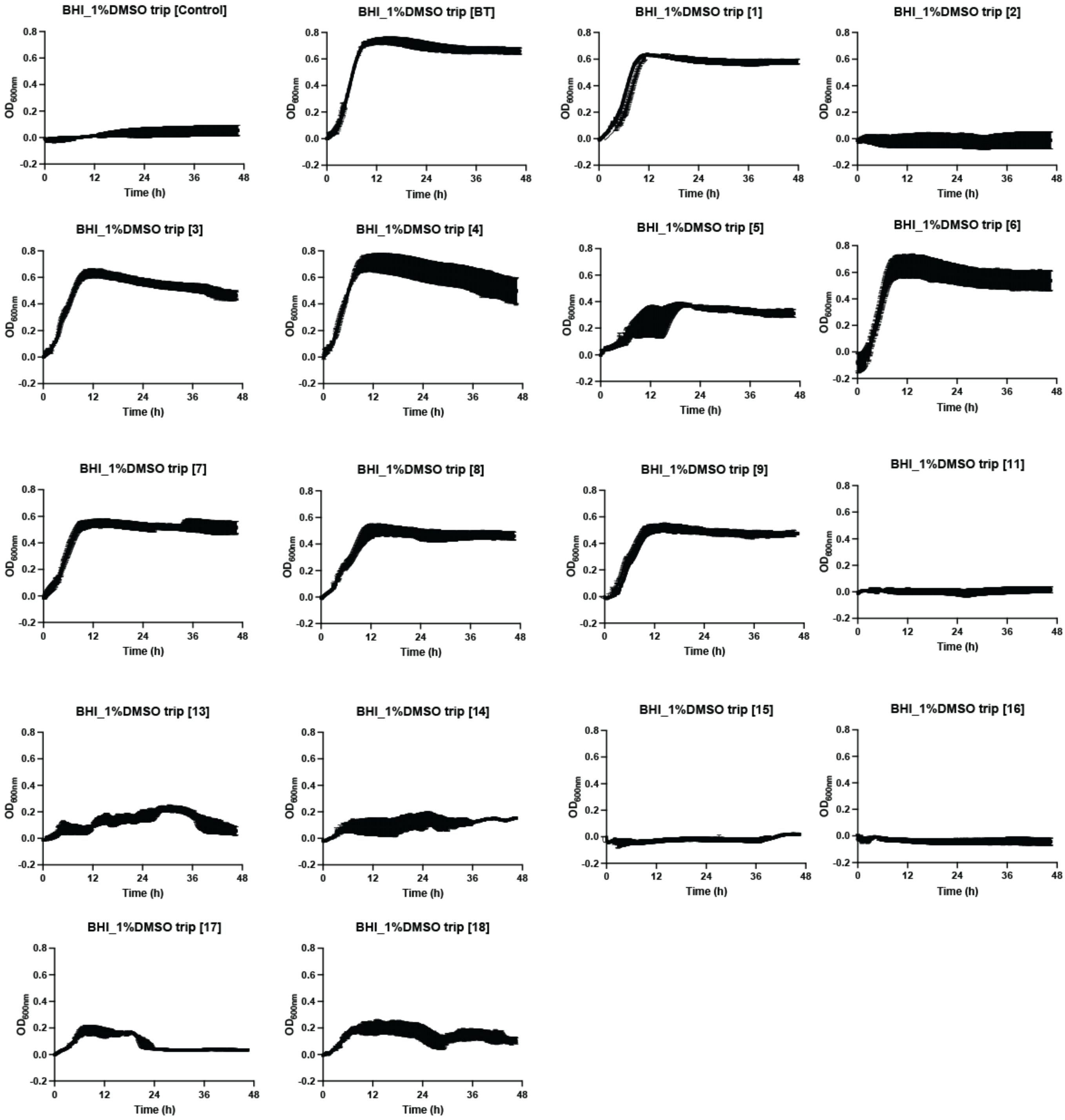

Supplementary figure 2. The effects of arylsulfamates on the growth of *Bacteroides thetaiotaomicron* VPI-5482 grown in BHI media

B. thetaiotaomicron VPI-5482 was grown in rich media (BHI), with 1% DMSO, and with or without 1 mM of the appropriate arylsulfamate. Data are technical triplicates with the standard error of the mean. Numbers indicate arylsulfamate compounds as in Figure 1.

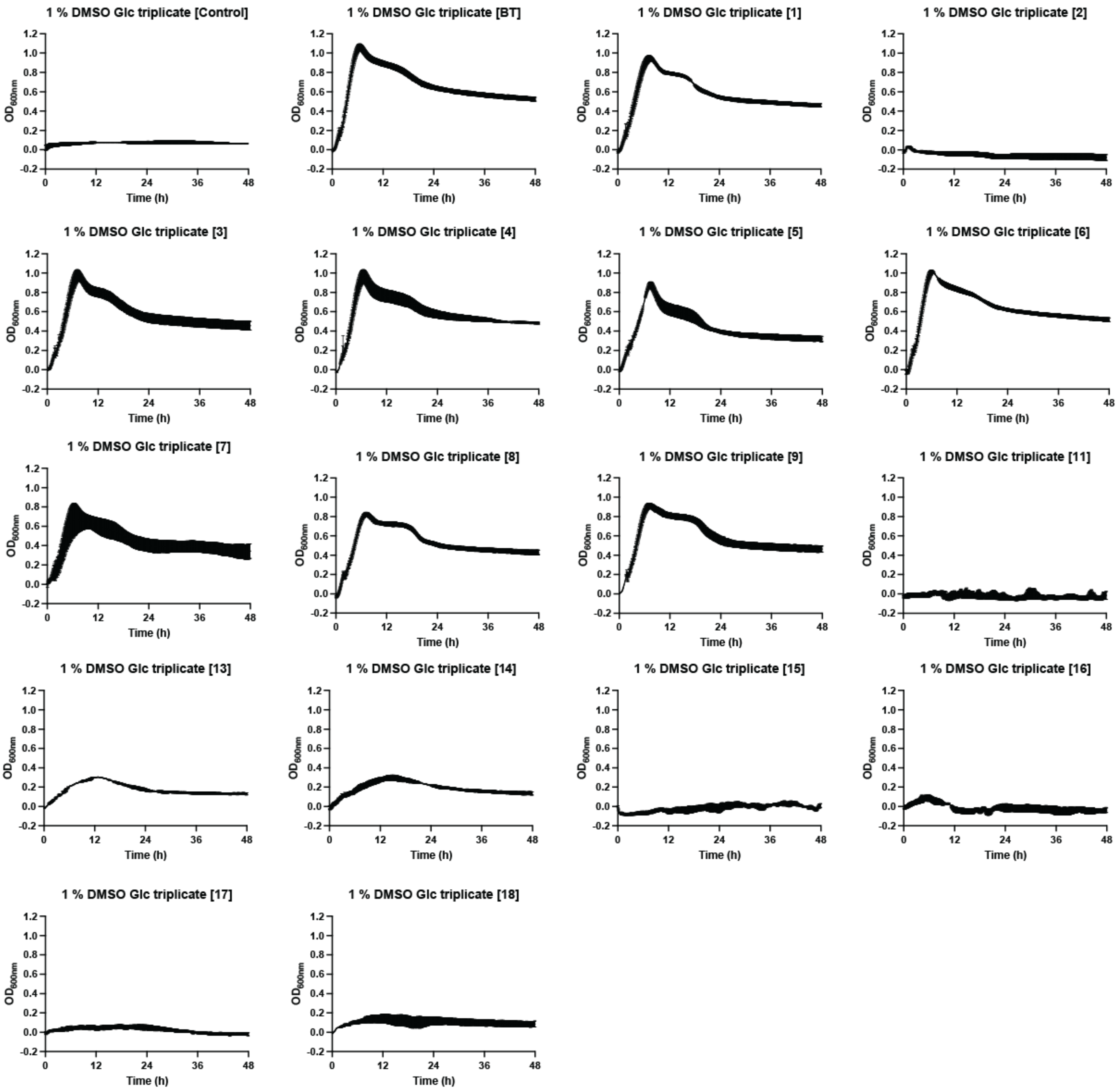

Supplementary figure 3. The effects of arylsulfamates on the growth of *Bacteroides thetaiotaomicron* VPI-5482 grown in minimal media with D-glucose

B. thetaiotaomicron VPI-5482 was grown in minimal media supplemented with 5 mg/ml D-glucose (Glc), 1% DMSO, and with or without 1 mM of the appropriate arylsulfamate. Data are technical triplicates with the standard error of the mean. Numbers indicate arylsulfamate compounds as in Figure 1.

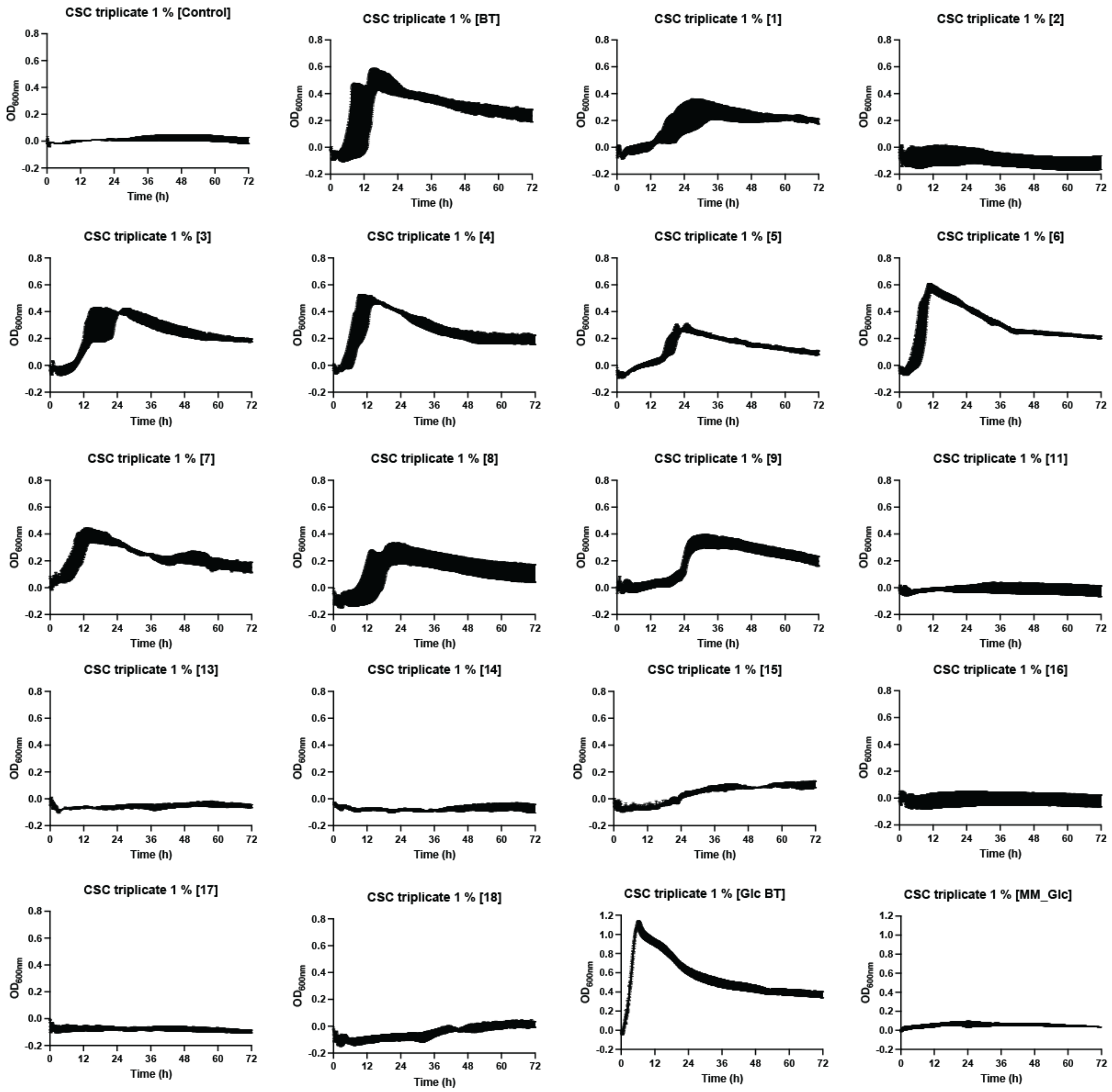

Supplementary figure 4. The effects of arylsulfamates on the growth of *Bacteroides thetaiotaomicron* VPI-5482 grown in minimal media with Chondroitin sulfate C (CSC)

B. thetaiotaomicron VPI-5482 was grown in minimal media supplemented with 5 mg/ml Chondroitin sulfate C (CSC), 1% DMSO, and with or without 1 mM of the appropriate arylsulfamate. Data are technical triplicates with the standard error of the mean. Numbers indicate arylsulfamate compounds as in Figure 1.

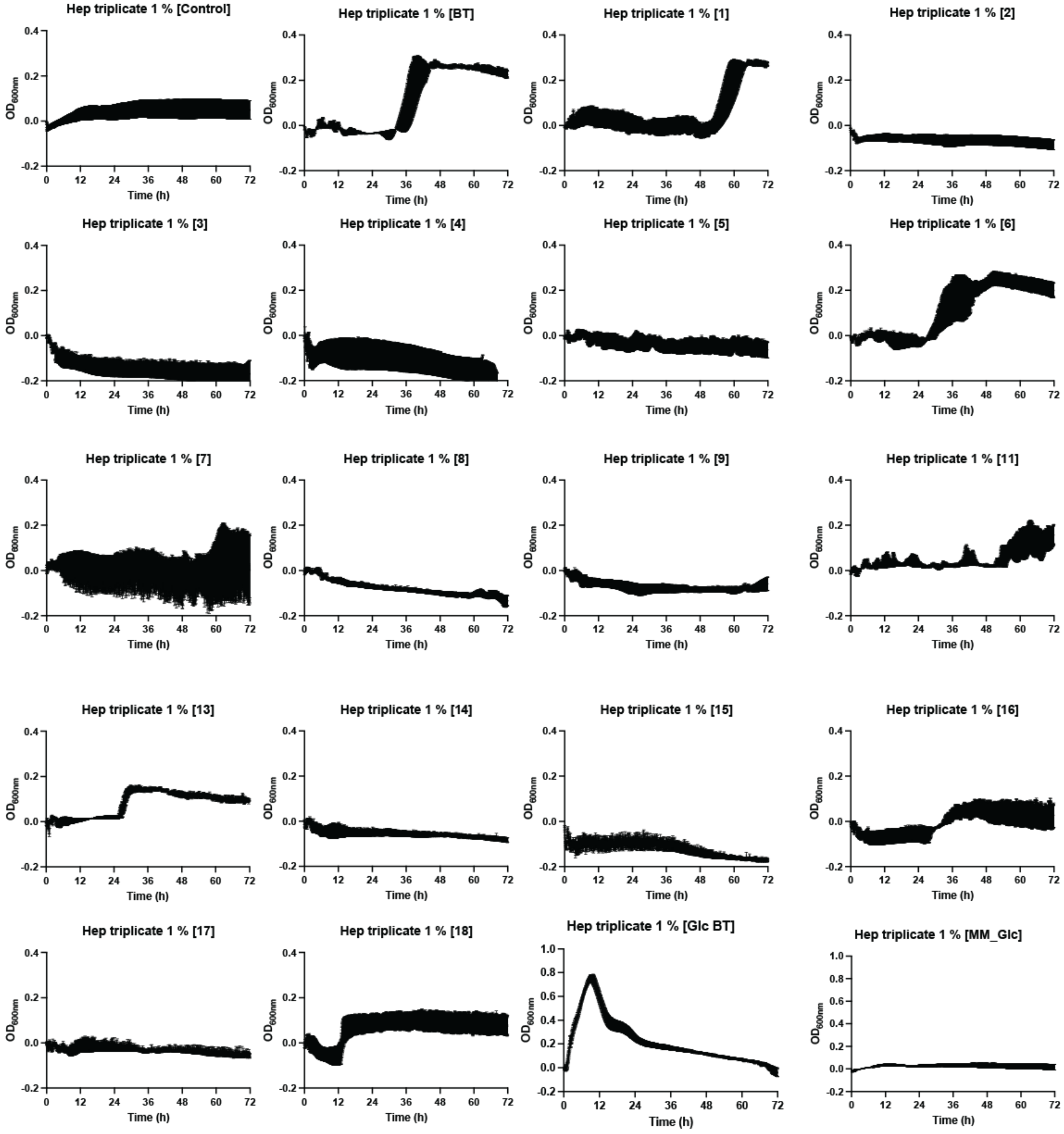

Supplementary figure 5. The effects of arylsulfamates on the growth of *Bacteroides thetaiotaomicron* VPI-5482 grown in minimal media with Heparin

B. thetaiotaomicron VPI-5482 was grown in minimal media supplemented with 5 mg/ml Heparin (Hep), 1% DMSO, and with or without 1 mM of the appropriate arylsulfamate. Data are technical triplicates with the standard error of the mean. Numbers indicate arylsulfamate compounds as in Figure 1.

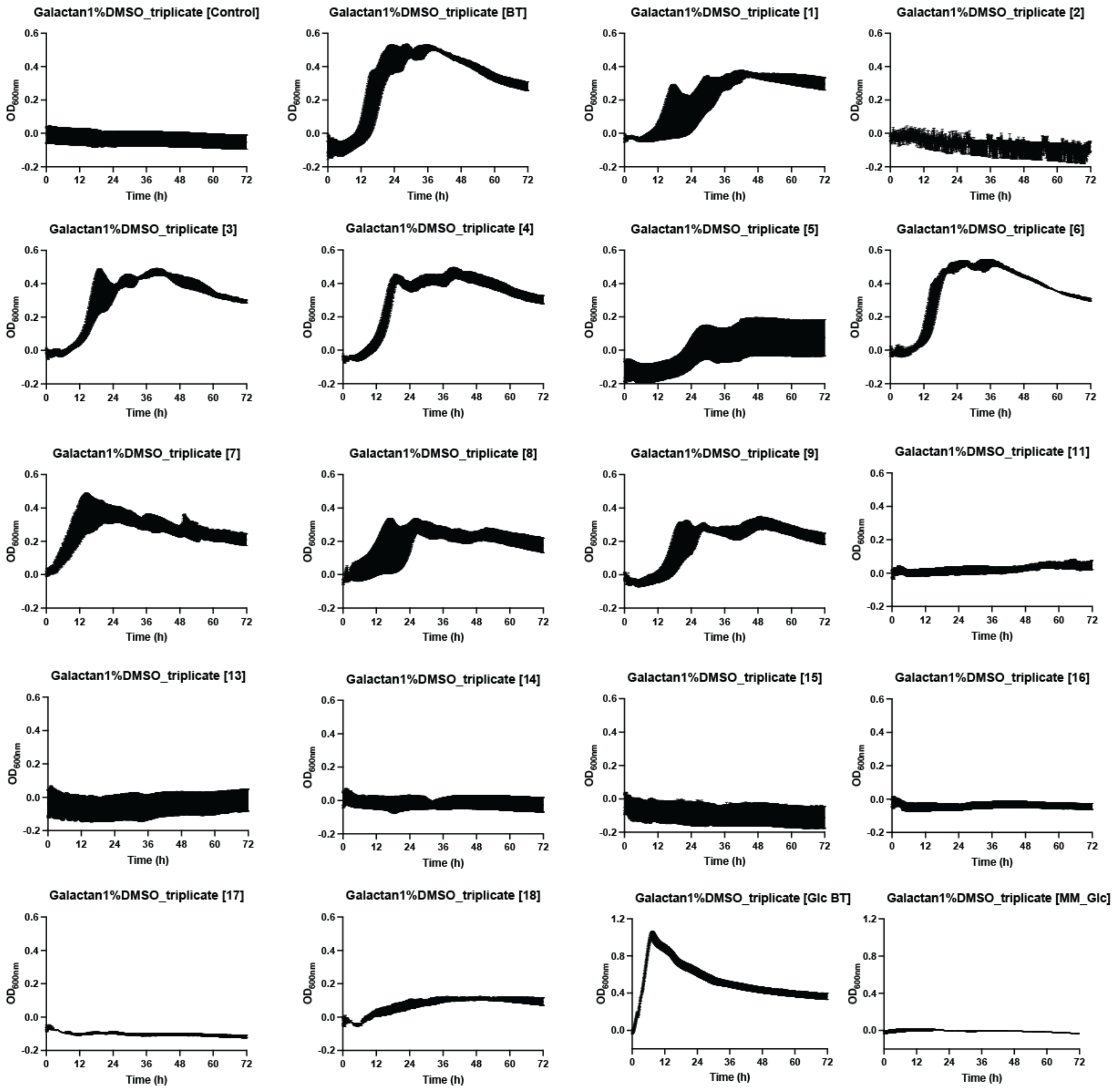

Supplementary figure 6. The effects of arylsulfamates on the growth of *Bacteroides thetaiotaomicron* VPI-5482 grown in minimal media with Potato galactan

B. thetaiotaomicron VPI-5482 was grown in minimal media supplemented with 5 mg/ml Potato galactan (PG), 1% DMSO, and with or without 1 mM of the appropriate arylsulfamate. Data are technical triplicates with the standard error of the mean. Numbers indicate arylsulfamate compounds as in Figure 1.

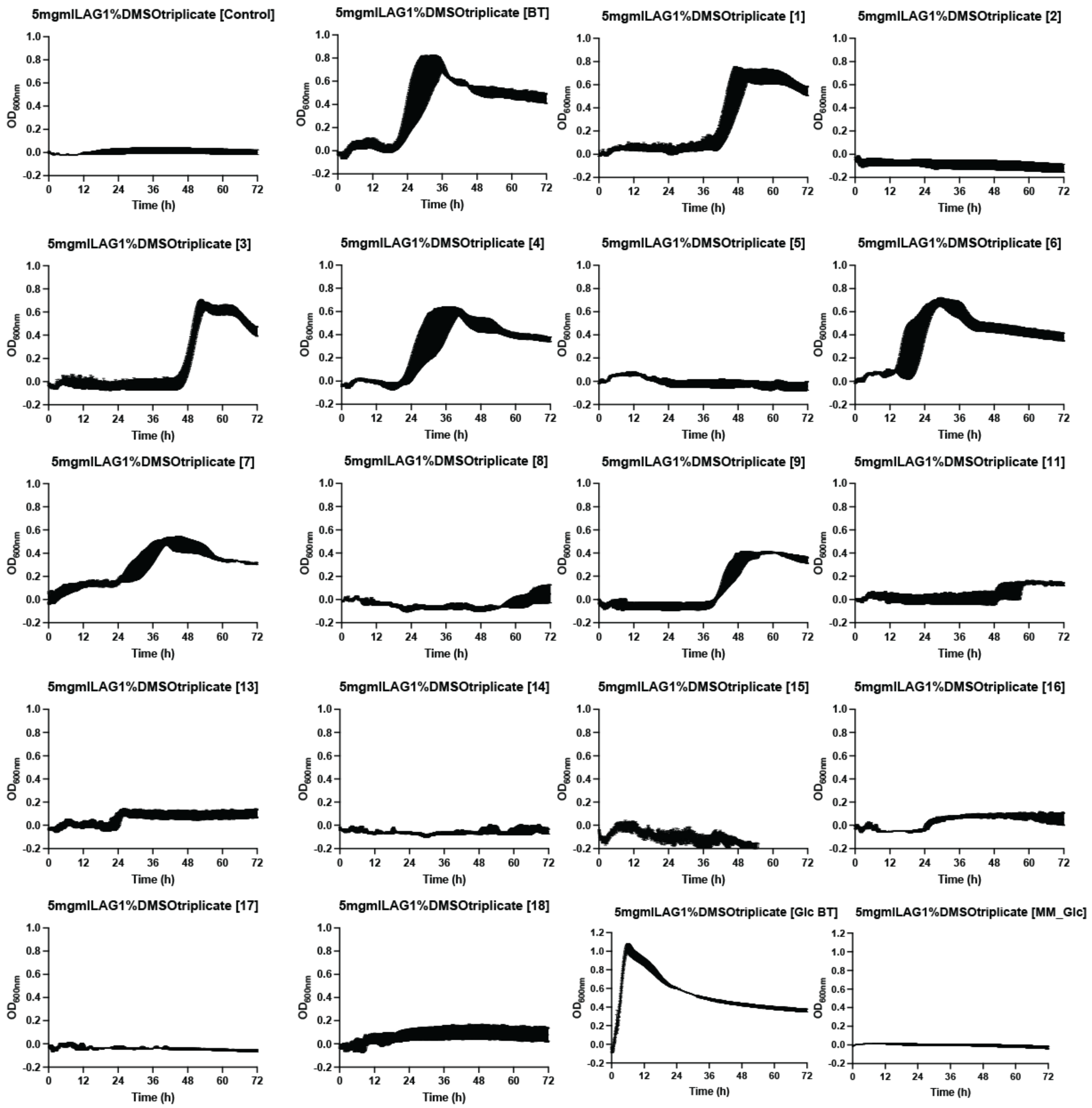

Supplementary figure 7. The effects of arylsulfamates on the growth of *Bacteroides thetaiotaomicron* VPI-5482 grown in minimal media with Larch arabinogalactan

B. thetaiotaomicron VPI-5482 was grown in minimal media supplemented with 5 mg/ml Larch arabinogalactan (LAG), 1% DMSO, and with or without 1 mM of the appropriate arylsulfamate. Data are technical triplicates with the standard error of the mean. Numbers indicate arylsulfamate compounds as in Figure 1.

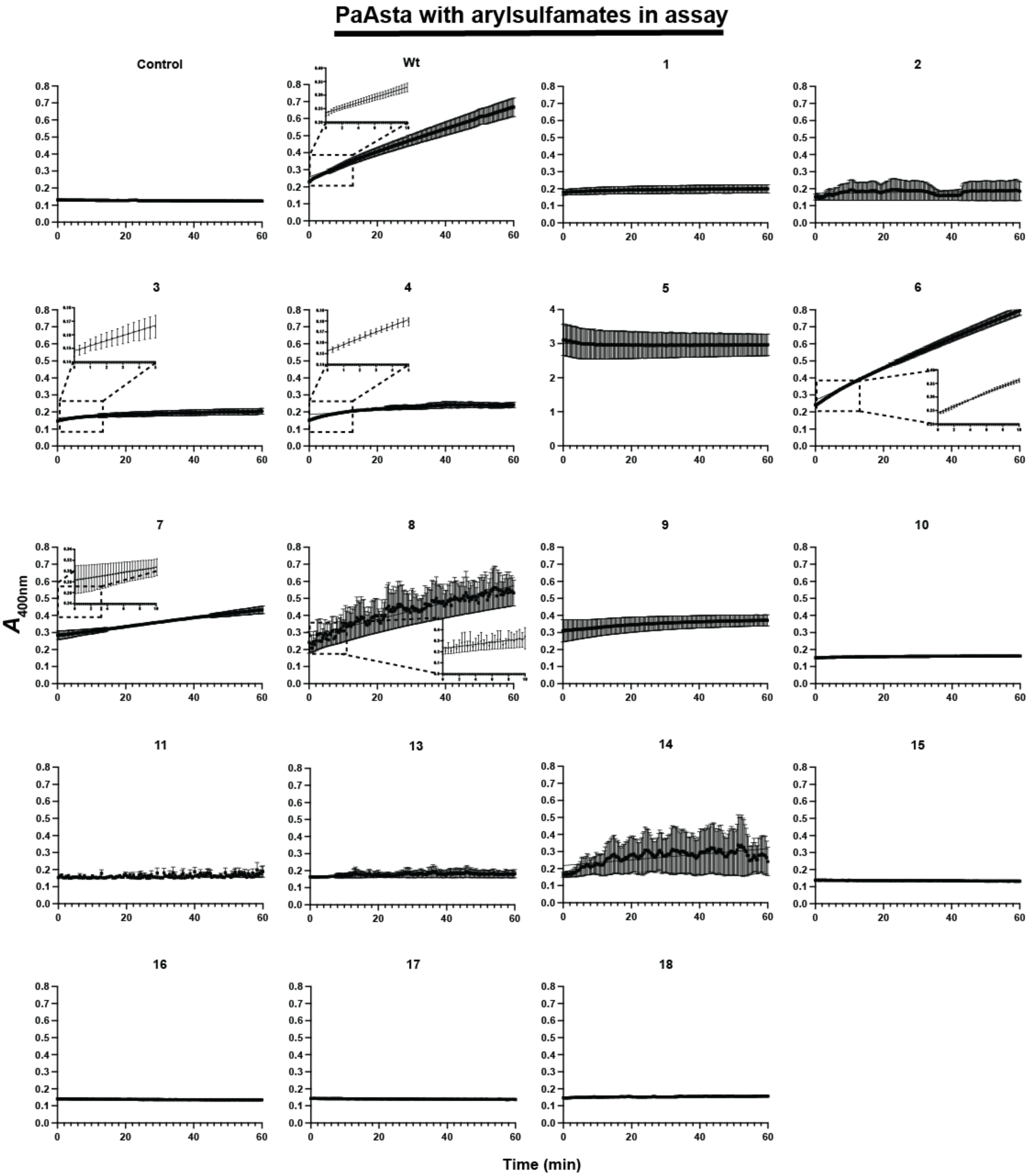

Supplementary figure 8. Kinetic curves of *Pa*Asta assayed against *para*-nitrophenol sulfate with and without arylsulfamate inhibitors

*Pa*Asta, at a concentration of 25 nM, was assayed against 1 mM *para*-nitrophenol with and without 1 mM of various arylsulfamate inhibitors included in the assay. The assay was performed in 100 mM of Bis-Tris-propane pH 7.0 with 5% DMSO, 150 mM NaCl, 0.02% (v/v) Brij-35 and 5 mM $CaCl_2$. Assays were performed in triplicate. Numbers indicate arylsulfamate compounds as in Figure 1.

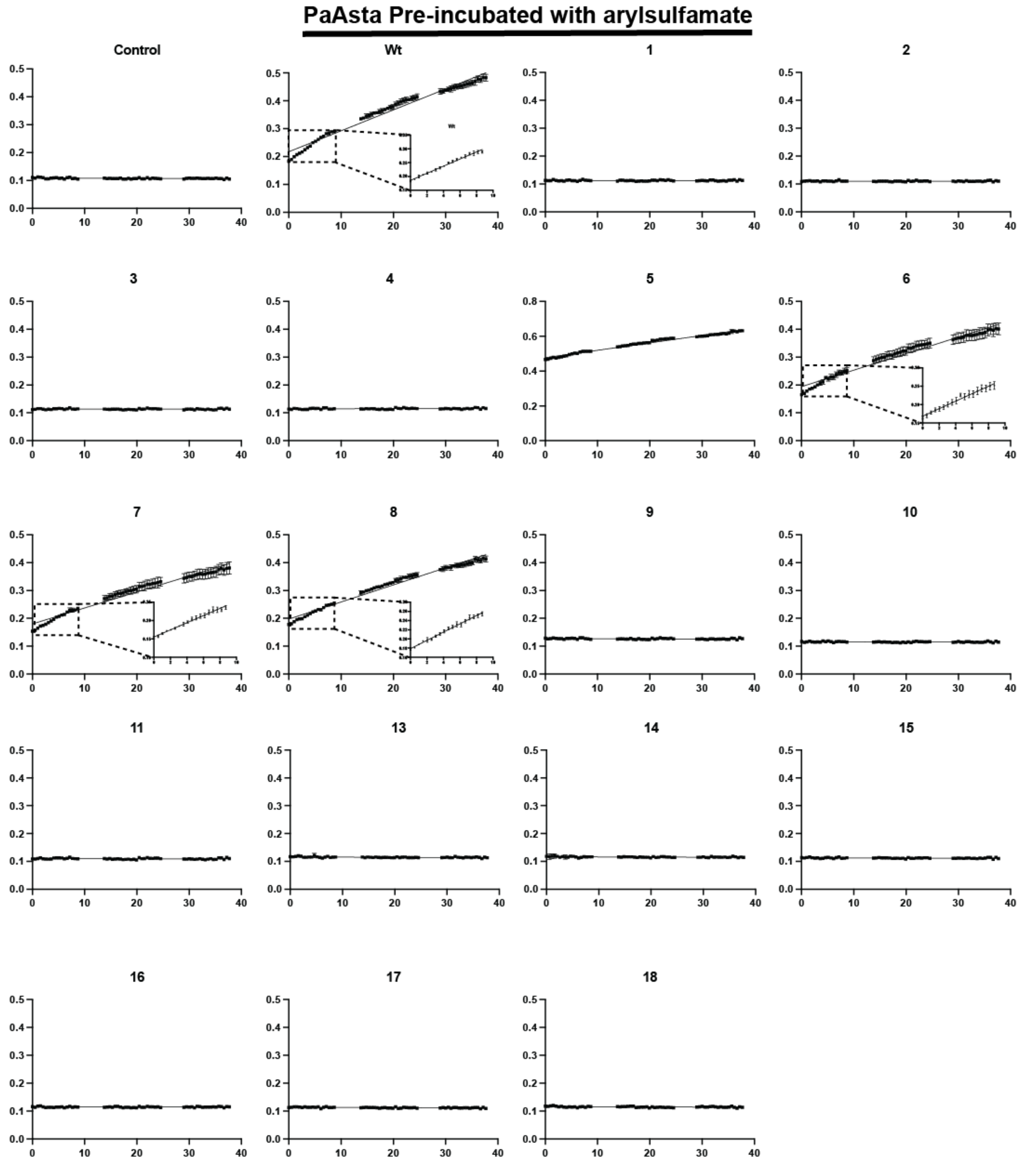

Supplementary figure 9. Kinetic curves of *Pa*Asta assayed against *para*-nitrophenol sulfate after pre-incubation of the enzyme with arylsulfamate inhibitors

*Pa*Asta, which had been incubated for ~24h with 1 mM of the appropriate arylsulfamate inhibitor, was assayed using a concentration of 25 nM against 1 mM para-nitrophenol. The assay was performed in 100 mM of Bis-Tris-propane pH 7.0 with 5% DMSO, 150 mM NaCl, 0.02% (v/v) Brij-35 and 5 mM $CaCl_2$. Assays were performed in triplicate. Numbers indicate arylsulfamate compounds as in Figure 1.

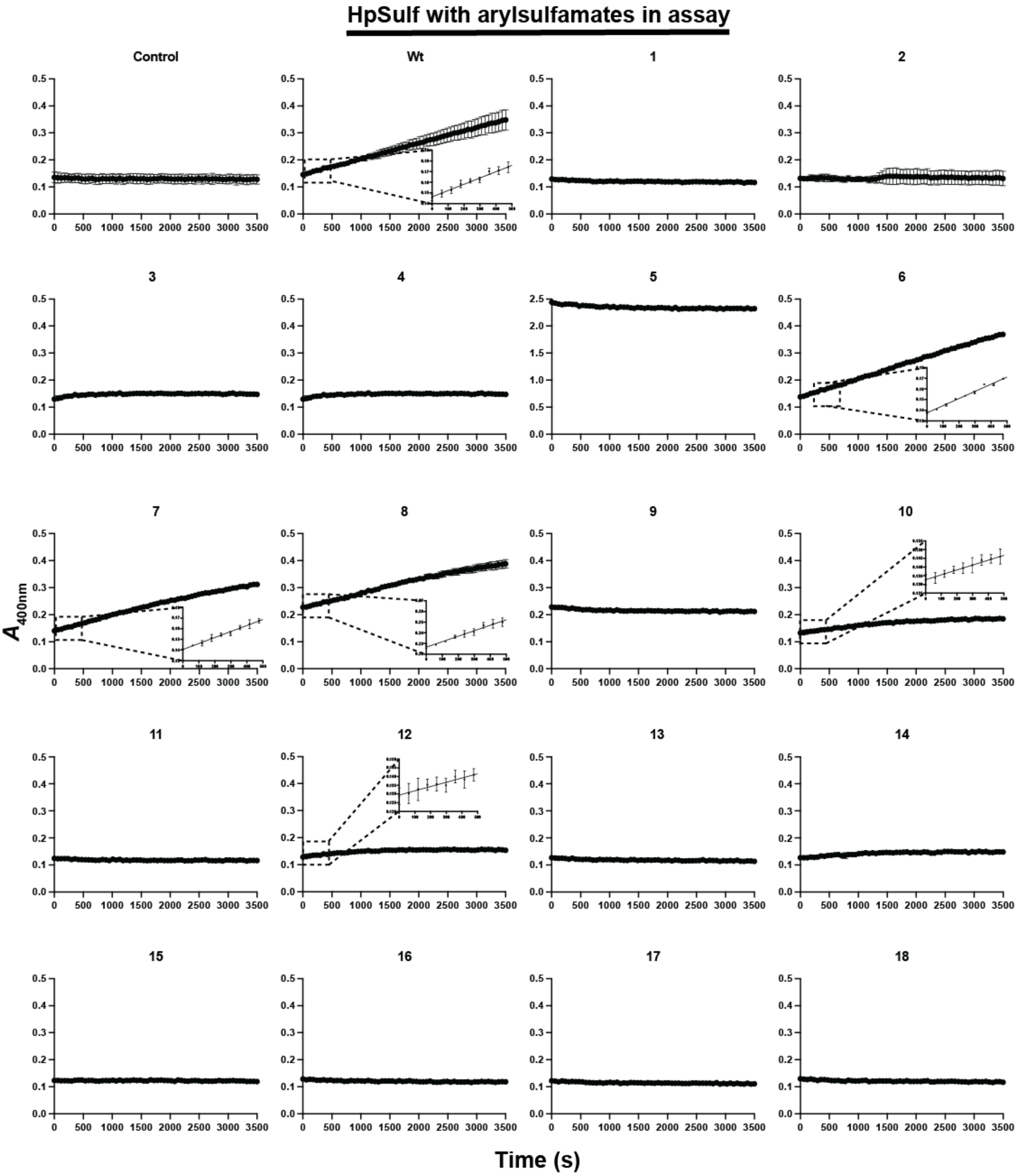

Supplementary figure 10. Kinetic curves of *Hp*Sulf assayed against para-nitrophenol sulfate with and without arylsulfamate inhibitors

*Hp*Sulf, at a concentration of 5 μg/ml, was assayed against 1 mM para-nitrophenol with and without 1 mM of various arylsulfamate inhibitors included in the assay. The assay was performed in 100 mM of Bis-Tris-propane pH 7.0 with 5% DMSO, 150 mM NaCl, 0.02% (v/v) Brij-35 and 5 mM $CaCl_2$. Assays were performed in triplicate. Numbers indicate arylsulfamate compounds as in Figure 1.

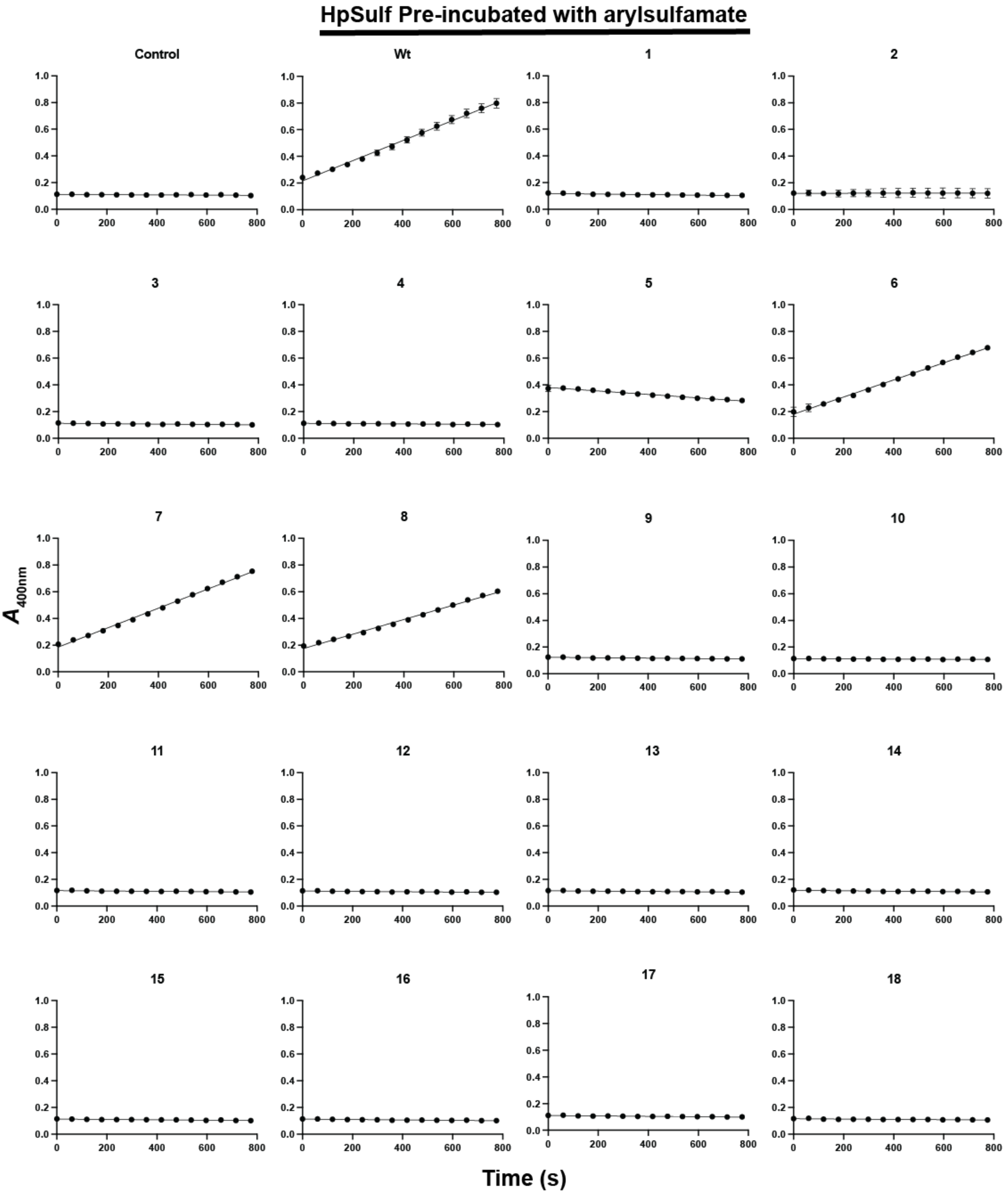

Supplementary figure 11. Kinetic curves of *Hp*Sulf assayed against para-nitrophenol sulfate after pre-incubation of the enzyme with arylsulfamate inhibitors

*Hp*Sulf, which had been incubated for ~24h with 1 mM of the appropriate arylsulfamate inhibitor, was assayed using a concentration of 50 μg/ml against 1 mM para-nitrophenol. The assay was performed in 100 mM of Bis-Tris-propane pH 7.0 with 5% DMSO, 150 mM NaCl, 0.02% (v/v) Brij-35 and 5 mM $CaCl_2$. Assays were performed in triplicate. Numbers indicate arylsulfamate compounds as in Figure 1.

BT3177$^{6S\text{-}GlcNAc}$ with arylsulfamate in assay

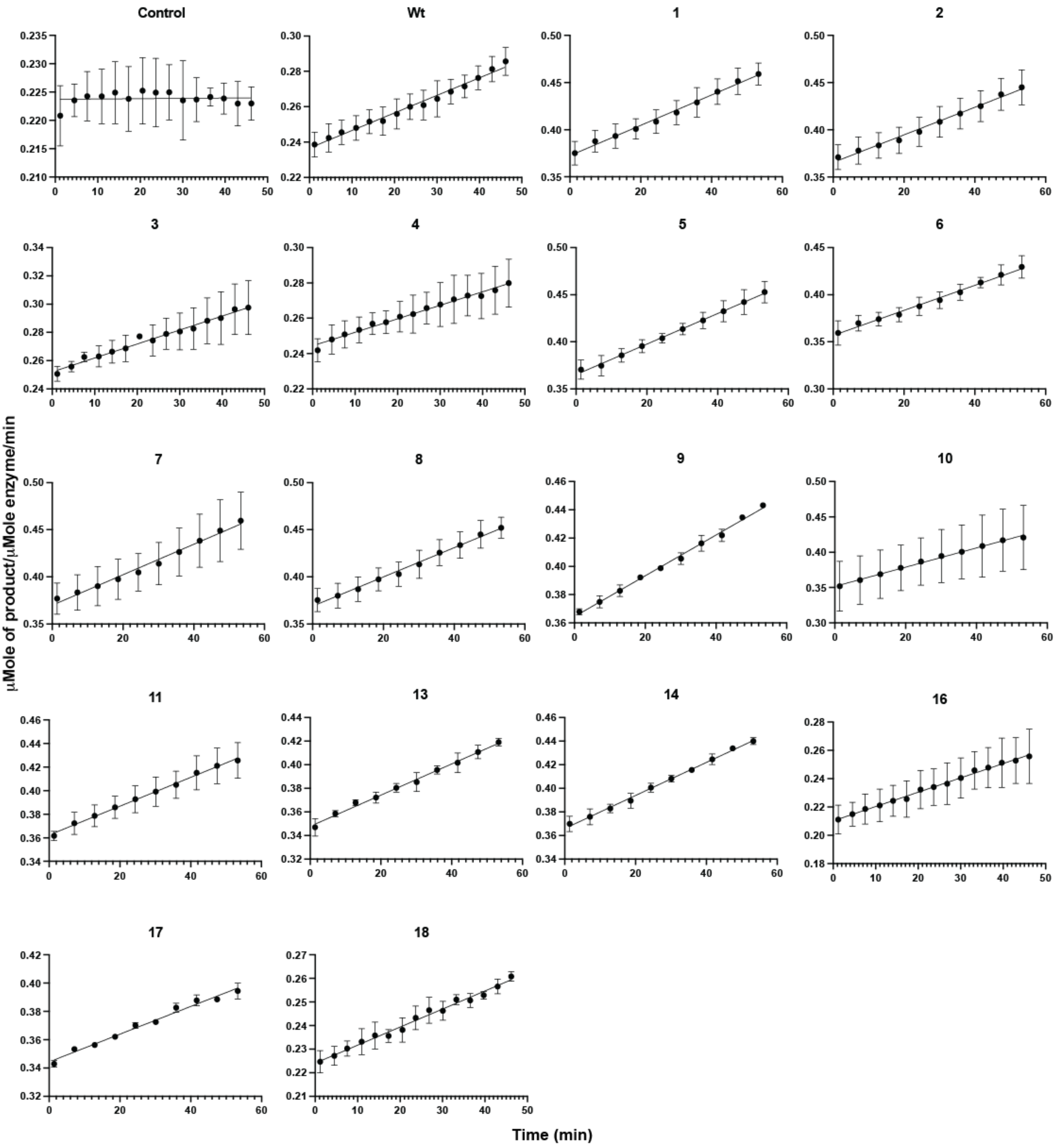

Supplementary figure 12. Kinetic curves of BT3177$^{6S\text{-}GlcNAc}$ assayed against BODIPY labelled 6S-*N*-acetylglucosamine with and without arylsulfamate inhibitors

BT3177$^{6S\text{-}GlcNAc}$, at a concentration of 446.5 nM, was assayed against 1 µM BODIPY labelled 6S-*N*-acetylglucosamine with and without 1 mM of various arylsulfamate inhibitors included in the assay. The assay was performed in 100 mM of Bis-Tris-propane pH 8.0 with 5% DMSO, 150 mM NaCl, 0.02% (v/v) Brij-35 and 5 mM $CaCl_2$. Assays were performed in triplicate. Numbers indicate arylsulfamate compounds as in Figure 1.

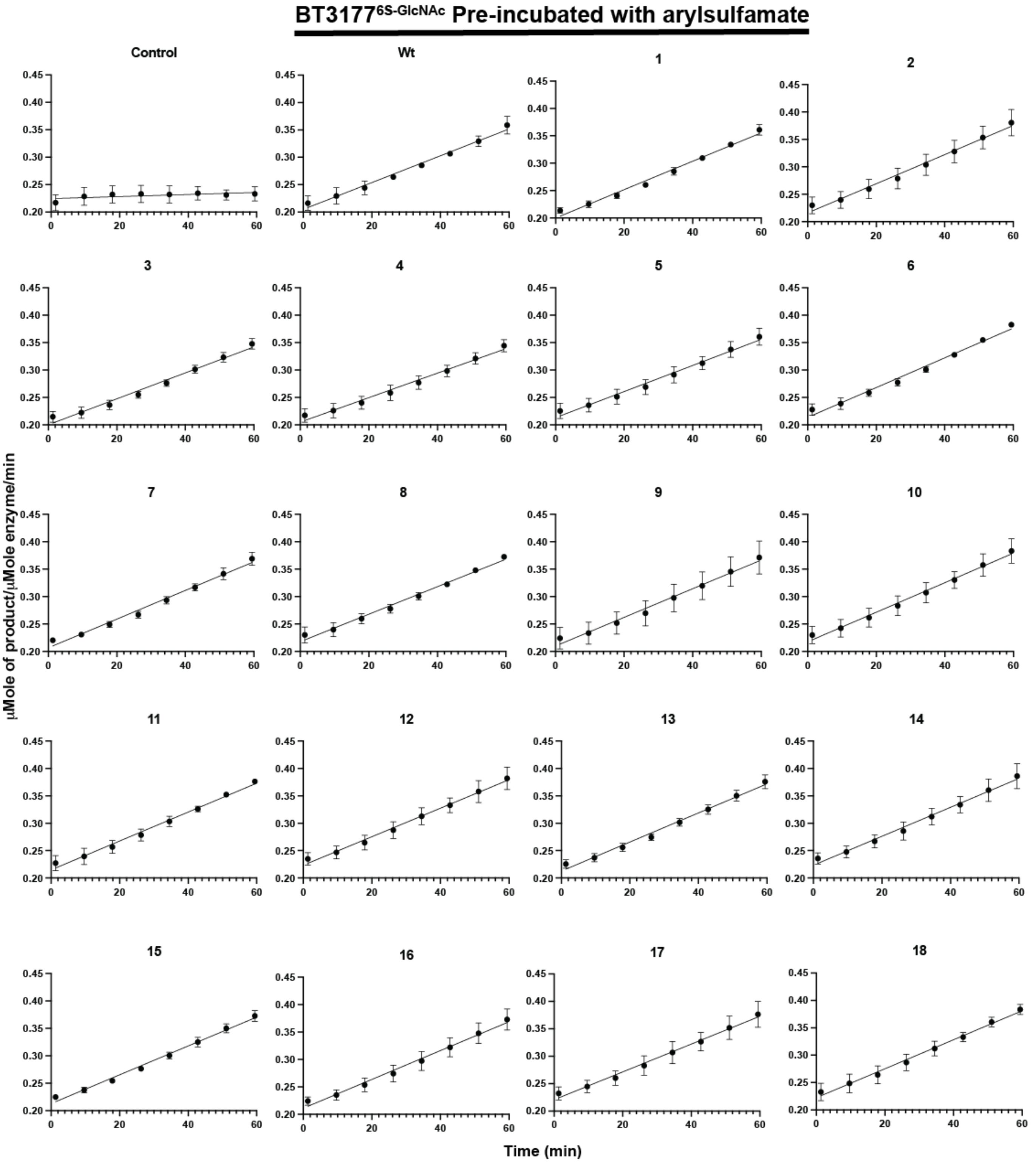

Supplementary figure 13. Kinetic curves of BT3177$^{6S-GlcNAc}$ assayed against BODIPY labelled 6S-*N*-acetylglucosamine after pre-incubation of the enzyme with arylsulfamate inhibitors

BT3177$^{6S-GlcNAc}$, which had been incubated for ~24h with 1 mM of the appropriate arylsulfamate inhibitor, was assayed using a concentration of 470 nM against 1 μM BODIPY labelled 6S-*N*-acetylglucosamine. The assay was performed in 100 mM of Bis-Tris-propane pH 8.0 with 5% DMSO, 150 mM NaCl, 0.02% (v/v) Brij-35 and 5 mM $CaCl_2$. Assays were performed in triplicate. Numbers indicate arylsulfamate compounds as in Figure 1.

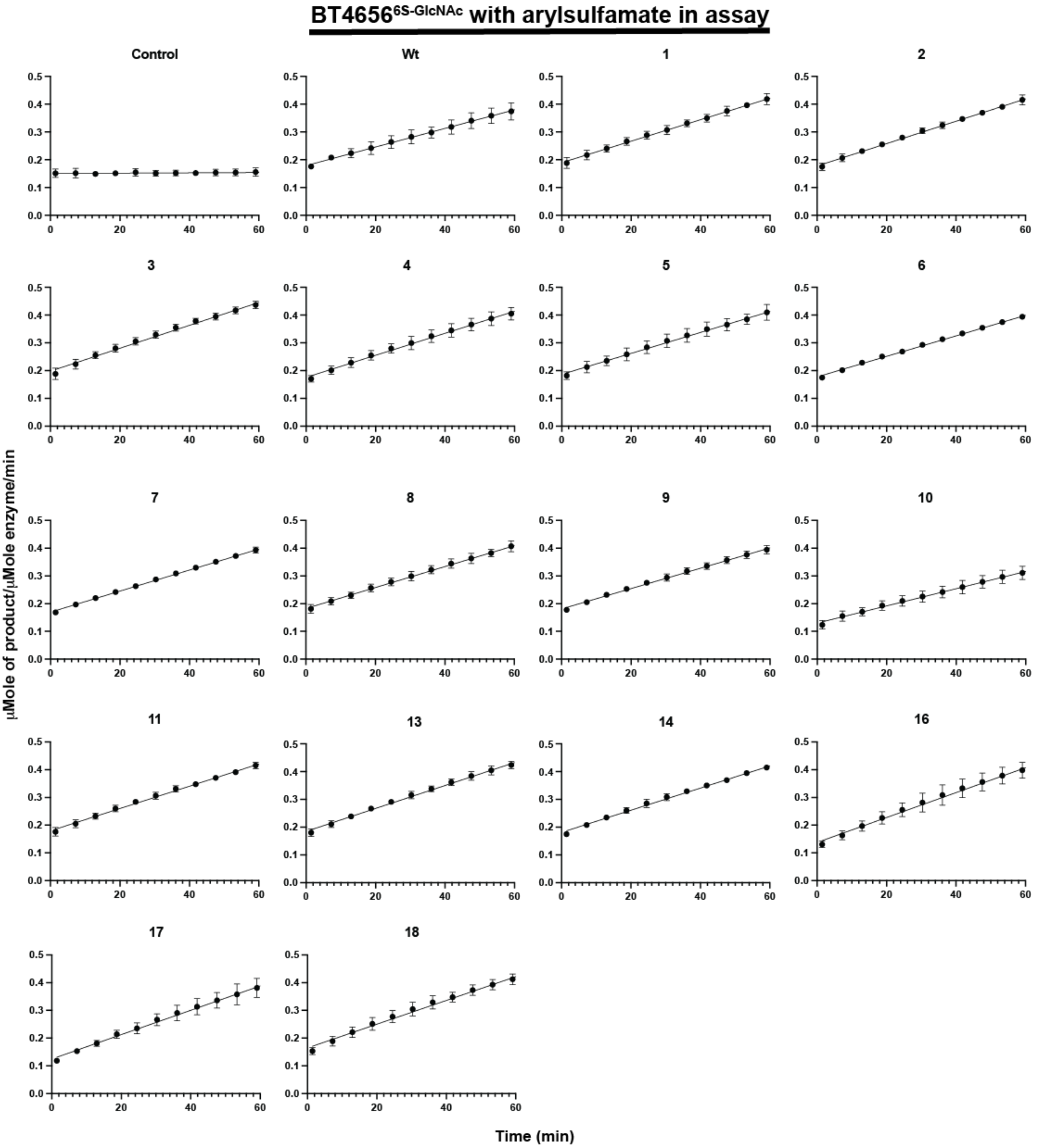

Supplementary figure 14. Kinetic curves of BT4656$^{6S\text{-}GlcNAc}$ assayed against BODIPY labelled 6S-*N*-acetylglucosamine with and without arylsulfamate inhibitors

BT4656$^{6S\text{-}GlcNAc}$, at a concentration of 200 nM, was assayed against 1 µM BODIPY labelled 6S-*N*-acetylglucosamine with and without 1 mM of various arylsulfamate inhibitors included in the assay. The assay was performed in 100 mM of MES pH 6.0 with 5% DMSO, 150 mM NaCl, 0.02% (v/v) Brij-35 and 5 mM $CaCl_2$. Assays were performed in triplicate. Numbers indicate arylsulfamate compounds as in Figure 1.

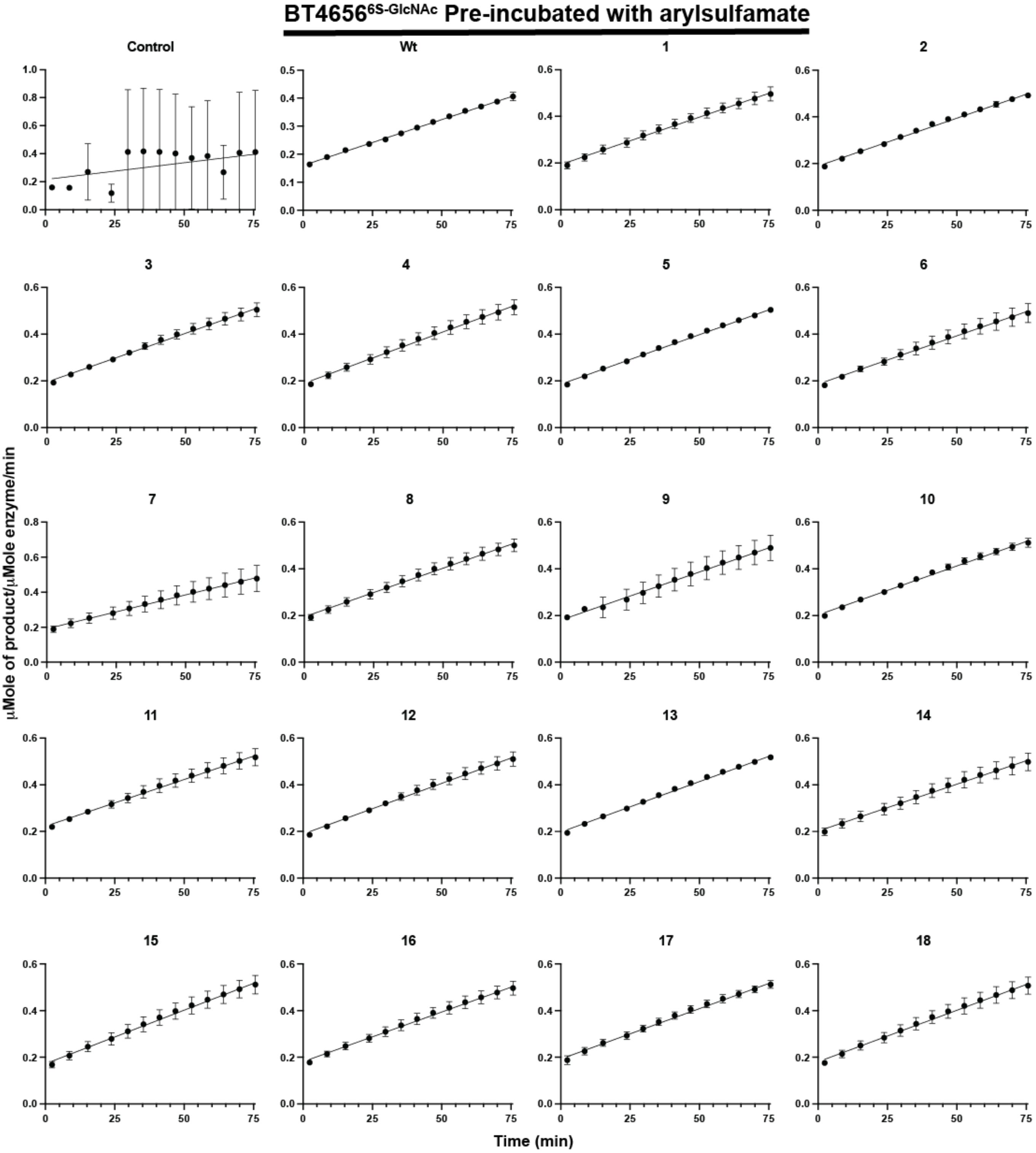

Supplementary figure 15. Kinetic curves of BT4656$^{6S\text{-}GlcNAc}$ assayed against BODIPY labelled 6S-*N*-acetylglucosamine after pre-incubation of the enzyme with arylsulfamate inhibitors

BT4656$^{6S\text{-}GlcNAc}$, which had been incubated for ~24h with 1 mM of the appropriate arylsulfamate inhibitor, was assayed using a concentration of 200 nM against 1 μM BODIPY labelled 6S-*N*-acetylglucosamine. The assay was performed in MES pH 6.0 with 5% DMSO, 150 mM NaCl, 0.02% (v/v) Brij-35 and 5 mM $CaCl_2$. Assays were performed in triplicate. Numbers indicate arylsulfamate compounds as in Figure 1.

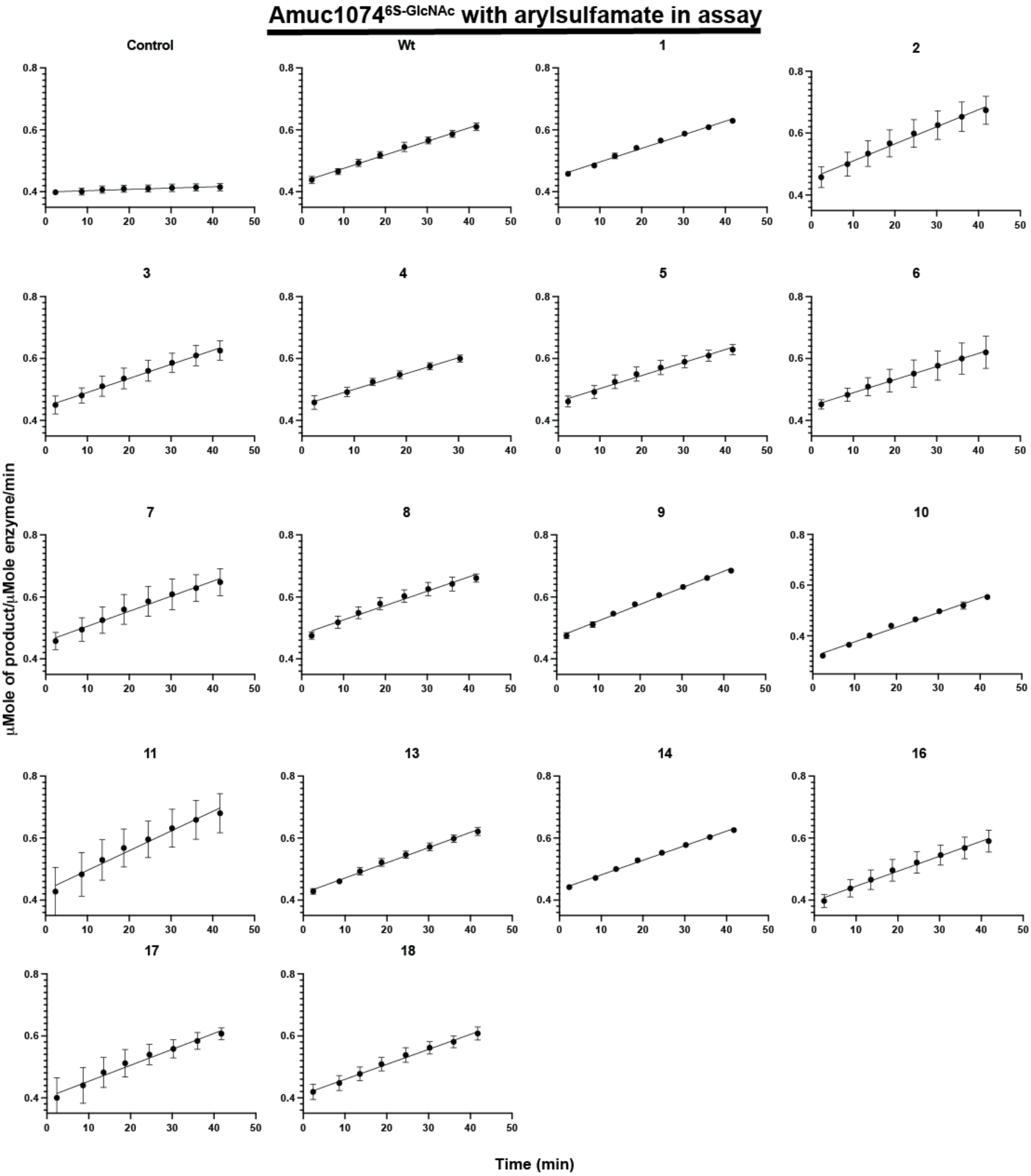

Supplementary figure 16. Kinetic curves of Amuc1074$^{6S\text{-}GlcNAc}$ assayed against BODIPY labelled 6S-*N*-acetylglucosamine with and without arylsulfamate inhibitors

Amuc1074$^{6S\text{-}GlcNAc}$, at a concentration of 6.4 µM, was assayed against 1 µM BODIPY labelled 6S-*N*-acetylglucosamine with and without 1 mM of various arylsulfamate inhibitors included in the assay. The assay was performed in 100 mM of MES pH 6.0 with 5% DMSO, 150 mM NaCl, 0.02% (v/v) Brij-35 and 5 mM $CaCl_2$. Assays were performed in triplicate. Numbers indicate arylsulfamate compounds as in Figure 1.

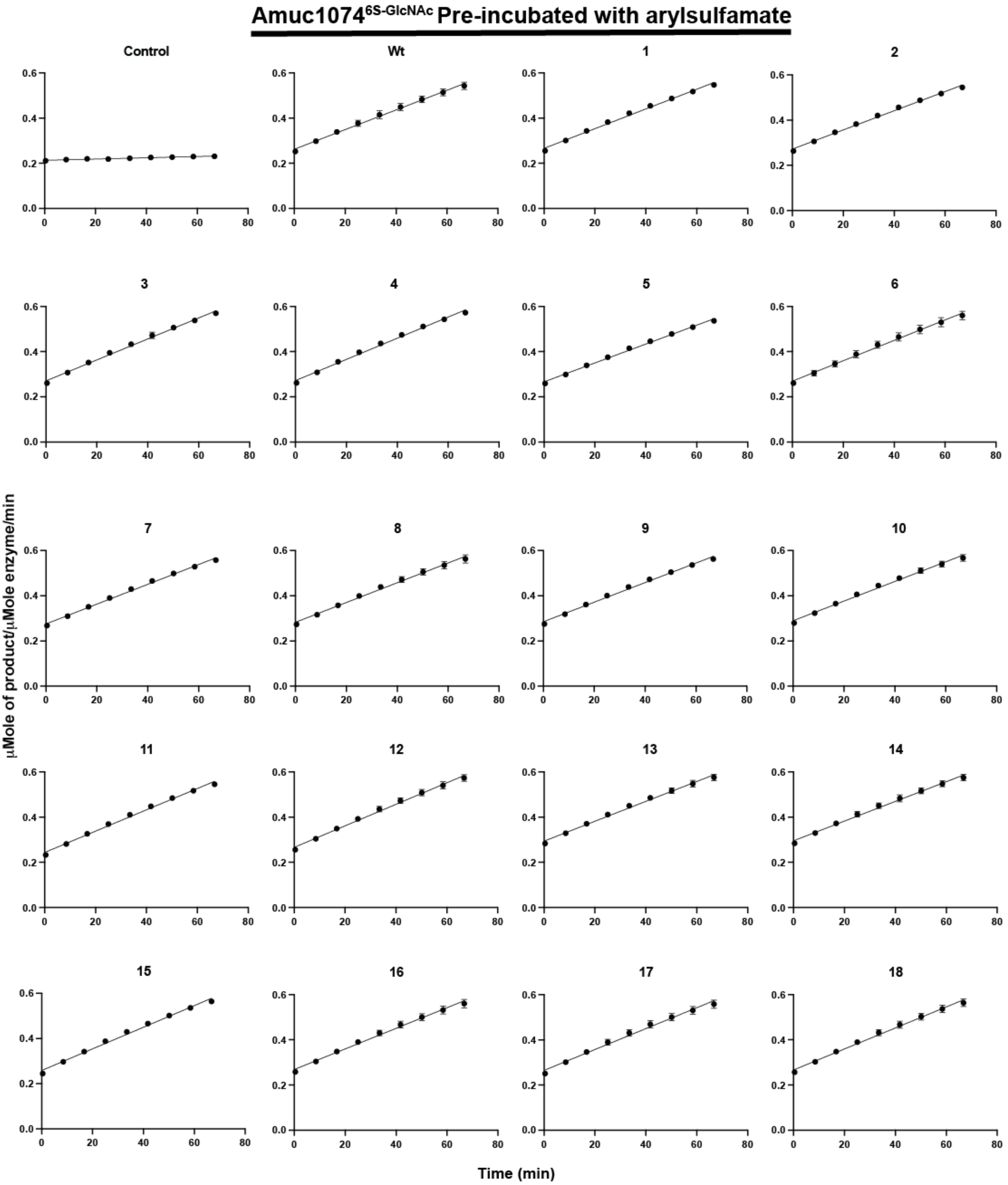

Supplementary figure 17. Kinetic curves of Amuc1074$^{6S\text{-}GlcNAc}$ assayed against BODIPY labelled 6S-*N*-acetylglucosamine after pre-incubation of the enzyme with arylsulfamate inhibitors

Amuc1074$^{6S\text{-}GlcNAc}$, which had been incubated for ~24h with 1 mM of the appropriate arylsulfamate inhibitor, was assayed using a concentration of 6.08 µM against 1 µM BODIPY labelled 6S-*N*-acetylglucosamine. The assay was performed in 100 mM of MES pH 6.0 with 5% DMSO, 150 mM NaCl, 0.02% (v/v) Brij-35 and 5 mM $CaCl_2$. Assays were performed in triplicate. Numbers indicate arylsulfamate compounds as in Figure 1.

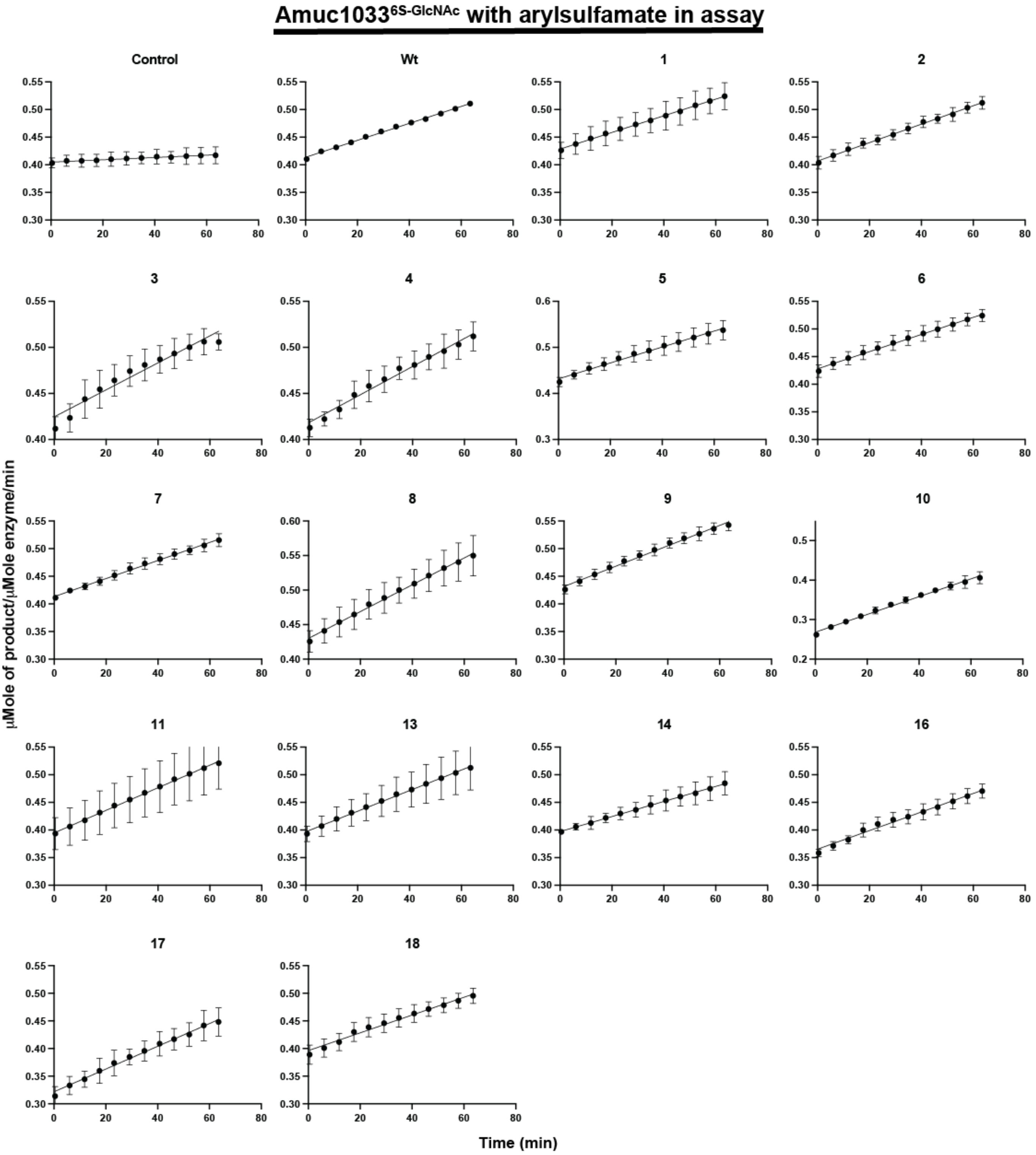

Supplementary figure 18. Kinetic curves of Amuc1033$^{6S\text{-}GlcNAc}$ assayed against BODIPY labelled 6S-*N*-acetylglucosamine with and without arylsulfamate inhibitors

Amuc1033$^{6S\text{-}GlcNAc}$, at a concentration of 4.6 μM, was assayed against 1 μM BODIPY labelled 6S-*N*-acetylglucosamine with and without 1 mM of various arylsulfamate inhibitors included in the assay. The assay was performed in 100 mM of MES pH 6.0 with 5% DMSO, 150 mM NaCl, 0.02% (v/v) Brij-35 and 5 mM $CaCl_2$. Assays were performed in triplicate. Numbers indicate arylsulfamate compounds as in Figure 1.

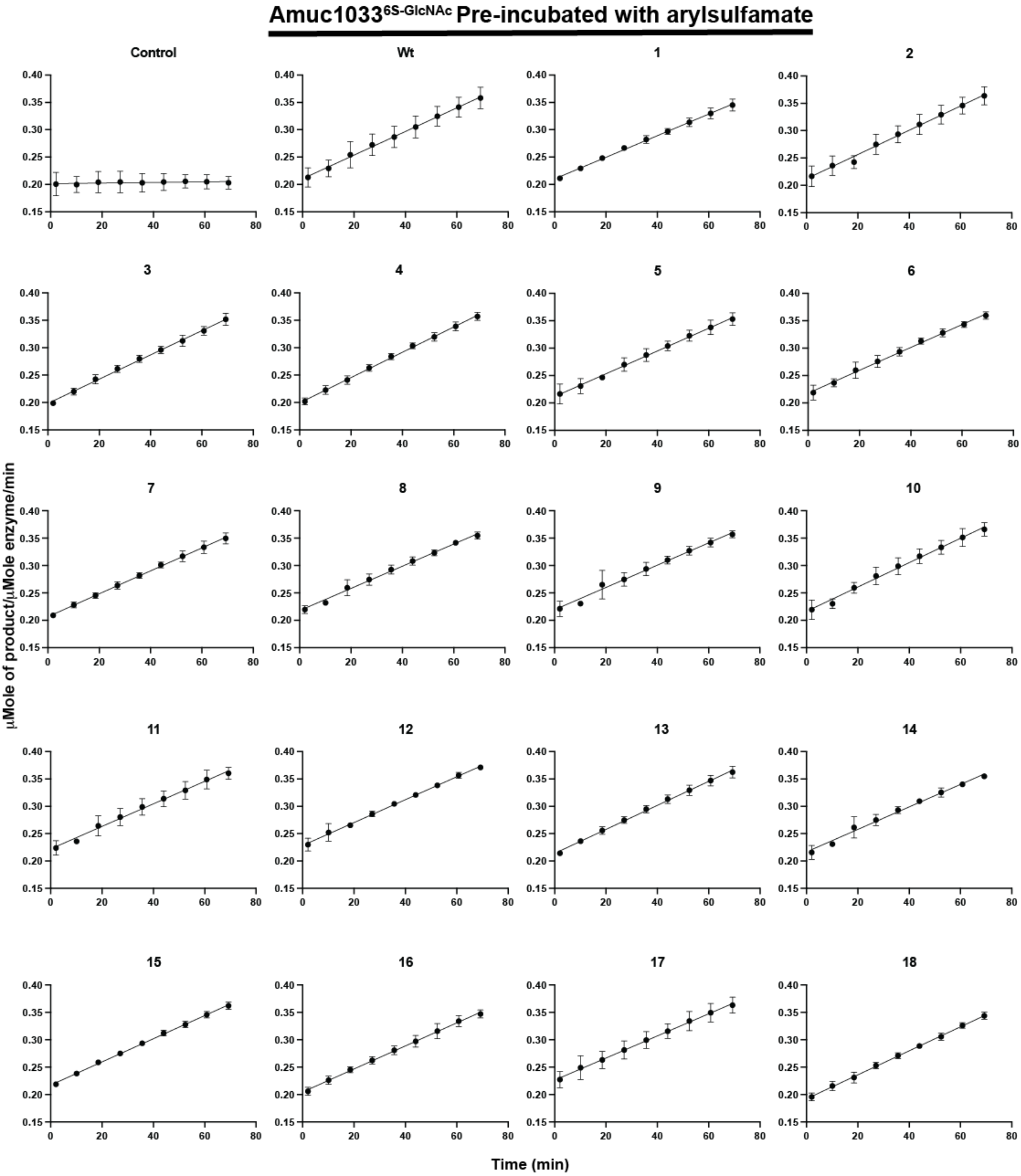

Supplementary figure 19. Kinetic curves of Amuc1033$^{6S\text{-}GlcNAc}$ assayed against BODIPY labelled 6S-*N*-acetylglucosamine after pre-incubation of the enzyme with arylsulfamate inhibitors

Amuc1033$^{6S\text{-}GlcNAc}$, which had been incubated for ~24h with 1 mM of the appropriate arylsulfamate inhibitor, was assayed using a concentration of 4.37 μM against 1 μM BODIPY labelled 6S-*N* acetylglucosamine. The assay was performed in 100 mM of MES pH 6.0 with 5% DMSO, 150 mM NaCl, 0.02% (v/v) Brij-35 and 5 mM $CaCl_2$. Assays were performed in triplicate. Numbers indicate arylsulfamate compounds as in Figure 1.

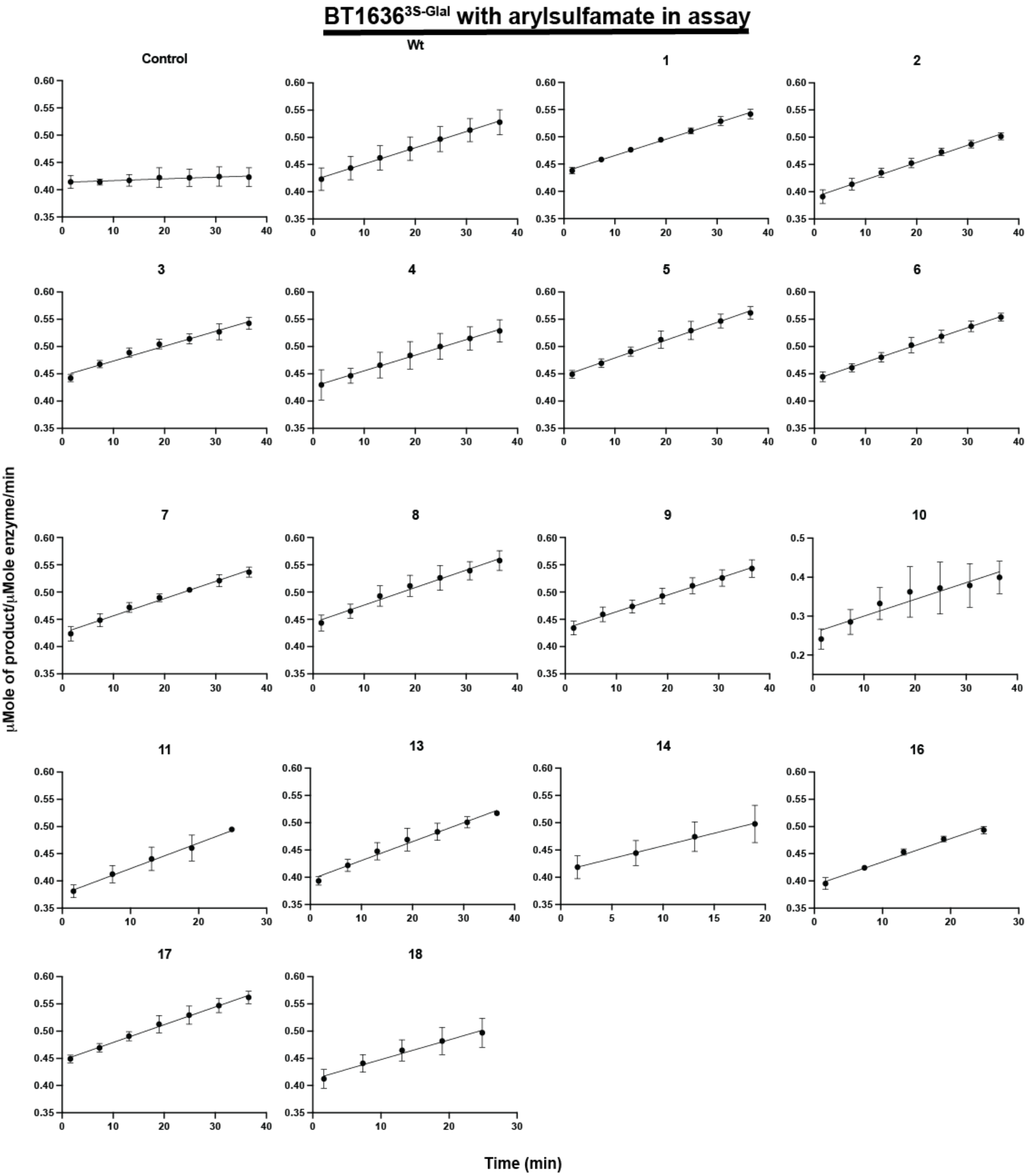

Supplementary figure 20. Kinetic curves of BT1636$^{3S\text{-}Gal}$ assayed against BODIPY labelled 3S-galactose with and without arylsulfamate inhibitors

BT1636$^{3S\text{-}Gal}$, at a concentration of 400 nM, was assayed against 1 μM BODIPY labelled 3S-galactose with and without 1 mM of various arylsulfamate inhibitors included in the assay. The assay was performed in 100 mM of MES pH 6.0 with 5% DMSO, 150 mM NaCl, 0.02% (v/v) Brij-35 and 5 mM $CaCl_2$. Assays were performed in triplicate. Numbers indicate arylsulfamate compounds as in Figure 1.

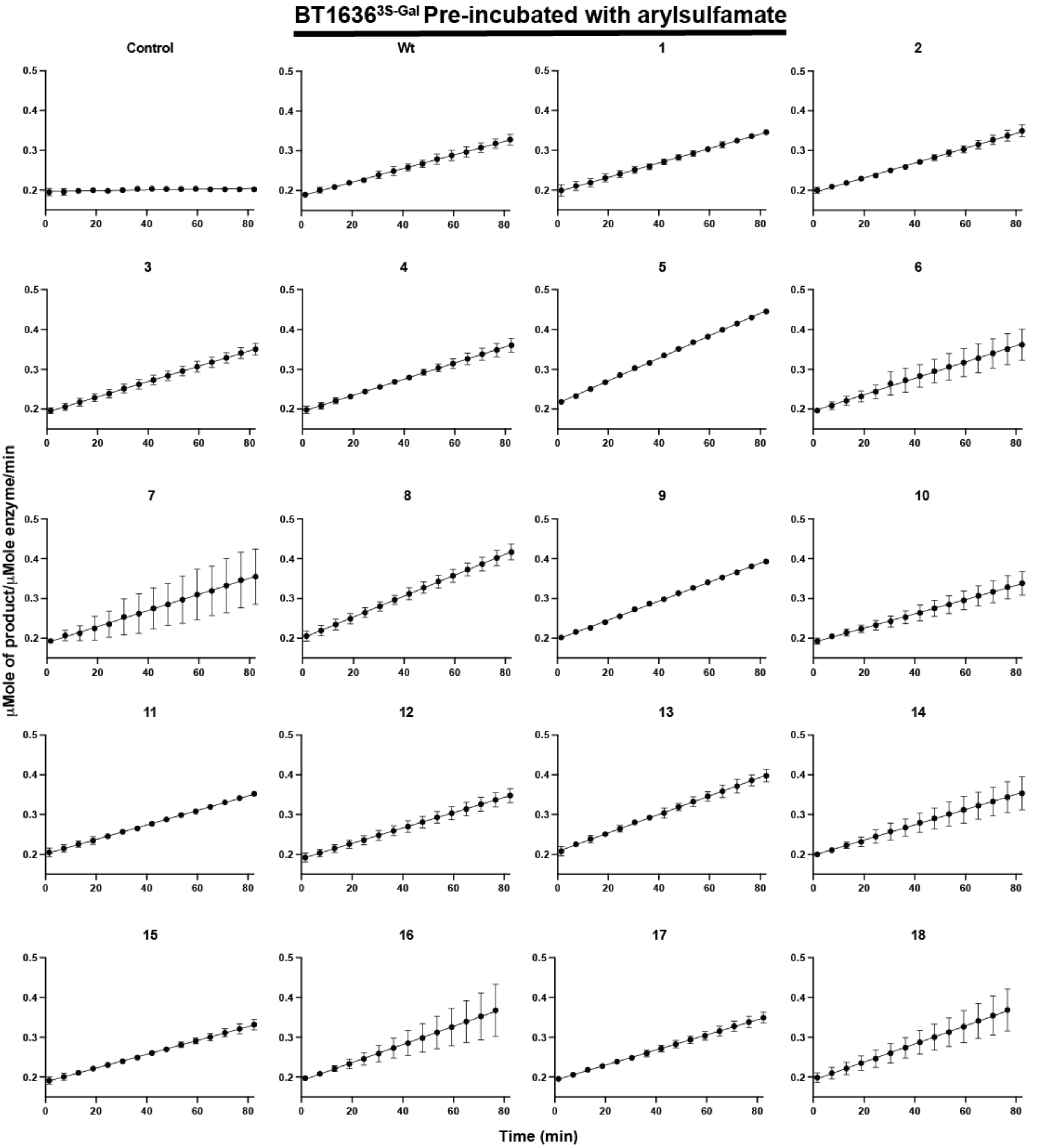

Supplementary figure 21. Kinetic curves of BT1636$^{3S\text{-}Gal}$ assayed against BODIPY labelled 3S-galactose sulfate after pre-incubation of the enzyme with arylsulfamate inhibitors

BT1636$^{3S\text{-}Gal}$, which had been incubated for ~24h with 1 mM of the appropriate arylsulfamate inhibitor, was assayed using a concentration of 200 nM against 1 μM BODIPY labelled 3S-galactose. The assay was performed in 100 mM of MES pH 6.0 with 5% DMSO, 150 mM NaCl, 0.02% (v/v) Brij-35 and 5 mM $CaCl_2$. Assays were performed in triplicate. Numbers indicate arylsulfamate compounds as in Figure 1.

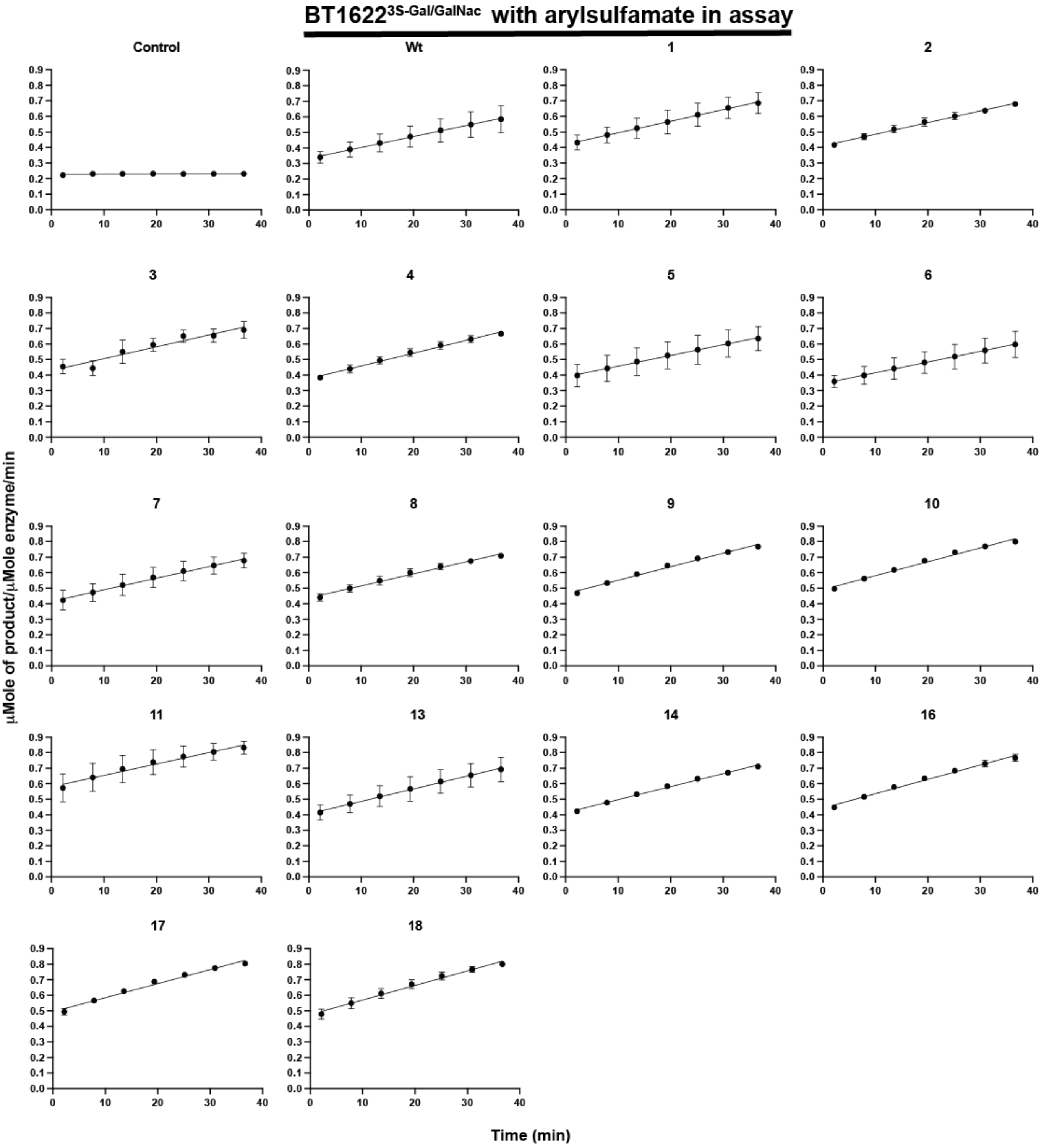

Supplementary figure 22. Kinetic curves of BT1622$^{3S-Gal/GalNAc}$ assayed BODIPY labelled 3S-*N*-acetylgalactosamine with and without arylsulfamate inhibitors

BT1622$^{3S-Gal/GalNAc}$, at a concentration of 6.5 µM, was assayed against 1 µM BODIPY labelled 3S-*N*-acetylgalactosamine with and without 1 mM of various arylsulfamate inhibitors included in the assay. The assay was performed in 100 mM of BTP pH 8.5 with 5% DMSO, 150 mM NaCl, 0.02% (v/v) Brij-35 and 5 mM $CaCl_2$. Assays were performed in triplicate. Numbers indicate arylsulfamate compounds as in Figure 1.

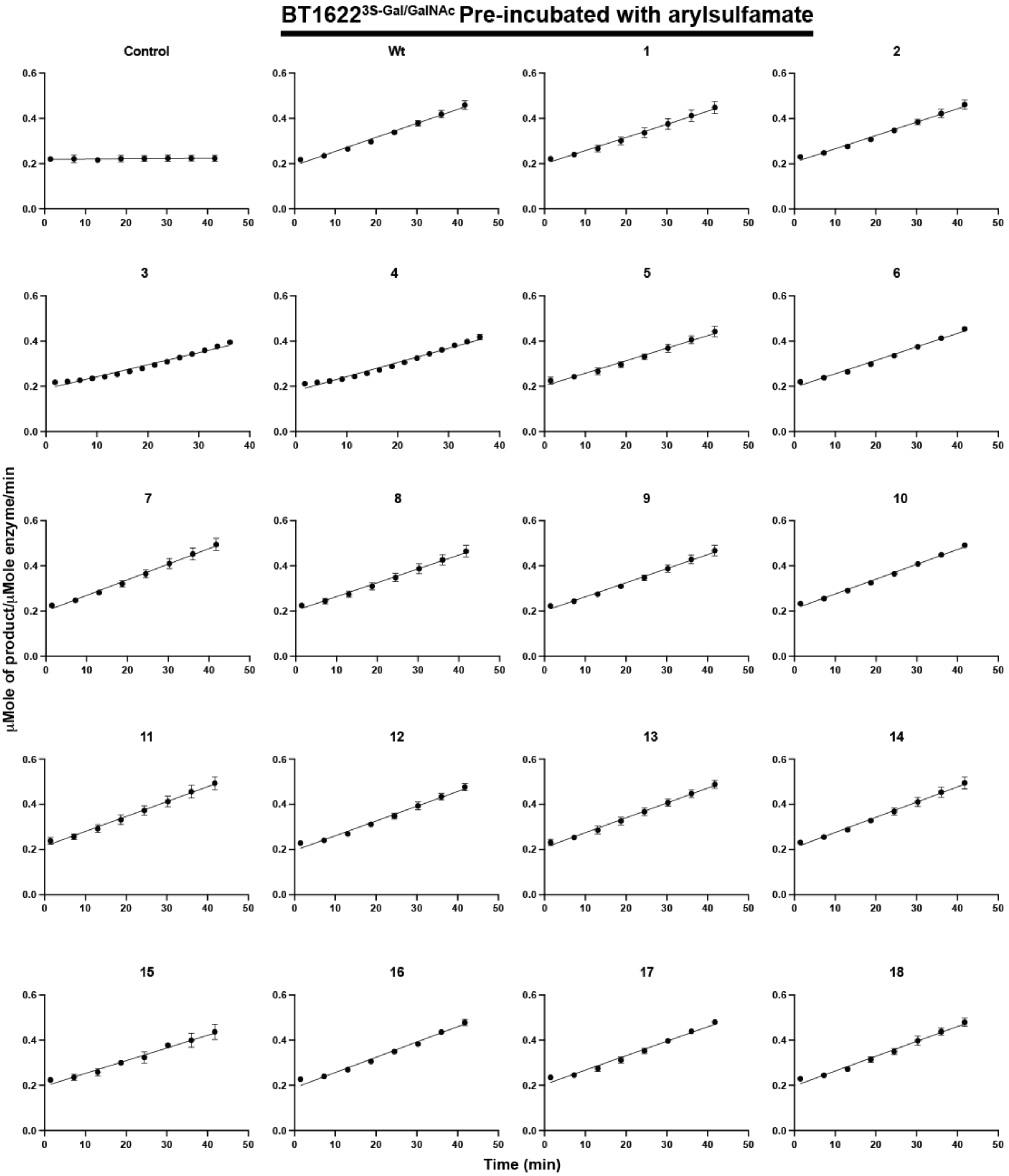

Supplementary figure 23. Kinetic curves of BT1622$^{3S\text{-}Gal/GalNAc}$ assayed against BODIPY labelled 3S-*N*-acetylgalactosamine after pre-incubation of the enzyme with arylsulfamate inhibitors
BT1622$^{3S\text{-}Gal/GalNAc}$, which had been incubated for ~24h with 1 mM of the appropriate arylsulfamate inhibitor, was assayed using a concentration of 2 μM against 1 μM BODIPY labelled 3S-*N*-acetylgalactosamine. The assay was performed in 100 mM of BTP pH 8.5 with 5% DMSO, 150 mM NaCl, 0.02% (v/v) Brij-35 and 5 mM $CaCl_2$. Assays were performed in triplicate. Numbers indicate arylsulfamate compounds as in Figure 1.

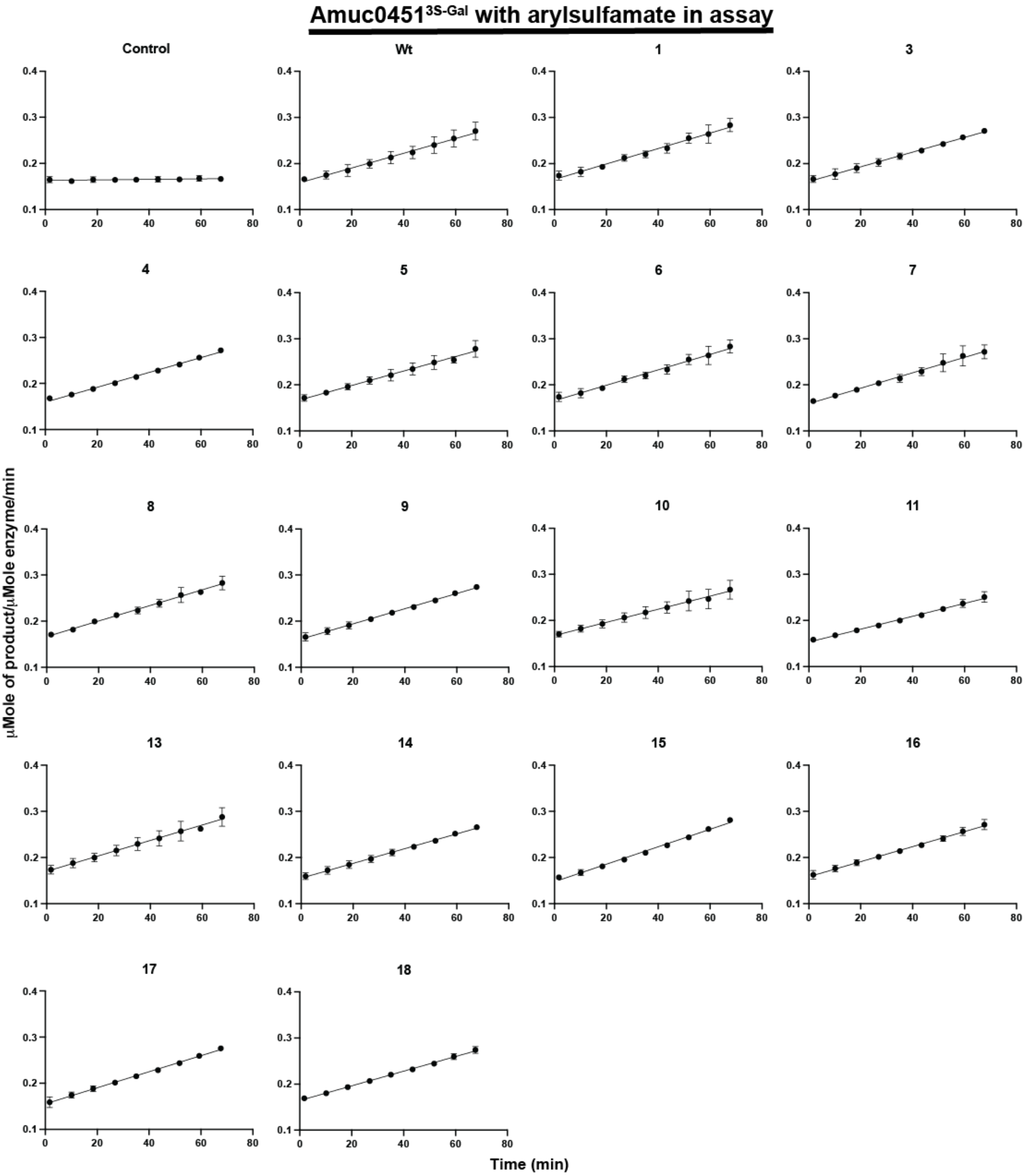

Supplementary figure 24. Kinetic curves of Amuc0451$^{3S\text{-}Gal}$ assayed against BODIPY labelled 3S-galactose with and without arylsulfamate inhibitors

Amuc0451$^{3S\text{-}Gal}$, at a concentration of 15.6 µM, was assayed against 1 µM BODIPY labelled 3S-galactose with and without 1 mM of various arylsulfamate inhibitors included in the assay. The assay was performed in 100 mM of MES pH 6.0 with 5% DMSO, 150 mM NaCl, 0.02% (v/v) Brij-35 and 5 mM $CaCl_2$. Assays were performed in triplicate. Numbers indicate arylsulfamate compounds as in Figure 1.

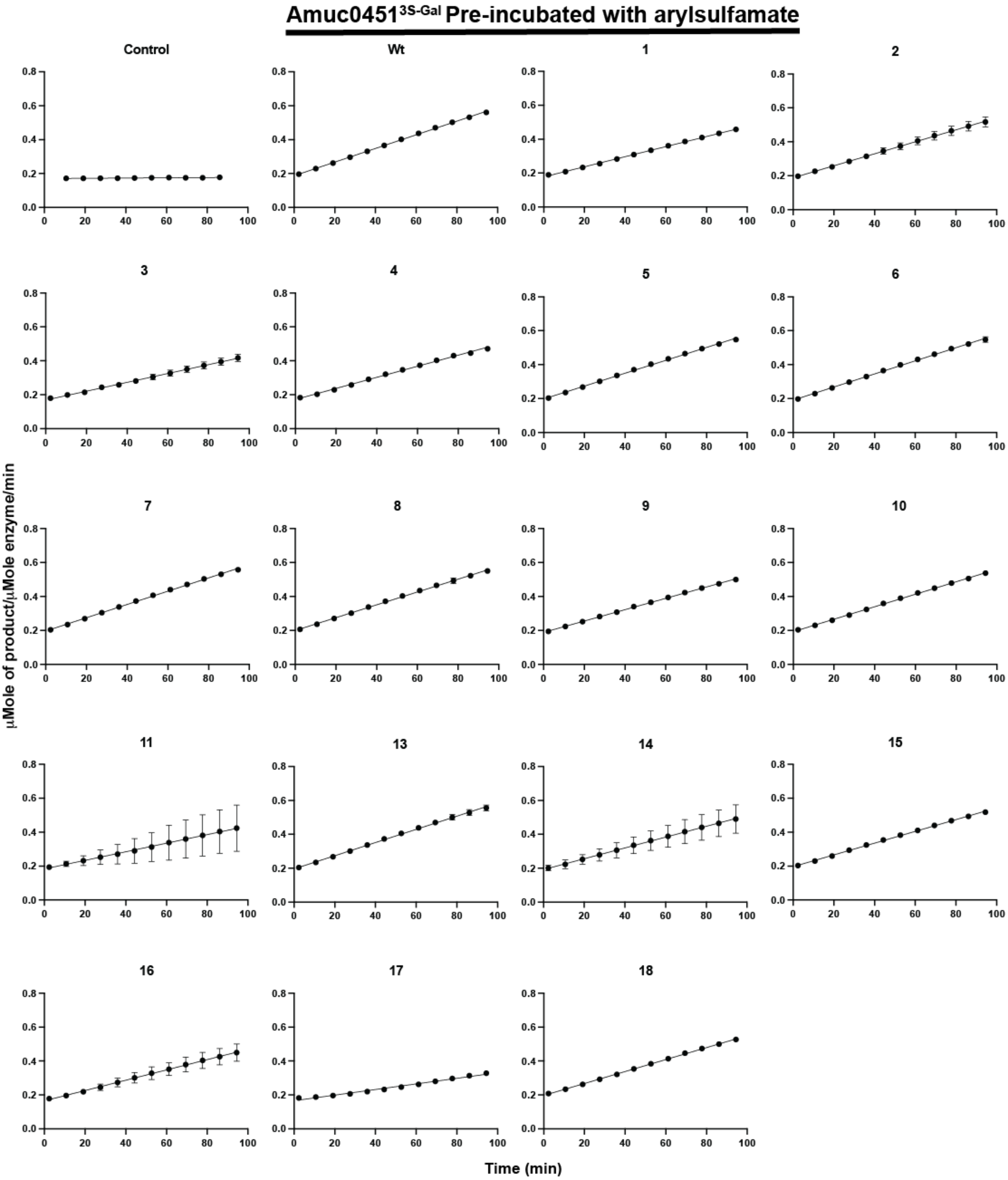

Supplementary figure 25. Kinetic curves of Amuc0451$^{3S\text{-}Gal}$ assayed against BODIPY labelled 3S-galactose after pre-incubation of the enzyme with arylsulfamate inhibitors

Amuc0451$^{3S\text{-}Gal}$, which had been incubated for ~24h with 1 mM of the appropriate arylsulfamate inhibitor, was assayed using a concentration of 15.6 μM against 1 μM BODIPY labelled 3S-galactose. The assay was performed in 100 mM of MES pH 6.0 with 5% DMSO, 150 mM NaCl, 0.02% (v/v) Brij-35 and 5 mM $CaCl_2$. Assays were performed in triplicate. Numbers indicate arylsulfamate compounds as in Figure 1.

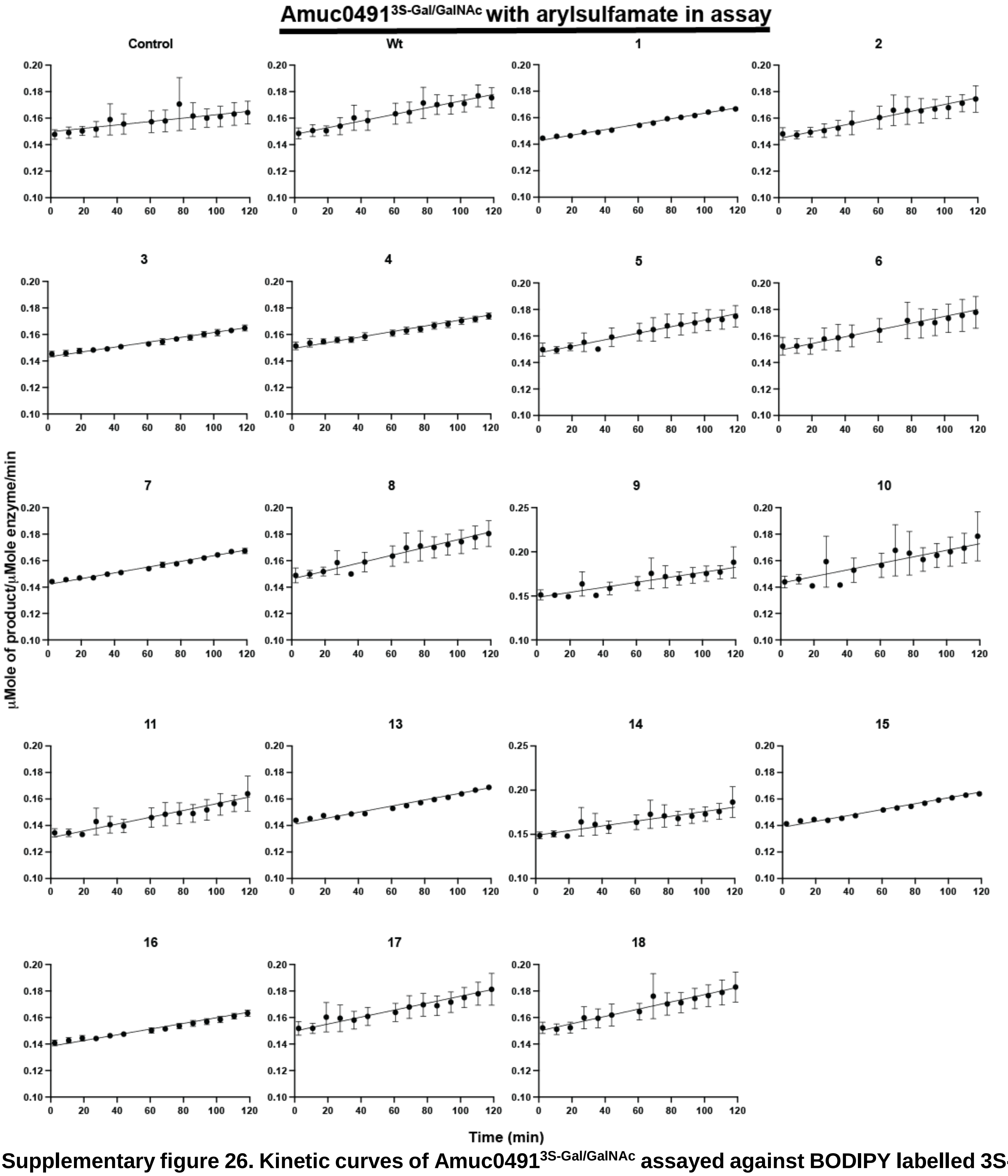

Supplementary figure 26. Kinetic curves of Amuc0491$^{3S\text{-}Gal/GalNAc}$ assayed against BODIPY labelled 3S-*N*-acetylgalactosamine with and without arylsulfamate inhibitors

Amuc0491$^{3S\text{-}Gal/GalNAc}$, at a concentration of 3.4 μM, was assayed against 1 μM BODIPY labelled 3S-*N*-acetylgalactosamine with and without 1 mM of various arylsulfamate inhibitors included in the assay. The assay was performed in 100 mM of MES pH 6.0 with 5% DMSO, 150 mM NaCl, 0.02% (v/v) Brij-35 and 5 mM $CaCl_2$. Assays were performed in triplicate. Numbers indicate arylsulfamate compounds as in Figure 1.

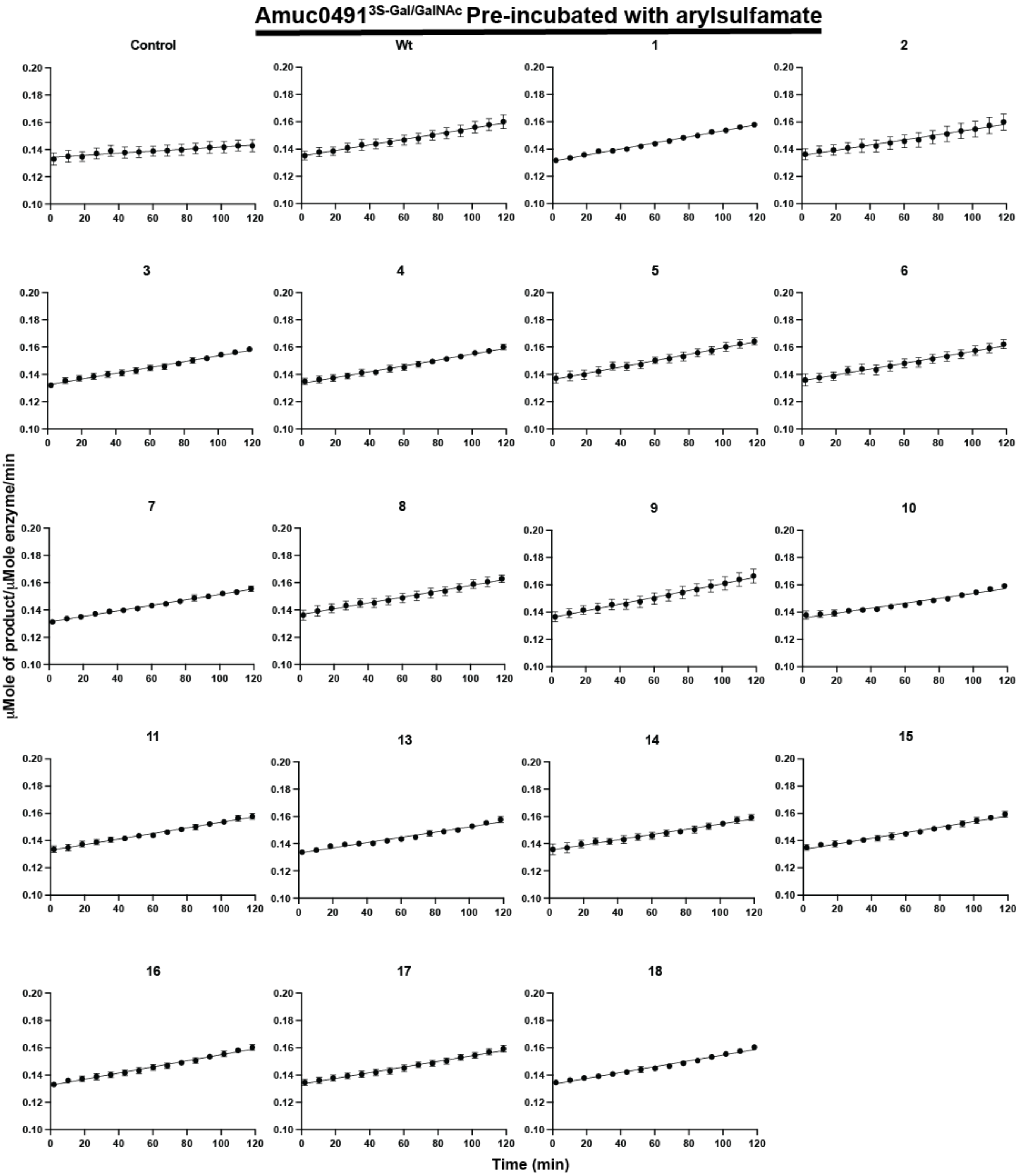

Supplementary figure 27. Kinetic curves of Amuc0491$^{3S\text{-}Gal/GalNAc}$ assayed against BODIPY labelled 3S-*N*-acetylgalactosamine after pre-incubation of the enzyme with arylsulfamate inhibitors
Amuc0491$^{3S\text{-}Gal/GalNAc}$, which had been incubated for ~24h with 1 mM of the appropriate arylsulfamate inhibitor, was assayed using a concentration of 3.2 μM against 1 μM BODIPY labelled 3S-*N*-acetylgalactosamine. The assay was performed in 100 mM of MES pH 6.0 with 5% DMSO, 150 mM NaCl, 0.02% (v/v) Brij-35 and 5 mM $CaCl_2$. Assays were performed in triplicate. Numbers indicate arylsulfamate compounds as in Figure 1.

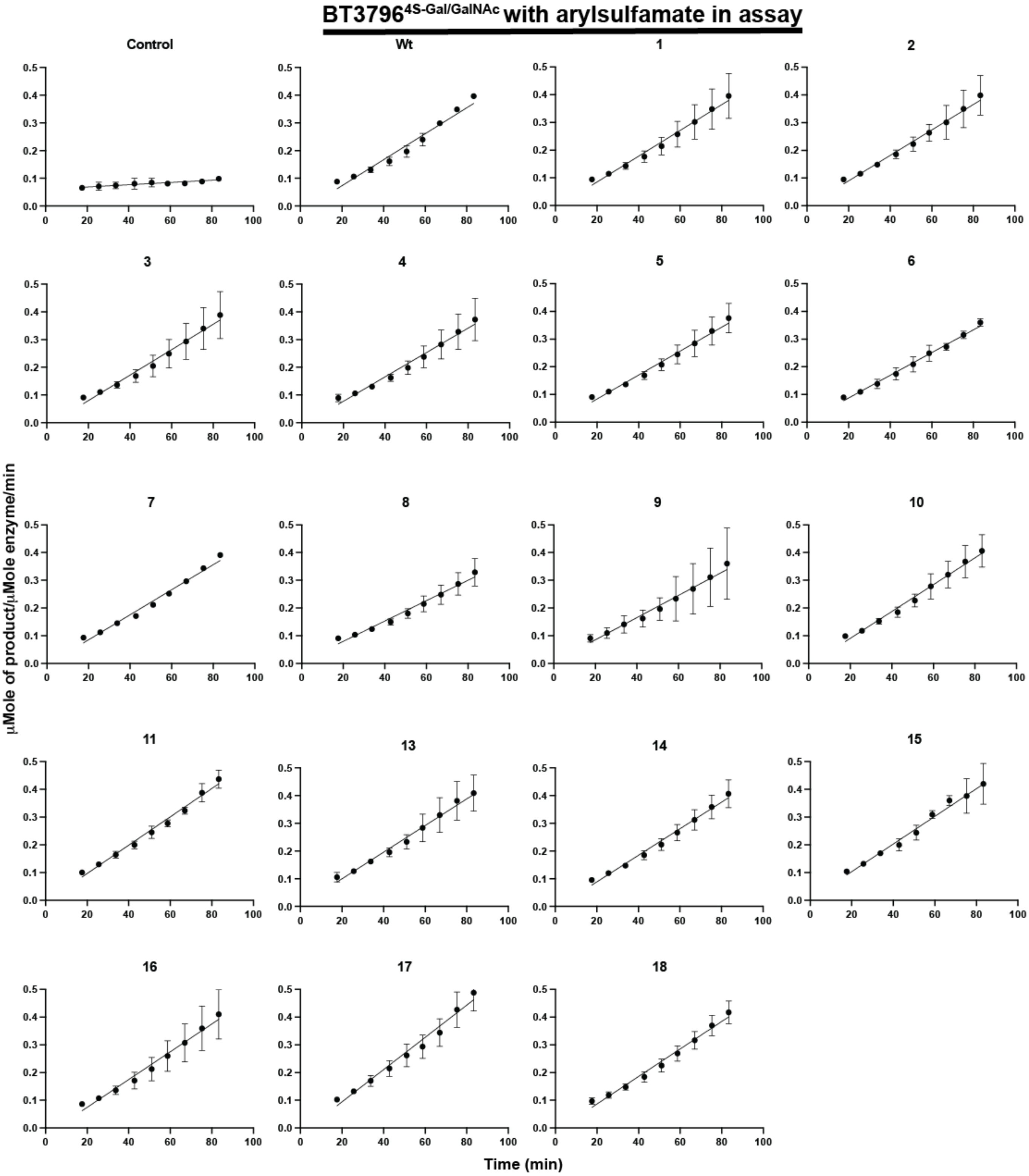

Supplementary figure 28. Kinetic curves of BT3796$^{4S-Gal/GalNAc}$ assayed against BODIPY labelled 4S-galactose with and without arylsulfamate inhibitors

BT3796$^{4S-Gal/GalNAc}$, at a concentration of 630 nM, was assayed against 1 µM BODIPY labelled 4S-galactose with and without 1 mM of various arylsulfamate inhibitors included in the assay. The assay was performed in 100 mM of BTP pH 8.5 with 5% DMSO, 150 mM NaCl, 0.02% (v/v) Brij-35 and 5 mM $CaCl_2$. Assays were performed in triplicate. Numbers indicate arylsulfamate compounds as in Figure 1.

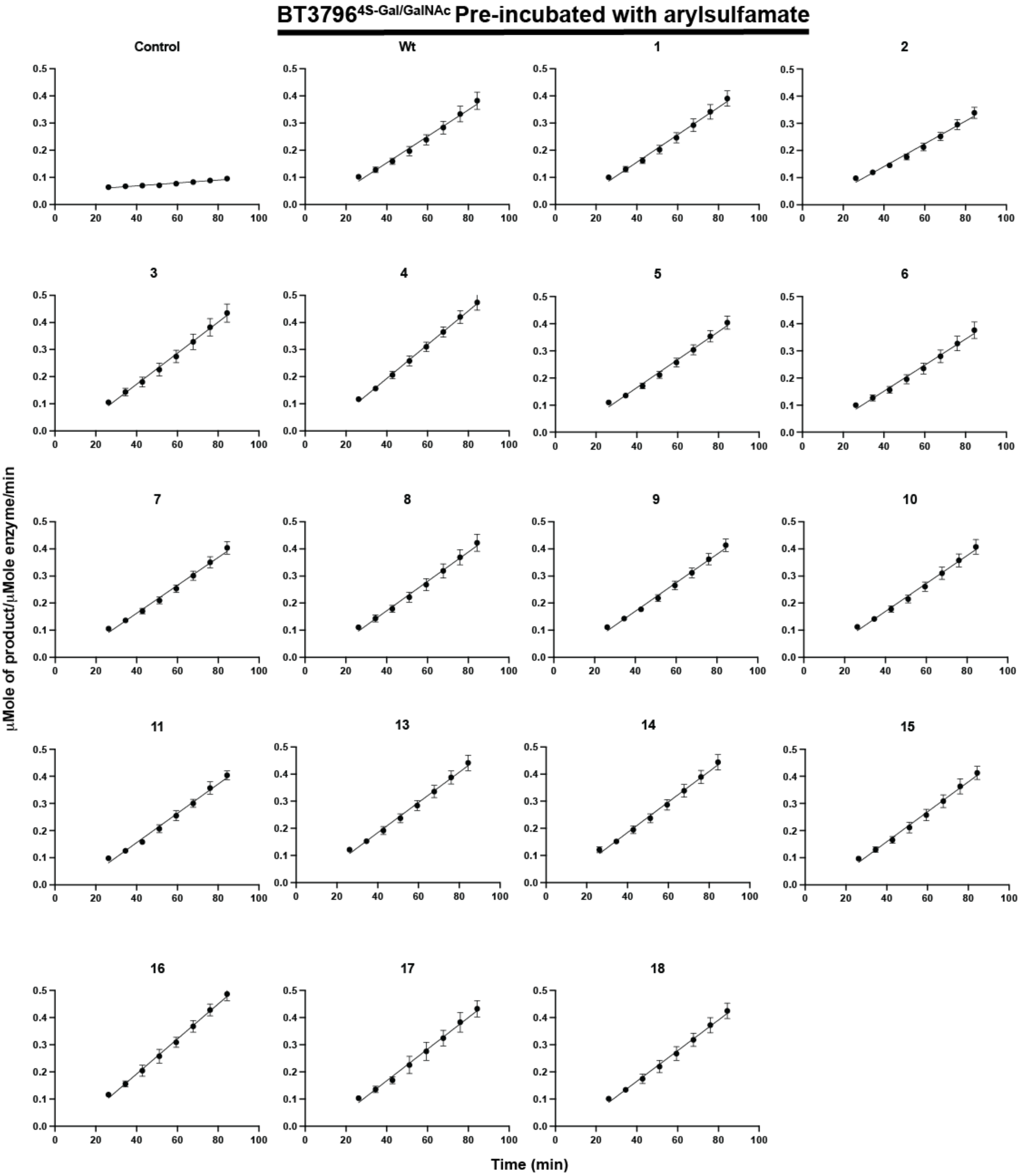

Supplementary figure 29. Kinetic curves of BT3796$^{4S-Gal/GalNAc}$ assayed against BODIPY labelled 4S-galactose after pre-incubation of the enzyme with arylsulfamate inhibitors

BT3796$^{4S-Gal/GalNAc}$, which had been incubated for ~24h with 1 mM of the appropriate arylsulfamate inhibitor, was assayed using a concentration of 630 nM against 1 μM BODIPY labelled 4S-galactose. The assay was performed in 100 mM of BTP pH 8.5 with 5% DMSO, 150 mM NaCl, 0.02% (v/v) Brij-35 and 5 mM $CaCl_2$. Assays were performed in triplicate. Numbers indicate arylsulfamate compounds as in Figure 1.

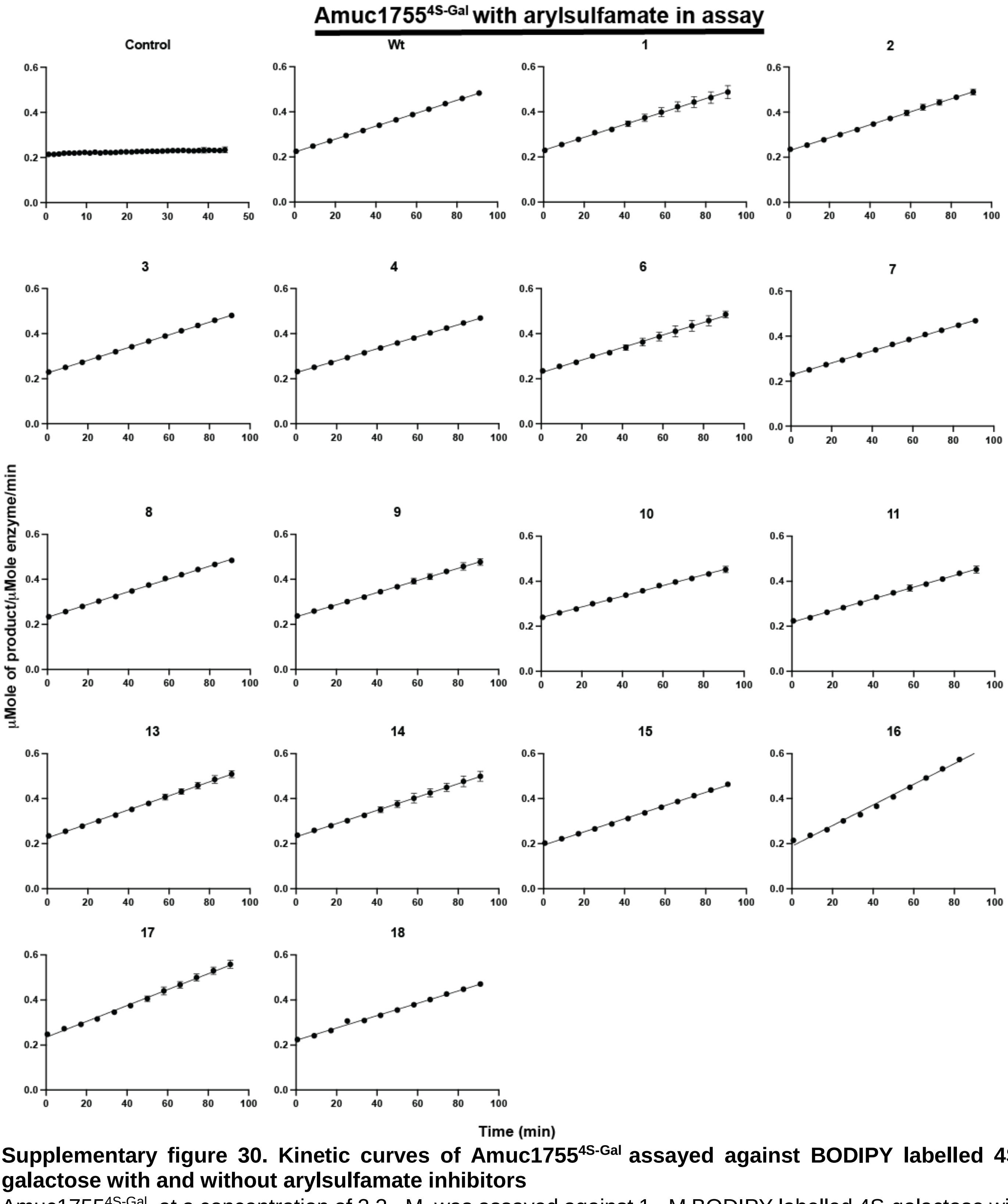

Supplementary figure 30. Kinetic curves of Amuc1755$^{4S\text{-}Gal}$ assayed against BODIPY labelled 4S-galactose with and without arylsulfamate inhibitors

Amuc1755$^{4S\text{-}Gal}$, at a concentration of 2.3 µM, was assayed against 1 µM BODIPY labelled 4S-galactose with and without 1 mM of various arylsulfamate inhibitors included in the assay. The assay was performed in 100 mM of MES pH 6.0 with 5% DMSO, 150 mM NaCl, 0.02% (v/v) Brij-35 and 5 mM $CaCl_2$. Assays were performed in triplicate. Numbers indicate arylsulfamate compounds as in Figure 1.

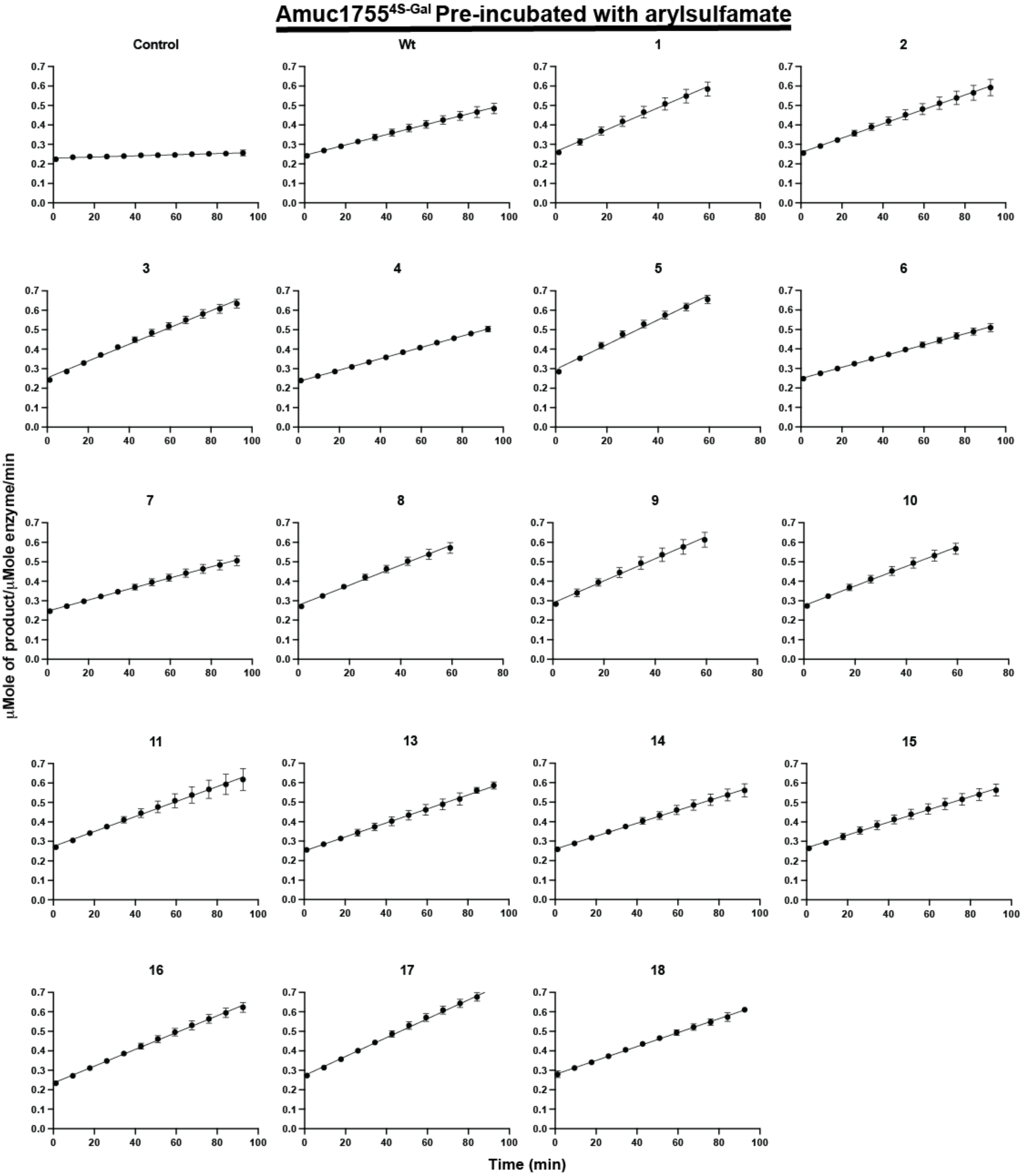

Supplementary figure 31. Kinetic curves of Amuc1755$^{4S\text{-}Gal}$ assayed against BODIPY labelled 4S-galactose after pre-incubation of the enzyme with arylsulfamate inhibitors

Amuc1755$^{4S\text{-}Gal}$, which had been incubated for ~24 h with 1 mM of the appropriate arylsulfamate inhibitor, was assayed using a concentration of 2.185 μM against 1 μM BODIPY labelled 4S-galactose. The assay was performed in 100 mM of MES pH 6.0 with 5% DMSO, 150 mM NaCl, 0.02% (v/v) Brij-35 and 5 mM $CaCl_2$. Assays were performed in triplicate. Numbers indicate arylsulfamate compounds as in Figure 1.

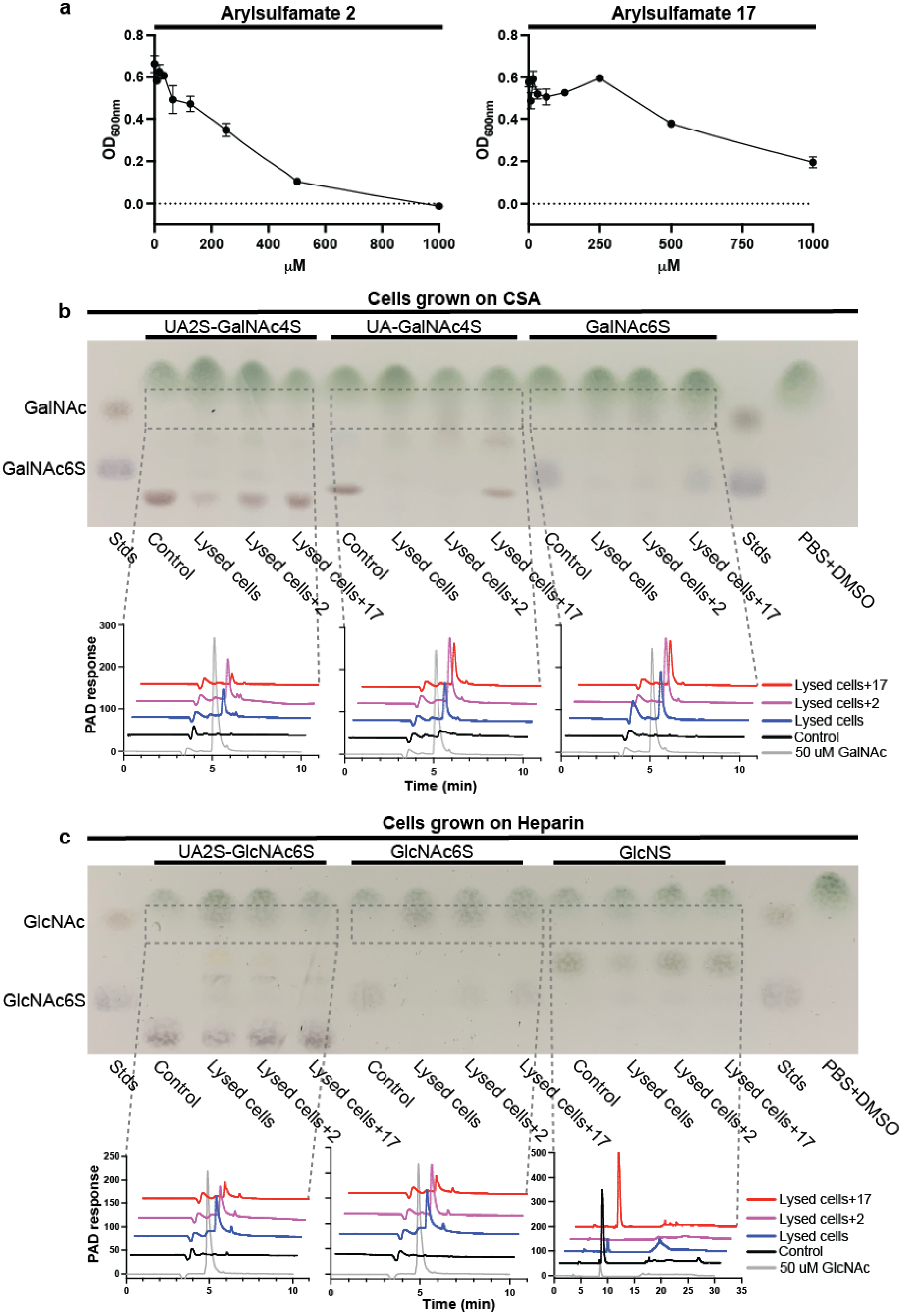
a
Arylsulfamate 2
Arylsulfamate 17
OD600nm
μM
b
Cells grown on CSA
UA2S-GalNAc4S
UA-GalNAc4S
GalNAc6S
GalNAc
GalNAc6S
Stds
Control
Lysed cells
Lysed cells+2
Lysed cells+17
PBS+DMSO
PAD response
Time (min)
50 uM GalNAc
c
Cells grown on Heparin
UA2S-GlcNAc6S
GlcNAc6S
GlcNS
GlcNAc
GlcNAc6S
50 uM GlcNAc

Supplementary figure 32 Cell lysates of Bacteroides thetaiotaomicron incubated with two arylsulfamates inhibitors

a. The effects of varied concentrations of arylsulfamate 2 and 17 on the growth of *B. theta* in BHI media. **b.** Thin layer chromatography (TLC) analysis of cell lysates from B.theta cells grown to mid-exponential phase on CSA as a carbon source, and incubated with arylsulfamates 2 and 17 (Figure 2), mixed 1:1 with the sulfated substrates: O2 sulfated uronic acid β1,3 linked to O4 sulfated N-acetylgalactosmaine (UA2S-GalNAc4S), uronic acid β1,3 linked to O4 sulfated N-acetylgalactosmaine (UA-GalNAc4S), and O6 sulfated N-acetylgalactosmaine (GalNAc6S). Below are high performance anion exchange chromatography (HPAEC) traces showing the production of GalNAc in all instances except the negative control; **c.** Thin layer chromatography (TLC) analysis of cell lysates from B.theta cells grown to mid-exponential phase on Heparin as a carbon source, and incubated with arylsulfamates 2 and 17 (Figure 2), mixed 1:1 with the sulfated substrates: O2 sulfated uronic acid α1,4 linked to O4 sulfated N-acetylgalactosmaine (UA2S-GalNAc4S), O6 sulfated N-acetylglucosmaine (GlcNAc6S), and N-sulfated glucosamine (GlcNS). Below are high performance anion exchange chromatography (HPAEC) traces showing the production of GlcNAc in all instances except the negative control. All reactions were in PBS, with 5 % DMSO, and incubated at 37ºC overnight.

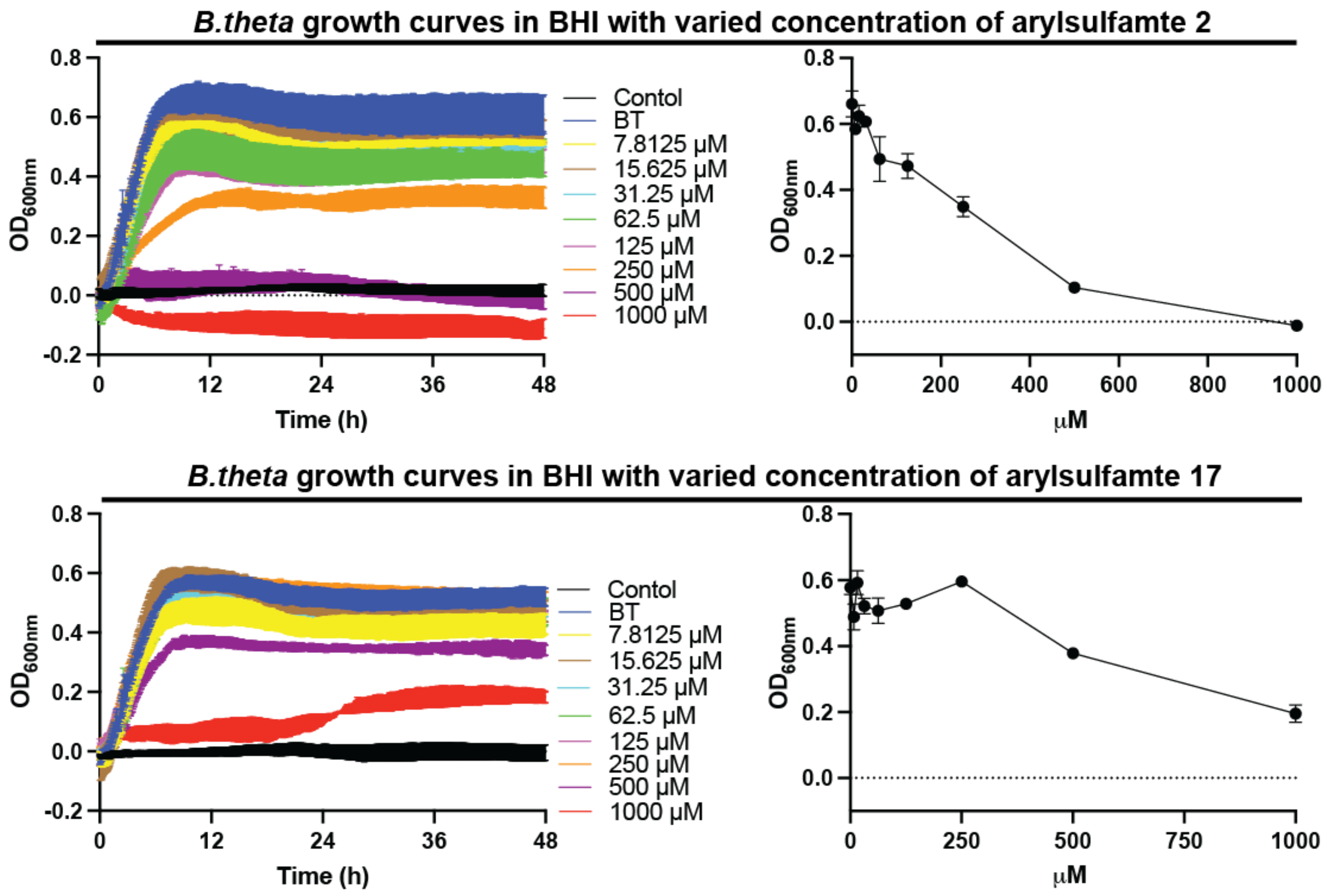

Supplementary figure 33. The effects of varied concentration arylsulfamates 2 and 17 on the growth of *Bacteroides thetaiotaomicron* VPI-5482 grown in BHI media. *Bacteroides thetaiotaomicron* VPI-5482 was grown in BHI, 1 % DMSO, with varied concentrations of arylsulfamate 2 and 17. Data are technical triplicates with the standard error of the mean. Numbers indicate arylsulfamate compound as in Figure 1.

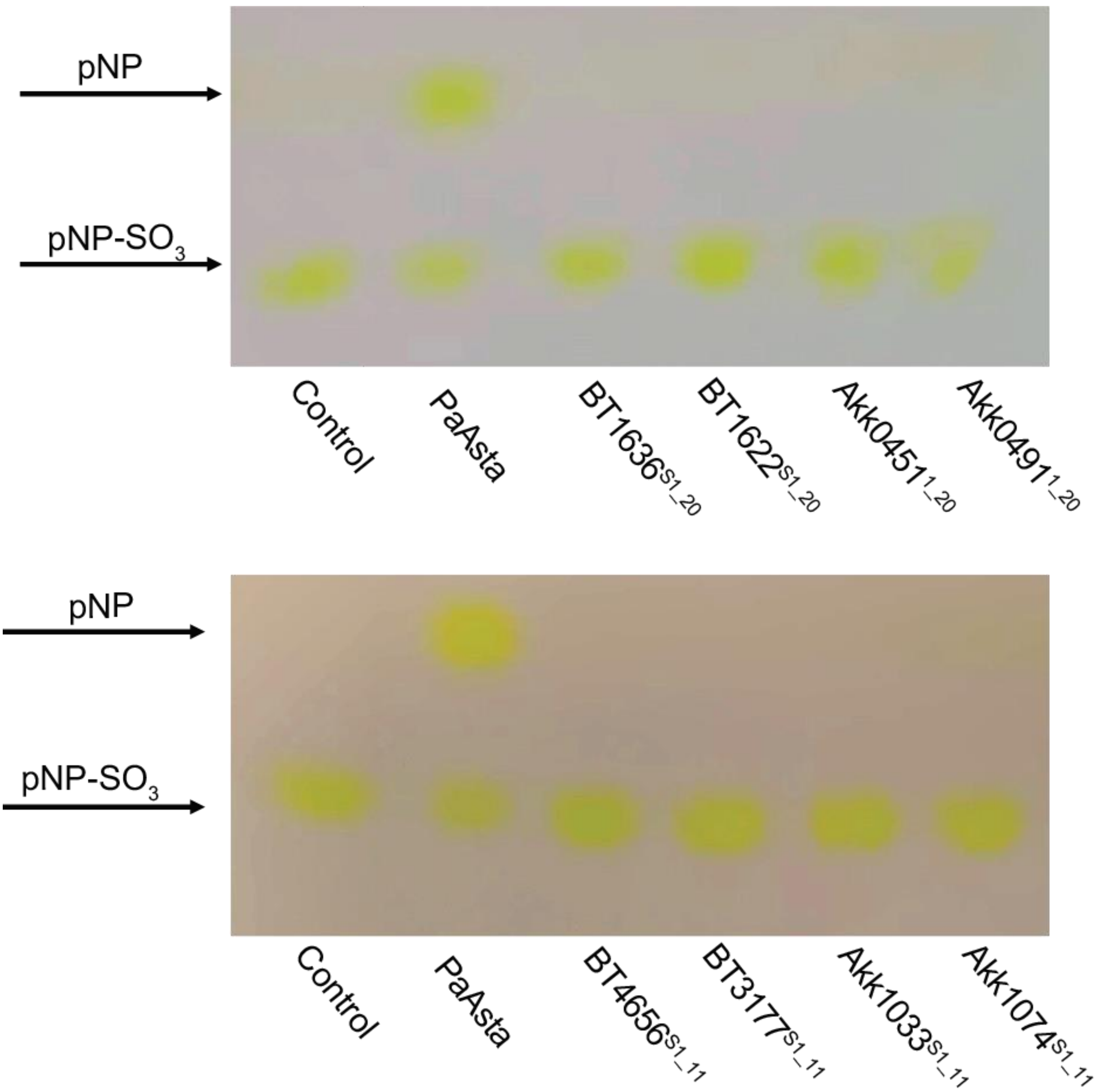

Supplementary figure 34. Activity of carbohydrate sulfatases on para-nitrophenol sulfate.

Sulfatases were incubated with 5 mM para-nitrophenol sulfate over night in 100 mM BTP pH 7.0 with 150 mM NaCl and 5 mM CaCl2. Reactions were then ran on silica based thin layer chromatography using a mobile phase of butanol:acetic acid:water (2:1:1) and develop by soaking the plates with 1 M NaOH and heating with a heat gun. It can be seen only the steroid sulfatase PaAsta shows the production of a para-nitrolphenol product.

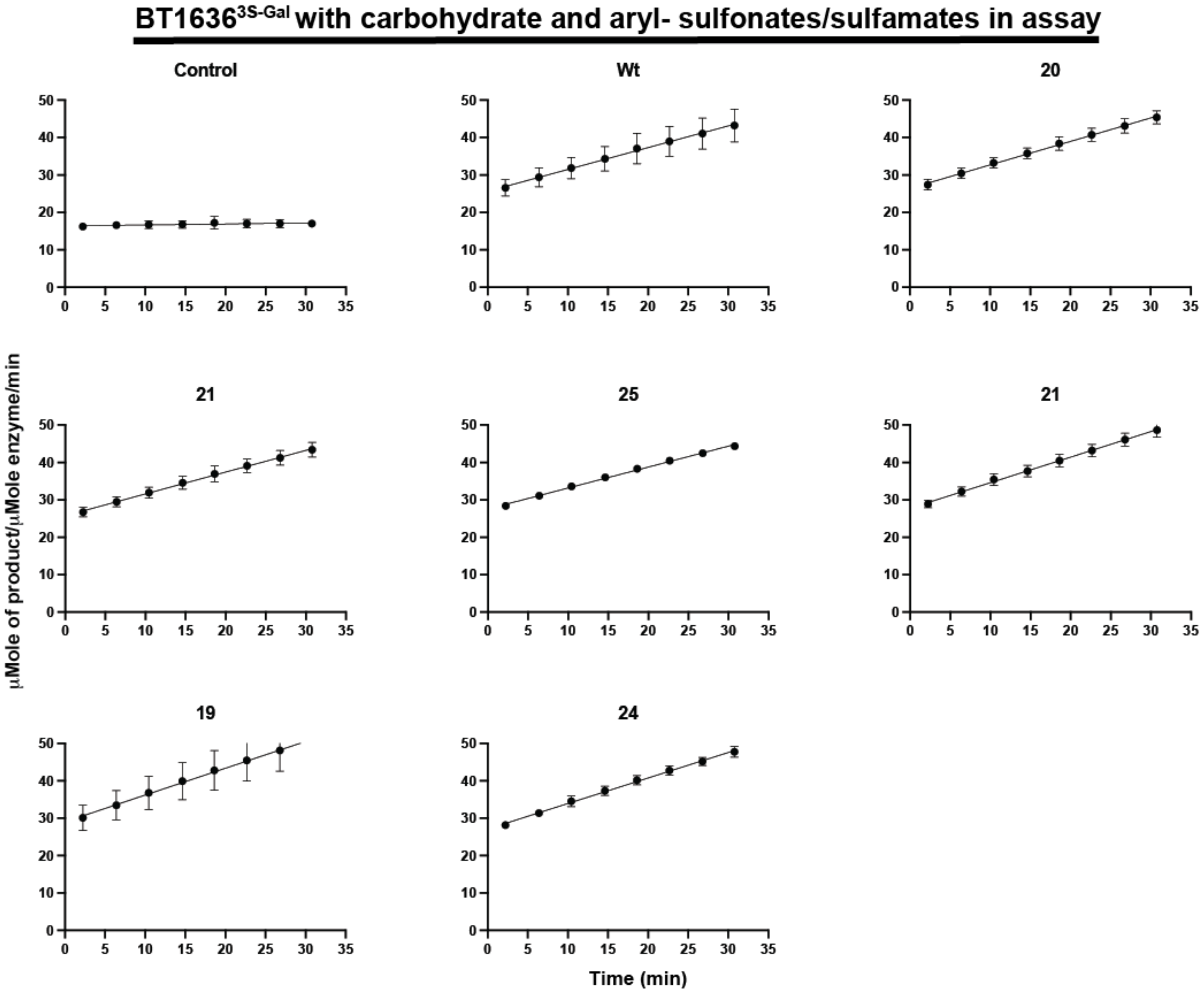

Supplementary figure 35. Kinetic curves of BT1636^{3S-Gal} assayed against BODIPY labelled 3S-galactose with and without carbohydrate and aryl- sulfonates/sulfamates BT1636^{3S-Gal}, at a concentration of 270 nM, was assayed against 1 μM BODIPY labelled 3S-galactose with and without 1 mM of various arylsulfamate inhibitors included in the assay. The assay was performed in 100 mM of MES pH 6.0 with 5% DMSO, 150 mM NaCl, 0.02% (v/v) Brij-35 and 5 mM $CaCl_2$. Assays were performed in triplicate. Numbers indicate arylsulfamate compounds as in Figure 1.

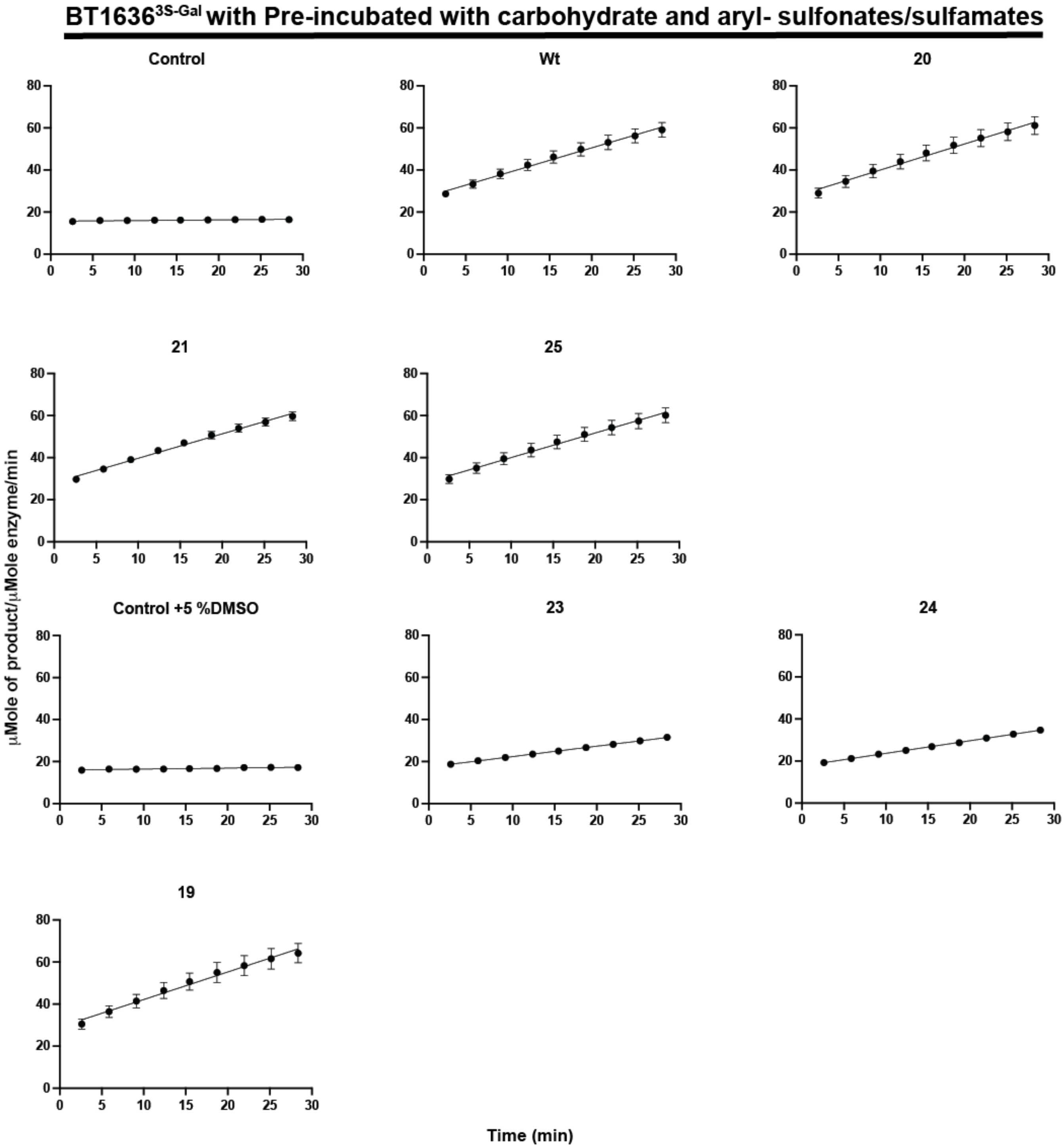

Supplementary figure 36. Kinetic curves of BT1636$^{3S\text{-}Gal}$ assayed against BODIPY labelled 3S-galactose sulfate after pre-incubation of the enzyme with carbohydrate and aryl- sulfonates/sulfamates

BT1636$^{3S\text{-}Gal}$, which had been incubated for ~24h with 1 mM of the appropriate arylsulfamate inhibitor, was assayed using a concentration of 270 nM against 1 µM BODIPY labelled 3S-galactose. The assay was performed in 100 mM of MES pH 6.0 with 5% DMSO, 150 mM NaCl, 0.02% (v/v) Brij-35 and 5 mM $CaCl_2$. Assays were performed in triplicate. Numbers indicate arylsulfamate compounds as in Figure 1.

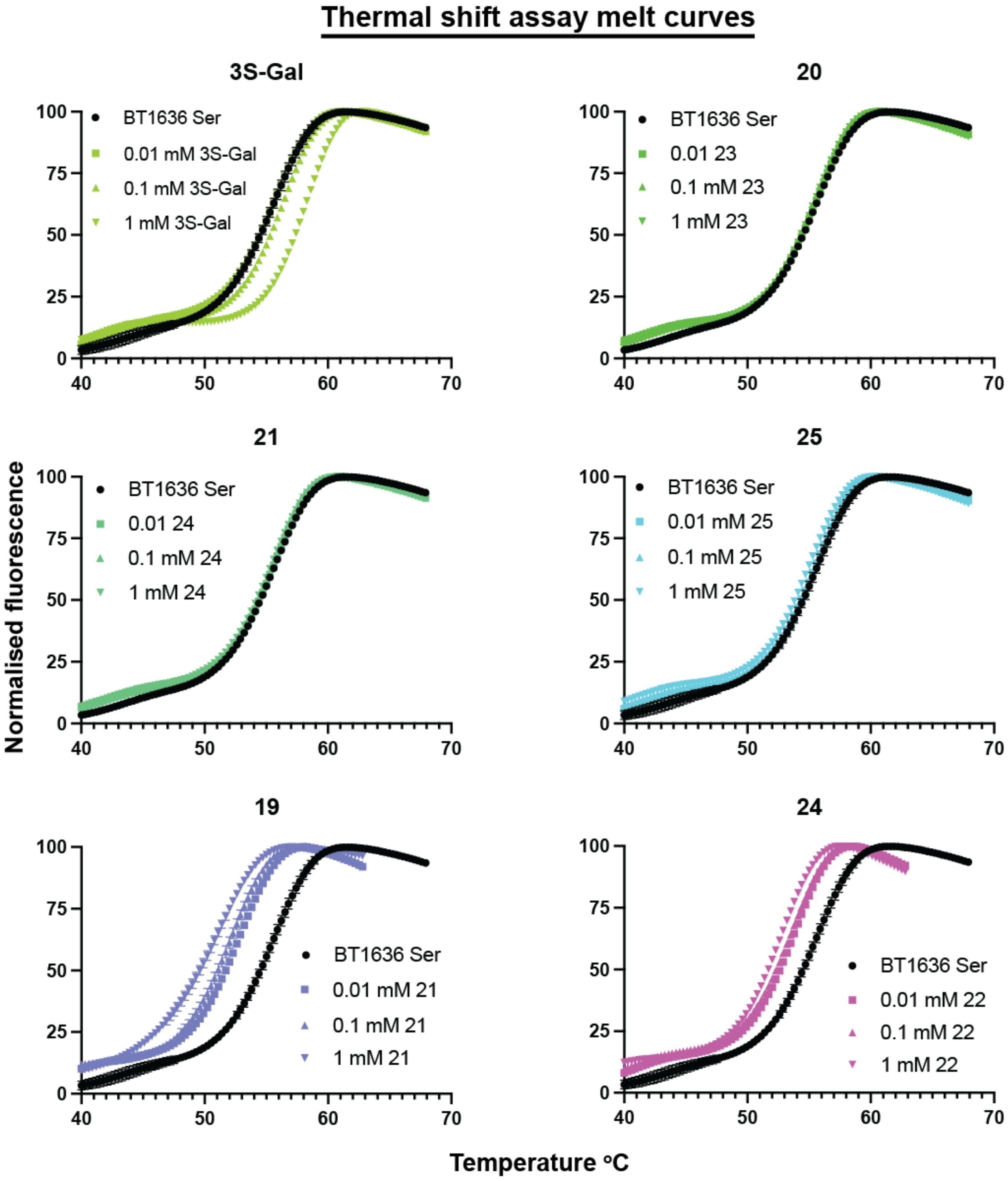

Supplementary figure 37. Thermal melt curves of BT1636$^{3S\text{-}Gal}$ incubated substrate and various carbohydrate and aryl- sulfonates/sulfamates

BT1636$^{3S\text{-}Gal}$, at a concentration of 5 μM, was incubated with no compound, and varying concentrations and the effect on its melting temperature monitored. A shift in melting temperature is indicative of an interaction. The assays were performed in 100 mM of BTP pH 7.0 with 5% DMSO, 150 mM NaCl. Assays were performed in triplicate.

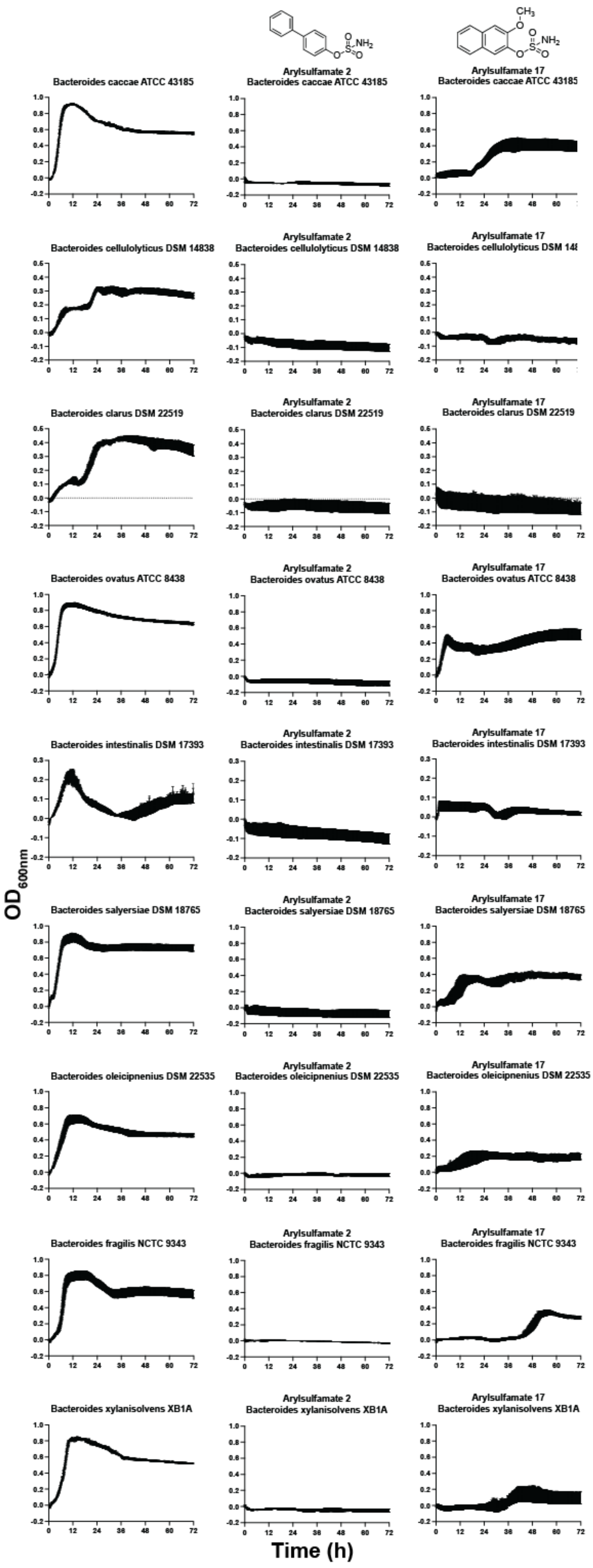

Supplementary figure 38. The effects of arylsulfamates 2 and 17 on the growth of HGM Bacteroidota species

Bacteroidota species were grown in BHI, 1% DMSO, with 1 mM of arylsulfamate 2 and 17 and the effects on growth observed. Data are technical triplicates with the standard error of the mean.

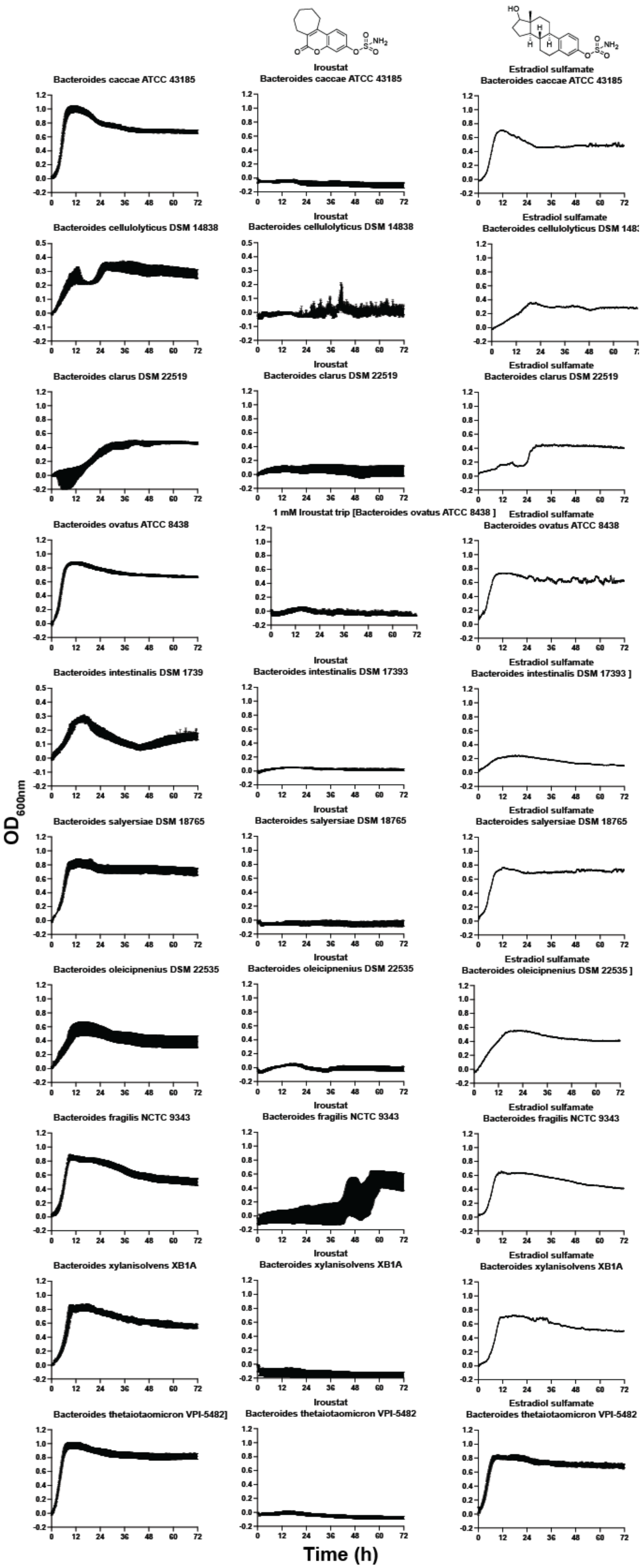

Supplementary figure 39. The effects of the phase I/II arylsulfamate drugs Irosustat and estradiol sulfamate on the growth of Bacteroidota species

Bacteroidota species were grown in BHI, 1% DMSO, with 1 mM of Irosustat and estradiol sulfamate and the effects on growth observed. Growths with Irosustat are technical triplicates with the standard error of the mean. Growths with estradiol sulfamate represent single growth experiments with the exception of *Bacteroides thetaiotaomicron* VPI-5482 where the data is from triplicate growths with standard error of the mean.

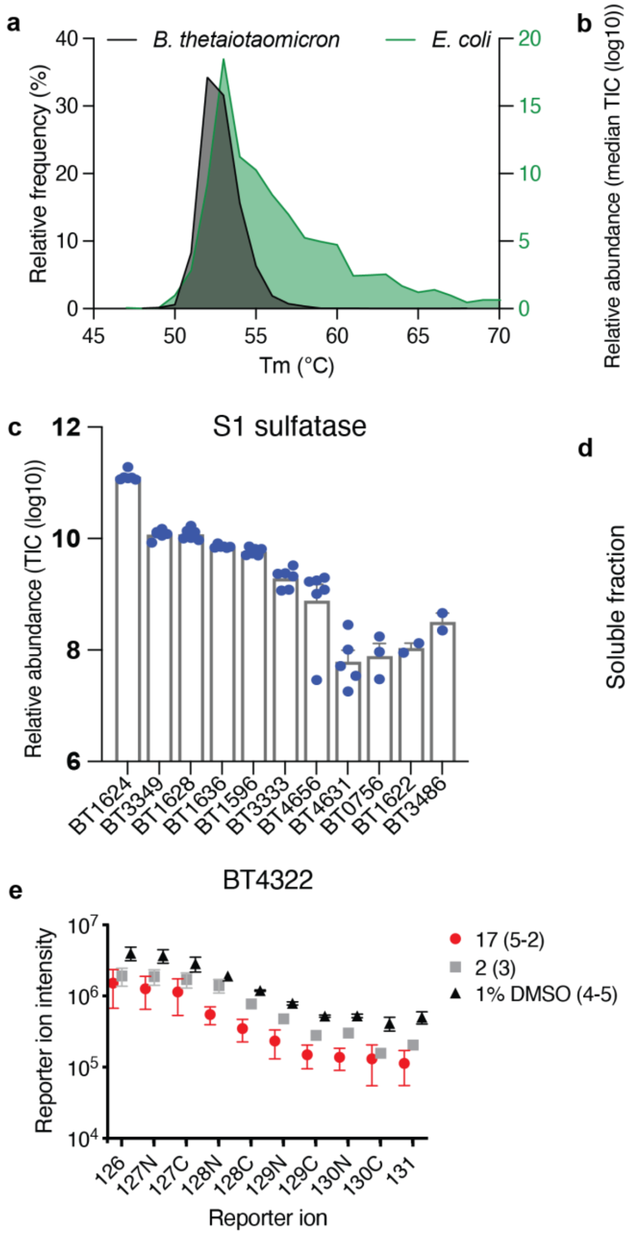

Supplementary figure 40. Global features of the thermal proteome profiling data
a. Distribution of the melting temperature (Tm) of Bacteroides thetaiotaomicron VPI-5482 (B. theta) lysate proteome and the whole cell E.coli proteome. **b.** Ranked protein abundance plot of all identified proteins in the 1%DMSO treated controls, S1 sulfatase are highlighted. **c.** Relative abundance of 11 S1 sulfatases identified by mass spectrometry for B. theta cultured in the presence of chondroitin sulfate A (n=6). (b) Melting curve profiles for all identified B. theta proteins in control (1%DMSO) treated lysate. The thermal stability profiles for each individual S1 sulfatases are highlighted in blue. **d.** Melting curve profiles for all identified B. theta proteins in control (1%DMSO) treated lysate. The thermal stability profiles for each individual S1 sulfatases are highlighted in blue. **e**. Extracted reporter ion intensities for the identified peptides assigned to BT4322 prior normalization and curve fitting. Data is presented as the summed intensity of the TMT reporter ions for all fragmentation spectra uniquely assigned to BT4322 for each temperature condition and treatment, presented as mean with range (n=2). Numbers indicate the arylsulfamate compound with the unique peptide count range per conditions between brackets.

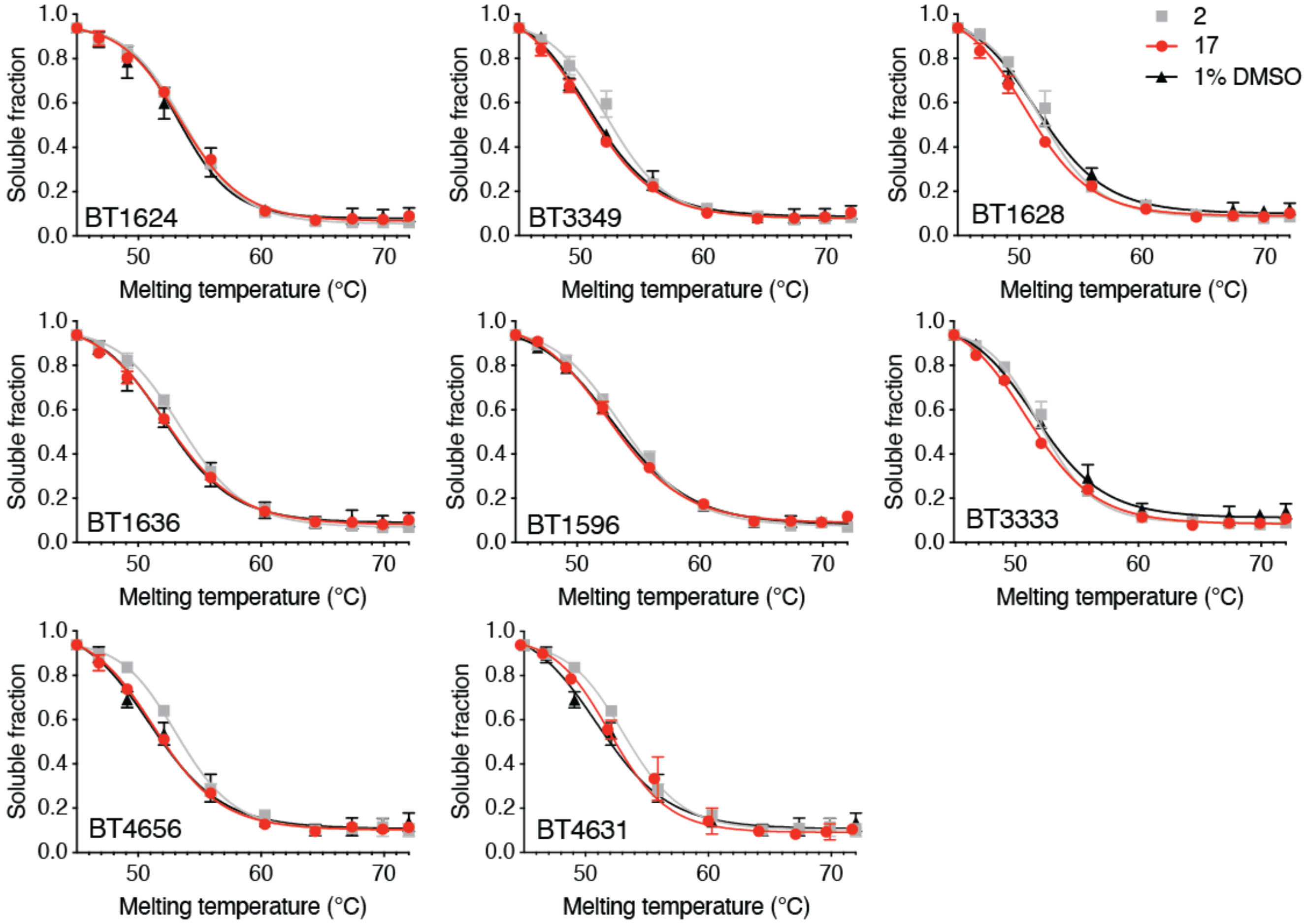

Supplementary figure 41. Global protein thermal stability analysis confirms that arysulfamate inhibitors do not alter the melting temperature of S1 sulfatases
2 and 17 do not directly interact with S1 sulfatases. Individual melt curve for selected S1 sulfatase for control and arysulfamate inhibitors 2 and 17 treated lysates (n=2 per treatment group).

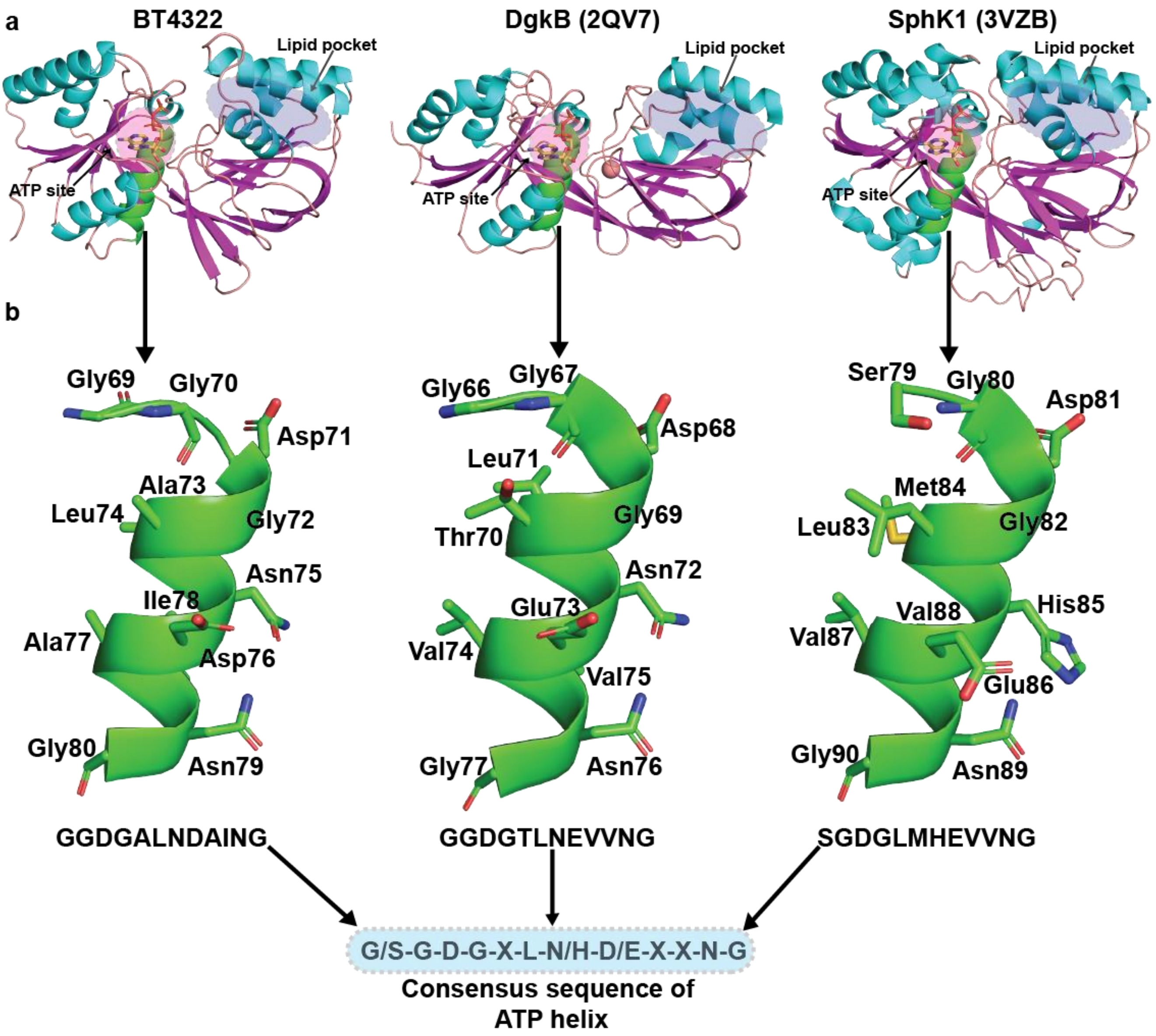

Supplementary figure 42. Structure of BT4322, SaDgKB, and SphK1 and comparison of the ATP helix

a. Tertiary structures of BT4322, SaDgKB, and SphK1 shown as with a helices, b sheets, and loops coloured cyan, magenta, and pink. The ‘ATP helix’ is coloured in green. **b.** An expanded view of the ATP helix and its sequence.

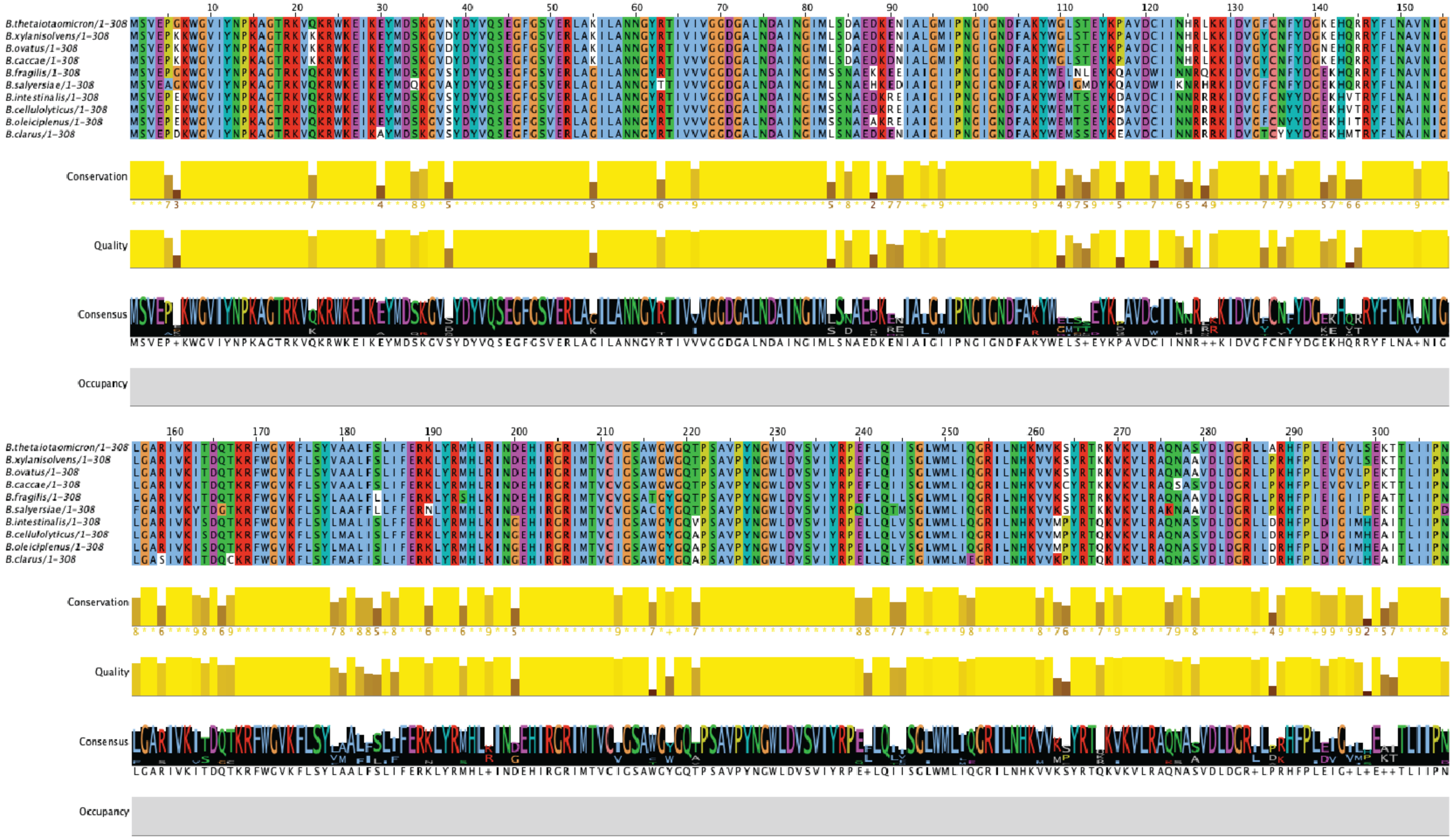

Supplementary figure 43. Sequence alignment of putative DAGKs from select Bacteroides species.
Sequences were aligned using the MAFFT online server and visualised using Jalview.

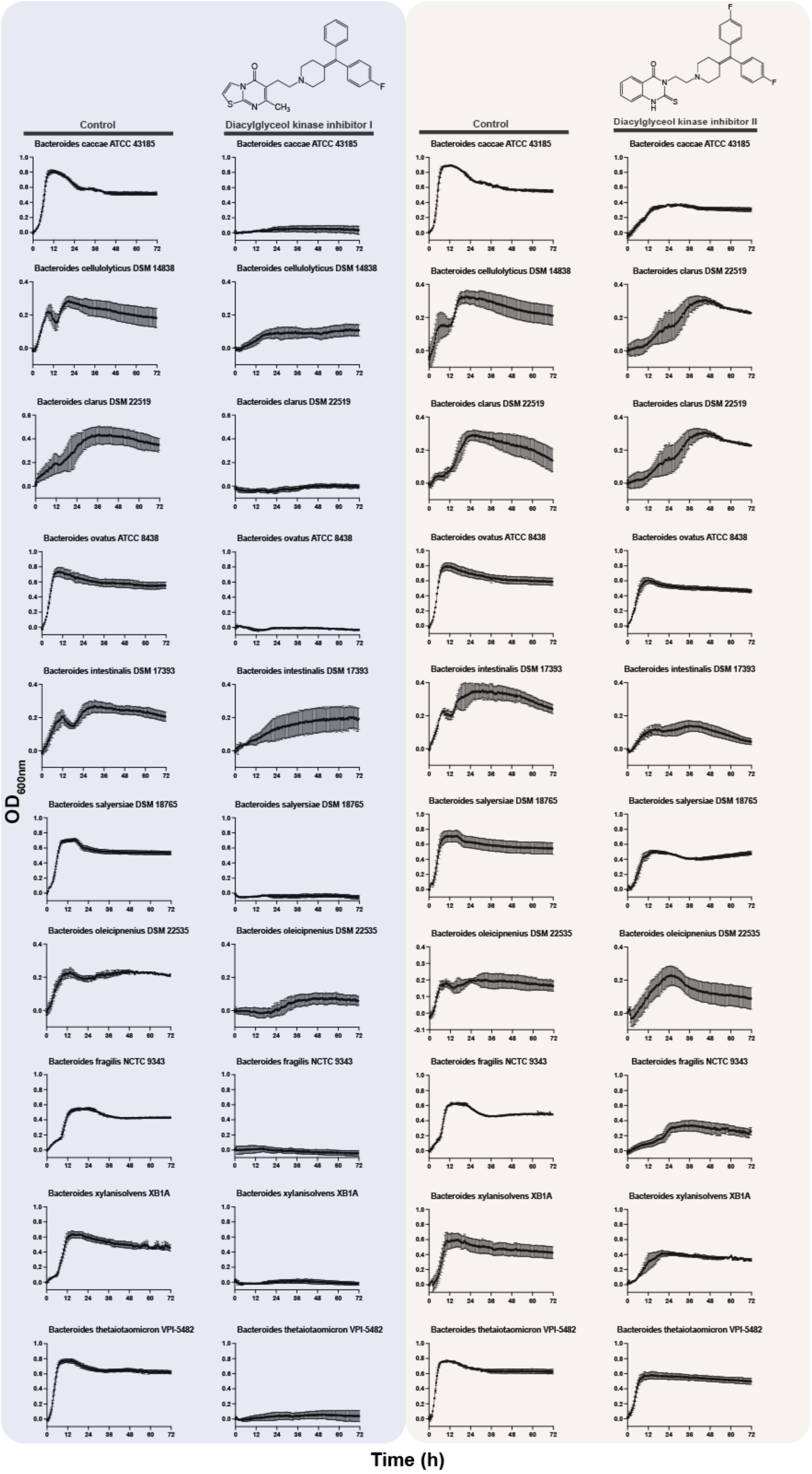

Control
Diacylglyceol kinase inhibitor I
Control
Diacylglyceol kinase inhibitor II
Bacteroides caccae ATCC 43185
Bacteroides cellulolyticus DSM 14838
Bacteroides clarus DSM 22519
Bacteroides ovatus ATCC 8438
Bacteroides intestinalis DSM 17393
Bacteroides salyersiae DSM 18765
Bacteroides oleicipnenius DSM 22535
Bacteroides fragilis NCTC 9343
Bacteroides xylanisolvens XB1A
Bacteroides thetaiotaomicron VPI-5482
OD600nm
Time (h)

Supplementary figure44. The effects of diacylglycerol kinase inhibitors on the growth of HGM Bacteroidota species.

Bacteroidota species were grown in BHI, 1 % DMSO, with 0.125 mM of diacylglycerol kinase inhibitor I (DAGKI-i) and 0.0625 mM diacylglycerol kinase inhibitor II (DAGKI-ii) and the effects on growth observed. Data are technical triplicates with the standard error of the mean.

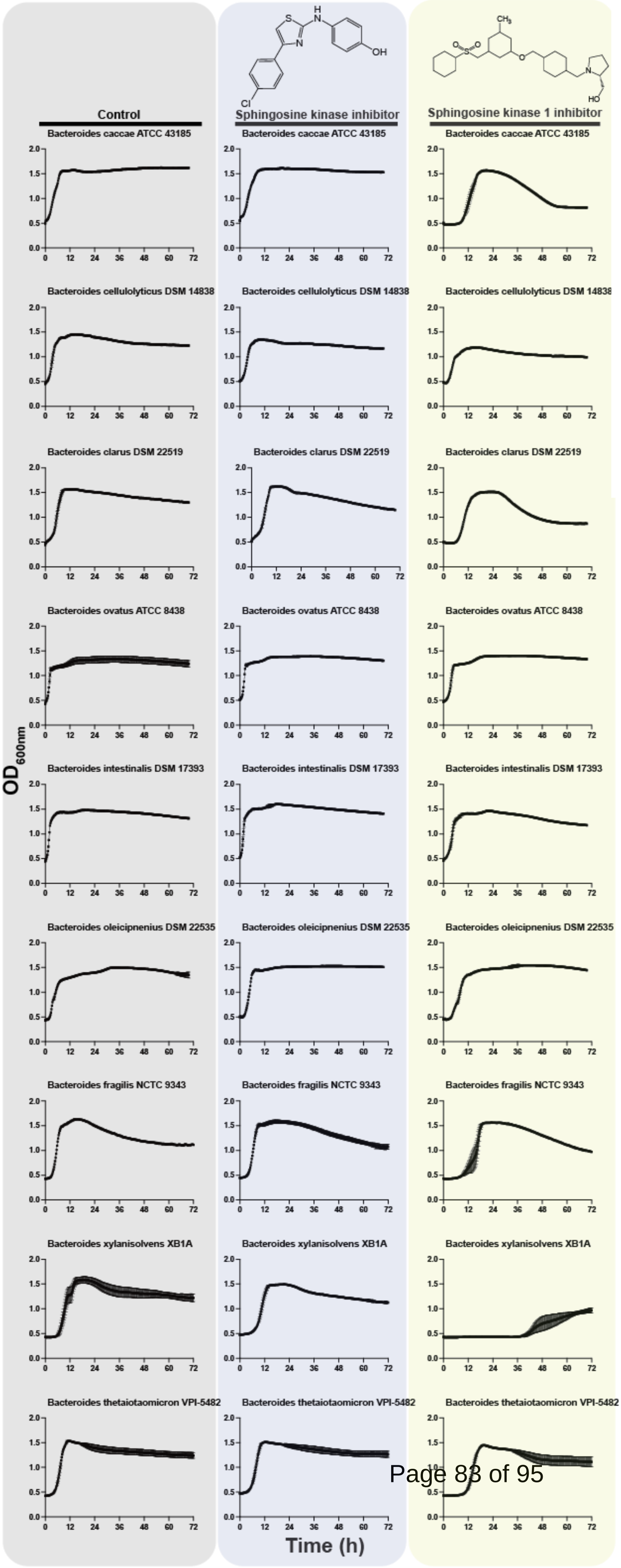

Supplementary figure 45. The effects of sphigosine kinase inhibitors on the growth of HGM Bacteroidota species

Bacteroidota species were grown in BHI, 1 % DMSO, with 0.1 mM of sphingosine kinase inhibitor (SKI) and 0.5 mM sphingosine kinase 1 inhibitor II and the effects on growth observed. Data are technical triplicates with the standard error of the mean.

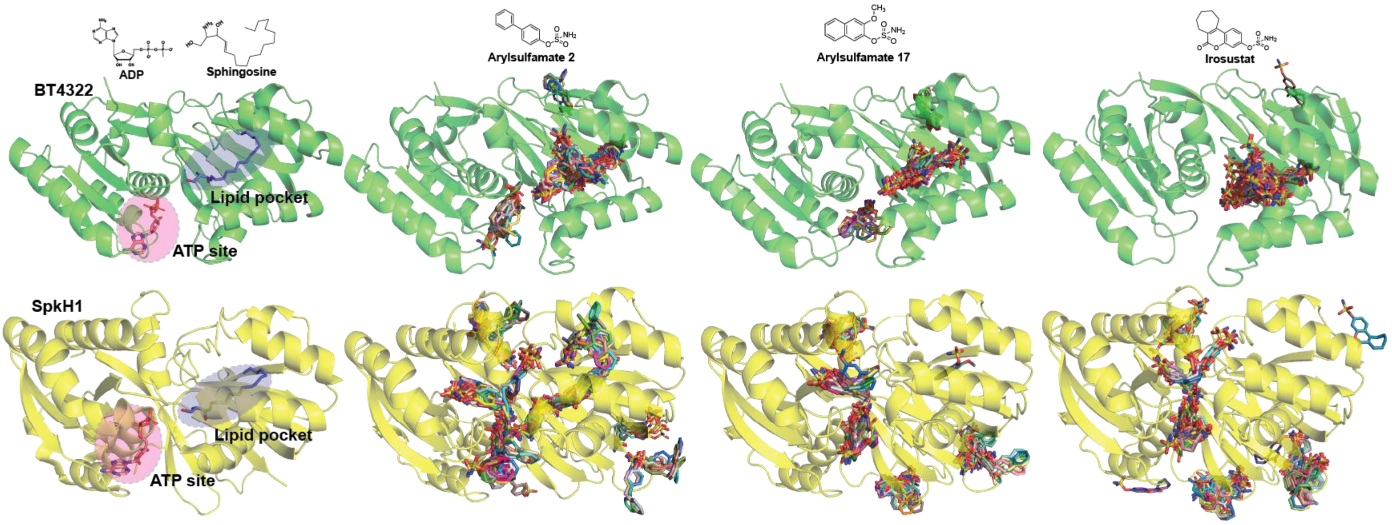

Supplementary figure 46. Blind docking of arylsulfamates onto BT4322 and SpkH1. The results of blind docking (no site specified) arylsulfamate inhibitors into the alphafold2 model of BT4322 (Q89ZQ4) and the crystal structure of SpKH1 (3VZB). It can be observed that all arylsulfamates preferentially cluster in, or near, the lipid binding pocket. For SpKH1 there appears to be no single preferred clustering site.

Compound	BHI			Glucose			Chondroitin sulfate C		
	Lag phase	*Growth rate*	*Max OD*	*Lag phase*	*Growth rate*	*Max OD*	*Lag phase*	*Growth rate*	*Max OD*
**1**	0.6139	0.0364	0.0444	0.7619	0.0098	0.0641	0.1456	0.0066	0.0255
**2**	ND	ND	ND	ND	ND	ND	ND	ND	ND
**3**	0.1482	0.0384	0.0503	0.8820	0.0890	0.2863	0.5727	0.0830	0.4548
**4**	0.1326	0.3574	0.6895	0.8541	0.2984	0.2756	0.4276	0.2872	0.4695
**5**	0.0803	0.0249	0.0012	0.0993	0.0005	0.0061	0.0616	0.0115	0.0117
**6**	0.5497	0.7241	0.2979	0.0651	0.2298	0.1024	0.2239	0.1954	0.9682
**7**	0.0245	0.0312	0.0174	0.2573	0.0366	0.0681	0.4966	0.0104	0.1935
**8**	0.0512	0.0028	0.0101	0.1649	0.0013	0.0033	0.4682	0.0339	0.0639
**9**	0.0947	0.0086	0.0109	0.2181	0.0181	0.0306	0.2210	0.0266	0.0037
**10**	ND	ND	ND	ND	ND	ND	ND	ND	ND
**11**	ND	ND	ND	ND	ND	ND	ND	ND	ND
**12**	ND	ND	ND	ND	ND	ND	ND	ND	ND
**13**	ND	ND	0.0020	0.0023	0.0001	0.0001	ND	ND	ND
**14**	ND	ND	ND	0.0167	0.0001	0.0001	ND	ND	ND
**15**	ND	ND	ND	ND	ND	ND	ND	ND	ND
**16**	ND	ND	ND	ND	ND	ND	ND	ND	ND
**17**	0.1466	0.0009	0.0001	ND	ND	ND	ND	ND	ND
**18**	0.7088	0.0081	0.0010	ND	ND	ND	ND	ND	ND

Supplementary table 1. Significant tests for brain heart infusion, glucose and chondroitin sulfate C growths.
For growth curves that displayed the classical features of a growth curve, and were measurable, a two-tailed unpaired t-test was performed to check for significance at a threshold of $p<0.5$. Green indicates a significant difference at the chosen threshold, orange no significant difference, and red indicates an analysis was not done due to the lack of a growth curve.

Compound	Heparin			Potato galactan			Larch arabinogalactan		
	Lag phase	*Growth rate*	*Max OD*	*Lag phase*	*Growth rate*	*Max OD*	*Lag phase*	*Growth rate*	*Max OD*
**1**	0.0062	0.7343	0.4803	0.4383	0.0715	0.0395	0.0100	0.9391	0.4074
**2**	ND	ND	ND	ND	ND	ND	ND	ND	ND
**3**	ND	ND	ND	0.0173	0.7817	0.3000	0.0010	0.6453	0.2329
**4**	ND	ND	ND	0.0205	0.8193	0.2273	0.6737	0.1880	0.1510
**5**	ND	ND	ND	0.4428	0.0541	0.0518	ND	ND	ND
**6**	0.5380	0.1845	0.3459	0.5185	0.5235	0.8915	0.2234	0.5538	0.4971
**7**	ND	ND	ND	0.0148	0.0360	0.2612	0.2024	0.0517	0.0170
**8**	ND	ND	ND	0.0205	0.0348	0.08515	ND	ND	ND
**9**	ND	ND	ND	0.0759	0.2361	0.0146	0.0139	0.1083	0.0034
**10**	ND	ND	ND	ND	ND	ND	ND	ND	ND
**11**	ND	ND	ND	ND	ND	ND	ND	ND	ND
**12**	ND	ND	ND	ND	ND	ND	ND	ND	ND
**13**	0.0034	0.0629	0.0012	ND	ND	ND	ND	ND	ND
**14**	ND	ND	ND	ND	ND	ND	ND	ND	ND
**15**	ND	ND	ND	ND	ND	ND	ND	ND	ND
**16**	ND	ND	ND	ND	ND	ND	ND	ND	ND
**17**	ND	ND	ND	ND	ND	ND	ND	ND	ND
**18**	0.0023	0.3306	0.0419	0.0512	0.0044	0.0004	ND	ND	ND

Supplementary table 2. Significant tests for heparin, larch arabinogalactan, and potato galactan growths.
For growth curves that displayed the classical features of a growth curve, and were measurable, a two-tailed unpaired t-test was performed to check for significance at a threshold of $p<0.5$. Green indicates a significant difference at the chosen threshold, orange no significant difference, and red indicates an analysis was not done due to the lack of a growth curve.

	PaAsta	HpSulf	BT3177	BT4656	Amuc1074	Amuc1033	BT1636	BT1622	Amuc0451	Amuc0491	BT3796	Amuc1755
**Control**	(-1.1 ± 0.02) x 10^{-4}	(-9.4 ± 0.1) x 10^{-6}	(2.7 ± 1.4) x 10^{-3}	(5.0 ± 8.8) x 10^{-5}	(4.2 ± 1.6) x 10^{-4}	(2.1 ± 0.09) x 10^{-4}	(3.2 ± 2.0) x 10^{-4}	(1.5 ± 2.0) x 10^{-4}	(5.4 ± 4.4) x 10^{-5}	(1.3 ± 0.3) x 10^{-4}	(4.1 ± 1.1) x 10^{-4}	(4.1 ± 0.06) x 10^{-4}
**WT**	(7.0 ± 0.1) x 10^{-3}	(5.8 ± 0.1) x 10^{-5}	(1.4 ± 0.05) x 10^{-3}	(3.3 ± 0.2) x 10^{-3}	(4.4 ± 0.2) x 10^{-3}	(1.5 ± 0.03) x 10^{-3}	(3.0 ± 0.1) x 10^{-3}	(7.0 ± 1.1) x 10^{-3}	(1.6 ± 0.01) x 10^{-3}	(2.5 ± 0.3) x 10^{-4}	(4.6 ± 0.2) x 10^{-3}	(2.9 ± 0.02) x 10^{-3}
**1**	NA	NA	(1.6 ± 0.01) x 10^{-3}	(3.9 ± 0.1) x 10^{-3}	(4.4 ± 0.1) x 10^{-3}	(1.5 ± 0.2) x 10^{-3}	(3.2 ± 0.2) x 10^{-3}	(7.4 ± 1.1) x 10^{-3}	(1.7 ± 0.08) x 10^{-3}	(2.1 ± 0.4) x 10^{-4}	(4.7 ± 0.3) x 10^{-3}	(2.5 ± 0.02) x 10^{-3}
**6**	(9.0 ± 0.01) x 10^{-3}	(6.7 ± 0.03) x 10^{-5}	(1.3 ± 0.09) x 10^{-3}	(3.7 ± 0.06) x 10^{-3}	(4.2 ± 0.5) x 10^{-3}	(1.6 ± 0.09) x 10^{-3}	(3.2 ± 0.2) x 10^{-3}	(6.9 ± 1.1) x 10^{-3}	(1.7 ± 0.09) x 10^{-3}	(2.6 ± 0.4) x 10^{-4}	(4.1 ± 0.2) x 10^{-3}	(2.9 ± 0.08) x 10^{-3}
**2**	NA	NA	(1.5 ± 0.02) x 10^{-3}	(4.0 ± 0.09) x 10^{-3}	(5.5 ± 0.6) x 10^{-3}	(1.7 ± 0.07) x 10^{-3}	(3.3 ± 0.2) x 10^{-3}	(7.5 ± 0.4) x 10^{-3}	(1.8 ± 0.07) x 10^{-3}	(2.6 ± 0.3) x 10^{-4}	(4.6 ± 0.3) x 10^{-3}	(2.8 ± 0.08) x 10^{-3}
**5**	NA	NA	(1.6 ± 0.1) x 10^{-3}	(3.8 ± 0.2) x 10^{-3}	(4.2 ± 0.3) x 10^{-3}	(1.7 ± 0.1) x 10^{-3}	(3.2 ± 0.2) x 10^{-3}	(6.9 ± 1.4) x 10^{-3}	(1.6 ± 0.09) x 10^{-3}	(2.5 ± 0.3) x 10^{-4}	(4.3 ± 0.3) x 10^{-3}	(2.9 ± 0.04) x 10^{-3}
**7**	(2.6 ± 0.05) x 10^{-3}	(5.0 ± 0.04) x 10^{-5}	(1.6 ± 0.2) x 10^{-3}	(3.8 ± 0.06) x 10^{-3}	(4.9 ± 0.6) x 10^{-3}	(1.6 ± 0.06) x 10^{-3}	(3.2 ± 0.3) x 10^{-3}	(7.4 ± 0.1) x 10^{-3}	(1.7 ± 0.09) x 10^{-3}	(2.2 ± 0.6) x 10^{-4}	(4.5 ± 0.1) x 10^{-3}	(2.7 ± 0.02) x 10^{-3}
**8**	(4.9 ± 0.3) x 10^{-3}	(4.7 ± 0.06) x 10^{-5}	(1.5 ± 0.01) x 10^{-3}	(3.8 ± 0.1) x 10^{-3}	(4.6 ± 0.3) x 10^{-3}	(1.9 ± 0.2) x 10^{-3}	(3.1 ± 0.1) x 10^{-3}	(7.7 ± 0.4) x 10^{-3}	(1.7 ± 0.07) x 10^{-3}	(3.0 ± 0.3) x 10^{-4}	(3.7 ± 0.2) x 10^{-3}	(2.8 ± 0.03) x 10^{-3}
**9**	NA	NA	(1.4 ± 0.04) x 10^{-3}	(3.7 ± 0.08) x 10^{-3}	(5.3 ± 0.1) x 10^{-3}	(1.8 ± 0.07) x 10^{-3}	(4.3 ± 0.9) x 10^{-3}	(8.7 ± 0.2) x 10^{-3}	(1.6 ± 0.04) x 10^{-3}	(2.8 ± 0.4) x 10^{-4}	(4.0 ± 0.5) x 10^{-3}	(2.7 ± 0.05) x 10^{-3}
**10**	NA	(0.3 ± 0.04) x 10^{-5}	(1.3 ± 0.04) x 10^{-3}	(3.1 ± 0.1) x 10^{-3}	(5.8 ± 0.2) x 10^{-3}	(2.3 ± 0.07) x 10^{-3}	(3.5 ± 0.2) x 10^{-3}	(8.9 ± 0.2) x 10^{-3}	(1.4 ± 0.01) x 10^{-3}	(2.5 ± 0.5) x 10^{-4}	(4.8 ± 0.3) x 10^{-3}	(2.4 ± 0.05) x 10^{-3}
**13**	NA	NA	(1.3 ± 0.05) x 10^{-3}	(4.1 ± 0.1) x 10^{-3}	(4.9 ± 0.2) x 10^{-3}	(1.9 ± 0.2) x 10^{-3}	(4.6 ± 1.0) x 10^{-3}	(8.0 ± 1.1) x 10^{-3}	(1.7 ± 0.01) x 10^{-3}	(2.3 ± 0.5) x 10^{-4}	(4.8 ± 0.3) x 10^{-3}	(3.1 ± 0.06) x 10^{-3}
**14**	NA	NA	(1.4 ± 0.05) x 10^{-3}	(4.0 ± 0.07) x 10^{-3}	(4.7 ± 0.07) x 10^{-3}	(1.4 ± 0.1) x 10^{-3}	(4.7 ± 0.6) x 10^{-3}	(8.4 ± 0.2) x 10^{-3}	(1.6 ± 0.05) x 10^{-3}	(2.7 ± 0.4) x 10^{-4}	(4.8 ± 0.2) x 10^{-3}	(2.9 ± 0.07) x 10^{-3}
**11**	NA	NA	(1.2 ± 0.01) x 10^{-3}	(4.0 ± 0.09) x 10^{-3}	(6.3 ± 1.0) x 10^{-3}	(2.0± 0.3) x 10^{-3}	(3.3 ± 0.2) x 10^{-3}	(7.3 ± 1.3) x 10^{-3}	(1.4 ± 0.05) x 10^{-3}	(2.6 ± 0.3) x 10^{-4}	(4.8 ± 0.2) x 10^{-3}	(2.6 ± 0.05) x 10^{-3}
**17**	NA	NA	(0.98 ± 0.04) x 10^{-3}	(4.4 ± 0.2) x 10^{-3}	(5.2 ± 0.6) x 10^{-3}	(2.1 ± 0.2) x 10^{-3}	(3.7 ± 0.7) x 10^{-3}	(9.0 ± 0.3) x 10^{-3}	(1.7 ± 0.05) x 10^{-3}	(2.6 ± 0.3) x 10^{-4}	(5.1 ± 0.2) x 10^{-3}	(3.5 ± 0.06) x 10^{-3}
**15**	NA	NA	NT	NT	NT	NT	NT	NT	(1.9 ± 0.05) x 10^{-3}	(2.2 ± 0.5) x 10^{-4}	(5.8 ± 0.4) x 10^{-3}	(2.9 ± 0.02) x 10^{-3}
**12**	NT	(2.4 ± 0.03) x 10^{-5}	NT	NT	NT	NT	NT	NT	NT	NT	NT	NT
**18**	NA	NA	(1.3 ± 0.02) x 10^{-3}	(4.3 ± 0.2) x 10^{-3}	(4.8 ± 0.3) x 10^{-3}	(1.6 ± 0.1) x 10^{-3}	(2.9 ± 0.2) x 10^{-3}	(9.4 ± 0.5) x 10^{-3}	(1.6 ± 0.03) x 10^{-3}	(2.8 ± 0.3) x 10^{-4}	(5.0 ± 0.2) x 10^{-3}	(2.7 ± 0.04) x 10^{-3}
**16**	NA	NA	(1.4 ± 0.03) x 10^{-3}	(4.5 ± 0.2) x 10^{-3}	(4.8 ± 0.4) x 10^{-3}	(1.7 ± 0.1) x 10^{-3}	(3.0 ± 0.4) x 10^{-3}	(9.2 ± 0.4) x 10^{-3}	(1.6 ± 0.06) x 10^{-3}	(2.2 ± 0.08) x 10^{-4}	(5.0 ± 0.4) x 10^{-3}	(4.6 ± 0.06) x 10^{-3}
**3**	*(3.8 ± 0.8) x 10^{-3}	NA	(1.2 ± 0.02) x 10^{-3}	(4.1 ± 0.1) x 10^{-3}	(4.8 ± 0.4) x 10^{-3}	(1.5 ± 0.1) x 10^{-3}	(3.2 ± 0.2) x 10^{-3}	(7.6 ± 0.1) x 10^{-3}	(1.6 ± 0.06) x 10^{-3}	(1.9± 0.07) x 10^{-4}	(4.6 ± 0.4) x 10^{-3}	(2.8 ± 0.3) x 10^{-3}
**4**	*(5.8 ± 0.4) x 10^{-3}	NA	(1.3 ± 0.03) x 10^{-3}	(4.0 ± 0.2) x 10^{-3}	(5.1 ± 0.3) x 10^{-3}	(1.5 ± 0.1) x 10^{-3}	(3.0 ± 0.1) x 10^{-3}	(8.2 ± 0.4) x 10^{-3}	(1.6 ± 0.04) x 10^{-3}	(2.1 ± 0.09) x 10^{-4}	(4.4 ± 0.3) x 10^{-3}	(2.9 ± 0.2) x 10^{-3}

Supplementary table 3. Kinetic rates of sulfatase when arylsulfamates were included in the assay conditions.
The concentration of arylsulfamate was always 1 mM. For the steroid sulfatases the substrate *para*-nitrophenol sulfate was at a concentration of 1 mM, whilst for carbohydrate sulfatases substrate concentration was 1 μM. NA means no activity when compared to the control. NT means not tested. Numbers in the left most column refer to the arylsulfamate in Figure 1c added to the reaction; control represents substrate only whilst WT is substrate plus enzyme without an arylsulfamate inhibitor. Rates are μM product/min.

	PaAsta	HpSulf	BT3177	BT4656	Amuc1074	Amuc1033	BT1636	BT1622	Amuc0451	Amuc0491	BT3796	Amuc1755
**Control**	$(-6.57 \pm 1.53) \times 10^{-5}$	$(-7.9 \pm 2.7) \times 10^{-6}$	$(1.9 \pm 1.4) \times 10^{-4}$	$(2.3 \pm 1.9) \times 10^{-3}$	$(2.8 \pm 0.2) \times 10^{-4}$	$(0.6 \pm 1.0) \times 10^{-4}$	$(8.8 \pm 2.7) \times 10^{-5}$	$(1.2 \pm 1.7) \times 10^{-4}$	$(7.6 \pm 3.2) \times 10^{-5}$	$(7.6 \pm 2.5) \times 10^{-5}$	$(5.3 \pm 0.5) \times 10^{-4}$	$(2.9 \pm 0.6) \times 10^{-4}$
**WT**	$(7.5 \pm 0.2) \times 10^{-3}$	$(7.5 \pm 0.2) \times 10^{-4}$	$(2.4 \pm 0.1) \times 10^{-3}$	$(3.3 \pm 0.05) \times 10^{-3}$	$(4.3 \pm 0.1) \times 10^{-3}$	$(2.2 \pm 0.1) \times 10^{-3}$	$(1.7 \pm 0.05) \times 10^{-3}$	$(6.2 \pm 0.2) \times 10^{-3}$	$(4.0 \pm 0.05) \times 10^{-3}$	$(2.0 \pm 0.2) \times 10^{-4}$	$(4.9 \pm 0.2) \times 10^{-3}$	$(2.7 \pm 0.09) \times 10^{-3}$
**1**	NA	NA	$(2.6 \pm 0.1) \times 10^{-3}$	$(4.0 \pm 0.1) \times 10^{-3}$	$(4.4 \pm 0.09) \times 10^{-3}$	$(2.0 \pm 0.05) \times 10^{-3}$	$(1.8 \pm 0.05) \times 10^{-3}$	$(5.8 \pm 0.3) \times 10^{-3}$	$(3.0 \pm 0.06) \times 10^{-3}$	$(2.2 \pm 0.08) \times 10^{-4}$	$(5.1 \pm 0.2) \times 10^{-3}$	$(5.6 \pm 0.2) \times 10^{-3}$
**6**	$(5.9 \pm 0.1) \times 10^{-3}$	$(6.4 \pm 0.1) \times 10^{-4}$	$(2.7 \pm 0.09) \times 10^{-3}$	$(4.1 \pm 0.2) \times 10^{-3}$	$(4.5 \pm 0.1) \times 10^{-3}$	$(2.1 \pm 0.07) \times 10^{-3}$	$(2.0 \pm 0.01) \times 10^{-3}$	$(6.0 \pm 0.2) \times 10^{-3}$	$(3.9 \pm 0.06) \times 10^{-3}$	$(2.1 \pm 0.2) \times 10^{-4}$	$(4.8 \pm 0.2) \times 10^{-3}$	$(2.9 \pm 0.06) \times 10^{-3}$
**2**	NA	NA	$(2.7 \pm 0.2) \times 10^{-3}$	$(4.0 \pm 0.06) \times 10^{-3}$	$(4.2 \pm 0.09) \times 10^{-3}$	$(2.2 \pm 0.1) \times 10^{-3}$	$(1.8 \pm 0.04) \times 10^{-3}$	$(5.9 \pm 0.2) \times 10^{-3}$	$(3.5 \pm 0.1) \times 10^{-3}$	$(1.9 \pm 0.3) \times 10^{-4}$	$(4.2 \pm 0.2) \times 10^{-3}$	$(3.7 \pm 0.1) \times 10^{-3}$
**5**	NA	NA	$(2.4 \pm 0.1) \times 10^{-3}$	$(4.2 \pm 0.06) \times 10^{-3}$	$(4.2 \pm 0.09) \times 10^{-3}$	$(2.1 \pm 0.09) \times 10^{-3}$	$(2.8 \pm 0.02) \times 10^{-3}$	$(5.5 \pm 0.3) \times 10^{-3}$	$(3.8 \pm 0.05) \times 10^{-3}$	$(2.3 \pm 0.2) \times 10^{-4}$	$(5.2 \pm 0.2) \times 10^{-3}$	$(6.4 \pm 0.2) \times 10^{-3}$
**7**	$(5.7 \pm 0.1) \times 10^{-3}$	$(7.3 \pm 0.7) \times 10^{-4}$	$(2.6 \pm 0.09) \times 10^{-3}$	$(3.9 \pm 0.3) \times 10^{-3}$	$(4.4 \pm 0.09) \times 10^{-3}$	$(2.1 \pm 0.05) \times 10^{-3}$	$(2.0 \pm 0.02) \times 10^{-3}$	$(6.9 \pm 0.3) \times 10^{-3}$	$(4.0 \pm 0.05) \times 10^{-3}$	$(2.0 \pm 0.08) \times 10^{-4}$	$(5.1 \pm 0.2) \times 10^{-3}$	$(2.8 \pm 0.09) \times 10^{-3}$
**8**	$(6.3 \pm 0.7) \times 10^{-3}$	$(5.4 \pm 0.5) \times 10^{-4}$	$(2.5 \pm 0.1) \times 10^{-3}$	$(4.1 \pm 0.1) \times 10^{-3}$	$(4.4 \pm 0.1) \times 10^{-3}$	$(2.0 \pm 0.07) \times 10^{-3}$	$(2.6 \pm 0.08) \times 10^{-3}$	$(6.1 \pm 0.3) \times 10^{-3}$	$(3.8 \pm 0.06) \times 10^{-3}$	$(2.1 \pm 0.2) \times 10^{-4}$	$(5.4 \pm 0.2) \times 10^{-3}$	$(5.2 \pm 0.2) \times 10^{-3}$
**9**	NA	NA	$(2.6 \pm 0.2) \times 10^{-3}$	$(4.1 \pm 0.3) \times 10^{-3}$	$(4.3 \pm 0.1) \times 10^{-3}$	$(2.0 \pm 0.1) \times 10^{-3}$	$(2.3 \pm 0.02) \times 10^{-3}$	$(6.2 \pm 0.2) \times 10^{-3}$	$(3.3 \pm 0.03) \times 10^{-3}$	$(2.5 \pm 0.3) \times 10^{-4}$	$(5.2 \pm 0.2) \times 10^{-3}$	$(5.7 \pm 0.3) \times 10^{-3}$
**10**	NA	NA	$(2.7 \pm 0.2) \times 10^{-3}$	$(4.1 \pm 0.08) \times 10^{-3}$	$(4.3 \pm 0.1) \times 10^{-3}$	$(2.2 \pm 0.1) \times 10^{-3}$	$(1.7 \pm 0.1) \times 10^{-3}$	$(6.6 \pm 0.1) \times 10^{-3}$	$(3.7 \pm 0.04) \times 10^{-3}$	$(1.8 \pm 0.1) \times 10^{-4}$	$(5.1 \pm 0.2) \times 10^{-3}$	$(5.0 \pm 0.2) \times 10^{-3}$
**13**	NA	NA	$(2.7 \pm 0.1) \times 10^{-3}$	$(4.3 \pm 0.06) \times 10^{-3}$	$(4.4 \pm 0.1) \times 10^{-3}$	$(2.2 \pm 0.06) \times 10^{-3}$	$(2.3 \pm 0.06) \times 10^{-3}$	$(6.6 \pm 0.2) \times 10^{-3}$	$(3.9 \pm 0.07) \times 10^{-3}$	$(1.9 \pm 0.1) \times 10^{-4}$	$(5.6 \pm 0.2) \times 10^{-3}$	$(3.6 \pm 0.1) \times 10^{-3}$
**14**	NA	NA	$(2.4 \pm 0.2) \times 10^{-3}$	$(4.0 \pm 0.2) \times 10^{-3}$	$(4.4 \pm 0.1) \times 10^{-3}$	$(2.1 \pm 0.08) \times 10^{-3}$	$(1.9 \pm 0.01) \times 10^{-3}$	$(6.7 \pm 0.2) \times 10^{-3}$	$(3.2 \pm 0.3) \times 10^{-3}$	$(1.9 \pm 0.2) \times 10^{-4}$	$(5.6 \pm 0.2) \times 10^{-3}$	$(3.3 \pm 0.1) \times 10^{-3}$
**11**	NA	NA	$(2.6 \pm 0.1) \times 10^{-3}$	$(4.3 \pm 0.1) \times 10^{-3}$	$(4.7 \pm 0.09) \times 10^{-3}$	$(2.1 \pm 0.1) \times 10^{-3}$	$(1.8 \pm 0.03) \times 10^{-3}$	$(6.6 \pm 0.3) \times 10^{-3}$	$(2.3 \pm 0.5) \times 10^{-3}$	$(2.0 \pm 0.1) \times 10^{-4}$	$(5.4 \pm 0.2) \times 10^{-3}$	$(3.8 \pm 0.2) \times 10^{-3}$
**17**	NA	NA	$(2.5 \pm 0.2) \times 10^{-3}$	$(4.3 \pm 0.1) \times 10^{-3}$	$(4.6 \pm 0.1) \times 10^{-3}$	$(2.0 \pm 0.1) \times 10^{-3}$	$(1.9 \pm 0.05) \times 10^{-3}$	$(6.4 \pm 0.2) \times 10^{-3}$	$(1.7 \pm 0.04) \times 10^{-3}$	$(2.0 \pm 0.1) \times 10^{-4}$	$(5.8 \pm 0.3) \times 10^{-3}$	$(4.8 \pm 0.09) \times 10^{-3}$
**15**	NA	NA	$(2.6 \pm 0.08) \times 10^{-3}$	$(4.5 \pm 0.2) \times 10^{-3}$	$(4.8 \pm 0.1) \times 10^{-3}$	$(2.2 \pm 0.03) \times 10^{-3}$	$(1.7 \pm 0.04) \times 10^{-3}$	$(5.6 \pm 0.3) \times 10^{-3}$	$(3.4 \pm 0.03) \times 10^{-3}$	$(2.0 \pm 0.1) \times 10^{-4}$	$(5.5 \pm 0.2) \times 10^{-3}$	$(3.3 \pm 0.1) \times 10^{-3}$
**12**	NT	NA	$(2.6 \pm 0.1) \times 10^{-3}$	$(4.4 \pm 0.1) \times 10^{-3}$	$(4.8 \pm 0.1) \times 10^{-3}$	$(2.1 \pm 0.06) \times 10^{-3}$	$(1.9 \pm 0.07) \times 10^{-3}$	$(6.5 \pm 0.2) \times 10^{-3}$	$(3.5 \pm 0.06) \times 10^{-3}$	$(2.0 \pm 0.1) \times 10^{-4}$	$(5.6 \pm 0.2) \times 10^{-3}$	$(3.6 \pm 0.07) \times 10^{-3}$
**18**	NA	NA	$(2.6 \pm 0.1) \times 10^{-3}$	$(4.4 \pm 0.2) \times 10^{-3}$	$(4.6 \pm 0.1) \times 10^{-3}$	$(2.2 \pm 0.05) \times 10^{-3}$	$(2.3 \pm 0.02) \times 10^{-3}$	$(6.6 \pm 0.3) \times 10^{-3}$	NT	NT	NT	NT
**16**	NA	NA	$(2.6 \pm 0.1) \times 10^{-3}$	$(4.2 \pm 0.1) \times 10^{-3}$	$(4.6 \pm 0.1) \times 10^{-3}$	$(2.2 \pm 0.06) \times 10^{-3}$	$(2.3 \pm 0.02) \times 10^{-3}$	$(6.8 \pm 0.2) \times 10^{-3}$	$(3.0 \pm 0.02) \times 10^{-3}$	$(2.2 \pm 0.1) \times 10^{-4}$	$(6.4 \pm 0.2) \times 10^{-3}$	$(4.3 \pm 0.09) \times 10^{-3}$
**3**	NA	NA	$(2.4 \pm 0.1) \times 10^{-3}$	$(4.2 \pm 0.1) \times 10^{-3}$	$(4.6 \pm 0.1) \times 10^{-3}$	$(2.2 \pm 0.06) \times 10^{-3}$	$(1.9 \pm 0.02) \times 10^{-3}$	$(5.3 \pm 0.1) \times 10^{-3}$	$(2.6 \pm 0.08) \times 10^{-3}$	$(2.1 \pm 0.1) \times 10^{-4}$	$(5.7 \pm 0.2) \times 10^{-3}$	$(2.7 \pm 0.09) \times 10^{-3}$
**4**	NA	NA	$(2.2 \pm 0.1) \times 10^{-3}$	$(4.3 \pm 0.1) \times 10^{-3}$	$(4.7 \pm 0.09) \times 10^{-3}$	$(2.2 \pm 0.05) \times 10^{-3}$	$(2.0 \pm 0.05) \times 10^{-3}$	$(6.3 \pm 0.2) \times 10^{-3}$	$(3.2 \pm 0.06) \times 10^{-3}$	$(2.1 \pm 0.1) \times 10^{-4}$	$(6.2 \pm 0.2) \times 10^{-3}$	$(4.3 \pm 0.1) \times 10^{-3}$

Supplementary table 4. Kinetic rates of sulfatase when sulfatases were pre-incubated with arylsulfamates then assayed.
The concentration of arylsulfamate used for preincubation was always 1 mM and the enzyme concentration 25 - 50 μM. For the steroid sulfatases the substrate *para*-nitrophenol sulfate was at a concentration of 1 mM, whilst for carbohydrate sulfatases substrate concentration was 1 μM. NA means no activity when compared to the control. NT means not tested. Numbers in the left most column refer to the arylsulfamate in Figure 1c added to the reaction; control represents substrate only whilst WT is substrate plus enzyme without an arylsulfamate inhibitor. Rates are μM product/min.

	BT1636	
	**In assay**	**Pre-incubated**
**Control**	(2.7 ± 0.2) x 10^{-2}	(3.1 ± 2.3) x10^{-2}
**WT**	0.58 ± 0.07	1.4 ± 0.06
**22**	0.68 ± 0.02	NT
**23**	NT	0.49 ± 0.01
**19**	0.72 ± 0.09	1.3 ± 0.08
**24**	0.68 ± 0.03	0.60± 0.01
**WT**	-	1.2 ± 0.06
**20**	0.63 ± 0.03	1.2 ± 0.08
**21**	0.58 ± 0.03	1.2 ± 0.04
**25**	0.56 ± 0.02	1.2 ± 0.06

Supplementary table 5. Kinetic rate of BT1636$^{3S\text{-}Gal}$ assayed against BODIPY labelled 3S-galactose sulfate with various carbohydrate and aryl- sulfonates/sulfamates.
BT1636$^{3S\text{-}Gal}$ was assayed against 1 μM BODIPY labelled 3S-galactose with 1 mM of **with** various carbohydrate and aryl- sulfonates/sulfamates the assay, or after BT1636$^{3S\text{-}Gal}$ had been incubated with 1 mM of the same compounds; concentrations of 300 nM and 285 nM of BT1636$^{3S\text{-}Gal}$ were used, respectively. The assay was performed in 100 mM of MES pH 6.0 with 5 % DMSO, 150 mM NaCl, 0.02% (v/v) Brij-35 and 5 mM $CaCl_2$. Assays were performed in triplicate. Rates are fluorescence of product/min. The values in green indicate 5 % was not present.

	0.00 mM	0.01 mM	0.10 mM	1.00 mM
**3S-Gal**	54.6 ± 0.06	54.7 ± 0.03	55.6 ± 0.04	57.6 ± 0.04
**20**	54.6 ± 0.06	54.6 ± 0.03	54.6 ± 0.02	54.4 ± 0.03
**21**	54.6 ± 0.06	54.6 ± 0.07	54.5 ± 0.02	54.4 ± 0.01
**25**	54.6 ± 0.06	54.5 ± 0.01	54.6 ± 0.07	54.1 ± 0.07
**19**	53.4 ± 0.05	52.0 ± 0.03	51.4 ± 0.11	49.9 ± 0.12
**24**	53.4 ± 0.05	52.9 ± 0.05	52.7 ± 0.04	52.0 ± 0.07

Supplementary table 6. Thermal melt values of BT1636$^{3S\text{-}Gal}$ in the absence and presence of various carbohydrate and aryl- sulfonates/sulfamates.

BT1636$^{3S\text{-}Gal}$, at a concentration of 5 μM, was incubated with no compound, and varying concentrations and the effect on its melting temperature monitored, a shift in melting temperature is indicative of an interaction. The assays were performed in 100 mM of BTP pH 7.0 with 5 % DMSO, 150 mM NaCl. Assays were performed in triplicate.

Description	Scientific name	Max Score	Total score	Query coverage	E-value	Identity (%)	Acc. Length	Accession no.
diacylglycerol kinase family protein	Bacteroides xylanisolvens XBA1	619	619	100%	0.0	96.75%	308	WP_004316677.1
YegS/Rv2252/BmrU family lipid kinase	Bacteroides ovatus ATCC 8438	615	615	100%	0.0	96.10%	308	WP_004301802.1
YegS/Rv2252/BmrU family lipid kinase	Bacteroides caccae ATCC 43185	610	610	100%	0.0	95.13%	308	WP_005677430.1
diacylglycerol kinase family protein	Bacteroides fragilis NCTC 9343	565	565	100%	0.0	88.64%	308	WP_010992246.1
diacylglycerol kinase family protein	Bacteroides intestinalis DSM 17393	548	548	100%	0.0	84.42%	308	WP_007666434.1
diacylglycerol kinase family protein	Bacteroides cellulolyticus DSM 14838	546	546	100%	0.0	83.77%	308	WP_007212750.1
diacylglycerol kinase family protein	Bacteroides oleiciplenus DSM 22535	545	545	100%	0.0	83.77%	308	WP_009129050.1
diacylglycerol kinase family protein	Bacteroides clarus DSM 22519	543	543	100%	0.0	83.77%	308	WP_009122527.1
YegS/Rv2252/BmrU family lipid kinase	Bacteroides salyersiae DSM 18765	537	537	100%	0.0	83.44%	308	WP_007478527.1

Supplementary table 7. Conservation of BT4322 in select Bacteroidota species of the human gut microbiota.
BT4322 was used as the query against the genomes of the 9 other *Bacteroidoes* species from Figure 5 against which arylsulfamates 3 and 12 were tested. The BLASTp search was done using default settings with the blastp algorithm accessed through the NCBI interface.

Dataset 1. Results output of the thermal proteome profiling analysis of compound 2 and 17

Dataset 2. Lipidomic analysis of *B. theta* grown in BHI in the presence of arylsulfamate 2.

References

1 Mateus, A. *et al.* Thermal proteome profiling in bacteria: probing protein state in vivo. *Mol Syst Biol* **14**, e8242 (2018). https://doi.org:10.15252/msb.20188242

2 Moggridge, S., Sorensen, P. H., Morin, G. B. & Hughes, C. S. Extending the Compatibility of the SP3 Paramagnetic Bead Processing Approach for Proteomics. *J Proteome Res* **17**, 1730-1740 (2018). https://doi.org:10.1021/acs.jproteome.7b00913

3 Smits, A. H. *et al.* Biological plasticity rescues target activity in CRISPR knock outs. *Nat Methods* **16**, 1087-1093 (2019). https://doi.org:10.1038/s41592-019-0614-5

4 Kong, A. T., Leprevost, F. V., Avtonomov, D. M., Mellacheruvu, D. & Nesvizhskii, A. I. MSFragger: ultrafast and comprehensive peptide identification in mass spectrometry-based proteomics. *Nat Methods* **14**, 513-520 (2017). https://doi.org:10.1038/nmeth.4256

5 da Veiga Leprevost, F. *et al.* Philosopher: a versatile toolkit for shotgun proteomics data analysis. *Nat Methods* **17**, 869-870 (2020). https://doi.org:10.1038/s41592-020-0912-y

6 Franken, H. *et al.* Thermal proteome profiling for unbiased identification of direct and indirect drug targets using multiplexed quantitative mass spectrometry. *Nat Protoc* **10**, 1567-1593 (2015). https://doi.org:10.1038/nprot.2015.101

7 Grosdidier, A., Zoete, V. & Michielin, O. Fast docking using the CHARMM force field with EADock DSS. *J Comput Chem* **32**, 2149-2159 (2011). https://doi.org:10.1002/jcc.21797

8 Lebedev, A. A. *et al.* JLigand: a graphical tool for the CCP4 template-restraint library. *Acta crystallographica. Section D, Biological crystallography* **68**, 431-440 (2012). https://doi.org:10.1107/S090744491200251X

9 Webb, J. *et al.* Systems Analyses Reveal the Resilience of Escherichia coli Physiology during Accumulation and Export of the Nonnative Organic Acid Citramalate. *mSystems* **4** (2019). https://doi.org:10.1128/mSystems.00187-19

10 Mirretta Barone, C. *et al.* Spatially resolved lipidomics shows conditional transfer of lipids produced by Bacteroides thetaiotaomicron into the mouse gut. *Cell host & microbe* **32**, 1025-1036 e1025 (2024). https://doi.org:10.1016/j.chom.2024.04.021

11 Koelmel, J. P. *et al.* LipidMatch: an automated workflow for rule-based lipid identification using untargeted high-resolution tandem mass spectrometry data. *BMC Bioinformatics* **18**, 331 (2017). https://doi.org:10.1186/s12859-017-1744-3

12 Kind, T. *et al.* LipidBlast in silico tandem mass spectrometry database for lipid identification. *Nat Methods* **10**, 755-758 (2013). https://doi.org:10.1038/nmeth.2551

13 Duhrkop, K. *et al.* SIRIUS 4: a rapid tool for turning tandem mass spectra into metabolite structure information. *Nat Methods* **16**, 299-302 (2019). https://doi.org:10.1038/s41592-019-0344-8

14 Birchenough, G. M. H. *et al.* Muc2-dependent microbial colonization of the jejunal mucus layer is diet sensitive and confers local resistance to enteric pathogen infection. *Cell Rep* **42**, 112084 (2023). https://doi.org:10.1016/j.celrep.2023.112084

15 Denehy, E., White, J. M. & Williams, S. J. Ground state structures of sulfate monoesters and sulfamates reveal similar reaction coordinates for sulfuryl and sulfamyl transfer. *Chem. Commun.*, 314-316 (2006).

16 Molander, G. A. & Shin, I. Pd-catalyzed Suzuki-Miyaura cross-coupling reactions between sulfamates and potassium Boc-protected aminomethyltrifluoroborates. *Org. Lett.* **15**, 2534-2537 (2013). https://doi.org:10.1021/ol401021x

17 Winum, J. Y. *et al.* Carbonic anhydrase inhibitors. Inhibition of cytosolic isozymes I and II and transmembrane, tumor-associated isozyme IX with sulfamates including EMATE also acting as steroid sulfatase inhibitors. *J. Med. Chem.* **46**, 2197-2204 (2003). https://doi.org:10.1021/jm021124k

18 Matsuo, K. *et al.* Nickel-Catalyzed Hydrodeoxygenation of Aryl Sulfamates with Alcohols as Mild Reducing Agents. *Synthesis* **53**, 4449-4460 (2021). https://doi.org:10.1055/a-1548-8362

19 Bojarová, P. *et al.* Direct evidence for ArO-S bond cleavage upon inactivation of *Pseudomonas aeruginosa* arylsulfatase by aryl sulfamates. *Chembiochem : a European journal of chemical biology* **9**, 613-623 (2008).
